# Use of study-specific MOE-like estimates to prioritize health effects from chemical exposure for analysis in human health assessments

**DOI:** 10.1016/j.envint.2020.105986

**Published:** 2020-08-30

**Authors:** Kevin Hobbie, Kan Shao, Cara Henning, William Mendez, Janice S. Lee, Ila Cote, Ingrid L. Druwe, J. Allen Davis, Jeffrey S. Gift

**Affiliations:** a ICF, 9300 Lee Highway, Fairfax, VA 22031-1207 USA; b ICF, 2635 Meridian Parkway Suite 200, Durham, NC 27713, USA; c Department of Environmental and Occupational Health, Indiana University, Bloomington, IN, USA; d CPHEA, U.S. Environmental Protection Agency, Research Triangle Park, NC, USA; e CPHEA, U.S. Environmental Protection Agency, Cincinnati, OH, USA

**Keywords:** Case-control, Cohort, Margin of exposure, Benchmark dose, Inorganic arsenic

## Abstract

There are unique challenges in estimating dose-response with chemicals that are associated with multiple health outcomes and numerous studies. Some studies are more suitable than others for quantitative dose-response analyses. For such chemicals, an efficient method of screening studies and endpoints to identify suitable studies and potentially important health effects for dose-response modeling is valuable. Using inorganic arsenic as a test case, we developed a tiered approach that involves estimating study-specific margin of exposure (MOE)-like unitless ratios for two hypothetical scenarios. These study-specific unitless ratios are derived by dividing the exposure estimated to result in a 20% increase in relative risk over the background exposure (RRE_20_) by the background exposure, as estimated in two different ways. In our case study illustration, separate study-specific ratios are derived using estimates of United States population background exposure (RRB-US) and the mean study population reference group background exposure (RRB-SP). Systematic review methods were used to identify and evaluate epidemiologic studies, which were categorized based on study design (case-control, cohort, cross-sectional), various study quality criteria specific to dose-response analysis (number of dose groups, exposure ascertainment, exposure uncertainty), and availability of necessary dose-response data. Both case-control and cohort studies were included in the RRB analysis. The RRE_20_ estimates were derived by modeling effective counts of cases and controls estimated from study-reported adjusted odds ratios and relative risks. Using a broad (but not necessarily comprehensive) set of epidemiologic studies of multiple health outcomes selected for the purposes of illustrating the RRB approach, this test case analysis would suggest that diseases of the circulatory system, bladder cancer, and lung cancer may be arsenic health outcomes that warrant further analysis. This is suggested by the number of datasets from adequate dose-response studies demonstrating an effect with RRBs close to 1 (i.e., RRE_20_ values close to estimated background arsenic exposure levels).

## 1. Introduction

The evaluation of the potential human health effects of chemicals, like arsenic, that have an extremely large volume of datasets and the wide variance in data quality represented across studies would benefit from an efficient screening approach that helps to narrow the focus of dose-response analyses to studies and health outcomes that are of the most concern for risk assessment. The screening approach described in this paper allows for the use of exposure- or dose-response data^2^ provided in studies without the need for resource- and time-consuming adjustments often performed on a set of critical studies, such as converting reported exposure metrics to a more relevant intake dose metric (e.g., μg/kg-day) and performing complex statistical dose-response modeling (e.g., model averaging or meta-regression). It is intended to preliminarily inform, not replace, a human health assessment. It offers an efficient approach for identifying studies that may be adequate for dose-response analysis and for approximating the relative potency of a chemical with respect to health effects that are known or presumed to be related to increases in exposure. This approach attempts to provide a portion of the information that can be used to prioritize studies and health effects for the more focused and in-depth dose-response analyses that would be performed as part of a full chemical health assessment.

This margin-of-exposure (MOE)-like analysis uses ratios of the study-specific estimates of exposures associated with a 20% increase in relative risk over a background exposure estimate (RRE_20_) divided by the background estimate in the same study-specific exposure units (RRB).^3^ The RRB is, therefore, a unitless, study-specific exposure:exposure ratio (not a risk:risk ratio) that can be used to assess the potential potency of a chemical across health outcomes without the need for extensive adjustments to the exposure metrics and relative risk estimates reported by study authors. The large database of arsenic epidemiological studies is used as a case study to illustrate the approach. Two types of study-specific RRB estimates are derived in this case study, one that uses a background exposure estimate for the U.S. population (RRB-US) in the exposure units of the study (see Table 2) and the other that uses the study-specific background exposure estimated for the study population reference group (RRB-SP).

As defined, the RRB-US ratios are study-specific estimates of exposures associated with a 20% increase in relative risk over an estimated U.S. background exposure level (RRE-US_20_) divided by the estimated U.S. background exposure level. While this provides a means of comparing approximations of potency for different health effects in relation to U.S. background levels, the derivation of the RRE-US_20_ can involve considerable model extrapolation. For this arsenic case study, the estimated U.S. background exposure levels (Table 3) are often well below the background (i.e., the study-specific reference group) exposure levels observed in arsenic studies, which are frequently conducted in regions that have been associated with high arsenic contaminations (e.g., Taiwan, Bangladesh, Chile). Consequently, there is model uncertainty in the RRE-US_20_ and, by extension, the RRB-US estimates from studies of populations with historically high arsenic exposures. A full assessment of all uncertainties associated with every RRB-US derivation presented here is beyond the scope of this screening approach and this paper. However, for comparison purposes, we have also reported RRB-SP values that relate reported relative risk to the study-specific background levels of exposure. RRB-SP estimates may not be as relevant to low exposure populations like the U.S. However, they provide study-specific estimates of potential use for comparing approximations of potency across health effects that will generally involve less extrapolation and, consequently, less model uncertainty and less RRB estimation variability (see further discussion in Section 4).

## 2. Methods

### 2.1. Overview

**Fig. 1.**
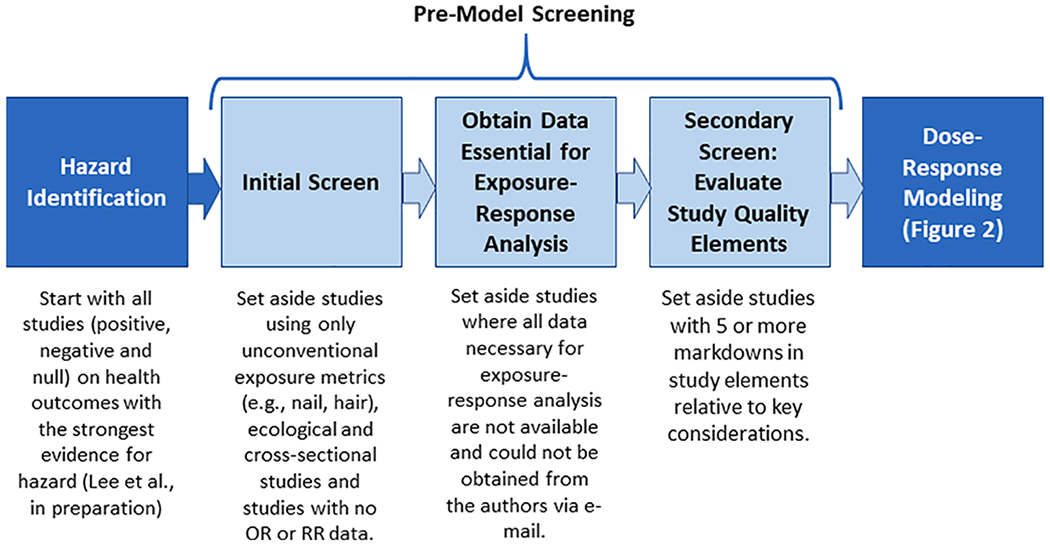
Study Selection.

**Fig. 2.**
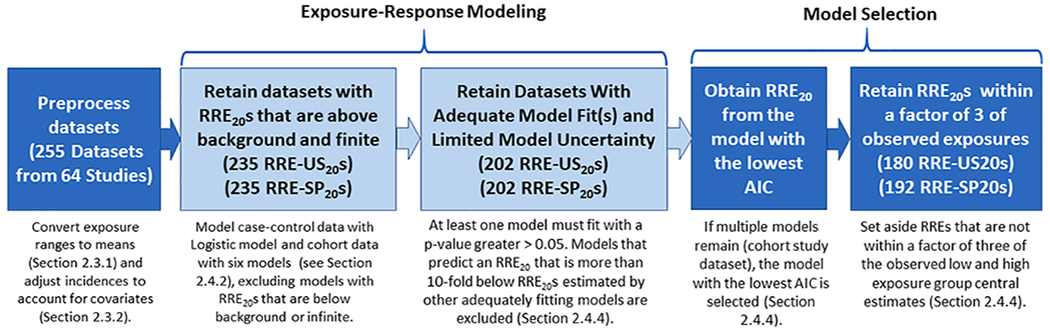
RRE_20_ Derivation.

For the inorganic arsenic test case, studies of 11 health outcomes (Table 1) that have been relatively strongly associated with inorganic arsenic exposure were evaluated.^4^ The RRB analyses derive individual study, preliminary (screening-level) relative risk estimates from analyses of grouped data (e.g., binned by exposure level) from individual epidemiological studies; for the database used in this inorganic arsenic case study, such datasets account for the bulk of exposure-response information. These analyses involve fitting standard parametric exposure-response models (e.g., logistic or Poisson regression) to the exposure or dose metrics reported by the study authors and deriving RRE_20_ estimates based on the results of the best-fitting models. The overall process is detailed in the following sections and illustrated in Fig. 1, “Study Selection,” and Fig. 2, “RRE_20_ Derivation.” Briefly, the methods used in this RRB analysis to screen, prepare, model exposure-response data from studies and derive RRE_20_ and RRB estimates included the following:

Pre-model screening and selection of datasets: a three-step strategy (described in Section 2.2) was used to select studies for modeling.

Data preprocessing: group-level means were estimated, incidence rates were adjusted to account for covariates, background exposures for the U.S. population were estimated, outcomes were mapped to outcome domains, and author-performed trend tests were considered.

Exposure-response modeling: case-control and cohort studies (see Section 2.2.1 for study selection criteria) were modeled^5^ to predict a relative risk exposure (RRE), which is defined as the exposure or dose level where the RR or OR has changed by a certain percentage (the benchmark relative risk, or BMRR) compared to the estimated RR or OR at the estimated U.S. background level of exposure (see Table 3 for estimated U.S. background exposure levels for the different exposure metrics) or at the estimated mean study population reference group level of exposure (see Section 2.3.1). The RRE estimates presented here are for a 20% increase in relative risk over background exposure estimates for the U.S. (RRE-US_20_) and the study population reference group (RRE-SP_20_). The exposure corresponding to an increase in relative risk of 20% is deemed to be sufficient for the purposes of the RRB analyses in this evidence base (see Section 2.5); different RRE benchmarks may be more appropriate for other evidence bases or chemicals.

Exclusion based on lack of fit: Models were excluded from selection if they did not provide adequate fit (p-value < 0.05).

Culling due to model uncertainty: Models with a tendency to predict very steep, sometimes supralinear dose-response curves (i.e., Exponential 4 and Michaelis-Menten models) were excluded if their RRE_20_ result was more than 10-fold below all other adequately fitting models. Further, to avoid including datasets with highly uncertain RRE_20_s, a dataset was excluded if the RRE_20_ was more than a factor of three below the central estimate for the lowest dose group or above the central estimate for the highest dose group of the study. If studies reported an unexposed reference population (i.e., zero exposure), these RRE_20_s were not considered in the RRB analysis.

Derivation of RRB ratios: to facilitate comparisons across exposure metrics, the RRE-US_20_ and RRE-SP_20_ estimates were divided by an estimated U.S. central tendency (background) exposure estimates (Table 3) or the estimated mean study population reference group exposure (Section 2.3.1) to derive the RRB-US and RRB-SP MOE-like estimates^6^.

### 2.2. Study/dataset selection process

This section describes the three-step “Pre-model Screening” process (see Fig. 1) used to select studies of those 11 health outcomes for modeling. The goals of this process were to efficiently identify studies that are potentially adequate for dose-response modeling by applying consistent and transparent preliminary study quality evaluation criteria.

#### 2.2.1. Initial study screen

To begin the study selection process, the datasets for each health outcome were characterized as (1) ecological, (2) cohort/cross-sectional dichotomous, (3) case-control dichotomous, or (4) continuous. For comparison purposes, the screening focuses on evaluating all adequate exposure-response datasets for the exposure level associated with a 20% increase in relative risk of a disease (see Section 2.5). In some cases, study authors were contacted in an attempt to obtain missing data necessary for an exposure-response analysis. The data provided are described in the Supplemental Material for this article. If the necessary data were not provided, the study was not included in the RRB analysis.

Next, studies that provided no quantitative exposure- or dose-response data were set aside. This selection criterion excluded “ecological” studies, where location of residence or geographically averaged arsenic estimates based on a small number of measurements were the sole exposure metrics. Also removed at this step were studies that reported arsenic concentrations in hair, nails, or blood as the sole metrics of exposure due to a relative lack of National background data for the approximation of RRBs from these biomarkers^7^.

In this RRB analysis, the exposure-response metrics were compared across study types. Rothman and Greenland (2005) point out that studies that estimate prevalence (i.e., cross-sectional studies) should not be compared with studies that estimate incidence or mortality rates (i.e., case-control and cohort studies). In addition, the method for adjusting the disease counts for covariates, which differs for case-control, cumulative incidence, and incident rate study metrics, does not have a readily-available counterpart for cross-sectional studies. Therefore, we excluded cross-sectional studies from these RRB analyses.

#### 2.2.2. Secondary screen: Evaluate study elements

Studies were evaluated for suitability for exposure-response modeling. A series of quality rating criteria were used to characterize the potential utility of specific studies for exposure-response (Table 2). The rating criteria were specifically tailored to identify studies that may have been relevant for qualitative information but did not provide suitable information to support exposure-response estimation (see example exposure-response study selection tables for 11 inorganic arsenic health outcomes in Supplemental Material).

For each quality rating criterion, each study was determined to be either “suitable,” “less suitable,” or “not suitable,” with a score of 0, 1, or 2 given to those categorizations, respectively. Additionally, borderline cases between “suitable” and “less suitable” were given a score of 0.5. The scores across all rating criteria were then summed, and datasets that had a score greater than or equal to five were excluded. All rating criteria were weighted equally in this approach (i.e., no individual rating criterion was deemed more important than another). In general, however, the large majority of studies providing quantitative exposure- or dose-response data were retained.

#### 2.2.3. Summary of datasets included in RRB analysis

Table 1 shows the numbers of studies for each health outcome category that were originally identified as potentially useful, the numbers set aside (excluded) in the different study selection steps, and the numbers ultimately included in the RRB analysis. Sixty-eight total studies were included in the RRB analysis: 18 studies evaluating bladder cancer outcomes, 4 studies evaluating diabetes outcomes, 17 studies evaluating diseases of the circulatory system outcomes, 3 studies evaluating liver cancer outcomes, 16 studies evaluating lung cancer outcomes, 2 studies evaluating nonmalignant respiratory outcomes, 3 studies evaluating pregnancy outcomes, 6 studies evaluating renal cancer outcomes, 3 studies evaluating skin cancer outcomes, and 10 studies evaluating skin lesion outcomes. Full details of the study selection rating criteria and decisions are provided in the Supplemental Material for this article.

Within each study selected for analysis, multiple datasets could be present, including analysis of different outcomes (e.g., both stroke and ischemic heart disease for “diseases of the circulatory system”), different populations (e.g., the full study population versus individual susceptible subpopulations), and different exposure metrics (e.g., both water and urinary concentrations). In general, all datasets within each of 68 selected studies were modeled based on the most-adjusted statistical model reported. These criteria led to the inclusion of 255 datasets (Table 1).

### 2.3. Data pre-processing

Once the datasets were identified, the data were pre-processed and the following data attributes were assessed to allow the RRE_20_ and RRB analyses to be performed (see Fig. 2).

#### 2.3.1. Estimating group-level mean exposures

All of the datasets that served as inputs to the modeling associated with this RRB analysis are group-summarized results. An important implication is that exposure metrics are provided at the group level, where the groups are often defined based on exposure bins; for example, an exposure group may be defined as “arsenic concentration less than 3 μg/L”. However, for dose-response modeling, a point estimate representing the group’s exposure or dose is needed. A few studies provided mean or median exposure or dose estimates for each of the exposure groups; for those studies, the provided means or medians were used directly in exposure-response modeling. Where only exposure ranges and numbers of subjects were provided, it was necessary to estimate mean exposures in each group. For the modeling performed in this RRB analysis, the proportions and exposure ranges were fit to lognormal distributions. Distribution fitting was performed by maximum likelihood methods, assuming no truncation of the uppermost exposure range. Group mean estimates were then derived by drawing large Monte Carlo samples (10 million iterations) from the fitted distributions, and sampling randomly within each exposure range for the appropriate numbers of “subjects.” In some cases, referent group exposures were described as “0” with no indication of the exposure range or variance. Such datasets were excluded from consideration for RRB derivation due to lack of a valid referent group background exposure estimate to use for RRB derivation.

#### 2.3.2. Adjusting incidence values to account for covariates

Outcome data were generally supplied only for groups; that is, total numbers of cancers, numbers of subjects/cases and controls/referents. “Crude” and/or covariate-adjusted statistical measures of risk, such as relative risks or hazard ratios (cohort studies) or odds ratios (case-control studies) were also provided. Because the exposure-response models for both case-control and cohort studies specifically are fitted to the numbers of cases and non-cases (or cases and controls), it is necessary to adjust the numbers of expected cases to account for covariate adjustment, similar to the way adjusted ORs account for covariates compared with crude ratios. The approach used for this adjustment involves generating effective-counts (Allen et al., 2020a). The effective-counts for both cases and controls are then used in the model-fitting (see example input data in Tables 5 and 6).

#### 2.3.3. Estimating U.S. population background exposure for RRB-US derivations

An estimate of U.S. background exposure is needed for two purposes in the RRB-US derivation, for the derivation of the RRE-US_20_ (to estimate the baseline RR or OR used in the RRE-US_20_ derivation; see Sections 2.4.2 and 2.4.3) and then also for the derivation of the RRB (RRE-US_20_/U.S background exposure estimate = RRB-US). For estimating the “background” arsenic level for each exposure metric, a U.S. central tendency and U.S. high exposure level was estimated, as shown in Table 3. When needed, we assumed a body weight of 70 kg and intake rates from the 2011 EPA Exposure Factors Handbook (U.S. EPA, 2011) to convert between metrics. The U.S. central tendency values were then used as the baseline.

#### 2.3.4. Mapping specific outcomes to outcome domains

The datasets across the health outcome categories covered many different levels of outcomes. To facilitate comparing RREs across studies within one outcome category, the different outcomes were divided into categories based on what was reported by the authors.

- Outcome Types: clinical – fatal, clinical – non-fatal, preclinical, and subclinical.
  - Outcome domains: subcategories within the health outcome categories used to group similar outcomes.
    - Outcome name in study: the name used for the specific outcome in the study.

Table 4 shows the different outcome types, outcome domains, and outcome names for each of the health outcome categories. The table includes both fatal and non-fatal cancer and non-cancer outcomes. All of the outcomes included in the RRB analysis had at least one dataset where an acceptable model fit could be found.

In some cases, the four outcome types above were not entirely applicable for the health outcome category. For example, pregnancy outcomes that resulted in death to the fetus/baby were characterized as “perinatal mortality” to distinguish it from cancer mortality. Skin lesions, while a preclinical marker for increased skin cancer risk, were given a separate type of “precancer lesions.” The outcome types were used to group results, which are presented in the Supplemental Material for this article.

### 2.4. Exposure-response analysis for case-control and cohort studies

#### 2.4.1. Input data and pre-analysis

Example input datasets are shown in Tables 5 and 6, with data as reported in the epidemiologic study included in the first 4 columns: exposure/dose, number of cases and controls, and adjusted odds ratio for case-control studies, and exposure/dose, number of cases, and adjusted relative risk for cohort studies. As discussed in Section 2.3.1., mean or median exposure/dose metrics for each group was as the authors reported or, alternatively, estimated by fitting distributions to the proportions of subjects in each exposure range. Modeling methods for reported and estimated means were the same. Given that all datasets were modeled individually, no attempt was made to convert to a common exposure metric. Instead, as covered in Section 2.3.3, background estimates for the author-reported exposure or dose metric were used in the benchmark relative risk calculations. In this way, the RRE_20_ values for any dataset using any exposure or dose metric are comparable in terms of potential potency, and thus conversion of all datasets to a common dose metric is not necessary. The cases and controls were adjusted using the effective count method described by Allen et al. (2020a) in a companion article to this article. This adjustment, broadly speaking, ensures the cases and controls are appropriately adjusted to account for any covariates.

#### 2.4.2. Exposure-response modeling

The RRB is a ratio obtained by dividing a study-specific estimate of the exposure associated with a defined health outcome risk by an estimate of the exposure (in the same study-specific units) typically experienced in the U.S. (U.S. background exposure). This section will describe the exposure-response modeling methods used to obtain the numerator of the RRB ratio.

As described in Section 2.3.3, the denominator of the RRB ratio, either the estimate of U.S. background or the study background (reference group) exposure, is also used in the derivation of the numerator of the RRB, the RRE_20_. Specifically, the modeled study data is used to estimate the arsenic exposure level (or dose) corresponding to a RR or OR of 1.2 where the baseline in the modeled data (i.e., a RR or OR of 1.0) corresponds to an estimate of arsenic background exposure (for either the U.S. or study population) in terms of the studied exposure metric. This was done to create RRE_20_, and therefore RRB, estimates that are comparable across studies with widely different referent group exposure levels.

For case-control studies, adjusted case and control numbers are treated as dichotomous data and fitted by the logistic model:

(1) f(dose)=1∕[1+exp(−a−b∗dose)]

where dose is either exposure or dose, depending on the data set. The logistic form was used because, under a logistic regression model, results from case-control studies can be analyzed as if they had been collected prospectively rather than retrospectively. That is, the likelihood contributions from each exposure group in such a study are the same binomial-based likelihoods encountered in other study designs (Prentice and Pyke, 1979). Parameter values in the logistic model were estimated using standard likelihood maximization techniques, similar to the methods incorporated in EPA’s Benchmark Dose Software (U.S. EPA, 2012). Chi-squared p values are reported and used to judge how well the model fit the data. Akaike information criterion (AIC) value are also reported but not used for comparing models because only one model is used for case-control data sets.

For cohort study data, the fundamental assumption made for modeling is that the counts of cases in each exposure group follow a Poisson distribution:

(2) $$o_{i}\;\text{Poisson}[e_{i}\times f(d_{i})]$$

where *o_i_* and *e_i_* are observed cases and expected case number in the ith exposure group, respectively, and *f*(*d_i_*) is an exposure- or dose-response function describing the relationship between exposure or dose and relative risk (which is a continuous measure). Seven continuous dose-dose-response models were used for *f*(·), including the linear model, power model, 2nd-degree polynomial model, Michaelis-Menten model, and the Exponential 2, 3, and 4 models. Models were again fit by maximum likelihood estimation, and chi-squared p-values and AICs were reported.

#### 2.4.3. Predicted odds ratio and relative risks and relative risk dose estimation

For case-control studies, the model also estimates adjusted odds ratios (ORs) associated with each exposure/dose level in the data set based on the logistic model, using the following equation:

(3) $$\text{OR}(d)=\frac{f(d)∕(1-f(d))}{f(d_{0})∕(1-f(d_{0}))}$$

where *f*(·) is the logistic function specified in Eq. (1), d refers to the individual exposure/dose levels, and *f*(*d*_0_) is the percentage of cases predicted at the estimated background arsenic exposure level (see Section 2.3.3). As previously described, the RRE is the study-specific modeled exposure or dose estimate of where OR has changed by a certain benchmark relative risk (BMRR) percentage compared to the modeled OR at the estimated background level of arsenic exposure (RREBMRR or, for our purposes, the RRE_20_):

(4) $$\frac{\text{OR}(\mathrm{RRD})-\text{OR}(d_{0})}{\text{OR}(d_{0})}=\mathrm{BMRR}$$

**Fig. 3.**
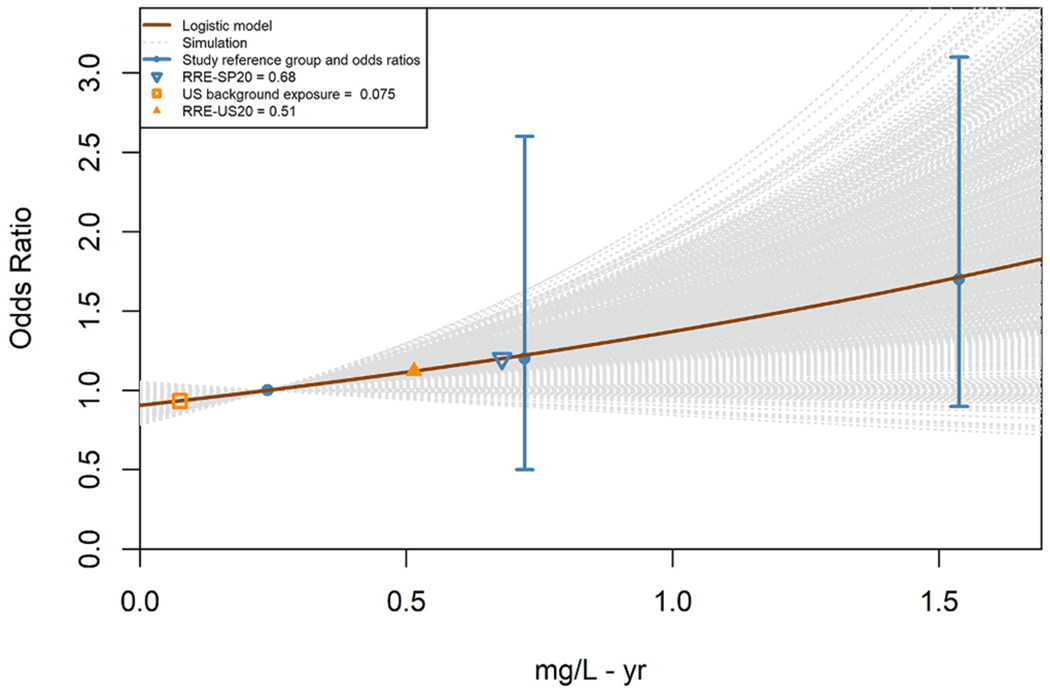
Example plot of RRE-SP_20_ and RRE-US_20_ derivations for a case control drinking water study evaluating carotid atherosclerosis by Hsieh et al. (2008).

The predicted odds ratios and the RRE for each individual study are estimated relative to either a study-specific estimate of the OR at a common arsenic exposure level across studies (the estimated U.S. background exposure levels as described in Section 2.3.3), in the case of the RRE-US_20_ derivations, or the estimated referent exposure level in the individual study, in the case of the RRE-SP_20_ derivations. For example, in the Fig. 3 plot, the RRE-US_20_ and RRE-SP_20_ estimates of 0.51 mg/L-yr and 0.68 mg/L-yr are the model estimates of the cumulative exposure associated with an OR increase of 20% (i.e., an OR of 1.2) over the OR estimated at the U.S. background level (0.075 mg/L-yr; see Table 3) and the study referent exposure level, respectively. By defining the estimated U.S. background arsenic exposure level as the reference point in each individual study (where the odds ratio is 1), in the case of the RRE-US_20_ derivations, differences in levels of exposure in the referent group across studies are not expected to strongly influence the final RRE-US_20_ estimate.

RREs were calculated for benchmark relative risk (BMRR) values representing percent elevations in adjusted OR (results for a BMRR of 20% are shown in the Supplemental Material). Substituting OR (d_0_) = 1, Eq. (3) can be simplified to

(5) OR(RRD)=1+BMRR

where OR(·) is Eq. (3). Thus, the RRE is the exposure level that satisfies Eq. (5).

A bootstrap method was used to derive confidence intervals for the predicted ORs and RRE estimates. One thousand sets of adjusted cases and controls were randomly generated based on binomial distributions of adjusted cases and controls about the maximum likelihood estimates (MLEs).

For cohort studies, the fitted exposure-response functions *f*(·) were used to calculate predicted relative risk at each level evaluated in each data set. In addition, RREs were calculated as the exposure levels that satisfy Eq. (6):

(6) $$\frac{f(\mathrm{RRE})-f(d_{0})}{f(d_{0})}=\mathrm{BMRR}$$

As for case-control data, RREs were calculated for BMRRs that represent increases in relative risk compared to study-specific estimates of risk at estimated background exposure levels (results for the selected BMRR of 20% are shown in the Supplemental Material).

As for the case-control studies, bootstrap simulation was used to derive confidence intervals for the predicted relative risks and RRE estimates. Because the convention is to assume the relative risk is log-normally distributed, the lower and upper confidence bounds on the adjusted relative risk reported by the authors were used to estimate geometric standard deviations of relative risk within each exposure group. One thousand sets of simulated adjusted relative risks were randomly generated from the resulting lognormal distributions. All exposure-exposure-response models considered were fit to these data sets to derive corresponding confidence intervals.

#### 2.4.4. Model fit assessment and model selection

For each dataset, the exposure-response model generated estimates of log-likelihood, AIC value (averaged across the bootstrap iterations) and χ^2^ p-value, estimates of model parameters and 95% upper and lower confidence limits, and predicted risks (ORs for case-control datasets and RRs for cohort datasets) at each exposure level, also with confidence limits (data not shown). During post-processing (see Fig. 2), models with chi-squared p-values less than 0.05 were rejected from consideration.^8^ Also, to address model uncertainty, we excluded results from the Michaelis-Menten and/or Exposure 4 models, both of which can have steep, sometimes supralinear exposure-response curves at low exposure levels, when RRE_20_s for these models were a factor of 10 lower than the other models. If multiple models remain after application of these model exclusion criteria (as can be the case for our analysis of cohort studies), the model with the lowest AIC was selected. Finally, to further address model uncertainty imparted by over-extrapolation, datasets that resulted in RRE_20_ estimates more than a factor of three below the lowest or above the highest study group exposure central estimates were excluded from consideration.

### 2.5. Selection of a benchmark relative risk percentage

For the purposes of this comparative analysis, a 20% BMRR was selected as the primary risk metric. The 20% BMRR was used to estimate the exposure associated with a 20% increase in relative risk (RRE_20_). It is used herein exactly the same way as a BMR is used to derive a BMD (U.S. EPA, 2012). Note that the RRE_20_ represents a maximum likelihood estimate (MLE), and is not a lower confidence limit like a BMDL.

The 20% effect level was chosen after preliminary examination of the effect sizes and exposure ranges of the input data sets evaluated in this case study. A key consideration was minimizing extrapolating far outside the range of the data. Thus, the focus was on deriving RRE values that were in or near the range of the input data. As compared to other tested BMRRs, for a large majority of the datasets, an RRE_20_ estimate was in or near the range of the input data and was therefore considered acceptable for the purposes of this comparative RRB analysis.

## 3. Results

The overall database ultimately used for this RRB test case analyses included 255 datasets, resulting in 180 RRE-US_20_ and 192 RRE-SP_20_ estimates, from 64 studies, and is heterogeneous (e.g., many different countries of origin, study types, exposure metrics, outcomes). A full list of the studies included in this RRB test case analysis and the individual RRE20 and RRB modeling results from ah 255 datasets are provided as supplemental material to this manuscript.

Not every dataset could be fit by the model forms employed. If no model met the model exclusion criteria set forth in Section 2.4.4, the models under consideration did not converge to a satisfactory solution and no outputs (coefficients, predicted risks, RREs) were generated. Also, one dataset (Bates et al., 1995, never smokers) was fit to models that predict a non-positive dose-response that cannot be used for the derivation of RRE_20_ estimates.

Modeling results for the 255 datasets evaluated for RRE_20_ derivation purposes are detailed in Table 1 and illustrated in Fig. 2. They can be categorized as follows:

1. 235 RRE-US_20_ and 235 RRE-SP_20_ estimations remained after removing RRE_20_s that were below background or infinite.
2. 202 RRE-US_20_ and 202 RRE-SP_20_ estimations remained after applying the model fit criteria described in Section 2.4.4.
3. 180 RRE-US_20_ and 192 for RRE-SP_20_ estimations remained after excluding models that resulted in RRE_20_ estimates more than a factor of three below the lowest or above the highest study group exposure central estimates.

**Fig. 4.**
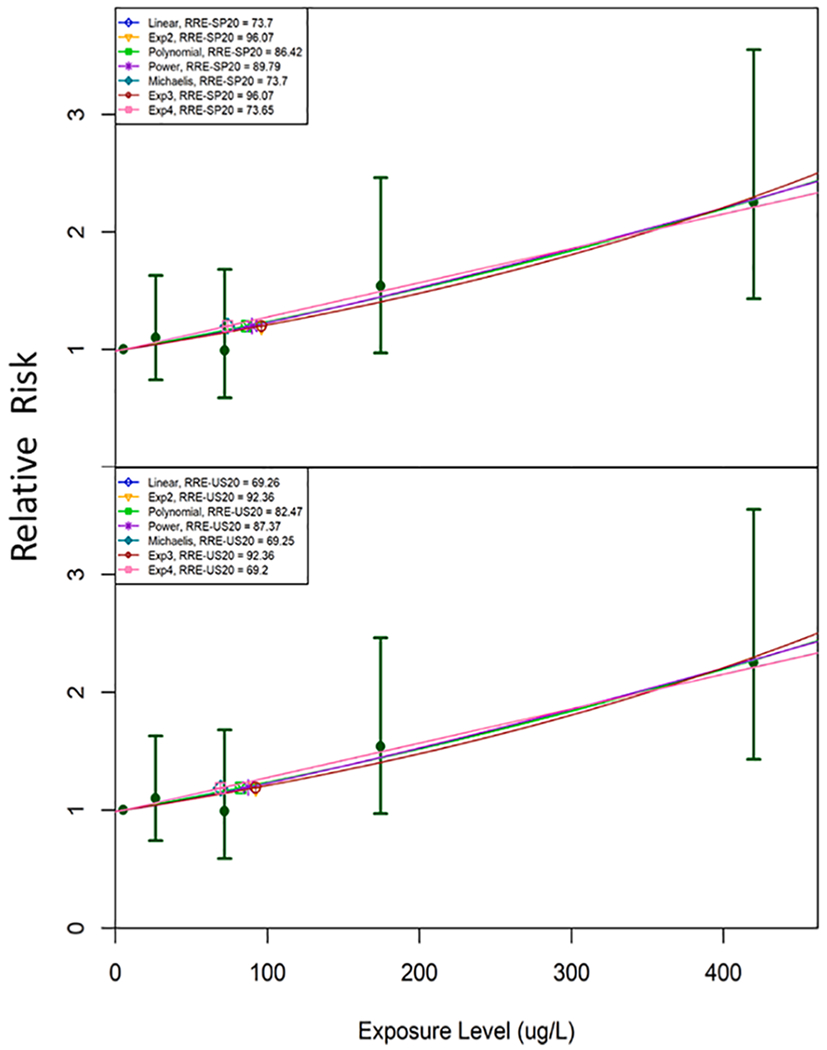
Example plots of RRE-SP_20_ and RRE-US_20_ derivations for cohort study evaluating drinking water exposure and lung cancer by Chen et al. (2010a).

**Fig. 5.**
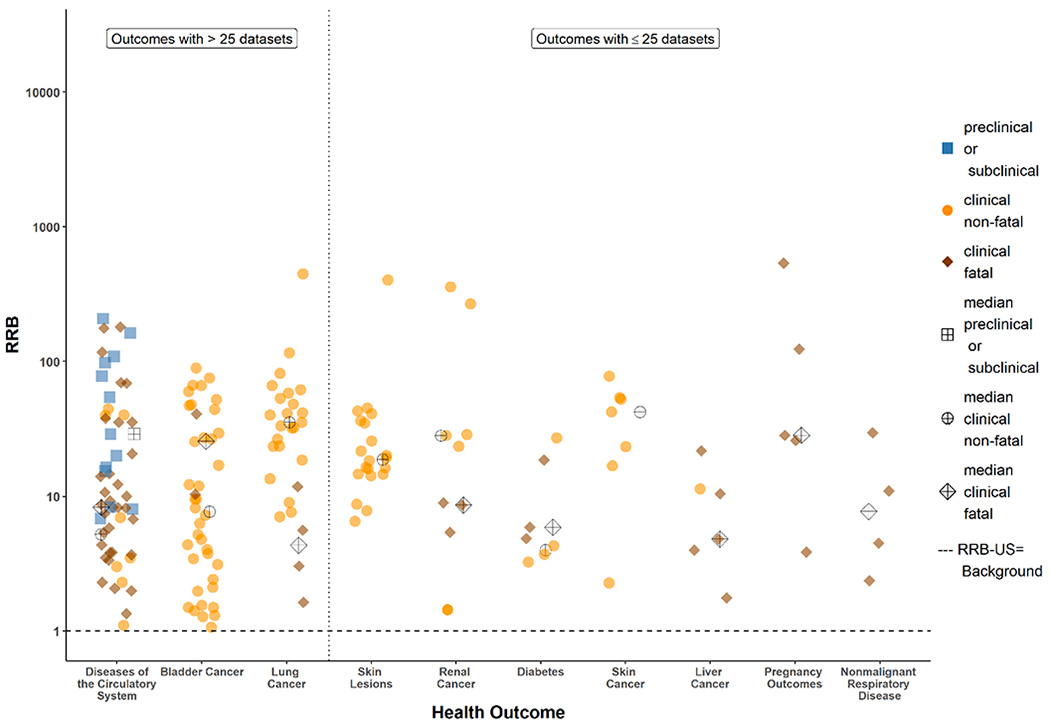
Arsenic Study RRB-US Estimates by Health Outcome and Endpoint Category. Individual and median RRB-US estimates for health outcomes with > 25 and ≤ 25 datasets supporting the derivation of RRE-US_20_ estimates from studies for which the estimated RRE-US_20_ estimate was not more than a factor of three below the central estimate for the lowest dose group or above the central estimate for the highest dose group of the study.

**Fig. 6.**
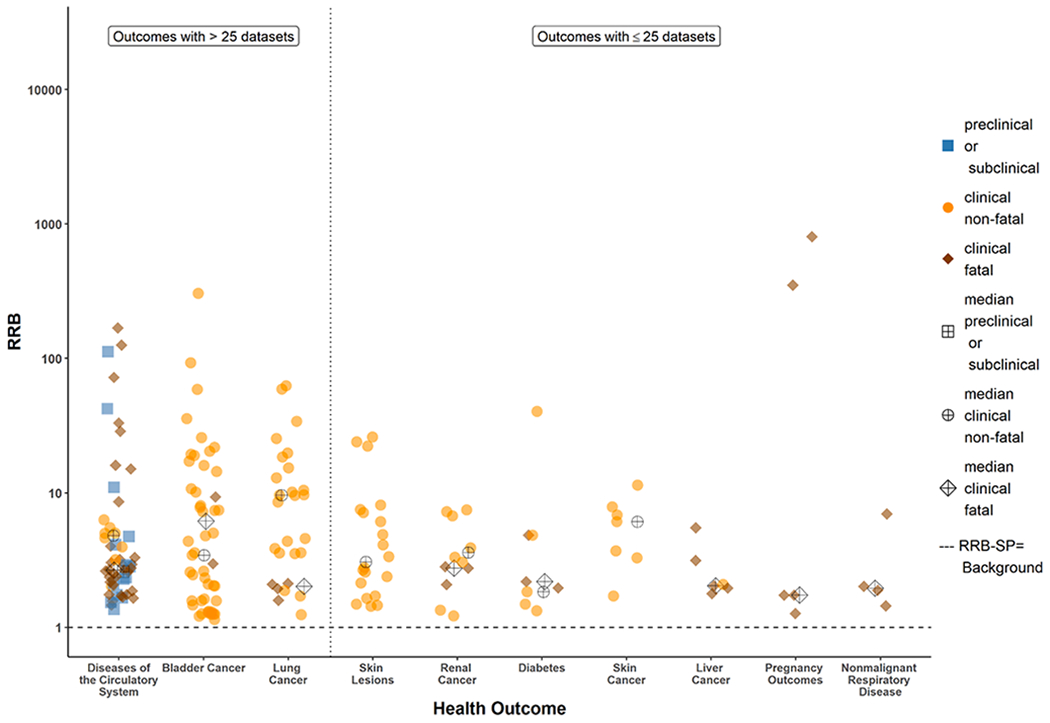
Arsenic Study RRB-SP Estimates by Health Outcome and Endpoint Category Individual and median RRB estimates for health outcomes with >25 and ≤25 datasets supporting the derivation of RRE-SP_20_ estimates from studies for which the estimated RRE-SP_20_ estimate was not more than a factor of three below the central estimate for the lowest dose group or above the central estimate for the highest dose group of the study.

Figs. 3 and 4 illustrate dose-response curves for two datasets: Fig. 3 from a case-control study (Hsieh et al., 2008) and Fig. 4 from a cohort retrospective study (Chen et ah, 2010a), where both studies that produced positive and finite estimates of both RRE-US_20_ and the RRE-SP_20_ from models with acceptable fit, and RRE_20_ estimates were within a factor of three of the range of exposure data reported in each study. Examples of the model uncertainty such as non-positive dose response (Fig. S-47) and uncertainty with the Michaelis-Menton and Exponential models (Fig. S-48) are provided in the Supplemental Material with additional discussion. The counts for datasets included in the RRB-US and RRB-SP analyses are provided in Table 1. Figs. 5 and 6 show individual and median RRB-US (Fig. 5) and RRB-SP (Fig. 6) results for health outcome-specific preclinical/subclinical, clinical nonfatal and clinical fatal health effect categories, organized from left to right by greatest to least number of supporting datasets. Medians are denoted with a cross hatch and shown only where the health outcome had more than 1 dataset with acceptable models fitted. As shown in Table 1 and Fig. 2, datasets with acceptable model fit and RRE_20_ estimates that did not lie far outside the range of data in the underlying study population (i.e., where the RRE_20_ was not more than three times below the lowest exposure group’s mean or three times above the highest exposure group’s) were used in the RRB analysis. Table 7 presents the results shown in Figs. 5 and 6 in tabular form, including the ranges of RRB estimates for each health outcome.

## 4. Discussion

The results of the RRB test case analysis suggest that diseases of the circulatory system, bladder cancer and lung cancer are likely to be important outcomes of concern for evaluating the potential health effects of inorganic arsenic exposure. Diseases of the circulatory system, bladder cancer and lung cancer have the most studies and datasets that meet the criteria for RRB derivation. In particular, they are responsible for 121 (54 diseases of the circulatory system, 40 bladder cancer and 27 lung cancer) of the 180 datasets that met all criteria for RRB-US derivation (67%), and 131 (55 diseases of the circulatory system, 49 bladder cancer and 27 lung cancer) of the 180 datasets that met ah criteria for RRB-SP derivation (68%) (see Table 1 and Supplemental Material). Notably, these outcomes also have a high percentage of RRB-US values below 10 (i.e., RRE-US_20_ values within 10-fold of estimated U.S. central tendency background exposure levels), 50%, 52.5%, and 22%, respectively (see Supplemental Tables S-30A, S-26A, and S-33A). Skin cancer and skin lesions have fewer studies and datasets that meet the criteria for RRB derivation. They are only responsible for 27 (7 skin cancer and 20 skin lesion) of the 180 datasets that met ah criteria for RRB-US derivation (15%), and 27 (8 Skin cancer and 20 skin lesion) of the 192 datasets that met ah criteria for RRB-SP derivation (14%). They also resulted in lower percentages of RRB-US values below 10, 14% and 15%, respectively (see Supplemental Tables S-44A and S-42A).

Ah of the remaining six health outcomes (diabetes, renal cancer, liver cancer, immune effects, pregnancy outcomes and non-malignant respiratory disease) have fewer studies and datasets that meet the criteria for RRB derivation. The number of datasets that meet ah study inclusion, model fit, and RRE_20_ reporting criteria, range from 0 for immune effects to 10 for renal cancer RRB-US derivations (see Table 1 and Supplemental Material Sections 1.2 and 1.4). However, the percentage of RRB-US estimates below 10 was high for several of these health outcomes, including 5 of 7 (71%) for diabetes, 3 of 6 (50%) for liver cancer, 2 of 4 (50%) for nonmalignant respiratory disease, and 5 of 10 (50%) for renal cancer (see Supplemental Tables S-28, S-32, S-36 and S-40). These RRB-US results suggest that, based on this preliminary analysis of limited number of datasets, diabetes, renal cancer, liver cancer and nonmalignant respiratory disease may represent potentially sensitive health outcomes that could warrant more complex, higher tier dose-response analyses.

As stated previously, RRB-SP estimates may not be as relevant to low exposure populations like the U.S. However, they provide study-specific estimates of potential use for comparing approximations of chemical potency across health effects that involve less extrapolation and, consequently, less model uncertainty and less RRB estimation variability. They tend to be associated with higher background levels, resulting in lower RRB estimates. As can be seen from Table 7, RRB-SP estimates are below 10 for all 15 health outcomes for which RRB values were derived (100%), whereas only 8 of 15 RRB-US values are below 10 (53%). Rare exceptions can occur when the study background estimate used in the RRB-SP derivation is lower than our estimate of U.S. background (Table 3). Such is the case for two RRP-SP pregnancy outcome values derived from a study of fetal loss and infant death in relation to arsenic drinking water exposure to Bangladeshi women (Rahman et al., 2007). For these RRP-SP derivations, the study reference group (background) water concentration was 1 μg/L, below our estimated U.S. background of 1.5 μg/L, and the reported RRs did not increase by 20% (i.e., did not reach 1.2), even at the highest, 500 μg/L exposure group. These two RRB-SP estimates were much higher than the other three RRB-SP estimates for pregnancy outcomes, resulting in a high mean, but low RRB-SP value for this health category. In general, however, the RRB-SP values are consistent with the RRB-US results with respect to what they suggest about potential differences in arsenic’s relative potencies across health outcomes, with clinical non-fatal lung and skin cancer studies resulting in the highest and clinical non-fatal diabetes studies resulting in the lowest RRB medians.

When applying this type of approach, investigators should consider whether dataset selection criteria are inordinately biasing results towards or away from the null. For example, in our approach, the exclusion of RRE_20_ results from adequate model fits that are below background, infinite or more than 3-fold above the high observed exposure mean (indicating extremely flat or negative slopes) has the potential to bias away from the null and the exclusion of RRE_20_ results from adequate model fits that are 3-fold below the lowest observed exposure mean but above background (indicating extremely steep slopes) has the potential to bias towards the null. Of the 255 datasets considered in our arsenic case study, 36 RRE-US_20_ (14%) and 21 RRE-SP_20_ (8%) were excluded because they reflected extremely flat or negative slopes and 4 RRE-US_20_ (2%) and 0 RRE-SP_20_ (0%) were excluded because they reflected extremely steep slopes. Thus, while more RRE_20_s indicating flat or negative slopes were excluded, a large majority of the RRE_20_ derivations are consistent with a positive dose-response, and there is little evidence that our exclusion criteria introduced a strong bias away from the null.

It should be recognized that this type of comparative screening approach, RRB analysis, is just one tool that can be used to help prioritize and focus analyses of large databases. Other considerations during the full assessment development process may play a prominent role in guiding the focus of human health assessments, including whether datasets from the same study should be treated independently, whether studies examined potentially susceptible populations or life-stages, whether studies provide adequate information to address response confounders (e.g., smoking), whether the exposure data are sufficient for estimating lifetime daily intake and the importance of the evaluated health outcome(s) for cost-benefit analyses. In addition, the RRB approach described here is not applicable to health outcomes that are generally characterized by continuous response measures (e.g., IQ) and not RR or OR estimates. Further, the RRE_20_ is not meant to represent an exposure associated with a “clinically significant” change in a health outcome or to have any other policy-relevant interpretation other than for purposes such as those described for this RRB analysis, particularly the identification of studies and health outcomes that may warrant further consideration for use in dose-response analysis. Depending on the severity of the health outcome being modeled and the established background risk of disease in the (unexposed) general population (i.e., not estimated in the RRB analysis), the public health significance of a 20% change in relative risk could have very different public health implications. For instance, a 20% increase in the relative risk for a health outcome with a high background lifetime risk might be viewed as having more serious public health implications than would a 20% increase for a health outcome with a low background lifetime risk. In this case, the background lifetime risk for most of the health outcomes under consideration for arsenic are comparable and below 5% with the notable exceptions of the high background lifetime risk in the United States for diabetes, estimated at 32.8% for males and 38.5% for females born in 2000 (Narayan et al. 2003) and for diseases of the circulatory system such as cardiovascular disease, estimated at 66% (2/3) and 50% (1/2) for males and females, respectively, at 40 or 70 years of age (Go et al. 2014).

With respect to diseases of the circulatory system, the smaller RRBs in Figs. 5 and 6 for the more severe (e.g., fatal) endpoints compared to the less severe (e.g., preclinical) endpoints might seem counter-intuitive at first. However, there are at least two possible reasons for this observation. First, pre-clinical measures are just one of many factors associated with the risk of dying from diseases of the circulatory system. Second, the pre-clinical studies are generally in young to middle age adults for which death from diseases of the circulatory system is not imminent. While the risk of an individual pre-clinical factor in a young or middle age adult might be low, the accumulation and progression of multiple factors as that individual ages can result in a relatively high risk of fatality over a lifetime. Thus, what the study-specific data are showing more explicitly is that, in some circumstances, the inorganic arsenic exposure potentially associated with a 20% increase in the relative risk of a young or middle age adult getting one of many pre-clinical markers of diseases of the circulatory system (e.g., cIMT, an indicator of atherosclerosis) can be higher (lower RRB) than the inorganic arsenic exposure potentially associated with a 20% increase in the lifetime relative risk of death from diseases of the circulatory system, a health outcome associated with a lifetime progression of multiple pre-clinical factors.

## 5. Conclusions

The RRB analysis described in this study represents a systematic and pragmatic method for analyzing large databases of exposure- or dose-response data. In applying this type of approach, it is important to achieve a useful balance between study quality, model fit criteria and the number of RRB derivations. The study quality and model fit criteria applied in this arsenic case study resulted in at least six RRB derivations for 73% (8/11) of the health outcomes considered. Depending on the size and quality of a chemical’s database and the purpose of the investigation, it might be reasonable to apply moderate adjustments to selection criteria to achieve an adequate the number of RRB derivations. Limitations of our approach include the inability to address health outcomes typically characterized by response metrics other than ORs and RRs (e.g., continuous response measures such as IQ) and the potential for model uncertainty associated with extrapolating RRE-US_20_ values from studies with substantially higher reference group exposures. This inorganic arsenic example illustrates how an RRB analysis can be helpful for identifying studies and health outcomes that may warrant more detailed and sophisticated analyses. As illustrated in companion case studies, more sophisticated dose-response methods can include model averaging approaches (Mendez et al., 2020) or multistudy Bayesian meta-regression (Allen et al., 2020a, 2020b). Although this RRB analysis used inorganic arsenic data as a test case, it can potentially be implemented for any chemical of concern with a large database of epidemiologic studies in order to prioritize health outcomes and datasets, and guide structured and/or tiered dose-response analyses.

## Supplementary Material

Supplement1

## Figures and Tables

**Table 1:** Literature flow table for selection of studies to be included in the RRB analysis, by health outcome category.

Health outcome category	All HI studies (Starting point)	Studies set aside in initial screen	Studies set aside due to lack of data necessary for exposure-response analysis	Studies set aside in 2nd screen (≥ 5 rating criteria fails)	Studies included in RRB modeling	Datasets included in RRB modeling	RRE-US_20_ Datasets that met model fit criteria (Section 2.4.4)	RRE-US_20_ estimates within a factor of three of the low and high exposure group central estimates	RRE-SP_20_ Datasets that met model fit criteria (Section 2.4.4)	RRE-SP20 estimates within a factor of three of the low and high exposure group central estimates
Bladder cancer	64	37	6	3	19^a^	76	53	40	55	49
Diabetes	49	43	2	0	4^b^	9	8	7	8	8
Diseases of circulatory system	105	75	9	4	14^c^	69	58	54	58	55
Immune effects	20	8	9	3	0	0	0	0	0	0
Liver cancer	30	27	0	0	3^d^	7	6	6	6	6
Lung cancer	87	53	10	8	14^e^	35	28	27	28	27
Nonmalignant respiratory	47	36	6	3	2^f^	5	4	4	4	4
Pregnancy outcomes	39	25	9	2	2^g^	6	6	5	5	5
Renal cancer	32	19	2	5	6^h^	16	12	10	11	11
Skin cancer	38	32	2	1	3^i^	7	7	7	7	7
Skin lesions	72	61	1	0	10^j^	25	20	20	20	20
Total Number of Studies^k^ or Datasets	415	289	47	23	62	255	202	180	202	192

a Baris et al. (2016); D’Ippoliti et al. (2015); Steinmaus et al. (2014a); Chung et al. (2013); Sawada et al. (2013); Steinmaus et al. (2013); Wu et al. (2013); Chung et al. (2011); Chen et al. (2010b); Meliker et al. (2010); Hsu et al. (2008); Huang et al. (2008a, 2008b); Pu et al. (2007); Bates et al. (2004); Chen et al. (2003b); Steinmaus et al. (2003); Bates et al. (1995); Ferreccio et al. (2013).

b Coronado-González et al. (2007); D’Ippoliti et al. (2015); James et al. (2013); Rahman et al. (1998).

c Chen et al. (2011); Chen et al. (2013a); Chen et al. (2013b); D’Ippoliti et al. (2015); Hsieh et al. (2008); James et al. (2015); Moon et al. (2013); Rahman et al. (2014); Sohel et al. (2009); Wade et al. (2009); Wade et al. (2015); Wang et al. (2007); Wu et al. (2006); Wu et al. (2010).

d D’Ippoliti et al. (2015); García-Esquinas et al. (2013); Sawada et al. (2013).

e Argos et al. (2014); Chen and Chen, 2002; Chen et al. (2004); Chen et al. (2010a); Dauphiné et al. (2013); D’Ippoliti et al. (2015); Fan et al. (2009); Ferreccio et al. (2000); García-Esquinas et al. (2013); Mostafa et al. (2008); Qiao et al. (1997); Sawada et al. (2013); Steinmaus et al. (2013); Steinmaus et al. (2014b); Steinmaus et al. (2014a); Grimsrud et al. (2005) was removed because reference group exposure was reported to be 0, resulting in a highly uncertain and infinite RRB.

f Argos et al. (2014); D’Ippoliti et al. (2015).

g Rahman et al. (2007); Rahman et al. (2010); Ihrig et al. (1998) was removed because reference group exposure was reported to be 0, resulting in a highly uncertain and infinite RRB.

h D’Ippoliti et al. (2015); Ferreccio et al. (2013); García-Esquinas et al. (2013); Huang et al. (2012); Mostafa and Cherry, 2013; Sawada et al. (2013).

i Chen et al. (2003a); Gilbert-Diamond et al. (2013); Hsueh (1997), 1018360.

j Argos et al. (2011); Chen et al. (2007); Guo et al. (2006); Hall et al. (2006); Haque et al. (2003); Lindberg et al. (2008); Lindberg et al. (2010); Melkonian et al. (2011); Pierce et al. (2011); Rahman et al. (2006).

k Study totals do not equal sum of columns due to study overlap across health outcome categories.

**Table 2:** Rating criteria for exposure- or dose-dichotomous response datasets.

Rating Element	Criteria
Health outcome	Table 4 describes the health outcomes that were the focus of this arsenic case example. While mortality studies were not excluded, incidence data is preferred
Exposure ascertainment method	Individual measurement or small group averages are preferred over just location of residence/exposure or large group averages
Exposure reporting	Summary statistics such as averages and measures of dispersion/variance preferred over just ranges. Ranges are preferred over absolute values without variance information^a^
Estimates control for smoking, gender, age and other key covariates	Adjusted estimates that include important covariates preferred over unadjusted estimates. Smoking status is a critical covariate that requires adjustment
Number of exposure groups	Studies using more exposure groups preferred, particularly over studies for which the limited number of exposure groups precludes adequate exposure-response modeling
Number of subjects (referents) and cases reported Exposure/dose metric	Explicit reporting of the number of subjects and cases preferred over just statistical summaries (RR, SMRs, etc.) Subject-specific cumulative intake and creatinine corrected urinary intake biomarker^b^ data preferred over cumulative exposure, group exposure or historical exposure measurements
Exposure timing and duration	Explicit ascertainment of exposure histories (timing, duration) preferred over studies that do not ascertain or reported exposure history (e.g., studies that report exposure levels for just one time point)
Representativeness of referent group/controls	Well documented reports that compare referent to exposed groups for key variables preferred over reports that do not provide such documentation or document major differences between referent and exposed groups.
Sufficient number of subjects, cases	A sufficient number of cases to conduct reliable statistical analyses (most applicable to cohort cancer studies) preferred; desirable to have > ~5 cases/exposure group

a Studies that report “0” for control exposures were excluded from consideration for RRB-SP derivations due to lack of a valid referent group background exposure estimate to use for the denominator of the RRB-SP equation.

b An exception is when the subject health outcome studied is associated with renal impairment that could substantially impact clearance rates resulting in higher blood creatinine but lower urinary creatinine in cases relative to controls.

**Table 3:** U.S. central tendency and high arsenic estimates for different exposure and dose metrics.

Exposure metric	Units	U.S. central tendency	U.S. High	Basis for U.S. estimate
Drinking water concentration	μg/L	1.5	15.4	median, 95th percentile county mean As in drinking water (USGS, 2011)
Cumulative exposure from drinking water	μg - yr/L	75	770	1.5 μg/L or 15.4 μg/L (above) × 50 yrs
Daily intake	μg/day (water)	1.5	15.4	1.5 μg/L or 15.4 μg/L (above) × 1.0 L/day (U.S. EPA, 2011)
Dietary intake	μg/day (food)	3.5	13.3	0.05 μg/kg-d mean or 0.19 μg/kg-d 95th percentile adult intake (Xue et al. 2010) × 70-kg adult
	μg/day (food + water)	5	28.7	Sum of food and water
Cumulative intake	mg (cumulative intake, water)	27.4	281	1.5 μg/day or 15.4 μg/day (above) × 50 yrs
	mg (cumulative intake, food + water)	91.3	524	5 μg/day or 28.7 μg/day (above) × 50 yrs
Urine concentration (cr. Adj.)	μg As excretion/g creatinine	7.4	18.4	NHANES (2013–2014) median or 95th percentile for total arsenic (CDC, 2016)
Urine concentration	μg AS excretion/L urine	5	16.8	NHANES (2013–2014) median or 95th percentile for total arsenic (CDC, 2016)
Air	μg/m^3^	0.00075	0.00156	https://cfpub.epa.gov/roe/indicator.cfm?i=90#8; EPA’s ambient monitoring archive, arsenic data averaged between 2010 and 2013
Cumulative air	μg/m^3^-years	0.0375	0.078	0.00075 μg/m^3^ or 0.00156 μg/m^3^ (above) × 50 yrs

**Table 4:** Outcome types, domains, and specific outcome names considered in the RRB analysis.

Health outcome category	Outcome type	Outcome domain	Outcome name from study
Bladder cancer	Clinical, fatal	Bladder cancer	Bladder cancer mortality
	Clinical, non-fatal	Bladder cancer	All urinary cancer
			Bladder cancer
			Urinary transitional cell carcinoma
			Urothelial carcinoma
Diabetes	Clinical, fatal	Diabetes	Diabetes mortality
	Clinical, non-fatal	Diabetes	Diabetes
			Type 2 diabetes
Diseases of the circulatory system	Clinical, fatal	Cerebrovascular	Cerebrovascular disease mortality
			Stroke mortality
		Coronary Heart Disease (CHD)	CHD & other heart disease mortality
			CHD mortality
			Coronary atherosclerosis mortality
			Myocardial infarction mortality
		Cardiovascular Disease (CVD)	CVD mortality
		Peripheral Artery Disease (PAD)	PAD mortality
	Clinical, non-fatal	Cerebrovascular	Cerebrovascular disease
			Stroke
		CHD	CHD
		CVD	CVD
		Hypertension	Hypertension
	Preclinical	Hypertension	Corrected Q wave-T wave interval (QTc) prolongation
	Subclinical	Atherosclerosis	Carotid atherosclerosis
Liver cancer	Clinical, fatal	Liver cancer	Liver and bile duct cancer mortality
			Liver, gallbladder, and bile duct cancer mortality
	Clinical, non-fatal	Liver cancer	Liver cancer
Lung cancer	Clinical, fatal	Lung cancer	Lung cancer mortality
	Clinical, non-fatal	Lung cancer	Lung adenocarcinoma
			Lung cancer
			Other lung cancer histopath. Types Squamous cell carcinoma
Nonmalignant respiratory disease	Clinical, fatal	Chronic Obstructive Pulmonary Disease (COPD)	COPD mortality
Pregnancy outcomes	Perinatal mortality	Fetal loss	Spontaneous abortion
		Infant mortality	Infant mortality
		Stillbirths	Stillbirths
Renal cancer	Clinical, fatal	Kidney cancer	Kidney cancer mortality
	Clinical, non-fatal	Kidney cancer	All kidney cancers
			Kidney cancer
			Renal and transitional cell cancer
			Renal cell cancer
			Renal cell carcinoma
			Transitional cell cancer
Skin cancer	Clinical, non-fatal	Skin cancer	Skin cancer
Skin lesions	Precancer lesions	Skin lesions	Keratosis
			Pigment disorder
			Skin lesions

**Table 5:** Example input data from case-control studies.

Drinking water arsenic intake, μg/d	Cases/Controls	Adjusted OR (95% CI)	Effective Cases	Effective Controls
0.399	189/252	–	189.00	210.37
3.31	162/234	0.83 (0.62–1.11)	145.13	194.62
28.7	43/48	1.01 (0.62–1.64)	37.01	40.79

**Table 6:** Example input data from cohort studies.

Cumulative water exposure, μg/L-years	Cases	Adjusted RR (95% CI)	Effective Cases	Effective Expected Number
157	6	–	6.00	6.00
657	3	1.11 (0.27–4.54)	2.84	2.56
2343	12	2.33 (0.86–6.36)	10.65	4.57
7048	5	3.77 (1.13–12.6)	4.72	1.25
37,873	11	7.49 (2.70–20.8)	9.56	1.28

**Table 7:** Ranges, mean, and median RRB-US and RRB-SP values from modeled datasets for each health outcome category with more than one dataset.

Health outcome	RRB	Preclinical or Subclinical			Clinical Non-Fatal			Clinical Fatal		
		
		Range	Mean	Median	Range	Mean	Median	Range	Mean	Median
Bladder Cancer	RRB-US	N/A	N/A	N/A	1.07–89.2	20.6	7.72	10.4–40.8	25.6	N/A
	RRB-SP	N/A	N/A	N/A	1.14–304	16.2	3.45	2.97–9.34	6.16	N/A
Diabetes	RRB-US	N/A	N/A	N/A	3.25–27.1	9.58	3.99	4.87–18.6	9.79	5.90
	RRB-SP	N/A	N/A	N/A	1.33–40.4	9.98	1.83	1.96–4.48	3.00	2.19
Diseases of the circulatory system	RRB-US	6.86–209	62.8	29.0	1.10–44.6	27.4	5.21	1.35–181	27.4	8.29
	RRB-SP	1.37–112	13.8	2.59	2.02–6.30	4.45	4.80	1.46–169	16.0	2.67
Liver Cancer	RRB-US	N/A	N/A	N/A	N/A	N/A	N/A	1.76–21.8	8.57	4.83
	RRB-SP	N/A	N/A	N/A	N/A	N/A	N/A	1.78–5.49	2.88	2.04
Lung Cancer	RRB-US	N/A	N/A	N/A	7.06–448	57.3	35.5	1.64–11.8	5.52	4.33
	RRB-SP	N/A	N/A	N/A	1.24–62.5	14.5	9.61	1.59–2.12	1.94	2.02
Nonmalignant Respiratory Disease	RRB-US	N/A	N/A	N/A	N/A	N/A	N/A	2.37–29.7	11.9	7.73
	RRB-SP	N/A	N/A	N/A	N/A	N/A	N/A	1.45–6.97	3.08	1.95
Pregnancy Outcomes	RRB-US	N/A	N/A	N/A	N/A	N/A	N/A	3.86–537	144	28.4
	RRB-SP	N/A	N/A	N/A	N/A	N/A	N/A	1.27–805	232	1.74
Renal Cancer	RRB-US	N/A	N/A	N/A	1.43–358	101	28.2	5.41–8.97	7.67	8.62
	RRB-SP	N/A	N/A	N/A	1.22–7.45	4.28	3.60	2.08–2.83	2.56	2.76
Skin Cancer	RRB-US	N/A	N/A	N/A	2.27–77.7	38.4	42.2	N/A	N/A	N/A
	RRB-SP	N/A	N/A	N/A	1.71–11.4	5.84	6.12	N/A	N/A	N/A
Skin Lesions	RRB-US	N/A	N/A	N/A	6.52–402	41.1	18.8	N/A	N/A	N/A
	RRB-SP	N/A	N/A	N/A	1.43–26.0	6.68	3.07	N/A	N/A	N/A

## Footnotes

2 As suggested by Zartarian et al. (2005), we will generally use the term “exposure” to represent contact between an agent and a target at an exposure surface, and the term “dose” to represent the amount of agent that crosses an exposure surface whether the surface is an absorption barrier (e.g., the lining of the stomach or lungs) or not (e.g., a conceptual surface over the nose and open mouth).

3 As defined in the EPA IRIS glossary, a margin of exposure (MOE) is a “point of departure” (i.e., an exposure level associated with a specific level of risk) divided by the actual or projected environmental exposure of interest. Like the MOE, the lower an RRB, the greater the concern. However, whereas MOE approaches are typically based on the results of an explicit exposure assessment and PODs (e.g., NOAELs or BMDLs) that are developed to be representative of the potential for effects across a broad population, the RRB analyses reflect a hypothetical exposure scenario based on individual study exposure estimates and results constrained to the studied population without further efforts that might inform their utility for extrapolation.

4 This is not meant to represent all health outcomes potentially associated with inorganic arsenic exposure. The RRB approach described here is not applicable to health effects such as developmental neurotoxicity that are generally characterized by continuous response measures (e.g., IQ) and not RR or OR estimates.

5 All model runs for each dataset were first evaluated per EPA benchmark dose (BMD) guidelines U.S. EPA. Benchmark dose technical guidance. Washington, DC: U.S. Environmental Protection Agency, Risk Assessment Forum; 2012 to choose a best-fitting model.

6 This results in a unitless, study-specific ratio that can be compared across studies regardless of the exposure metric reported in the study.

7 While extrapolations from these metrics were not pursued for this the RRB case study, they could be useful for the purposes of a more in-depth dose-response analysis, particularly when a reasonable method of associating the metric with water concentrations is provided or available.

8 This includes models that are overparameterized (i.e., contain as many or more parameters than the number of dose groups) and for which a p-value cannot be estimated due to having 0 degrees of freedom.
