# Supplementary material for: Use of study-specific MOE-like estimates to prioritize health effects from chemical exposure for analysis in human health assessments
Source: Environ Int. Author manuscript; Available in PMC 2021 Nov 1. (PMC7572727; doi:10.1016/j.envint.2020.105986)
Supplement: Supplement1 [file NIHMS1627772-supplement-Supplement1.docx]

**Supplemental Material – Inorganic Arsenic Study-Specific Margin of Exposure-Type Analysis**

**CONTENTS**

[**Supplemental Material – Inorganic Arsenic Study-Specific Margin of Exposure-Type Analysis** 1](#_Toc39263860)

[1. Study Selection Summary Tables 8](#_Toc39263861)

[1.1. Overview 8](#_Toc39263862)

[1.2. Study Selection Results 9](#_Toc39263863)

[1.2.1. Bladder Cancer 9](#_Toc39263865)

[1.2.2. Diseases of the Circulator System 13](#_Toc39263866)

[1.2.3. Lung Cancer 23](#_Toc39263867)

[1.2.4. Skin Cancer 30](#_Toc39263868)

[1.2.5. Skin Lesions 33](#_Toc39263869)

[1.2.6. Diabetes 40](#_Toc39263872)

[1.2.7. Immune System Impairment 45](#_Toc39263873)

[1.2.8. Liver Cancer 48](#_Toc39263874)

[1.2.9. Nonmalignant Respiratory Disease 51](#_Toc39263875)

[1.2.10. Pregnancy Outcomes 60](#_Toc39263876)

[1.2.11. Renal Cancer 67](#_Toc39263877)

[1.3. Author-Provided Data 70](#_Toc39263878)

[1.4. Additional Graphs and Tables 82](#_Toc39263879)

[1.4.1. Bladder Cancer Exposure-Response Modeling Results 82](#_Toc39263880)

[1.4.2. Diabetes Exposure-Response Modeling Results 99](#_Toc39263881)

[1.4.3. Disease of the Circulatory System Exposure-Response Modeling Results 108](#_Toc39263882)

[1.4.4. Liver Cancer Exposure-Response Modeling Results 123](#_Toc39263883)

[1.4.5. Lung Cancer Exposure-Response Modeling Results 133](#_Toc39263885)

[1.4.6. Nonmalignant Respiratory Disease Exposure-Response Modeling Results 150](#_Toc39263886)

[1.4.7. Pregnancy Outcomes Exposure-Response Modeling Results 158](#_Toc39263888)

[1.4.8. Renal Cancer Exposure-Response Modeling Results 166](#_Toc39263889)

[1.4.9. Skin Cancer Exposure-Response Modeling Results 176](#_Toc39263891)

[1.4.10. Skin Lesions Exposure-Response Modeling Results 182](#_Toc39263892)

[1.5. Examples of Exposure-Response Model Uncertainty 195](#_Toc39263893)

[1.5.1. Non-Positive Exposure-Response Models 195](#_Toc39263894)

[Uncertainty in Michaelis-Menton and Exponential 4 196](#_Toc39263895)

[1.5.2. Exposure-Response Models 196](#_Toc39263896)

**TABLES**

[Table S-1. Bladder cancer exposure-response study selection 9](#_Toc39263902)

[Table S-2. Diseases of the circulatory system exposure-response study selection 13](#_Toc39263903)

[Table S-3. Lung cancer exposure-response study selection 23](#_Toc39263904)

[Table S-4. Skin cancer exposure-response study selection 30](#_Toc39263905)

[Table S-5. Skin lesions exposure-response study selection 33](#_Toc39263906)

[Table S-6. Diabetes exposure-response study selection 40](#_Toc39263907)

[Table S-7. Immune system impairment exposure-response study selection 45](#_Toc39263908)

[Table S-8. Liver cancer exposure-response study selection 48](#_Toc39263909)

[Table S-9. Nonmalignant respiratory disease exposure-response study selection 51](#_Toc39263910)

[Table S-10. Pregnancy outcomes exposure-response study selection 60](#_Toc39263911)

[Table S-11. Renal cancer exposure-response study selection 67](#_Toc39263912)

[Table S-12. Data provided for Argos et al. (2007) 70](#_Toc39263913)

[Table S-13. Data provided for Aschengrau et al. (1989) 71](#_Toc39263914)

[Table S-14. Data provided for Chen et al. (2011b) 72](#_Toc39263915)

[Table S-15. Data provided for D'Ippoliti et al. (2015) 72](#_Toc39263916)

[Table S-16. Data provided for Gilbert-Diamond et al. (2013) 73](#_Toc39263917)

[Table S-17. Data provided for James et al. (2015) 73](#_Toc39263918)

[Table S-18. Data provided for Moon et al. (2013) 74](#_Toc39263919)

[Table S-19. Data provided for Sohel et al. (2009) 76](#_Toc39263920)

[Table S-20. Data provided for Tseng et al. (2003) 76](#_Toc39263921)

[Table S-21. Data provided for Wade et al. (2009) 77](#_Toc39263922)

[Table S-22. Data provided for Wade et al. (2015) 77](#_Toc39263923)

[Table S-23. Data provided for Wasserman et al. (2004) 77](#_Toc39263924)

[Table S-24. Data provided for Wu et al. (2012b) 78](#_Toc39263925)

[Table S-25. Summary of datasets considered in bladder cancer exposure response RRB analysis by exposure metric 82](#_Toc39263926)

[Table S-26A. Summary of RRE-US_20_ and RRB-US for bladder cancer studies 97](#_Toc39263927)

[Table S-26B. Summary of RRE-SP_20_ and RRB-SP for bladder cancer studies 98](#_Toc39263928)

[Table S-27. Summary of datasets considered in diabetes exposure-response RRB analysis by exposure metric 99](#_Toc39263929)

[Table S-28A. Summary of RRE-US_20_s and RRB-US for diabetes studies 107](#_Toc39263930)

[Table S-28B. Summary of RRE-SP_20_s and RRB-SP for diabetes studies 107](#_Toc39263931)

[Table S-29. Summary of datasets considered in diseases of the circulatory system exposure-response RRB by exposure metric 108](#_Toc39263932)

[Table S-30A. Summary of RRE-US_20_s and RRB-US for diseases of the circulatory system studies 121](#_Toc39263933)

[Table S-30B. Summary of RRE-SP_20_s and RRB-SP for diseases of the circulatory system studies 122](#_Toc39263934)

[Table S-31. Summary of datasets considered in liver cancer exposure-response RRB analysis by exposure metric 123](#_Toc39263935)

[Table S-32A. Summary of RRE-US_20_s and RRB-US for liver cancer studies 132](#_Toc39263936)

[Table S-32B. Summary of RRE-SP_20_s and RRB-SP for liver cancer studies 132](#_Toc39263937)

[Table S-33. Summary of datasets considered in lung cancer exposure-response RRB analysis by exposure metric 133](#_Toc39263938)

[Table S-34A. Summary of RRE-US_20_s and RRB-US for lung cancer studies 148](#_Toc39263939)

[Table S-34B. Summary of RRE-SP_20_s and RRE-SP for lung cancer studies 149](#_Toc39263940)

[Table S-35. Summary of datasets considered in nonmalignant respiratory disease exposure-response RRB analysis by exposure metric 150](#_Toc39263941)

[Table S-36A. Summary of RRE-US_20_s and RRB-US for nonmalignant respiratory disease studies 157](#_Toc39263942)

[Table S-36B. Summary of RRE-SP_20_s and RRB-SP for nonmalignant respiratory disease studies 157](#_Toc39263943)

[Table S-37. Summary of datasets considered in pregnancy outcomes exposure-response RRB analysis by exposure metric 158](#_Toc39263944)

[Table S-38A. Summary of RRE-US_20_s and RRB-US for pregnancy outcomes studies 165](#_Toc39263945)

[Table S-38B. Summary of RRE-SP_20_s and RRB-SP for pregnancy outcomes studies 165](#_Toc39263946)

[Table S-39. Summary of datasets considered in renal cancer exposure-response RRB analysis by exposure metric 166](#_Toc39263947)

[Table S-40A. Summary of RRE-US_20_s and RRB-US for renal cancer studies 175](#_Toc39263948)

[Table S-40B. Summary of RRE-SP_20_s and RRB-SP for renal cancer studies 175](#_Toc39263949)

[Table S-41. Summary of datasets considered in skin cancer exposure-response RRB analysis by exposure metric 176](#_Toc39263950)

[Table S-42A. Summary of RRE-US_20_s and RRB-US for skin cancer studies 181](#_Toc39263951)

[Table S-42B. Summary of RRE-SP_20_s and RRB-SP for skin cancer studies 181](#_Toc39263952)

[Table S-43. Summary of datasets considered in skin lesions exposure-response RRB analysis by exposure metric 183](#_Toc39263953)

[Table S-44A. Summary of RRE-US_20_s and RRB-US for skin lesions studies 194](#_Toc39263954)

[Table S-44B. Summary of RRE-SP_20_s and RRB-SP for skin lesions studies 194](#_Toc39263955)

FIGURES

[Figure S-1A. Exposure levels and RRE-US20 for bladder cancer using cumulative exposure. 83](#_Toc39263956)

[Figure S-1B. Exposure levels and RRE-SP20 for bladder cancer using cumulative exposure. 84](#_Toc39263957)

[Figure S-2A. Exposure levels and RRE-US20 for bladder cancer using cumulative intake. 85](#_Toc39263958)

[Figure S-2B. Exposure levels and RRE-SP20 for bladder cancer using cumulative intake. 86](#_Toc39263959)

[Figure S-3A. Exposure levels and RRE-US20 for bladder cancer using daily intake. 87](#_Toc39263960)

[Figure S-3B. Exposure levels and RRE-SP20 for bladder cancer using daily intake. 88](#_Toc39263961)

[Figure S-4A. Exposure levels and RRE-US20 for bladder cancer using dietary intake. 89](#_Toc39263962)

[Figure S-4B. Exposure levels and RRE-SP20 for bladder cancer using dietary intake. 90](#_Toc39263963)

[Figure S-5A. Exposure levels and RRE-US20 for bladder cancer using creatinine adjusted urine concentration. 91](#_Toc39263964)

[Figure S-5B. Exposure levels and RRE-SP20 for bladder cancer using creatinine adjusted urine concentration. 92](#_Toc39263965)

[Figure S-6A. Exposure levels and RRE-US20 for bladder cancer using urine concentration. 93](#_Toc39263966)

[Figure S-6B. Exposure levels and RRE-SP20 for bladder cancer using urine concentration. 94](#_Toc39263967)

[Figure S-7A. Exposure levels and RRE-US20 for bladder cancer using water concentration. 95](#_Toc39263968)

[Figure S-7B. Exposure levels and RRE-SP20 for bladder cancer using water concentration. 96](#_Toc39263969)

[Figure S-8. Exposure levels and RRE-SP20 for diabetes using cumulative exposure. 100](#_Toc39263970)

[Figure S-9A. Exposure levels and RRE-US20 for diabetes using cumulative intake. 101](#_Toc39263971)

[Figure S-9B. Exposure levels and RRE-SP20 for diabetes using cumulative intake. 102](#_Toc39263972)

[Figure S-10A. Exposure levels and RRE-US20 for diabetes using creatinine adjusted urine concentration. 103](#_Toc39263973)

[Figure S-10B. Exposure levels and RRE-SP20 for diabetes using creatinine adjusted urine concentration. 104](#_Toc39263974)

[Figure S-11A. Exposure levels and RRE-US20 for diabetes using water concentration. 105](#_Toc39263975)

[Figure S-11B. Exposure levels and RRE-SP20 for diabetes using water concentration. 106](#_Toc39263976)

[Figure S-12A. Exposure levels and RRE-US20 for diseases of the circulatory system using cumulative exposure. 109](#_Toc39263977)

[Figure S-12B. Exposure levels and RRE-SP20 for diseases of the circulatory system using cumulative exposure. 110](#_Toc39263978)

[Figure S-13A. Exposure levels and RRE-US20 for diseases of the circulatory system using cumulative intake. 111](#_Toc39263979)

[Figure S-13B. Exposure levels and RRE-SP20 for diseases of the circulatory system using cumulative intake. 112](#_Toc39263980)

[Figure S-14A. Exposure levels and RRE-US20 for diseases of the circulatory system using creatinine adjusted urine concentration. 113](#_Toc39263981)

[Figure S-14B. Exposure levels and RRE-SP20 for diseases of the circulatory system using creatinine adjusted urine concentration. 114](#_Toc39263982)

[Figure S-15A. Exposure levels and RRE-US20 for diseases of the circulatory system using water concentration Part 1 of 3. 115](#_Toc39263983)

[Figure S-15B. Exposure levels and RRE-SP20 for diseases of the circulatory system using water concentration Part 1 of 3. 116](#_Toc39263984)

[Figure S-16A. Exposure levels and RRE-US20 for diseases of the circulatory system using water concentration Part 2 of 3. 117](#_Toc39263985)

[Figure S-16B. Exposure levels and RRE-SP20 for diseases of the circulatory system using water concentration Part 2 of 3. 118](#_Toc39263986)

[Figure S-17A. Exposure levels and RRE-US20 for diseases of the circulatory system using water concentration Part 3 of 3. 119](#_Toc39263987)

[Figure S-17B. Exposure levels and RRE-SP20 for diseases of the circulatory system using water concentration Part 3 of 3. 120](#_Toc39263988)

[Figure S-18A. Exposure levels and RRE-US20 for liver cancer using cumulative intake. 124](#_Toc39263989)

[Figure S-18A. Exposure levels and RRE-SP20 for liver cancer using cumulative intake. 125](#_Toc39263990)

[Figure S-19A. Exposure levels and RRE-US20 for liver cancer using dietary intake. 126](#_Toc39263991)

[Figure S-19B. Exposure levels and RRE-SP20 for liver cancer using dietary intake. 127](#_Toc39263992)

[Figure S-20A. Exposure levels and RRE-US20 for liver cancer using creatinine adjusted urine concentration. 128](#_Toc39263993)

[Figure S-20B. Exposure levels and RRE-SP20 for liver cancer using creatinine adjusted urine concentration. 129](#_Toc39263994)

[Figure S-21A. Exposure levels and RRE-US20 for liver cancer using water concentration. 130](#_Toc39263995)

[Figure S-21B. Exposure levels and RRE-SP20 for liver cancer using water concentration. 131](#_Toc39263996)

[Figure S-22A. Exposure levels and RRE-US20 for lung cancer using cumulative air exposure. 134](#_Toc39263997)

[Figure S-22B. Exposure levels and RRE-SP20 for lung cancer using cumulative air exposure. 135](#_Toc39263998)

[Figure S-23A. Exposure levels and RRE-US20 for lung cancer using cumulative exposure. 136](#_Toc39263999)

[Figure S-23B. Exposure levels and RRE-SP20 for lung cancer using cumulative exposure. 137](#_Toc39264000)

[Figure S-24A. Exposure levels and RRE-US20 for lung cancer using cumulative intake. 138](#_Toc39264001)

[Figure S-24B. Exposure levels and RRE-SP20 for lung cancer using cumulative intake. 139](#_Toc39264002)

[Figure S-25A. Exposure levels and RRE-US20 for lung cancer using daily intake. 140](#_Toc39264003)

[Figure S-25B. Exposure levels and RRE-SP20 for lung cancer using daily intake. 141](#_Toc39264004)

[Figure S-26A. Exposure levels and RRE-US20 for lung cancer using dietary intake. 142](#_Toc39264005)

[Figure S-26B. Exposure levels and RRE-SP20 for lung cancer using dietary intake. 143](#_Toc39264006)

[Figure S-27A. Exposure levels and RRE-US20 for lung cancer using creatinine adjusted urine concentration. 144](#_Toc39264007)

[Figure S-27B. Exposure levels and RRE-SP20 for lung cancer using creatinine adjusted urine concentration. 145](#_Toc39264008)

[Figure S-28A. Exposure levels and RRE-US20 for lung cancer using water concentration. 146](#_Toc39264009)

[Figure S-28B. Exposure levels and RRE-SP20 for lung cancer using water concentration. 147](#_Toc39264010)

[Figure S-29A. Exposure levels and RRE-US20 for nonmalignant respiratory disease using cumulative intake. 151](#_Toc39264011)

[Figure S-29B. Exposure levels and RRE-SP20 for nonmalignant respiratory disease using cumulative intake. 152](#_Toc39264012)

[Figure S-30A. Exposure levels and RRE-US20 for nonmalignant respiratory disease using water concentration. 153](#_Toc39264013)

[Figure S-30B. Exposure levels and RRE-SP20 for nonmalignant respiratory disease using water concentration. 154](#_Toc39264014)

[Figure S-31A. Exposure levels and RRE-US20 for nonmalignant respiratory disease using creatinine adjusted urine concentrations. 155](#_Toc39264015)

[Figure S-31B. Exposure levels and RRE-SP20 for nonmalignant respiratory disease using creatinine adjusted urine concentrations. 156](#_Toc39264016)

[Figure S-32A. Exposure levels and RRE-US20 for pregnancy outcomes using air concentration. 159](#_Toc39264017)

[Figure S-32B. Exposure levels and RRE-SP20 for pregnancy outcomes using air concentration. 160](#_Toc39264018)

[Figure S-33A. Exposure levels and RRE-US20 for pregnancy outcomes using urine concentration. 161](#_Toc39264019)

[Figure S-33B. Exposure levels and RRE-SP20 for pregnancy outcomes using urine concentration. 162](#_Toc39264020)

[Figure S-34A. Exposure levels and RRE-US20 for pregnancy outcomes using water concentration. 163](#_Toc39264021)

[Figure S-34B. Exposure levels and RRE-SP20 for pregnancy outcomes using water concentration. 164](#_Toc39264022)

[Figure S-35A. Exposure levels and RRE-US20 for renal cancer using cumulative intake. 167](#_Toc39264023)

[Figure S-35B. Exposure levels and RRE-SP20 for renal cancer using cumulative intake. 168](#_Toc39264024)

[Figure S-36A. Exposure levels and RRE-US20 for renal cancer using daily intake. 169](#_Toc39264025)

[Figure S-36B. Exposure levels and RRE-SP20 for renal cancer using daily intake. 170](#_Toc39264026)

[Figure S-37A. Exposure levels and RRE-US20 for renal cancer using creatinine adjusted urine concentration. 171](#_Toc39264027)

[Figure S-37B. Exposure levels and RRE-SP20 for renal cancer using creatinine adjusted urine concentration. 172](#_Toc39264028)

[Figure S-38A. Exposure levels and RRE-US20 for renal cancer using water concentration. 173](#_Toc39264029)

[Figure S-38B. Exposure levels and RRE-SP20 for renal cancer using water concentration. 174](#_Toc39264030)

[Figure S-39A. Exposure levels and RRE-US20 for skin cancer using cumulative exposure. 177](#_Toc39264031)

[Figure S-39B. Exposure levels and RRE-SP20 for skin cancer using cumulative exposure. 178](#_Toc39264032)

[Figure S-40A. Exposure levels and RRE-US20 for skin cancer using water concentration. 179](#_Toc39264033)

[Figure S-40B. Exposure levels and RRE-SP20 for skin cancer using water concentration. 180](#_Toc39264034)

[Figure S-41A. Exposure levels and RRE-US20 for skin lesions using cumulative exposure. 184](#_Toc39264035)

[Figure S-41B. Exposure levels and RRE-SP20 for skin lesions using cumulative exposure. 185](#_Toc39264036)

[Figure S-42A. Exposure levels and RRE-US20 for skin lesions using daily intake. 186](#_Toc39264037)

[Figure S-42B. Exposure levels and RRE-SP20 for skin lesions using daily intake. 187](#_Toc39264038)

[Figure S-43A. Exposure levels and RRE-US20 for skin lesions using creatinine adjusted urine concentration. 188](#_Toc39264039)

[Figure S-43B. Exposure levels and RRE-SP20 for skin lesions using creatinine adjusted urine concentration. 189](#_Toc39264040)

[Figure S-44A. Exposure levels and RRE-US20 for skin lesions using urine concentration. 190](#_Toc39264041)

[Figure S-44B. Exposure levels and RRE-SP20 for skin lesions using urine concentration. 191](#_Toc39264042)

[Figure S-45A. Exposure levels and RRE-US20 for skin lesions using water concentration. 192](#_Toc39264043)

[Figure S-45B. Exposure levels and RRE-SP20 for skin lesions using water concentration. 193](#_Toc39264044)

[Figure S-46 Example of non-positive exposure-response from Bates et al. 1995 195](#_Toc39264045)

[Figure S-47 Example of model uncertainty with Michaelis Menton and Exponential 4 models from dataset in Rahman 2010. 196](#_Toc39264046)

**ABBREVIATIONS**

| adj. | adjusted | LBW | low birth weight |
| --- | --- | --- | --- |
| avg. | average, averaged | LOD | limit of detection |
| BMI | body mass index | meas. | measure, measures |
| CAI | cumulative arsenic index | mult. | multiple |
| CI, CIs | confidence interval, confidence intervals | NA | not applicable |
| coeff. | coefficient, coefficients | no. | number |
| conc. | concentration, concentrations | nos. | numbers |
| corr. | Correlation | pop. | population |
| cum. | Cumulative | preg. | pregnancy, pregnancies |
| DW | drinking water | ref. | referents |
| exp. | Exposure | regr. | regression |
| HEALS | Health Effects of Arsenic Longitudinal Study | RR | relative risk |
| iAs | inorganic arsenic | SES | socioeconomic status |
| IgG | immunoglobulin G | tot. | total |
| incl. | Including | TWA | time-weighted average |
| IRR | incidence rate ratio |  |  |

# Study Selection Summary Tables

#### Overview

This appendix presents the results of the processes the authors used to select and model studies for the exposure-response margin of exposure (MOE)-like analyses that involves estimating a health outcome and study-specific estimate of chemical exposure associated with a 20% increase in relative risk (RRE20) and dividing it by an estimate of the U.S. background exposure in the same units (called an RRB in the manuscript).

The 11 health outcomes considered for dose-response analysis in this manuscript were selected based on an analysis and synthesis of the available evidence using systematic review methods, as described in Lee et al. (in preparation). In the Lee at el. analysis, the strength of the published epidemiological evidence supporting an association with iAs exposure was determined to be adequate for a 12^th^ outcome as well, developmental neurotoxicity (particularly effects on cognitive function), but the RRB approach described here is not applicable to this health category because it is generally characterized by continuous response measures (e.g., IQ) and not RR or OR estimates. Also, RRBs could not be derived for one of the 11 health outcomes considered, immune system effects, due to a lack of adequate dose-response information for use in RRB derivations. The factors and criteria for selecting studies for dose-response analysis in this manuscript are summarized in Table S-1 through Table S-11.

#### Study Selection Results

#### Bladder Cancer

Table S-1. Bladder cancer exposure-response study selection

| Study | Initial Screen. Rec. | Rationale for Initial Exclusion | Endpoint (I, M) | Exposure | | Est. Adj. (Smoking, Gender, Age) | Number | | Exposure | | Ref. Grp. Represent. | Sufficient Cases (Nos.) | Mark-downs | All Data  Available  for DR? | **Author Provided Data?** | **Final Rec.** |
| --- | --- | --- | --- | --- | --- | --- | --- | --- | --- | --- | --- | --- | --- | --- | --- | --- |
|  |  |  |  | **Ascertain.** | **Uncertainty** |  | **Exp. Grps.** | **Subj., Cases Rept’d.** | **Metric** | **Timing, Dur.** |  |  |  |  |  |  |
| [Baastrup et al. (2008)](#_ENREF_20) | consider |  | S | LS (M) | LS (regr.) | LS (S) | LS (regr.) | LS | S | S | NA | S | 5 |  |  | exclude |
| [Baris et al. (2016)](#_ENREF_22) | consider |  | S | S–LS | LS (range) | S | S | S | S | S | S | S | 1.5 |  |  | include |
| [Bates et al. (1995)](#_ENREF_24) | consider |  | S | S | LS (ranges)^2^ | S | S | NA | S | S | S | S | 1 |  |  | include |
| [Bates et al. (2004)](#_ENREF_23) | consider |  | S | LS (M) | S | LS (S)^3^ | S | NA | LS | S | S | S | 3 |  |  | include |
| [Besuschio et al. (1980)](#_ENREF_28) | exclude | ecological |  |  |  |  |  |  |  |  |  |  |  |  |  | exclude |
| [Buchet and Lison (1998)](#_ENREF_40) | exclude | ecological |  |  |  |  |  |  |  |  |  |  |  |  |  | exclude |
| [Chen and Wang (1990)](#_ENREF_55) | exclude | ecological |  |  |  |  |  |  |  |  |  |  |  |  |  | exclude |
| [Chen et al. (1985)](#_ENREF_54) | exclude | ecological |  |  |  |  |  |  |  |  |  |  |  |  |  | exclude |
| [Chen et al. (1986)](#_ENREF_50) | exclude | exp. assessment based on yrs. of residence in a blackfoot disease-endemic area with no measured iAs concs. |  |  |  |  |  |  |  |  |  |  |  |  |  | exclude |
| [Chen et al. (1988)](#_ENREF_52) | exclude | ecological |  |  |  |  |  |  |  |  |  |  |  |  |  | exclude |
| [Chen et al. (1992)](#_ENREF_48) | exclude | ecological |  |  |  |  |  |  |  |  |  |  |  |  |  | exclude |
| [Chen et al. (2003b)](#_ENREF_64) | consider |  | S | LS (M, q) | LS (ranges) | S | S | NA | S | LS | S | S | 3 |  |  | include |
| [Chen et al. (2010b)](#_ENREF_57) | consider |  | S | S | LS (ranges) | S | S | S | LS^5^ | S | NA | S | 2 |  |  | include |
| [Chiang et al. (1993)](#_ENREF_78) | exclude | ecological |  |  |  |  |  |  |  |  |  |  |  |  |  | exclude |
| [Chiou et al. (1995)](#_ENREF_82) | consider |  | S | LS (M) | LS (ranges) | LS (blackfoot disease)^4^ | S | S | S | S | NA | S | 3 | no, tot. no. subjects per exp. stratum not provided (provided person-yrs.) |  | exclude |
| [Chiou et al. (2001a)](#_ENREF_80) | consider |  | S | LS | LS (ranges) | S | S | S | LS | S | NA | LS | 4 | number of subjects not provided |  | exclude |
| [Chung et al. (2011)](#_ENREF_89) | consider |  | S | S (U) | LS (ranges) | S | S | NA | S | LS (current only) | S | S | 2 |  |  | include |
| {Chung, 2012, 1453800@@author-year} | consider |  | LS | LS (M) | LS (ranges) | S | S | LS7 | S | S | NA | LS | 5 | no, tot. no. subjects per exp. stratum not provided (provided person-yrs.) |  | exclude |
| [Chung et al. (2013)](#_ENREF_88) | consider |  | S | S (U) | LS (ranges) | S | S | NA | S | LS | S | S | 2 |  |  | include |
| [D'Ippoliti et al. (2015)](#_ENREF_92) | consider | note: no data on number of subjects per exp. stratum; requested information from study authors | LS (mortal-ity) | LS | LS (range) | S | LS  (2 + ref.) | S | S | S | NA | S | 4 | Number of subjects not provided | Number of subjects per exposure stratum | include |
| [Feki-Tounsi et al. (2013)](#_ENREF_115) | consider |  | S | LS (blood) | S | S (males) | LS, 2 | NA | LS (blood) | S | LS | S | 4 | two exposure groups only |  | exclude |
| [Fernández et al. (2012)](#_ENREF_117) | exclude | ecological |  |  |  |  |  |  |  |  |  |  |  |  |  | exclude |
| [Ferreccio et al. (2013b)](#_ENREF_121) | consider |  | S | LS (M) | LS (ranges) | S | LS, 2, disjoint | NA | LS | S | S | S | 4 | no, only 2 exp. groups |  | exclude |
| [Gunduz et al. (2015)](#_ENREF_142) | exclude | no data for exposure-response |  |  |  |  |  |  |  |  |  |  |  |  |  | exclude |
| [Guo et al. (1997)](#_ENREF_145) | exclude | ecological |  |  |  |  |  |  |  |  |  |  |  |  |  | exclude |
| [Han et al. (2009)](#_ENREF_155) | exclude | ecological |  |  |  |  |  |  |  |  |  |  |  |  |  | exclude |
| [Hinwood et al. (1999)](#_ENREF_162) | exclude | ecological |  |  |  |  |  |  |  |  |  |  |  |  |  | exclude |
| [Hopenhayn-Rich et al. (1996)](#_ENREF_164) | exclude | ecological |  |  |  |  |  |  |  |  |  |  |  |  |  | exclude |
| [Hopenhayn-Rich et al. (1998)](#_ENREF_165) | exclude | ecological |  |  |  |  |  |  |  |  |  |  |  |  |  | exclude |
| [Hsu et al. (2008)](#_ENREF_173) | consider |  | S | LS (M) | LS (ranges) | S | S | NA | LS | S | S | S | 3 |  |  | include |
| [Hsu et al. (2013a)](#_ENREF_171) | exclude | main purpose to evaluate relationship with skin lesions, at least 1/4 of subjects had missing exp. information |  |  |  |  |  |  |  |  |  |  |  |  |  | exclude |
| [Huang et al. (2008b)](#_ENREF_184) | consider |  | S | S (U) | LS (ranges) | S | S | NA | S | LS | S | S | 2 |  |  | include |
| [Huang et al. (2008a)](#_ENREF_183) | consider |  | S | LS (M) | LS (range) | S | S (3 + ref.) | S | S | S | NA | LS | 3 |  |  | include |
| [Karagas et al. (2004)](#_ENREF_201) | exclude | non-PBPK dose metric |  |  |  |  |  |  |  |  |  |  |  |  |  | exclude |
| [Kurttio et al. (1999)](#_ENREF_211) | consider |  | S | LS (M)^1^ | LS (ranges) | S | S | NA | S | S | S | S | 2 | no, tot. no. subjects per exp. stratum not provided (provided person-yrs.) |  | exclude |
| [Lamm et al. (2003)](#_ENREF_220) | exclude | ecological |  |  |  |  |  |  |  |  |  |  |  |  |  | exclude |
| [Lamm et al. (2004)](#_ENREF_221) | exclude | ecological |  |  |  |  |  |  |  |  |  |  |  |  |  | exclude |
| [Lewis et al. (1999)](#_ENREF_225) | exclude | relatively high exp. uncertainty; too few bladder cancer deaths (tot. 5) |  |  |  |  |  |  |  |  |  |  |  |  |  | exclude |
| [Marshall et al. (2007)](#_ENREF_253) | exclude | ecological |  |  |  |  |  |  |  |  |  |  |  |  |  | exclude |
| [Meliker et al. (2007)](#_ENREF_262) | exclude | ecological |  |  |  |  |  |  |  |  |  |  |  |  |  | exclude |
| [Meliker et al. (2010)](#_ENREF_261) | consider |  | S | LS (KR) | LS (ranges) | S | S | NA | S | S | S | S | 2 |  |  | include |
| [Michaud et al. (2004)](#_ENREF_264) | exclude | toenail arsenic |  |  |  |  |  |  |  |  |  |  |  |  |  | exclude |
| [Mikoczy et al. (1996)](#_ENREF_265) | exclude | only 2 exp. groups, referent & >0.5 & very few cases (only 6 tot. kidney cancer cases) |  |  |  |  |  |  |  |  |  |  |  |  |  | exclude |
| [Morales et al. (2000)](#_ENREF_272) | exclude | ecological |  |  |  |  |  |  |  |  |  |  |  |  |  | exclude |
| [Pou et al. (2011)](#_ENREF_308) | exclude | ecological |  |  |  |  |  |  |  |  |  |  |  |  |  | exclude |
| [Pu et al. (2007)](#_ENREF_309) | consider |  | S | S (U) | LS (ranges, SE) | S | S | NA | S | LS | S | S | 2 |  |  | include |
| [Rivara et al. (1997)](#_ENREF_330) | exclude | ecological |  |  |  |  |  |  |  |  |  |  |  |  |  | exclude |
| [Sawada et al. (2013)](#_ENREF_335) | consider |  | S | LS^6^ | S | S | S | S | S | LS (recent only) | NA | S | 2 |  |  | include |
| [Smith et al. (1998)](#_ENREF_344) | exclude | ecological |  |  |  |  |  |  |  |  |  |  |  |  |  | exclude |
| [Smith et al. (2012)](#_ENREF_345) | exclude | ecological |  |  |  |  |  |  |  |  |  |  |  |  |  | exclude |
| [Steinmaus et al. (2003)](#_ENREF_354) | consider |  | S | LS (M) | LS (ranges, 3-4) | S | S | NA | S | S | S | S | 2 |  |  | include |
| [Steinmaus et al. (2013)](#_ENREF_356) | consider |  | S | LS (M) | LS (ranges, 4) | S | S | NA | S | S | S | S | 2 |  |  | include |
| [Steinmaus et al. (2014a)](#_ENREF_352) | consider |  | S | LS (M) | LS (ranges, SD) | S | S | NA | S | S | S | S | 2 |  |  | include |
| [Su et al. (2011)](#_ENREF_358) | exclude | ecological |  |  |  |  |  |  |  |  |  |  |  |  |  | exclude |
| [Tsai et al. (1999)](#_ENREF_364) | exclude | ecological |  |  |  |  |  |  |  |  |  |  |  |  |  | exclude |
| [Tsuda et al. (1995)](#_ENREF_371) | exclude | tot. 3 cancer deaths |  |  |  |  |  |  |  |  |  |  |  |  |  | exclude |
| [Wadhwa et al. (2011b)](#_ENREF_381) | exclude | no data for exposure-response |  |  |  |  |  |  |  |  |  |  |  |  |  | exclude |
| [Wang et al. (2009c)](#_ENREF_389) | consider |  | S | LS (M) | LS (high/low) | S | LS, 2 | NA | LS | LS | S | S | 5 | only 2 exp. groups |  | exclude |
| [Wu et al. (1989)](#_ENREF_397) | exclude | ecological |  |  |  |  |  |  |  |  |  |  |  |  |  | exclude |
| [Wu et al. (2012a)](#_ENREF_394) | consider |  | S | S (U) | LS (ranges, 2) | S | LS, 2 | NA | S | LS | S | S | 3 | only 2 exp. groups |  | exclude |
| [Wu et al. (2013)](#_ENREF_393) | consider |  | S | S (U) | LS (ranges) | S | S | NA | S | LS | S | S | 2 |  |  | include |
| [Yang et al. (2005)](#_ENREF_402) | exclude | ecological |  |  |  |  |  |  |  |  |  |  |  |  |  | exclude |
| [Yang et al. (2013)](#_ENREF_404) | exclude | same data as Chen 2010 (reanalysis); |  |  |  |  |  |  |  |  |  |  |  |  |  | exclude |
| [Yorifuji et al. (2011)](#_ENREF_406) | exclude | ecological |  |  |  |  |  |  |  |  |  |  |  |  |  | exclude |

S = suitable; LS = less suitable; NS = not suitable

1. Qualifiers for Exposure Ascertainment:

M = municipal or village water average

KR = kriged average

U = urinary arsenic

toes = toenail arsenic

blood = blood arsenic

Inh = inhalation with no assessment of other routes of exposure

W = work history + estimated mg/m^3^ levels for different jobs and timeframes

2. Qualifiers for Exposure Uncertainty

(ranges) = exposure presented as ranges (numbers indicate numbers of strata)

SD, SE = standard deviation, standard error

3. Does not adjust for gender

4. Results might be affected by blackfoot disease incidence

5. Risks as a function of cumulative exposure only for total urinary cancer (not urothelial carcinoma)

6. Total creatinine-adjusted urinary arsenic reported, but no speciation and no documentation of fish consumption (to determine arsenobetaine and arsenocholine levels are not abnormally high)

7. Number of deaths, but not number of subjects, given for each exposure stratum

8. Averages reported but no measures of dispersion

9. Death rates not adjusted for effect of smoking due to lack of smoking information

#### Diseases of the Circulator System

Table S-2. Diseases of the circulatory system exposure-response study selection

| **Study** | **Initial Screen. Rec.** | **Rationale for Initial Exclusion** | **End-point (I, M)** | **Exposure** | | **Est. Adj. (Smoking, Gender, Age)** | **Number** | | **Exposure** | | **Ref. Grp. Represent.** | **Sufficient Cases (Nos.)** | **Mark-downs** | **All Data  Available  for DR?** | **Author Provided Data?** | **Final Rec.** |
| --- | --- | --- | --- | --- | --- | --- | --- | --- | --- | --- | --- | --- | --- | --- | --- | --- |
|  |  |  |  | **Ascertain.** | **Uncertainty** |  | **Exp. Grps.** | **Subj., Cases Rept’d.** | **Metric** | **Timing, Dur.** |  |  |  |  |  |  |
| [Ahmad et al. (2006)](#_ENREF_6) | exclude | no data for exposure-response |  |  |  |  |  |  |  |  | NA |  |  |  |  | exclude |
| [Ameer et al. (2015)](#_ENREF_13) | exclude | cross-sectional |  |  |  |  |  |  |  |  |  |  |  |  |  | exclude |
| [Axelson et al. (1978)](#_ENREF_19) | consider |  | LS | LS (Inh) | LS  (no actual estimates in mg/m^3^) | LS | S  (3+ref) | S | LS | S | LS | S | 6 |  |  | exclude |
| [Bencko et al. (1980)](#_ENREF_27) | exclude | no data for exposure-response |  |  |  |  |  |  |  |  | NA |  |  |  |  | exclude |
| [Bošnjak et al. (2008)](#_ENREF_37) | exclude | only provides corr. coeff. between urinary As & cardiovascular markers (e.g., cholesterol, triglycerides, homocysteine), & none were significant |  |  |  |  |  |  |  |  | NA |  |  |  |  | exclude |
| [Buchet and Lison (1998)](#_ENREF_40) | exclude | ecological |  |  |  |  |  |  |  |  | NA |  |  |  |  | exclude |
| [Burgess et al. (2013)](#_ENREF_42) | exclude | only provides corr. coeff. between urinary As & cardiovascular markers (e.g., MMP-9) |  |  |  |  |  |  |  |  | NA |  |  |  |  | exclude |
| [Chang et al. (2004)](#_ENREF_46) | exclude | Ecological |  |  |  |  |  |  |  |  | NA |  |  |  |  | exclude |
| [Chen et al. (1988)](#_ENREF_52) | exclude | Ecological |  |  |  |  |  |  |  |  | NA |  |  |  |  | exclude |
| [Chen et al. (1995)](#_ENREF_51) | exclude | cross-sectional |  |  |  |  |  |  |  |  |  |  |  |  |  | exclude |
| [Chen et al. (1996)](#_ENREF_49) | consider |  | LS  (M) | LS  (M) | LS | S | S  (3+ref) | S | S | S | NA | LS | 4 |  |  | Include |
| [Chen et al. (2006b)](#_ENREF_69) | exclude | screening study |  |  |  |  |  |  |  |  |  |  |  |  |  | exclude |
| [Chen et al. (2007a)](#_ENREF_66) | exclude | cross-sectional |  |  |  |  |  |  |  |  |  |  |  |  |  | exclude |
| [Chen et al. (2011b)](#_ENREF_68) | consider |  | LS  (M) | S | S  (range w/mean) | S | S  (3+ref) | S | S | S | NA | S | 1 |  |  | Include |
| [Chen et al. (2012b)](#_ENREF_61) | exclude | cross-sectional |  |  |  |  |  |  |  |  |  |  |  |  |  | Exclude |
| [Chen et al. (2013a)](#_ENREF_71) | exclude | cross-sectional |  |  |  |  |  |  |  |  |  |  |  |  |  | exclude |
| [Chen et al. (2013b)](#_ENREF_72) | consider |  | S | S–LS^3^ | S  (range w/mean) | S | LS  (2 + ref) | S | S | S | S | S | 1.5 |  |  | include |
| [Chen et al. (2013c)](#_ENREF_73) | consider |  | S | S | S  (range w/mean) | S | S  (3+ref) | S | S | S | NA | S | 0 |  |  | Include |
| [Cheng et al. (2010)](#_ENREF_75) | exclude | ecological |  |  |  |  |  |  |  |  | NA |  |  |  |  | exclude |
| [Chiou et al. (1997)](#_ENREF_81) | exclude | cross-sectional |  |  |  |  |  |  |  |  |  |  |  |  |  | exclude |
| [Chiou et al. (2001b)](#_ENREF_83) | exclude | cross-sectional |  |  |  |  |  |  |  |  |  |  |  |  |  | exclude |
| [Chiou et al. (2005)](#_ENREF_84) | consider |  | S  (P) | LS  (M) | LS  (regr.) | S^4^ | LS (regr.) | LS | LS | LS | NA | S | 6 | no, regr. coeff. only |  | exclude |
| [Chiu et al. (2007)](#_ENREF_87) | exclude | Ecological |  |  |  |  |  |  |  |  | NA |  |  |  |  | exclude |
| [Das et al. (2012)](#_ENREF_94) | exclude | no data for exposure-response |  |  |  |  |  |  |  |  | NA |  |  |  |  | exclude |
| [Dastgiri et al. (2010)](#_ENREF_95) | exclude | Ecological |  |  |  |  |  |  |  |  | NA |  |  |  |  | exclude |
| [D'Ippoliti et al. (2015)](#_ENREF_92) | consider | no data on no. subjects per exp. stratum; requested information from study authors | LS (mortal-ity) | LS | LS  (range) | S | LS (2+ref) | S | S | S | NA | S | 4 | number of subjects not provided | author provided number of subjects per exposure stratum | include |
| [Engel and Smith (1994)](#_ENREF_103) | exclude | Ecological |  |  |  |  |  |  |  |  | NA |  |  |  |  | exclude |
| [Farzan et al. (2015a)](#_ENREF_109) | consider |  | LS (M) | S (toenail) | LS | LS  (not clear if age or gender adj. for) | LS | S  (no. deaths, tot. participants, person yrs. provided) | S | S | NA | S | 4 | continuous measure of exposure |  | exclude |
| [Farzan et al. (2015b)](#_ENREF_110) | consider | if consider blood pressure as a viable endpoint | S | S | LS (regr.) | S | LS  (regr.) | S  (provides tot. no. subjects, but table w/betas provides no. blood pressure measure-ments) | S | S | NA | S | 2 | no, betas only |  | exclude |
| [Ghosh (2013)](#_ENREF_127) | exclude | no data for exposure-response |  |  |  |  |  |  |  |  |  |  |  |  |  | exclude |
| [Gong and O'Bryant (2012)](#_ENREF_130) | exclude | need original data to model, GIS estimates w/a small range (2.2–15.3 µg/L), likely exp. misclassification |  |  |  |  |  |  |  |  | NA |  |  |  |  | exclude |
| [Guha Mazumder et al. (2012)](#_ENREF_135) | exclude | compares exposed & unexposed; divided exposed group into 2 categories, but considers unexposed to have 0 cum. exp. |  |  |  |  |  |  |  | LS | NA |  |  |  |  | exclude |
| [Gunduz et al. (2015)](#_ENREF_142) | exclude | no data for exposure-response |  |  |  |  |  |  |  |  | NA |  |  |  |  | exclude |
| [Guo et al. (2007)](#_ENREF_149) | exclude | no data for dose response |  |  |  |  |  |  |  | LS | NA |  |  |  |  | exclude |
| [Hawkesworth et al. (2013)](#_ENREF_158) | consider | would need to get original data | S | S | LS (regr.) | S^5^ | LS (regr.) | LS | S | S | NA | S | 3 | no, regr. coeff. only |  | exclude |
| [Hertz-Picciotto et al. (2000)](#_ENREF_161) | consider |  | LS  (M) | LS  (W) | LS  (range) | S^4^ | S  (5+ref) | LS | S (cum.) | S | NA | S | 4 | CVD rate ratios not suitable for Tier 1 |  | exclude |
| [Hsieh et al. (2008a)](#_ENREF_169) | exclude | no data for exposure-response |  |  |  |  |  |  |  |  |  |  |  |  |  | exclude |
| [Hsieh et al. (2008b)](#_ENREF_170) | consider |  | S | S | LS  (range) | S^4^ | LS (2+ref) | S | S (cum.) | LS | S | S | 3 |  |  | include |
| [Hsueh et al. (1998)](#_ENREF_175) | consider |  | S | LS  (M) | LS  (range) | S^4^ | LS (2+ref) | S | LS | S | S | S | 4 |  |  | Include |
| [Islam et al. (2012a)](#_ENREF_188) | exclude | cross-sectional |  |  |  |  |  |  |  |  |  |  |  |  |  | exclude |
| [James et al. (2015)](#_ENREF_190) | consider |  | S | LS | LS | S  (age not adj. for but same across exp. groups) | S  (3+ref) | S | LS | S | S | LS | 4 |  |  | include |
| [Jarup et al. (1989)](#_ENREF_193) | consider | inhalation study w/poor exp. characterization | LS  (M) | LS  (W) | LS  (range) | LS | S  (6+ref) | S | S (cum.) | S | NA | S  (IHD);  LS (cerebro) | 4 | no, number of subjects per exp. group not provided |  | exclude |
| [Jensen and Hansen (1998)](#_ENREF_194) | exclude | no data for exposure-response |  |  |  |  |  |  |  | LS | NA |  |  |  |  | exclude |
| [Jiang et al. (2015)](#_ENREF_195) | exclude | change in blood pressure per yr. (HEALS) |  |  |  |  |  |  |  |  |  |  |  |  |  | exclude |
| [Jones et al. (2011)](#_ENREF_196) | exclude | cross-sectional |  |  |  |  |  |  |  |  |  |  |  |  |  | exclude |
| [Jovanović et al. (2012)](#_ENREF_199) | exclude | ecological |  |  |  |  |  |  |  |  | NA |  |  |  |  | exclude |
| [Karim et al. (2013)](#_ENREF_202) | exclude | basically compares exposed & unexposed; divided exposed group into 3 categories, but control & low group overlap, other results were just regressions. |  |  |  |  |  |  |  | LS | NA |  |  |  |  | exclude |
| [Kunrath et al. (2013)](#_ENREF_210) | exclude | no data for exposure-response |  |  |  |  |  |  |  | LS | NA |  |  |  |  | exclude |
| [Kwok et al. (2007)](#_ENREF_215) | exclude | cross-sectional |  |  |  |  |  |  |  |  |  |  |  |  |  | exclude |
| [Lagerkvist et al. (1986)](#_ENREF_216) | exclude | no data for exposure-response |  |  |  |  |  |  |  | LS | NA |  |  |  |  | exclude |
| [Lagerkvist et al. (1988)](#_ENREF_217) | exclude | no data for exposure-response |  |  |  |  |  |  |  |  |  |  |  |  |  | exclude |
| [Lewis et al. (1999)](#_ENREF_225) | exclude | poor exp. estimates, some cardiovascular effects decreased, hypertensive heart disease increased, but mainly in low exp. group |  |  |  |  |  |  |  |  | NA |  |  |  |  | exclude |
| [Li et al. (2009)](#_ENREF_226) | exclude | cross-sectional |  |  |  |  |  |  |  |  |  |  |  |  |  | exclude |
| [Li et al. (2013a)](#_ENREF_227) | exclude | cross-sectional |  |  |  |  |  |  |  |  |  |  |  |  |  | exclude |
| [Li et al. (2013b)](#_ENREF_228) | exclude | cross-sectional |  |  |  |  |  |  |  |  |  |  |  |  |  | exclude |
| [Li et al. (2015)](#_ENREF_229) | exclude | cross-sectional |  |  |  |  |  |  |  |  |  |  |  |  |  | exclude |
| [Liao et al. (2009)](#_ENREF_231) | exclude | no data for exposure-response |  |  |  |  |  |  |  |  |  |  |  |  |  | exclude |
| [Liao et al. (2012)](#_ENREF_230) | exclude | no data for exposure-response |  |  |  |  |  |  |  |  | NA |  |  |  |  | exclude |
| [Lisabeth et al. (2010)](#_ENREF_237) | exclude | ecological |  |  |  |  |  |  |  |  | NA |  |  |  |  | exclude |
| [Lubin et al. (1981)](#_ENREF_241) | exclude | exp. not adequately quantified |  |  |  |  |  |  |  |  | NA |  |  |  |  | exclude |
| [Marsh et al. (2009)](#_ENREF_252) | consider |  | LS  (M) | LS  (W) | LS  (range) | S^4^ | S  (4+ref) | LS | S (cum.) | S | NA | S | 4 | no, number of subjects per exp. group not provided |  | exclude |
| [Medrano et al. (2010)](#_ENREF_260) | exclude | ecological |  |  |  |  |  |  |  |  | NA |  |  |  |  | exclude |
| [Meliker et al. (2007)](#_ENREF_262) | exclude | ecological |  |  |  |  |  |  |  |  | NA |  |  |  |  | exclude |
| [Moon et al. (2013)](#_ENREF_270) | consider |  | S  (I & M) | S | S  (range w/median) | S | S  (3+ref) | S | S | S | NA | S | 0 | Number of subjects not provided | Author provided number of subjects per exposure stratum | include |
| [Mordukhovich et al. (2009)](#_ENREF_273) | exclude | toenail arsenic |  |  |  |  |  |  |  | LS | NA |  |  |  |  | exclude |
| [Mumford et al. (2007)](#_ENREF_279) | exclude | cross-sectional |  |  |  |  |  |  |  |  |  |  |  |  |  | exclude |
| [Nabi et al. (2005)](#_ENREF_281) | exclude | no data for exposure-response |  |  |  |  |  |  |  | LS | NA |  |  |  |  | exclude |
| [Osorio-Yáñez et al. (2013)](#_ENREF_290) | exclude | cross-sectional |  |  |  |  |  |  |  |  |  |  |  |  |  | exclude |
| [Osorio-Yáñez et al. (2015)](#_ENREF_291) | exclude | cross-sectional |  |  |  |  |  |  |  |  |  |  |  |  |  | exclude |
| [Pi et al. (2005)](#_ENREF_305) | exclude | no data for exposure-response |  |  |  |  |  |  |  |  | NA |  |  |  |  | exclude |
| [Rahman and Axelson (2001)](#_ENREF_316) | exclude | no data for exposure-response |  |  |  |  |  |  |  | LS | NA |  |  |  |  | exclude |
| [Rahman et al. (1999a)](#_ENREF_319) | consider |  | S  (P) | S | LS  (range) | S^4^ | S  (4+ref) | S | S (cum.) | LS | NA | S | 2 |  |  | include |
| [Rahman et al. (2014)](#_ENREF_317) | consider |  | LS  (M) | S | LS  (range) | S^4^ | LS  (2+ref) | S | LS | S | NA | S | 4 |  |  | include |
| [Skröder et al. (2015)](#_ENREF_342) | exclude | study focuses on cadmium, although some logistic regr. results for arsenic; evaluating effects on kidney function & blood pressure & whether selenium levels alleviate the effect |  |  |  |  |  |  |  |  |  |  |  |  |  | exclude |
| [Smith et al. (2012)](#_ENREF_345) | exclude | ecological |  |  |  |  |  |  |  |  | NA |  |  |  |  | exclude |
| [Sohel et al. (2009)](#_ENREF_349) | consider |  | LS  (M) | S | LS  (range) | LS^4^ | S  (4+ref) | S | LS | S | NA | S | 4 | no, no. of subjects per exp. group not provided | obtained number of subjects from author | include |
| [Tsai et al. (1999)](#_ENREF_364) | exclude | ecological |  |  |  |  |  |  |  |  | NA |  |  |  |  | exclude |
| [Tseng (1977)](#_ENREF_370) | exclude | ecological |  |  |  |  |  |  |  |  | NA |  |  |  |  | exclude |
| [Tseng et al. (1996)](#_ENREF_365) | exclude | cross-sectional |  |  |  |  |  |  |  |  |  |  |  |  |  | exclude |
| [Tseng et al. (1997)](#_ENREF_366) | exclude | cross-sectional |  |  |  |  |  |  |  |  |  |  |  |  |  | exclude |
| [Tseng et al. (2003)](#_ENREF_367) | exclude | cross-sectional |  |  |  |  |  |  |  |  |  |  |  |  |  | exclude |
| [Tseng et al. (2005)](#_ENREF_368) | exclude | no data for exposure-response |  |  |  |  |  |  |  |  | NA |  |  |  |  | exclude |
| [Valentine et al. (1992)](#_ENREF_373) | exclude | ecological |  |  |  |  |  |  |  |  | NA |  |  |  |  | exclude |
| [Varsányi et al. (1991)](#_ENREF_375) | exclude | ecological |  |  |  |  |  |  |  |  | NA |  |  |  |  | exclude |
| [Wade et al. (2009)](#_ENREF_379) | consider |  | LS  (M) | S | LS  (range) | S | S  (4+ref) | S–LS (no. of deaths & rate per 100,000) | LS | S | NA | LS (only 1 in highest group) | 4 |  |  | include |
| [Wade et al. (2015)](#_ENREF_378) | consider |  | S | S | LS | S | LS  (2+ref) | S | LS (curr-ent water or toenail) | S | S | S | 3 |  |  | include |
| [Wang et al. (2002)](#_ENREF_384) | consider |  | S | LS  (M) | LS  (range) | S | LS  (2+ref) | LS | S (cum.) | S | NA | LS | 5 | no, no. of subjects per exp. group not provided |  | exclude |
| [Wang et al. (2003)](#_ENREF_386) | exclude | ecological |  |  |  |  |  |  |  |  | NA |  |  |  |  | exclude |
| [Wang et al. (2007)](#_ENREF_388) | consider |  | S | LS  (M) | LS  (range) | S | LS (2+ref) | S | S | LS | S | S | 4 |  |  | include |
| [Wang et al. (2009a)](#_ENREF_382) | exclude | cross-sectional |  |  |  |  |  |  |  |  |  |  |  |  |  | exclude |
| [Wang et al. (2009b)](#_ENREF_385) | exclude | no data for exposure-response |  |  |  |  |  |  |  | LS | NA |  |  |  |  | exclude |
| [Wang et al. (2010)](#_ENREF_383) | consider |  | S | LS  (M) | LS  (range) | LS | LS (2+ref) | S | S (cum.) | S | NA | S | 4 | confidence intervals not available |  | exclude |
| [Wang et al. (2011)](#_ENREF_387) | consider |  | S | S (U), LS (M, water) | LS  (range) | S | LS (2+ref) | LS | S | S (water), LS (U) | NA | S | 4 | no, only cases available |  | exclude |
| [Welch et al. (1982)](#_ENREF_391) | consider | consider; inhalation study w/poor exp. characterization | LS | LS  (W) | LS  (range) | S | S  (3+ref) | S | LS (TWA & ceiling) | S | NA | S | 4 | only mortality ratios reported |  | exclude |
| [Wu et al. (1989)](#_ENREF_397) | exclude | ecological |  |  |  |  |  |  |  |  | NA |  |  |  |  | exclude |
| [Wu et al. (2006)](#_ENREF_396) | consider |  | S | S | LS  (range) | S | LS  (2+ ref) | S | S (cum.) | S | S | S | 2 |  |  | include |
| [Wu et al. (2010)](#_ENREF_398) | consider |  | S | S (Lanyang cohort, house-hold wells)  LS  (LMN cohort; M) | LS  (range) | S | S (Lanyang, 4+ref); LS (LMN cohort, 2+ref) | S | LS (avg.) | S | S | S | 2  (Lan-yang cohort) 4  (LMN cohort) |  |  | include |
| [Wu et al. (2012b)](#_ENREF_395) | exclude | cross-sectional |  |  |  |  |  |  |  |  |  |  |  |  |  | exclude |
| [Xia et al. (2009)](#_ENREF_399) | exclude | exclude for cardio-vascular, good study for skin lesions, but only OR per 50-µg/L increase in arsenic provided for cardiovascular disease |  |  |  |  |  |  |  | LS | NA |  |  |  |  | exclude |
| [Yang (2006)](#_ENREF_400) | exclude | ecological |  |  |  |  |  |  |  |  | NA |  |  |  |  | exclude |
| [Yildiz et al. (2008)](#_ENREF_405) | exclude | no data for exposure-response |  |  |  |  |  |  |  | LS | NA |  |  |  |  | exclude |
| [Yoshikawa et al. (2008)](#_ENREF_407) | consider |  | LS | LS | LS | LS | S | S | LS | LS | NA | S | 6 |  |  | exclude |
| [Yuan et al. (2007)](#_ENREF_410) | exclude | ecological |  |  |  |  |  |  |  |  | NA |  |  |  |  | exclude |
| [Zhang et al. (2013)](#_ENREF_411) | exclude | exp. only based on duration |  |  |  |  |  |  |  | LS | NA |  |  |  |  | exclude |
| [Zierold et al. (2004)](#_ENREF_412) | exclude | cross-sectional |  |  |  |  |  |  |  |  |  |  |  |  |  | exclude |

S = suitable; LS = less suitable; NS = not suitable

1. Qualifiers for Exposure Ascertainment:

M = municipal or village water average

KR = kriged average

U = urinary arsenic

toes = toenail arsenic

blood = blood arsenic

inh = inhalation with no assessment of other routes of exposure

W = work history + estimated mg/m^3^ levels for different jobs and timeframes

2. Qualifiers for Exposure Uncertainty

(ranges) = exposure presented as ranges (numbers indicate numbers of strata)

SD, SE = standard deviation, standard error

3. Does not adjust for gender

4. Results might be affected by blackfoot disease incidence

5. Risks as a function of cumulative exposure only for total urinary cancer (not urothelial carcinoma)

6. Total creatinine-adjusted urinary arsenic reported, but no speciation and no documentation of fish consumption (to determine arsenobetaine and arsenocholine levels are not abnormally high)

7. Number of deaths, but not number of subjects, given for each exposure stratum

8. Averages reported but no measures of dispersion

9. Death rates not adjusted for effect of smoking due to lack of smoking information

#### Lung Cancer

Table S-3. Lung cancer exposure-response study selection

| **Study** | **Initial Screen. Rec.** | **Rationale for Initial Exclusion** | **End-point (I, M)** | **Exposure** | | **Est. Adj. (Smoking, Gender, Age)** | **Number** | | **Exposure** | | **Ref. Grp. Represent.** | **Sufficient Cases (Nos.)** | **Mark-downs** | **All Data  Available  for DR?** | **Author Provided Data?** | **Final Rec.** |
| --- | --- | --- | --- | --- | --- | --- | --- | --- | --- | --- | --- | --- | --- | --- | --- | --- |
|  |  |  |  | **Ascertain.** | **Uncertainty** |  | **Exp. Grps.** | **Subj., Cases Rept’d.** | **Metric** | **Timing, Dur.** |  |  |  |  |  |  |
| [Ades and Kazantzis (1988)](#_ENREF_2) | exclude | exp. not adequately quantified |  |  |  |  |  |  |  |  |  |  |  |  |  | exclude |
| [Argos et al. (2014)](#_ENREF_17) | consider | note: large study but very high exp. uncertainty & mortality endpoint | LS | S–LS (U)^6^ | LS; | S | LS, 2, | S | S | LS | NA | S | 4.5 | subjects per exp. stratum not provided; approximated |  | include |
| [Axelson et al. (1978)](#_ENREF_19) | exclude | exp. not adequately quantified |  |  |  |  |  |  |  |  |  |  |  |  |  | exclude |
| [Baastrup et al. (2008)](#_ENREF_20) | consider | note: large study, but high exp. uncertainty compared to range | S | LS (M) | LS (regr.) | LS (S) | LS (regr.) | LS | S | LS | NA | S | 6 |  |  | exclude |
| [Besuschio et al. (1980)](#_ENREF_28) | exclude | ecological |  |  |  |  |  |  |  |  |  |  |  |  |  | exclude |
| [Buchet and Lison (1998)](#_ENREF_40) | exclude | ecological |  |  |  |  |  |  |  |  |  |  |  |  |  | exclude |
| [Bulbulyan et al. (1996)](#_ENREF_41) | exclude | exp. not adequately quantified |  |  |  |  |  |  |  |  |  |  |  |  |  | exclude |
| [Chen and Chen (2002)](#_ENREF_62) | consider |  | LS (mortal-ity) | S–LS | LS | S–LS (matched for age, i.e., decade of birth, but did not adj.) | S | S | S | S | S | S | 3 |  |  | include |
| [Chen and Wang (1990)](#_ENREF_55) | exclude | Ecological |  |  |  |  |  |  |  |  |  |  |  |  |  | exclude |
| [Chen et al. (1985)](#_ENREF_54) | exclude | Ecological |  |  |  |  |  |  |  |  |  |  |  |  |  | exclude |
| [Chen et al. (1986)](#_ENREF_50) | exclude | exp. assessment based on yrs. of residence in a blackfoot disease-endemic area with no measured iAs concs. |  |  |  |  |  |  |  |  |  |  |  |  |  | exclude |
| [Chen et al. (1988)](#_ENREF_52) | exclude | Ecological |  |  |  |  |  |  |  |  |  |  |  |  |  | exclude |
| [Chen et al. (1992)](#_ENREF_48) | exclude | Ecological |  |  |  |  |  |  |  |  |  |  |  |  |  | exclude |
| [Chen et al. (2004)](#_ENREF_53) | consider |  | S | LS (M) | LS (ranges) | S | S | S | LS | S | NA | S | 3 |  |  | include |
| [Chen et al. (2010a)](#_ENREF_56) | consider | include; perhaps the best overall cohort study | S | S | LS (ranges) | S | S | S | LS^5^ | S | NA | S | 2 |  |  | include |
| [Chen et al. (2014)](#_ENREF_60) | exclude | relative risk for cancer mortality based on 1 ppm increase in soil As |  |  |  |  |  |  |  |  |  |  |  |  |  | exclude |
| [Chiazze et al. (1997)](#_ENREF_79) | exclude | only 2 exp. groups, referent & >0.001 |  |  |  |  |  |  |  |  |  |  |  |  |  | exclude |
| [Chiou et al. (1995)](#_ENREF_82) | consider | note: carefully evaluate covariate effects, especially blackfoot disease | S | LS (M) | LS (ranges) | LS (blackfoot disease)^4^ | S | S | S | S | NA | S | 3 | no, tot. n per exp. stratum not available |  | exclude |
| [Chiu et al. (2004)](#_ENREF_86) | exclude | Ecological |  |  |  |  |  |  |  |  |  |  |  |  |  | exclude |
| {Chung, 2012, 1453800@@author-year} | consider |  | LS | LS (M) | LS (ranges) | S | S | LS^7^ | S | S | NA | LS | 5 | no, tot. n per exp. stratum not available |  | exclude |
| [Dauphiné et al. (2013)](#_ENREF_97) | consider |  | S | LS | LS (range) | S | LS (2+ref) | S | LS | S | NA | S | 4 |  |  | include |
| [D'Ippoliti et al. (2015)](#_ENREF_92) | consider | note: no data on number of subjects per exp. stratum; requested information from study authors | LS (mortal-ity) | LS | LS (range) | S | LS (2+ref) | S | S | S | NA | S | 4 | number of subjects not reported | author provided number of subjects per exposure stratum | include |
| [Engel and Smith (1994)](#_ENREF_103) | exclude | ecological |  |  |  |  |  |  |  |  |  |  |  |  |  | exclude |
| [Enterline and Marsh (1982)](#_ENREF_105) | exclude | high risk of bias; reviewer states "no confounders were considered" |  |  |  |  |  |  |  |  |  |  |  |  |  | exclude |
| [Enterline et al. (1987)](#_ENREF_106) | exclude | high risk of bias |  |  |  |  |  |  |  |  |  |  |  |  |  | exclude |
| [Enterline et al. (1995)](#_ENREF_104) | exclude | high risk of bias |  |  |  |  |  |  |  |  |  |  |  |  |  | exclude |
| [Fan et al. (2009)](#_ENREF_108) | consider |  | S | S–LS | LS | LS | S | S–LS | LS (study is not clear what expo-sure was) | S | NA | S | 4 |  |  | Include |
| [Ferreccio et al. (1998)](#_ENREF_119) | exclude | basically the same data & results provided in the 2000 study |  |  |  |  |  |  |  |  |  |  |  |  |  | exclude |
| [Ferreccio et al. (2000)](#_ENREF_118) | consider |  | S | LS | LS (range) | S | S (4+ref) | S | LS | S | S | S | 3 |  |  | include |
| [Ferreccio et al. (2013b)](#_ENREF_121) | consider |  | S | LS (M) | LS (ranges) | S | LS, 2, disjoint | NA | LS | S | S | S | 4 | no, only two exp. groups |  | exclude |
| [García-Esquinas et al. (2013)](#_ENREF_123) | consider |  | LS | S (U) | LS (ranges) | S | LS, 2,  <y, y-z, >z | S | S | LS | NA | S | 4 |  |  | include |
| [Grimsrud et al. (2005)](#_ENREF_133) | consider | note: inhalation study w/poor exp. characterization | S | LS | LS | S | S (3+ref) | S | LS | LS | S | S | 4 |  |  | include |
| [Gunduz et al. (2015)](#_ENREF_142) | exclude | only two deaths, small number of exp. measurements |  |  |  |  |  |  |  |  |  |  |  |  |  | exclude |
| [Guo (2004)](#_ENREF_144) | exclude | ecological |  |  |  |  |  |  |  |  |  |  |  |  |  | exclude |
| [Guo et al. (2004)](#_ENREF_147) | exclude | ecological |  |  |  |  |  |  |  |  |  |  |  |  |  | exclude |
| [Han et al. (2009)](#_ENREF_155) | exclude | ecological |  |  |  |  |  |  |  |  |  |  |  |  |  | exclude |
| [Heck et al. (2009)](#_ENREF_160) | exclude | toenail arsenic |  |  |  |  |  |  |  |  |  |  |  |  |  | exclude |
| [Hinwood et al. (1999)](#_ENREF_162) | exclude | ecological |  |  |  |  |  |  |  |  |  |  |  |  |  | exclude |
| [Hopenhayn-Rich et al. (1998)](#_ENREF_165) | exclude | ecological |  |  |  |  |  |  |  |  |  |  |  |  |  | exclude |
| [Hsu et al. (2013a)](#_ENREF_171) | exclude | large proportion of study pop. had skin lesions; hard to generalize to other pops. |  |  |  |  |  |  |  |  |  |  |  |  |  | exclude |
| [Hu et al. (1999)](#_ENREF_178) | exclude | authors acknowledge likely exp. misclassification |  |  |  |  |  |  |  |  |  |  |  |  |  | exclude |
| [Järup and Pershagen (1991)](#_ENREF_192) | consider |  | LS | LS | LS | S | S | LS (cases reported, but controls were not) | S | S | S | LS | 5 | no, do not have no. people in each exp. stratum |  | exclude |
| [Jarup et al. (1989)](#_ENREF_193) | consider | note: inhalation study w/poor exp. characterization | LS | LS (Inh) | S–LS (7 strata) | LS | S | S | S–LS | S | NA | S | 4 | no, tot. n per exp. stratum not available |  | exclude |
| [Jones et al. (2007)](#_ENREF_197) | consider |  | LS | S–LS | S | LS | S | S | S | S | NA | S | 2.5 | no, do not have number of people in each exp. Stratum |  | exclude |
| [Khlifi et al. (2014)](#_ENREF_203) | exclude | upper respiratory cancer only & only provides blood arsenic levels in cases & controls |  |  |  |  |  |  |  |  |  |  |  |  |  | exclude |
| [Kusiak et al. (1991)](#_ENREF_213) | consider |  | LS | LS | LS (no range, only mean) | LS | S | S | LS | S | NA | S | 5 |  |  | exclude |
| [Kusiak et al. (1993)](#_ENREF_212) | consider |  | LS | LS | LS | LS | S | S | LS | S | NA | S | 5 |  |  | exclude |
| [Lee-Feldstein (1989)](#_ENREF_223) | consider | note: inhalation study w/poor exp. characterization | LS | LS (Inh) | S (6 averages) | LS (age only) | S | LS | S–LS | S | NA | S | 4.5 | no, tot. n per exp. stratum not available |  | exclude |
| [Lewis et al. (1999)](#_ENREF_225) | exclude | too few deaths to support statistical analyses (3, 0, 2, in exp. groups), relatively high exp. uncertainty |  |  |  |  |  |  |  |  |  |  |  |  |  | exclude |
| [Lubin et al. (1981)](#_ENREF_241) | exclude | exp. not adequately quantified |  |  |  |  |  |  |  |  |  |  |  |  |  | exclude |
| [Lubin and Fraumeni (2000)](#_ENREF_239) | exclude | updated by Lubin et. al 2008 for same cohort |  |  |  |  |  |  |  |  |  |  |  |  |  | exclude |
| [Lubin et al. (2008)](#_ENREF_240) | consider | note: updated analysis of same cohort used in Lubin et. al 2000 | LS | S–LS (Inh) | S (6 strata) | S–LS  (no smoking adj.) | S | S | S | S | NA | S | 2 | Referent group is external to study population |  | exclude |
| [Lundstrom et al. (2006)](#_ENREF_242) | consider |  | LS | S–LS | LS (regr.) | S–LS (age not adj., but matched) | LS (regr.) | S | S | S | S | S | 4 | continuous measure of exposure |  | exclude |
| [Luo et al. (2011)](#_ENREF_243) | exclude | only evaluates arsenic as yes, no, or continuous, but there were only 2 counties |  |  |  |  |  |  |  |  |  |  |  |  |  | exclude |
| [Marshall et al. (2007)](#_ENREF_253) | exclude | ecological |  |  |  |  |  |  |  |  |  |  |  |  |  | exclude |
| [Mazumdar et al. (1989)](#_ENREF_254) | consider | note: inhalation study w/poor exp. characterization | LS | LS (Inh) | LS^8^ | LS^9^ | S–LS, 2 | S–LS | S | S | NA | S | 5 | no, tot. n per exp. stratum not available |  | exclude |
| [Mclaughlin et al. (1992)](#_ENREF_259) | consider |  | LS | S–LS | LS | S | S | S | S | S | S | S (tin mine only) | 2.5 | confidence intervals not available |  | exclude |
| [Meliker et al. (2007)](#_ENREF_262) | exclude | ecological |  |  |  |  |  |  |  |  |  |  |  |  |  | exclude |
| [Mikoczy et al. (1996)](#_ENREF_265) | exclude | only 2 exp. groups, referent & >0.5 & very few cases (only 6 tot. kidney cancer cases) |  |  |  |  |  |  |  |  |  |  |  |  |  | exclude |
| [Morales et al. (2000)](#_ENREF_272) | exclude | ecological |  |  |  |  |  |  |  |  |  |  |  |  |  | exclude |
| [Mostafa et al. (2008)](#_ENREF_277) | consider | evaluated smokers & nonsmokers separately only | S | LS | LS | S | S (3+ref) | S | LS | LS | S | S | 4.5 |  |  | include |
| [Nakadaira et al. (2002)](#_ENREF_284) | exclude | exp. only based on residency & presence of skin lesions |  |  |  |  |  |  |  |  |  |  |  |  |  | exclude |
| [Perry et al. (1948)](#_ENREF_301) | exclude | high risk of bias; not really a lung cancer study |  |  |  |  |  |  |  |  |  |  |  |  |  | exclude |
| [Pinto et al. (1978)](#_ENREF_307) | exclude | early smelter study, got (--) on unintended exp. |  |  |  |  |  |  |  |  |  |  |  |  |  | exclude |
| [Qiao et al. (1997)](#_ENREF_310) | consider |  | S | S–LS | LS | S–LS (smoking not adj.) | S | S–LS (provides %) | S | S | NA | S | 2.5 |  |  | include |
| [Rivara et al. (1997)](#_ENREF_330) | exclude | ecological |  |  |  |  |  |  |  |  |  |  |  |  |  | exclude |
| [Sawada et al. (2013)](#_ENREF_335) | consider | note: examine potential uncertainty in dietary intake estimates |  |  |  |  |  |  |  |  |  |  |  |  |  | include |
| [Smith et al. (1998)](#_ENREF_344) | exclude | ecological |  |  |  |  |  |  |  |  |  |  |  |  |  | exclude |
| [Smith et al. (2006)](#_ENREF_346) | exclude | ecological |  |  |  |  |  |  |  |  |  |  |  |  |  | exclude |
| [Smith et al. (2012)](#_ENREF_345) | exclude | ecological |  |  |  |  |  |  |  |  |  |  |  |  |  | exclude |
| [Sorahan (2009)](#_ENREF_350) | exclude | exp. not adequately quantified |  |  |  |  |  |  |  |  |  |  |  |  |  | exclude |
| [Steinmaus et al. (2013)](#_ENREF_356) | consider |  | S | LS (M) | LS (ranges, 4) | S | S | NA | S | S | S | S | 2 |  |  | include |
| [Steinmaus et al. (2014b)](#_ENREF_353) | consider | note: low levels in Chile (all levels <100 µg/L) | S | LS (M) | LS (range, which varied depending on proxy or nonproxy) | S | LS (2+ref) | S | S | S | S | S | 3 |  |  | include |
| [Steinmaus et al. (2014a)](#_ENREF_352) | consider |  | S | LS (M) | LS (ranges, SD) | S | S | NA | S | S | S | S | 2 |  |  | include |
| [Stocks (1960)](#_ENREF_357) | exclude | ecological |  |  |  |  |  |  |  |  |  |  |  |  |  | exclude |
| [Su et al. (2011)](#_ENREF_358) | exclude | ecological |  |  |  |  |  |  |  |  |  |  |  |  |  | exclude |
| ['t Mannetje et al. (2011)](#_ENREF_1) | exclude | exp. not adequately quantified |  |  |  |  |  |  |  |  |  |  |  |  |  | exclude |
| [Taeger et al. (2009)](#_ENREF_360) | consider |  | LS | S–LS | LS | LS | S | S–LS (cases reported, but controls were not) | S | S | NA | S | 4 | prevalence ratio not appropriate for screening level modeling |  | exclude |
| [Taylor et al. (1989)](#_ENREF_361) | exclude | exp. not adequately quantified |  |  |  |  |  |  |  |  |  |  |  |  |  | exclude |
| [Tsai et al. (1999)](#_ENREF_364) | exclude | ecological |  |  |  |  |  |  |  |  |  |  |  |  |  | exclude |
| [Tsuda et al. (1995)](#_ENREF_371) | consider |  | LS | LS | LS (range) | LS | LS (2+ref) | S (observed/ expected) | LS | S | NA | LS (only 9 tot.) | 7 |  |  | exclude |
| [Wadhwa et al. (2011b)](#_ENREF_381) | exclude | no data for exposure-response |  |  |  |  |  |  |  |  |  |  |  |  |  | exclude |
| [Welch et al. (1982)](#_ENREF_391) | consider | note: inhalation study w/poor exp. characterization | LS | LS (Inh) | LS^8^ | S | S | S | S | S | NA | S | 3 | confidence intervals are not available |  | exclude |
| [Wu et al. (1989)](#_ENREF_397) | exclude | ecological |  |  |  |  |  |  |  |  |  |  |  |  |  | exclude |
| [Yang et al. (2013)](#_ENREF_404) | exclude | reanalysis of same data in Chen 2010 |  |  |  |  |  |  |  |  |  |  |  |  |  | exclude |
| [Yorifuji et al. (2011)](#_ENREF_406) | exclude | ecological |  |  |  |  |  |  |  |  |  |  |  |  |  | exclude |
| [Yoshikawa et al. (2008)](#_ENREF_407) | consider |  | LS | LS | LS | LS | S | S | LS | LS | NA | S | 6 |  |  | exclude |

S = suitable; LS = less suitable; NS = not suitable

1. Qualifiers for Exposure Ascertainment:

M = municipal or village water average

KR = kriged average

U = urinary arsenic

toes = toenail arsenic

blood = blood arsenic

Inh = inhalation with no assessment of other routes of exposure

W = work history + estimated mg/m^3^ levels for different jobs and timeframes

2. Qualifiers for Exposure Uncertainty

(ranges) = exposure presented as ranges (numbers indicate numbers of strata)

SD, SE = standard deviation, standard error

3. Does not adjust for gender

4. Results might be affected by blackfoot disease incidence

5. Risks a function of cumulative exposure only for total urinary (not urothelial) cancer

6. Total creatinine-adjusted urinary arsenic reported, but no speciation and no documentation of fish consumption (to determine arsenobetaine and arsenocholine levels are not abnormally high)

7. Number of deaths, but not number of subjects, given for each exposure stratum

8. Averages reported but no measures of dispersion

9. Death rates not adjusted for effect of smoking due to lack of smoking information

#### Skin Cancer

Table S-4. Skin cancer exposure-response study selection

| **Study** | **Initial Screen. Rec.** | **Rationale for Initial Exclusion** | **Endpoint (I, M)** | **Exposure** | | **Est. Adj. (Smoking, Gender, Age)** | **Number** | | **Exposure** | | **Ref. Grp. Represent.** | **Sufficient Cases (Nos.)** | **Mark-downs** | **All Data  Available  for DR?** | **Author Provided Data?** | **Final Rec.** |
| --- | --- | --- | --- | --- | --- | --- | --- | --- | --- | --- | --- | --- | --- | --- | --- | --- |
|  |  |  |  | **Ascertain.** | **Uncertainty** |  | **Exp. Grps.** | **Subj., Cases Rept’d.** | **Metric** | **Timing, Dur.** |  |  |  |  |  |  |
| [Applebaum et al. (2007)](#_ENREF_14) | exclude | exp. surrogate toenails |  |  |  |  |  |  |  |  |  |  |  |  |  | exclude |
| [Baastrup et al. (2008)](#_ENREF_20) | consider | note: large study, but high exp. uncertainty compared to range | S | LS (M) | LS (regr.) | LS | LS (regr.) | LS | S | LS | NA | S | 6 |  |  | exclude |
| [Beane Freeman et al. (2004)](#_ENREF_25) | exclude | exp. surrogate toenails |  |  |  |  |  |  |  |  |  |  |  |  |  | exclude |
| [Bencko et al. (2009)](#_ENREF_26) | exclude | ecological |  |  |  |  |  |  |  |  |  |  |  |  |  | exclude |
| [Besuschio et al. (1980)](#_ENREF_28) | exclude | ecological |  |  |  |  |  |  |  |  |  |  |  |  |  | exclude |
| [Chakraborti et al. (2013)](#_ENREF_45) | exclude | not relevant to skin cancer |  |  |  |  |  |  |  |  |  |  |  |  |  | exclude |
| [Chen and Wang (1990)](#_ENREF_55) | exclude | ecological |  |  |  |  |  |  |  |  |  |  |  |  |  | exclude |
| [Chen et al. (1985)](#_ENREF_54) | exclude | ecological |  |  |  |  |  |  |  |  |  |  |  |  |  | exclude |
| [Chen et al. (1988)](#_ENREF_52) | exclude | ecological |  |  |  |  |  |  |  |  |  |  |  |  |  | exclude |
| [Chen et al. (2003a)](#_ENREF_63) | consider |  | S | LS (M) | LS | S | S | S | S | S | S | S | 2 |  |  | include |
| [Cheng et al. (2015)](#_ENREF_74) | exclude | ecological |  |  |  |  |  |  |  |  |  |  |  |  |  | exclude |
| [Gilbert-Diamond et al. (2013)](#_ENREF_129) | consider |  | S | S | S | S | S | LS | S | S | S | S | 1 | no cases/controls provided per exp. stratum | author provided number of cases | include |
| [Guo et al. (1998)](#_ENREF_146) | exclude | ecological |  |  |  |  |  |  |  |  |  |  |  |  |  | exclude |
| [Guo et al. (2001)](#_ENREF_148) | exclude | ecological |  |  |  |  |  |  |  |  |  |  |  |  |  | exclude |
| [Hinwood et al. (1999)](#_ENREF_162) | exclude | ecological |  |  |  |  |  |  |  |  |  |  |  |  |  | exclude |
| [Hopenhayn-Rich et al. (1998)](#_ENREF_165) | exclude | ecological |  |  |  |  |  |  |  |  |  |  |  |  |  | exclude |
| [Hsu et al. (2013b)](#_ENREF_172) | exclude | study is about diabetes; no usable exp.-response for cancer |  |  |  |  |  |  |  |  |  |  |  |  |  | exclude |
| [Hsu et al. (2015)](#_ENREF_174) | exclude | small number of cases, 20% unknown arsenic levels, Table 2 has data for cum. arsenic, but not adj. for age or sex even though cases & controls were matched |  |  |  |  |  |  |  |  |  |  |  |  |  | exclude |
| [Hsueh et al. (1995)](#_ENREF_176) | exclude | cross-sectional |  |  |  |  |  |  |  |  |  |  |  |  |  | exclude |
| [Hsueh et al. (1997)](#_ENREF_177) | consider |  | S | LS (M) | LS | S | S | S | S | S | NA | S | 2 |  |  | include |
| [Karagas et al. (2001)](#_ENREF_200) | exclude | exp. surrogate toenails |  |  |  |  |  |  |  |  |  |  |  |  |  | exclude |
| [Knobeloch et al. (2006)](#_ENREF_209) | exclude | cross-sectional |  |  |  |  |  |  |  |  |  |  |  |  |  | exclude |
| [Lamm et al. (2007)](#_ENREF_222) | exclude | cross-sectional |  |  |  |  |  |  |  |  |  |  |  |  |  | exclude |
| [Leonardi et al. (2012)](#_ENREF_224) | consider |  | S | LS (M) | S | S | S | LS OR | S | S | S | S | 2 | no cases/controls provided per exp. stratum |  | exclude |
| [Lewis et al. (1999)](#_ENREF_225) | exclude | too few deaths to support statistical analyses (3, 0, 2, in exp. groups), relatively high exp. uncertainty |  |  |  |  |  |  |  |  |  |  |  |  |  | exclude |
| [Morton et al. (1976)](#_ENREF_274) | exclude | ecological |  |  |  |  |  |  |  |  |  |  |  |  |  | exclude |
| [Pesch et al. (2002)](#_ENREF_302) | exclude | exposure = low, medium, or high distance from source; no measurements taken |  |  |  |  |  |  |  |  |  |  |  |  |  | exclude |
| [Philipp et al. (1983)](#_ENREF_304) | exclude | ecological |  |  |  |  |  |  |  |  |  |  |  |  |  | exclude |
| [Rahman et al. (2005b)](#_ENREF_325) | exclude | ecological |  |  |  |  |  |  |  |  |  |  |  |  |  | exclude |
| {Ranft, 2003, 1022505@@author-year} | exclude | focused on urinary As & As exp. only; no response information |  |  |  |  |  |  |  |  |  |  |  |  |  | exclude |
| [Rivara et al. (1997)](#_ENREF_330) | exclude | ecological |  |  |  |  |  |  |  |  |  |  |  |  |  | exclude |
| [Rosales-Castillo et al. (2004)](#_ENREF_331) | exclude | exposure = low or high; levels not given |  |  |  |  |  |  |  |  |  |  |  |  |  | exclude |
| [Smith et al. (1998)](#_ENREF_344) | exclude | ecological |  |  |  |  |  |  |  |  |  |  |  |  |  | exclude |
| [Surdu et al. (2013)](#_ENREF_359) | consider |  | S | LS | LS | S | S | LS | S | S | S | S | 3 | paper covers anemia |  | exclude |
| [Tsai et al. (1999)](#_ENREF_364) | exclude | ecological |  |  |  |  |  |  |  |  |  |  |  |  |  | exclude |
| [Tseng (1977)](#_ENREF_370) | exclude | ecological |  |  |  |  |  |  |  |  |  |  |  |  |  | exclude |
| [Wheeler et al. (2013)](#_ENREF_392) | exclude | ecological |  |  |  |  |  |  |  |  |  |  |  |  |  | exclude |
| [Wu et al. (1989)](#_ENREF_397) | exclude | ecological |  |  |  |  |  |  |  |  |  |  |  |  |  | exclude |

S = suitable; LS = less suitable; NS = not suitable

1. Qualifiers for Exposure Ascertainment:

M = municipal or village water average

KR = kriged average

U = urinary arsenic

toes = toenail arsenic

blood = blood arsenic

Inh = inhalation with no assessment of other routes of exposure

W = work history + estimated mg/m^3^ levels for different jobs and timeframes

2. Qualifiers for Exposure Uncertainty

(ranges) = exposure presented as ranges (numbers indicate numbers of strata)

SD, SE = standard deviation, standard error

3. Does not adjust for gender

4. Results might be affected by blackfoot disease incidence

5. Risks as a function of cumulative exposure only for total urinary cancer (not urothelial carcinoma)

6. Total creatinine-adjusted urinary arsenic reported, but no speciation and no documentation of fish consumption (to determine arsenobetaine and arsenocholine levels are not abnormally high)

7. Number of deaths, but not number of subjects, given for each exposure stratum

8. Averages reported but no measures of dispersion

9. Death rates not adjusted for effect of smoking due to lack of smoking information

#### Skin Lesions

Table S-5. Skin lesions exposure-response study selection

| **Study** | **Initial Screen. Rec.** | **Rationale for Initial Exclusion** | **Endpoint (I, M)** | **Exposure** | | **Est. Adj. (Smoking, Gender, Age)** | **Number** | | **Exposure** | | **Ref. Grp. Represent.** | **Sufficient Cases (Nos.)** | **Mark-downs** | **All Data  Available  for DR?** | **Author Provided Data?** | **Final Rec.** |
| --- | --- | --- | --- | --- | --- | --- | --- | --- | --- | --- | --- | --- | --- | --- | --- | --- |
|  |  |  |  | **Ascertain.** | **Uncertainty** |  | **Exp. Grps.** | **Subj., Cases Rept’d.** | **Metric** | **Timing, Dur.** |  |  |  |  |  |  |
| [Ahamed et al. (2006b)](#_ENREF_5) | exclude | inadequate exp. characterization; categorical response |  |  |  |  |  |  |  |  |  |  |  |  |  | exclude |
| [Ahamed et al. (2006a)](#_ENREF_4) | exclude | inadequate exp.-response characterization |  |  |  |  |  |  |  |  |  |  |  |  |  | exclude |
| [Ahmad et al. (1999)](#_ENREF_8) | exclude | inadequate exp.-response characterization |  |  |  |  |  |  |  |  |  |  |  |  |  | exclude |
| [Ahsan et al. (2000)](#_ENREF_12) | exclude | cross-sectional |  |  |  |  |  |  |  |  |  |  |  |  |  | exclude |
| [Ahsan et al. (2006)](#_ENREF_11) | exclude | cross-sectional |  |  |  |  |  |  |  |  |  |  |  |  |  | exclude |
| [Argos et al. (2007)](#_ENREF_16) | exclude | cross-sectional |  |  |  |  |  |  |  |  |  |  |  |  |  | exclude |
| [Argos et al. (2011)](#_ENREF_15) | consider |  | S mult. skin lesion endpoints | S 5 ranges of well water; urine; cum. exp. estimated | S 5 ranges; cum. exp. estimated | S adj. for age, sex & body mass index, not smoking | S 5 | S adequate N & cases; adj. | S multi-ple; urine, well water; CAI | S cum. exp. esti-mated | NA | S | 0 |  |  | include |
| [Barati et al. (2010)](#_ENREF_21) | exclude | inadequate exp. characterization |  |  |  |  |  |  |  |  |  |  |  |  |  | exclude |
| [Bhowmick et al. (2013)](#_ENREF_30) | exclude | inadequate exp.-response characterization |  |  |  |  |  |  |  |  |  |  |  |  |  | exclude |
| [Biswas et al. (1998)](#_ENREF_31) | exclude | inadequate exp. characterization; co-exp. likely |  |  |  |  |  |  |  |  |  |  |  |  |  | exclude |
| [Borgoño et al. (1977)](#_ENREF_36) | exclude | inadequate exp.-response characterization; hair and nail clippings |  |  |  |  |  |  |  |  |  |  |  |  |  | exclude |
| [Breton et al. (2006)](#_ENREF_39) | exclude | inadequate exp.-response characterization; toenail clippings |  |  |  |  |  |  |  |  |  |  |  |  |  | exclude |
| [Cebrián et al. (1983)](#_ENREF_43) | exclude | ecological |  |  |  |  |  |  |  |  |  |  |  |  |  | exclude |
| [Chakraborti et al. (2003)](#_ENREF_44) | exclude | inadequate exp.-response characterization |  |  |  |  |  |  |  |  |  |  |  |  |  | exclude |
| [Chakraborti et al. (2013)](#_ENREF_45) | exclude | inadequate exp.-response characterization |  |  |  |  |  |  |  |  |  |  |  |  |  | exclude |
| [Chen et al. (2006a)](#_ENREF_67) | exclude | cross-sectional |  |  |  |  |  |  |  |  |  |  |  |  |  | exclude |
| [Chen et al. (2007b)](#_ENREF_70) | consider |  | S | S TWA well-water conc.; urinary arsenic | LS 3 ranges; cum. exp. not estimated | S adj. for age, sex & body mass index, not smoking | LS–S 3 ranges | S adequate N & cases; adj. RR | S urine, TWA well water | LS–S limited exp. history | NA | S | 2 |  |  | include |
| [Dastgiri et al. (2010)](#_ENREF_95) | exclude | ecological |  |  |  |  |  |  |  |  |  |  |  |  |  | exclude |
| [Fatmi et al. (2009)](#_ENREF_114) | exclude | cross-sectional |  |  |  |  |  |  |  |  |  |  |  |  |  | exclude |
| [Fatmi et al. (2013)](#_ENREF_113) | exclude | cross-sectional |  |  |  |  |  |  |  |  |  |  |  |  |  | exclude |
| [García-Vargas et al. (1994)](#_ENREF_124) | exclude | cross-sectional |  |  |  |  |  |  |  |  |  |  |  |  |  | exclude |
| [Ghosh (2013)](#_ENREF_127) | exclude | inadequate exp.-response characterization |  |  |  |  |  |  |  |  |  |  |  |  |  | exclude |
| [Graham et al. (1961)](#_ENREF_131) | exclude | arsenic levels in lesions measured |  |  |  |  |  |  |  |  |  |  |  |  |  | exclude |
| [Guo et al. (2006a)](#_ENREF_150) | exclude | cross-sectional |  |  |  |  |  |  |  |  |  |  |  |  |  | exclude |
| [Guo et al. (2006b)](#_ENREF_151) | consider |  | S | S | LS | S | S | S adj. OR | LS (no cum. exp. esti-mates) | LS–S some history ob-tained, but cum. exp. not esti-mated | S | S | 2.5 |  |  | include |
| [Guo et al. (2007)](#_ENREF_149) | exclude | no data for diabetes stratified by exp. |  |  |  |  |  |  |  |  |  |  |  |  |  | exclude |
| [Hall et al. (2006)](#_ENREF_154) | consider |  | S | S | S (mean also reported) | S | S (4+ref) | S | LS | S | NA | S | 1 |  |  | include |
| [Haque et al. (2003)](#_ENREF_156) | consider |  | S | LS | S (mean also reported) | S | S (4+ref) | S | LS | LS | NA | S | 3 |  |  | include |
| [Hashim et al. (2013)](#_ENREF_157) | exclude | only 2 exp. groups; hair concentration only exp. metric |  |  |  |  |  |  |  |  |  |  |  |  |  | exclude |
| [Hon et al. (2012)](#_ENREF_163) | exclude | inadequate exp.-response information |  |  |  |  |  |  |  |  |  |  |  |  |  | exclude |
| [Hsu et al. (2013a)](#_ENREF_171) | exclude | lack of exp.-response info; reported significant difference between mean arsenic exp. of group w/no skin lesions & group w/skin lesions, but means not reported |  |  |  |  |  |  |  |  |  |  |  |  |  | exclude |
| [Huang et al. (2014)](#_ENREF_182) | exclude | focused on hypertension & diabetes; skin lesions in highest dose group |  |  |  |  |  |  |  |  |  |  |  |  |  | exclude |
| [Lamm et al. (2007)](#_ENREF_222) | exclude | cross-sectional |  |  |  |  |  |  |  |  |  |  |  |  |  | exclude |
| [Li et al. (2013a)](#_ENREF_227) | exclude | exclude. Study focused on hypertension and diabetes. Skin lesions at highest dose group |  |  |  |  |  |  |  |  |  |  |  |  |  | exclude |
| [Lindberg et al. (2008)](#_ENREF_235) | consider |  | S | LS | LS (range) | S | LS (2+ref) | S | S–LS | S–LS | NA | S | 4 |  |  | include |
| [Lindberg et al. (2010)](#_ENREF_236) | consider |  | S | LS | S | S | S | S | S | S | S | S | 1 |  |  | include |
| [Liu et al. (2013)](#_ENREF_238) | exclude | inadequate exp. response information |  |  |  |  |  |  |  |  |  |  |  |  |  | exclude |
| [Maden et al. (2011)](#_ENREF_244) | exclude | cross-sectional |  |  |  |  |  |  |  |  |  |  |  |  |  | exclude |
| [Maharjan et al. (2005)](#_ENREF_246) | exclude | cross-sectional |  |  |  |  |  |  |  |  |  |  |  |  |  | exclude |
| [Maharjan et al. (2007)](#_ENREF_247) | exclude | cross-sectional |  |  |  |  |  |  |  |  |  |  |  |  |  | exclude |
| [Maharjan et al. (2006)](#_ENREF_245) | exclude | ecological |  |  |  |  |  |  |  |  |  |  |  |  |  | exclude |
| [Majumdar et al. (2014)](#_ENREF_249) | exclude | no exposure-response (reversal of skin lesions after arsenic removal) |  |  |  |  |  |  |  |  |  |  |  |  |  | exclude |
| [Guha Mazumder et al. (1998)](#_ENREF_139) | exclude | cross-sectional |  |  |  |  |  |  |  |  |  |  |  |  |  | exclude |
| [Guha Mazumder et al. (2009)](#_ENREF_140) | exclude | skin lesions not evaluated by exp.; key purpose was other health effects in subjects w/o skin lesions |  |  |  |  |  |  |  |  |  |  |  |  |  | exclude |
| [Guha Mazumder et al. (2010)](#_ENREF_137) | exclude | only avg. exp. included for cases & controls |  |  |  |  |  |  |  |  |  |  |  |  |  | exclude |
| [Guha Mazumder et al. (2013)](#_ENREF_136) | exclude | focused on diet & biomarkers |  |  |  |  |  |  |  |  |  |  |  |  |  | exclude |
| [McCarty et al. (2006)](#_ENREF_255) | exclude | not enough exp. information; focus on diet as modifying factor |  |  |  |  |  |  |  |  |  |  |  |  |  | exclude |
| [McDonald et al. (2007)](#_ENREF_258) | consider |  | S | LS | S | LS no smoking | S | S | LS | S | S | S | 3 | no, ORs not adj. |  | exclude |
| [McDonald et al. (2006)](#_ENREF_257) | exclude | ecological |  |  |  |  |  |  |  |  |  |  |  |  |  | exclude |
| [Melkonian et al. (2011)](#_ENREF_263) | consider |  | S | S | LS | S | S | S | S | S | NA | S | 1 |  |  | include |
| [Mitra et al. (2002)](#_ENREF_269) | exclude | cross-sectional |  |  |  |  |  |  |  |  |  |  |  |  |  | exclude |
| [Mosaferi et al. (2008)](#_ENREF_275) | exclude | only avg. exp. incl. for each village |  |  |  |  |  |  |  |  |  |  |  |  |  | exclude |
| [Pavittranon et al. (2003)](#_ENREF_297) | exclude | urine samples; one exp. group |  |  |  |  |  |  |  |  |  |  |  |  |  | exclude |
| [Pei et al. (2013)](#_ENREF_298) | exclude | mechanistic study; exp. groups are level of skin lesions, not arsenic levels |  |  |  |  |  |  |  |  |  |  |  |  |  | exclude |
| [Perry et al. (1948)](#_ENREF_301) | exclude | no skin lesions data |  |  |  |  |  |  |  |  |  |  |  |  |  | exclude |
| [Pesola et al. (2012)](#_ENREF_303) | exclude | cross-sectional |  |  |  |  |  |  |  |  |  |  |  |  |  | exclude |
| [Pierce et al. (2011)](#_ENREF_306) | consider |  | S | LS | S | S | S | S | LS | S | NA | S | 2 |  |  | include |
| [Rahman et al. (2006)](#_ENREF_321) | exclude | Same population data as in [Rahman et al. (2006)](#_ENREF_321) |  |  |  |  |  |  |  |  |  |  |  |  |  | exclude |
| [Rahman et al. (2006)](#_ENREF_321) | consider |  | S | LS | LS | LS no smoking | S | S | S | S | S | S | 3 |  |  | include |
| [Rahman et al. (2003)](#_ENREF_323) | exclude | ecological |  |  |  |  |  |  |  |  |  |  |  |  |  | exclude |
| [Rahman et al. (2005b)](#_ENREF_325) | exclude | ecological |  |  |  |  |  |  |  |  |  |  |  |  |  | exclude |
| [Rahman et al. (2005c)](#_ENREF_326) | exclude | ecological |  |  |  |  |  |  |  |  |  |  |  |  |  | exclude |
| [Rahman et al. (2005a)](#_ENREF_324) | exclude | estimates made on other studies |  |  |  |  |  |  |  |  |  |  |  |  |  | exclude |
| [Saha and Poddar (1986)](#_ENREF_333) | exclude | ecological |  |  |  |  |  |  |  |  |  |  |  |  |  | exclude |
| [Schäfer et al. (1999)](#_ENREF_336) | exclude | atopic eczema study |  |  |  |  |  |  |  |  |  |  |  |  |  | exclude |
| [Seow et al. (2012)](#_ENREF_338) | exclude | baseline reduction on arsenic exp./change in arsenic conc. |  |  |  |  |  |  |  |  |  |  |  |  |  | exclude |
| [Smith et al. (2000)](#_ENREF_343) | exclude | inadequate exp.-response information |  |  |  |  |  |  |  |  |  |  |  |  |  | exclude |
| [Tondel et al. (1999)](#_ENREF_363) | exclude | cross-sectional |  |  |  |  |  |  |  |  |  |  |  |  |  | exclude |
| [Valentine et al. (1991)](#_ENREF_372) | exclude | only 2/36 cases |  |  |  |  |  |  |  |  |  |  |  |  |  | exclude |
| [Valentine et al. (1992)](#_ENREF_373) | exclude | ecological |  |  |  |  |  |  |  |  |  |  |  |  |  | exclude |
| [Xia et al. (2009)](#_ENREF_399) | exclude | cross-sectional |  |  |  |  |  |  |  |  |  |  |  |  |  | exclude |
| [Yu et al. (2007)](#_ENREF_408) | exclude | ecological |  |  |  |  |  |  |  |  |  |  |  |  |  | exclude |

S = suitable; LS = less suitable; NS = not suitable

1. Qualifiers for Exposure Ascertainment:

M = municipal or village water average

KR = kriged average

U = urinary arsenic

toes = toenail arsenic

blood = blood arsenic

Inh = inhalation with no assessment of other routes of exposure

W = work history + estimated mg/m^3^ levels for different jobs and timeframes

2. Qualifiers for Exposure Uncertainty

(ranges) = exposure presented as ranges (numbers indicate numbers of strata)

SD, SE = standard deviation, standard error

3. Does not adjust for gender

4. Results might be affected by blackfoot disease incidence

r of deaths, but not number of subjects, given for each exposure stratum

8. Averages reported but no measures of dispersion

9. Death rates not adjusted for effect of smoking due to lack of smoking information

#### Diabetes

Table S-6. Diabetes exposure-response study selection

| **Study** | **Initial Screen. Rec.** | **Rationale for Initial Exclusion** | **Endpoint (I, M)** | **Exposure** | | **Est. Adj. (Smoking, Gender, Age)** | **Number** | | **Exposure** | | **Ref. Grp. Represent.** | **Sufficient Cases (Nos.)** | **Mark-downs** | **All Data  Available  for DR?** | **Author Provided Data?** | **Final Rec.** |
| --- | --- | --- | --- | --- | --- | --- | --- | --- | --- | --- | --- | --- | --- | --- | --- | --- |
|  |  |  |  | **Ascertain.** | **Uncertainty** |  | **Exp. Grps.** | **Subj., Cases Rept’d.** | **Metric** | **Timing, Dur.** |  |  |  |  |  |  |
| [Bräuner et al. (2014)](#_ENREF_38) | exclude | no (exp. group Ns not provided); incl. in Tier 2? |  |  |  |  |  |  |  |  |  |  |  |  |  | exclude |
| [Chen et al. (2010c)](#_ENREF_65) | exclude | cross-sectional |  |  |  |  |  |  |  |  |  |  |  |  |  | exclude |
| [Chen et al. (2011a)](#_ENREF_58) | exclude | cross-sectional |  |  |  |  |  |  |  |  |  |  |  |  |  | exclude |
| [Chen et al. (2012a)](#_ENREF_59) | exclude | no, endpoint = metabolic syndrome |  |  |  |  |  |  |  |  |  |  |  |  |  | exclude |
| [Chiu et al. (2006)](#_ENREF_85) | exclude | SMRs per yr. in Taiwan, no actual arsenic measurement |  |  |  |  |  |  |  |  |  |  |  |  |  | exclude |
| [Coronado-González et al. (2007)](#_ENREF_90) | consider |  | S | S | LS (ranges) | S | S | S | S  (U-AS) | LS | S | S | 2 |  |  | include |
| [Currier et al. (2014)](#_ENREF_91) | exclude | cross-sectional |  |  |  |  |  |  |  |  |  |  |  |  |  | exclude |
| [Del Razo et al. (2011)](#_ENREF_100) | exclude | linear regr. data presented |  |  |  |  |  |  |  |  |  |  |  |  |  | exclude |
| [D'Ippoliti et al. (2015)](#_ENREF_92) | consider |  | LS (mortal-ity) | LS | LS (range) | S | LS (2+ref) | S | S | S | NA | S | 4 | no data on no. of subjects | Author provided number of subjects per exposure stratum | include |
| [Drobná et al. (2012)](#_ENREF_101) | exclude | focuses on GST polymorphisms |  |  |  |  |  |  |  |  |  |  |  |  |  | exclude |
| [Ettinger et al. (2009)](#_ENREF_107) | exclude | address in Tier 2 (sensitive pop.); authors’ exposure-response = good |  |  |  |  |  |  |  |  |  |  |  |  |  | exclude |
| [Feng et al. (2015)](#_ENREF_116) | exclude | cross-sectional |  |  |  |  |  |  |  |  |  |  |  |  |  | exclude |
| [Feseke et al. (2015)](#_ENREF_122) | exclude | cross-sectional |  |  |  |  |  |  |  |  |  |  |  |  |  | exclude |
| [Gribble et al. (2012)](#_ENREF_132) | exclude | cross-sectional |  |  |  |  |  |  |  |  |  |  |  |  |  | exclude |
| [Guo et al. (2007)](#_ENREF_149) | exclude | no data for diabetes stratified by exp. |  |  |  |  |  |  |  |  |  |  |  |  |  | exclude |
| [Hsu et al. (2013b)](#_ENREF_172) | exclude | data presented as internal cancer incidence by diabetes status |  |  |  |  |  |  |  |  |  |  |  |  |  | exclude |
| [Huang et al. (2014)](#_ENREF_182) | exclude | only 2 dose groups (> or <907.25 µg/L in DW) |  |  |  |  |  |  |  |  |  |  |  |  |  | exclude |
| [Islam et al. (2012b)](#_ENREF_189) | exclude | cross-sectional |  |  |  |  |  |  |  |  |  |  |  |  |  | exclude |
| [James et al. (2013)](#_ENREF_191) | consider |  | S | LS | LS | S | S | LS | S (TWA water As) | S | S | S | 3 |  |  | include |
| [Jensen and Hansen (1998)](#_ENREF_194) | exclude | no data for diabetes stratified by exp.; measured glycosylated hemoglobin, occupational exp. |  |  |  |  |  |  |  |  |  |  |  |  |  | exclude |
| [Jovanovic et al. (2013)](#_ENREF_198) | exclude | no data for diabetes stratified by exp. |  |  |  |  |  |  |  |  |  |  |  |  |  | exclude |
| [Kim and Lee (2011)](#_ENREF_207) | exclude | no data for diabetes stratified by exp.; regr. analysis |  |  |  |  |  |  |  |  |  |  |  |  |  | exclude |
| [Kim et al. (2013)](#_ENREF_206) | exclude | no; maybe extract data in Tier 2 |  |  |  |  |  |  |  |  |  |  |  |  |  | exclude |
| [Lai et al. (1994)](#_ENREF_218) | exclude | cross-sectional |  |  |  |  |  |  |  |  |  |  |  |  |  | exclude |
| [Lewis et al. (1999)](#_ENREF_225) | exclude | no, SMRs w/o Ns |  |  |  |  |  |  |  |  |  |  |  |  |  | exclude |
| [Li et al. (2013a)](#_ENREF_227) | exclude | cross-sectional |  |  |  |  |  |  |  |  |  |  |  |  |  | exclude |
| [Lin et al. (2014)](#_ENREF_234) | exclude | linear regr. data presented |  |  |  |  |  |  |  |  |  |  |  |  |  | exclude |
| [Maiti et al. (2012)](#_ENREF_248) | exclude | diabetes status not reported |  |  |  |  |  |  |  |  |  |  |  |  |  | exclude |
| [Makris et al. (2012)](#_ENREF_251) | exclude | exp. analyzed as each quintile relative to 1st quintile; individual exp. level values not given |  |  |  |  |  |  |  |  |  |  |  |  |  | exclude |
| [Meliker et al. (2007)](#_ENREF_262) | exclude | no data for diabetes stratified by exp.; SMRs for males, females |  |  |  |  |  |  |  |  |  |  |  |  |  | exclude |
| [Nabi et al. (2005)](#_ENREF_281) | exclude | no data for exposure-response |  |  |  |  |  |  |  |  |  |  |  |  |  | exclude |
| [Navas-Acien et al. (2008)](#_ENREF_285) | exclude | same data as Steinmaus (2009) |  |  |  |  |  |  |  |  |  |  |  |  |  | exclude |
| [Navas-Acien et al. (2009)](#_ENREF_286) | exclude | no data by exp.; present ORs for 80th- vs. 20th-%tile urine arsenic distribution |  |  |  |  |  |  |  |  |  |  |  |  |  | exclude |
| [Nizam et al. (2013)](#_ENREF_287) | exclude | no data stratified by exp. for diabetes; compares urinary As in diabetic vs. nondiabetic subjects |  |  |  |  |  |  |  |  |  |  |  |  |  | exclude |
| [Pan et al. (2013)](#_ENREF_292) | consider |  | S | LS | LS | S | S | S | LS (water) | LS | NA | S | 4 | no. of controls per exp. stratum not provided |  | exclude |
| [Peng et al. (2015a)](#_ENREF_299) | exclude | cross-sectional |  |  |  |  |  |  |  |  |  |  |  |  |  | exclude |
| [Peng et al. (2015b)](#_ENREF_300) | consider |  | S | S | LS (ranges are not always reported, overall mean & IQR) | S | S (3+ref) | S | LS (meco-nium) | NS  (4 wks. after out-come) | S | S | 4 | no; no mean or range for exp. |  | exclude |
| [Rahman and Axelson (1995)](#_ENREF_315) | exclude | exp. assessed relative to occupational standard by safety engineer |  |  |  |  |  |  |  |  |  |  |  |  |  | exclude |
| [Rahman and Axelson (2001)](#_ENREF_316) | exclude | no data stratified by exp.; measured glucosuria |  |  |  |  |  |  |  |  |  |  |  |  |  | exclude |
| [Rahman et al. (1996)](#_ENREF_322) | exclude | no data by exp. |  |  |  |  |  |  |  |  |  |  |  |  |  | exclude |
| [Rahman et al. (1998)](#_ENREF_318) | consider |  | S | LS | LS (0.5, 0.5–1.0, >1.0 mg/L) | S | S | S | LS (water) | LS | NA | S | 4 |  |  | Include |
| [Rahman et al. (1999b)](#_ENREF_320) | exclude | Tier 2; endpoint = glycosuria; sensitive pops. (skin lesions) |  |  |  |  |  |  |  |  |  |  |  |  |  | exclude |
| [Rhee et al. (2013)](#_ENREF_329) | exclude | no. subjects for each exp. level not provided |  |  |  |  |  |  |  |  |  |  |  |  |  | exclude |
| [Shapiro et al. (2015)](#_ENREF_340) | exclude | gestational diabetes, arsenic measured in blood, only tot. no. cases & controls, assume quartiles would have even nos., but not all cases even nos. |  |  |  |  |  |  |  |  |  |  |  |  |  | exclude |
| [Steinmaus et al. (2009)](#_ENREF_355) | exclude | cross-sectional |  |  |  |  |  |  |  |  |  |  |  |  |  | exclude |
| [Tsai et al. (1999)](#_ENREF_364) | exclude | no data by exp. |  |  |  |  |  |  |  |  |  |  |  |  |  | exclude |
| [Tseng et al. (2000)](#_ENREF_369) | exclude | only 2 dose groups (> or <17 mg/L-yr cum. arsenic exp.) |  |  |  |  |  |  |  |  |  |  |  |  |  | exclude |
| [Wang et al. (2003)](#_ENREF_386) | exclude | ecological |  |  |  |  |  |  |  |  |  |  |  |  |  | exclude |
| [Zierold et al. (2004)](#_ENREF_412) | exclude | report ORs in 2 groups (2 µg/L As <10 µg/L, >10 µg/L) |  |  |  |  |  |  |  |  |  |  |  |  |  | exclude |

S = suitable; LS = less suitable; NS = not suitable

1. Qualifiers for Exposure Ascertainment:

M = municipal or village water average

KR = kriged average

U = urinary arsenic

toes = toenail arsenic

blood = blood arsenic

Inh = inhalation with no assessment of other routes of exposure

W = work history + estimated mg/m^3^ levels for different jobs and timeframes

2. Qualifiers for Exposure Uncertainty

(ranges) = exposure presented as ranges (numbers indicate numbers of strata)

SD, SE = standard deviation, standard error

3. Does not adjust for gender

4. Results might be affected by blackfoot disease incidence

5. Risks as a function of cumulative exposure only for total urinary cancer (not urothelial carcinoma)

6. Total creatinine-adjusted urinary arsenic reported, but no speciation and no documentation of fish consumption (to determine arsenobetaine and arsenocholine levels are not abnormally high)

7. Number of deaths, but not number of subjects, given for each exposure stratum

8. Averages reported but no measures of dispersion

9. Death rates not adjusted for effect of smoking due to lack of smoking information

#### Immune System Impairment

Table S-7. Immune system impairment exposure-response study selection

| **Study** | **Initial Screen. Rec.** | **Rationale for Initial Exclusion** | **Endpoint (I, M)** | **Exposure** | | **Est. Adj. (Smoking, Gender, Age)** | **Number** | | **Exposure** | | **Ref. Grp. Represent.** | **Sufficient Cases (Nos.)** | **Mark-downs** | **All Data  Available  for DR?** | **Author Provided Data?** | **Final Rec.** |
| --- | --- | --- | --- | --- | --- | --- | --- | --- | --- | --- | --- | --- | --- | --- | --- | --- |
|  |  |  |  | **Ascertain.** | **Uncertainty** |  | **Exp. Grps.** | **Subj., Cases Rept’d.** | **Metric** | **Timing, Dur.** |  |  |  |  |  |  |
| [Ahmed et al. (2012)](#_ENREF_9) | consider | will need raw data for exposure-response | S | S (U, summed organic & inorganic, specific gravity adj.) | LS (regr.) | S | LS (regr.; U at GW has 2 exp. group breaks) | S | S (U) | S (U at DW 8 & 30) | NA | S | 2 | no; only regr. coeffs. |  | exclude |
| [Ahmed et al. (2014)](#_ENREF_10) | consider |  | S | S (U, summed organic & inorganic, specific gravity adj., seafood consump-tion minimal) | S (range, median) | S^3^ | S | S | S | S | S | S | 0 | no; no. cases not reported |  | exclude |
| [Biswas et al. (2008)](#_ENREF_32) | exclude | no data for exposure-response |  |  |  |  |  |  |  |  |  |  |  |  |  | exclude |
| [Das et al. (2012)](#_ENREF_94) | consider |  | LS^4^ | S (U)^6^ | LS (range) | LS^5^ | S | S | S | LS | LS^7^ | S | 5 |  |  | exclude |
| [Farzan et al. (2015c)](#_ENREF_112) | consider | need raw data for exposure-response | S | LS | LS (per doubling) | S | LS (per doubling) | S | S | S | NA | S | 3 | no, relative risk per doubling of maternal urine |  | exclude |
| [Farzan et al. (2013)](#_ENREF_111) | consider | same data reviewed for nonmalignant respiratory effects; would need raw data for exposure-response | S | S (U, summed inorganic & metab-olites) | LS (regr.) | S | LS (regr.) | S | S (U) | S (mater-nal U, effects in 4-mo. olds) | NA | S | 2 | no; only regr. coeffs. |  | exclude |
| [Heaney et al. (2015)](#_ENREF_159) | exclude | no data for dose- response |  |  |  |  |  |  |  |  |  |  |  |  |  | exclude |
| [Islam et al. (2007)](#_ENREF_187) | exclude | no data for exposure-response |  |  |  |  |  |  |  |  |  |  |  |  |  | exclude |
| [Kile et al. (2014a)](#_ENREF_204) | consider | need raw data for exposure-response | LS  (T-cell compos-ition) | LS (water from primary well) | LS (regr.) | LS (only infant sex) | LS (regr.) | LS (tot. 44 subjects) | LS (mater-nal log water arsenic conc.) | S | NA | LS (44 subjects) | 8 |  |  | exclude |
| [Kile et al. (2014b)](#_ENREF_205) | exclude | high risk for bias, subjects self-reported, many aware of exp. Status |  |  |  |  |  |  |  |  |  |  |  |  |  | exclude |
| [Moore et al. (2009)](#_ENREF_271) | consider | need raw data for exposure-response | S | S (U, summed organic & inorganic, specific gravity adj.) | LS (regr.) | S^3^ | LS (regr.) | S (tot. subjects reported) | LS | S | NA | S (1,556 subjects) | 3 | need raw data for exposure-response |  | exclude |
| [Nadeau et al. (2014)](#_ENREF_282) | consider | need raw data for exposure-response | S | S | LS  (per  1-µg/L increase) | S | LS  (per 1‑µg/L increase) | S (n used in regr. provided) | S | S | NA | LS (S for some outcomes) | 3 | need raw data for exposure-response |  | exclude |
| [Rahman et al. (2011)](#_ENREF_312) | consider |  | LS | S (U, summed inorganic & metab-olites) | LS (range) | LS (women stated not to smoke, but paternal smoking not addressed) | S | LS (reported as no. episodes & no. wks. of recall) | S (U) | S (mater-nal U) | NA | S (no. episodes) | 4 | number of subjects not provided |  | exclude |
| [Raqib et al. (2009)](#_ENREF_327) | consider | (study addressed & considered for nonmalignant respiratory, considered no usable data) | S (2-wk. recall) | S (U, summed inorganic & metab-olites) | LS (regr.) | LS | LS (regr.) | LS | S (U) | S (mater-nal urine) | NA | LS | 5 |  |  | exclude |
| [Saha et al. (2013)](#_ENREF_332) | exclude | no data for dose- response |  |  |  |  |  |  |  |  |  |  |  |  |  | exclude |
| [Ser et al. (2014)](#_ENREF_339) | consider | need raw data for exposure-response | LS (IgG) | S (U, tot. specific gravity adj.) | LS (regr.) | S | LS (regr.) | S (tot. subjects reported) | S (U) | S (mater-nal urine; timing LS (mater-nal IgG) | NA | S | 3 | need raw data for exposure-response |  | exclude |
| [Shiue (2013)](#_ENREF_341) | exclude | cross-sectional |  |  |  |  |  |  |  |  |  |  |  |  |  | exclude |
| [Smith et al. (2011)](#_ENREF_347) | exclude | exposure based on region |  |  |  |  |  |  |  |  |  |  |  |  |  | exclude |
| [Sohel et al. (2009)](#_ENREF_349) | consider |  | LS (mortal-ity) | S | LS (range) | S^3^ | S | LS | LS^8^ | S | NA | S | 4 | no, no. subjects per exp. group not provided |  | exclude |
| [Soto-Peña et al. (2006)](#_ENREF_351) | exclude | cross-sectional |  |  |  |  |  |  |  |  |  |  |  |  |  | exclude |

S = suitable; LS = less suitable; NS = not suitable

1. Qualifiers for Exposure Ascertainment:

M = municipal or village water average

KR = kriged average

U = urinary arsenic

toes = toenail arsenic

blood = blood arsenic

Inh = inhalation with no assessment of other routes of exposure

W = work history + estimated mg/m^3^ levels for different jobs and timeframes

2. Qualifiers for Exposure Uncertainty

(ranges) = exposure presented as ranges (numbers indicate numbers of strata)

SD, SE = standard deviation, standard error

3. Does not adjust for gender

4. Results might be affected by blackfoot disease incidence

5. Risks as a function of cumulative exposure only for total urinary cancer (not urothelial carcinoma)

6. Total creatinine-adjusted urinary arsenic reported, but no speciation and no documentation of fish consumption (to determine arsenobetaine and arsenocholine levels are not abnormally high)

7. Number of deaths, but not number of subjects, given for each exposure stratum

8. Averages reported but no measures of dispersion

9. Death rates not adjusted for effect of smoking due to lack of smoking information

#### Liver Cancer

Table S-8. Liver cancer exposure-response study selection

| **Study** | **Initial Screen. Rec.** | **Rationale for Initial Exclusion** | **Endpoint (I, M)** | **Exposure** | | **Est. Adj. (Smoking, Gender, Age)** | **Number** | | **Exposure** | | **Ref. Grp. Represent.** | **Sufficient Cases (Nos.)** | **Mark-downs** | **All Data  Available  for DR?** | **Author Provided Data?** | **Final Rec.** |
| --- | --- | --- | --- | --- | --- | --- | --- | --- | --- | --- | --- | --- | --- | --- | --- | --- |
|  |  |  |  | **Ascertain.** | **Uncertainty** |  | **Exp. Grps.** | **Subj., Cases Rept’d.** | **Metric** | **Timing, Dur.** |  |  |  |  |  |  |
| [Baastrup et al. (2008)](#_ENREF_20) | exclude | large study but high exp. uncertainty; only regr. results presented for all health outcomes |  |  |  |  |  |  |  |  |  |  |  |  |  | exclude |
| [Besuschio et al. (1980)](#_ENREF_28) | exclude | ecological |  |  |  |  |  |  |  |  |  |  |  |  |  | exclude |
| [Chen and Wang (1990)](#_ENREF_55) | exclude | ecological |  |  |  |  |  |  |  |  |  |  |  |  |  | exclude |
| [Chen et al. (1986)](#_ENREF_50) | exclude | exp. was only yrs. of use so not useful |  |  |  |  |  |  |  |  |  |  |  |  |  | exclude |
| [Chen et al. (1985)](#_ENREF_54) | exclude | ecological |  |  |  |  |  |  |  |  |  |  |  |  |  | exclude |
| [Chen et al. (1988)](#_ENREF_52) | exclude | ecological |  |  |  |  |  |  |  |  |  |  |  |  |  | exclude |
| [Chen et al. (1992)](#_ENREF_48) | exclude | ecological |  |  |  |  |  |  |  |  |  |  |  |  |  | exclude |
| [Chen et al. (2014)](#_ENREF_60) | exclude | relative risk for cancer mortality based on 1 ppm increase in soil As |  |  |  |  |  |  |  |  |  |  |  |  |  | exclude |
| {Chung, 2012, 1453800@@author-year} | exclude | no. subjects per exp. stratum not provided |  |  |  |  |  |  |  |  |  |  |  |  |  | exclude |
| [D'Ippoliti et al. (2015)](#_ENREF_92) | consider | no data on no. subjects per exp. stratum; requested information from study authors | LS (mortal-ity) | LS | LS (range) | S | LS  (2+ref) | S | S | S | NA | S | 4 | number of subjects not provided | author provided number of subjects per exposure stratum | include |
| [García-Esquinas et al. (2013)](#_ENREF_123) | consider |  | LS | S (U) | LS (ranges) | S | LS  (2 + ref) | S | S | LS | NA | S | 4 |  |  | include |
| [Gunduz et al. (2015)](#_ENREF_142) | exclude | only 2 deaths |  |  |  |  |  |  |  |  |  |  |  |  |  | exclude |
| [Guo (2003)](#_ENREF_143) | exclude | ecological |  |  |  |  |  |  |  |  |  |  |  |  |  | exclude |
| [Han et al. (2009)](#_ENREF_155) | exclude | ecological |  |  |  |  |  |  |  |  |  |  |  |  |  | exclude |
| [Hinwood et al. (1999)](#_ENREF_162) | exclude | ecological |  |  |  |  |  |  |  |  |  |  |  |  |  | exclude |
| [Hopenhayn-Rich et al. (1998)](#_ENREF_165) | exclude | ecological |  |  |  |  |  |  |  |  |  |  |  |  |  | exclude |
| [Lewis et al. (1999)](#_ENREF_225) | exclude | exclude, very few deaths & deaths not in all exp. categories |  |  |  |  |  |  |  |  |  |  |  |  |  | exclude |
| [Liaw et al. (2008)](#_ENREF_232) | exclude | ecological |  |  |  |  |  |  |  |  |  |  |  |  |  | exclude |
| [Lin et al. (2013)](#_ENREF_233) | exclude | ecological |  |  |  |  |  |  |  |  |  |  |  |  |  | exclude |
| [Meliker et al. (2007)](#_ENREF_262) | exclude | ecological |  |  |  |  |  |  |  |  |  |  |  |  |  | exclude |
| [Morales et al. (2000)](#_ENREF_272) | exclude | ecological |  |  |  |  |  |  |  |  |  |  |  |  |  | exclude |
| [Rivara et al. (1997)](#_ENREF_330) | exclude | ecological |  |  |  |  |  |  |  |  |  |  |  |  |  | exclude |
| [Sawada et al. (2013)](#_ENREF_335) | consider |  | S | LS | S | S | S | S | S | LS (recent only) | NA | S | 2 |  |  | include |
| [Smith et al. (2012)](#_ENREF_345) | exclude | ecological |  |  |  |  |  |  |  |  |  |  |  |  |  | exclude |
| [Smith et al. (1998)](#_ENREF_344) | exclude | ecological |  |  |  |  |  |  |  |  |  |  |  |  |  | exclude |
| [Tsai et al. (1999)](#_ENREF_364) | exclude | ecological |  |  |  |  |  |  |  |  |  |  |  |  |  | exclude |
| [Tsuda et al. (1995)](#_ENREF_371) | exclude | only 2 cancers observed (in high dose) |  |  |  |  |  |  |  |  |  |  |  |  |  | exclude |
| [Wadhwa et al. (2011a)](#_ENREF_380) | exclude | no data for exposure-response, only exposed vs. unexposed |  |  |  |  |  |  |  |  |  |  |  |  |  | exclude |
| [Wu et al. (1989)](#_ENREF_397) | exclude | ecological |  |  |  |  |  |  |  |  |  |  |  |  |  | exclude |
| [Yorifuji et al. (2011)](#_ENREF_406) | exclude | ecological |  |  |  |  |  |  |  |  |  |  |  |  |  | exclude |

S = suitable; LS = less suitable; NS = not suitable

1. Qualifiers for Exposure Ascertainment:

M = municipal or village water average

KR = kriged average

U = urinary arsenic

toes = toenail arsenic

blood = blood arsenic

Inh = inhalation with no assessment of other routes of exposure

W = work history + estimated mg/m^3^ levels for different jobs and timeframes

2. Qualifiers for Exposure Uncertainty

(ranges) = exposure presented as ranges (numbers indicate numbers of strata)

SD, SE = standard deviation, standard error

3. Does not adjust for gender

4. Results might be affected by blackfoot disease incidence

5. Risks as a function of cumulative exposure only for total urinary cancer (not urothelial carcinoma)

6. Total creatinine-adjusted urinary arsenic reported, but no speciation and no documentation of fish consumption (to determine arsenobetaine and arsenocholine levels are not abnormally high)

7. Number of deaths, but not number of subjects, given for each exposure stratum

8. Averages reported but no measures of dispersion

9. Death rates not adjusted for effect of smoking due to lack of smoking information

#### Nonmalignant Respiratory Disease

Table S-9. Nonmalignant respiratory disease exposure-response study selection

| **Study** | **Initial Screen. Rec.** | **Rationale for Initial Exclusion** | **Endpoint (I, M)** | **Exposure** | | **Est. Adj. (Smoking, Gender, Age)** | **Number** | | **Exposure** | | **Ref. Grp. Represent.** | **Sufficient Cases (Nos.)** | **Mark-downs** | **All Data  Available  for DR?** | **Author Provided Data?** | **Final Rec.** |
| --- | --- | --- | --- | --- | --- | --- | --- | --- | --- | --- | --- | --- | --- | --- | --- | --- |
|  |  |  |  | **Ascertain.** | **Uncertainty** |  | **Exp. Grps.** | **Subj., Cases Rept’d.** | **Metric** | **Timing, Dur.** |  |  |  |  |  |  |
| [Argos et al. (2014)](#_ENREF_17) | consider | large study but mortality endpoint | LS | S (U)^6^ | LS (range) | S | LS (2) | S | S (U) | LS | NA | S | 4 | subjects per exp. stratum not provided; approximated |  | include |
| [Bhattacharyya et al. (2014)](#_ENREF_29) | exclude | cross-sectional |  |  |  |  |  |  |  |  |  |  |  |  |  | exclude |
| [Chakraborti et al. (2013)](#_ENREF_45) | exclude | survey, effects reported in subjects w/skin lesion, no data for exposure-response |  |  |  |  |  |  |  |  |  |  |  |  |  | exclude |
| [Chattopadhyay et al. (2010)](#_ENREF_47) | exclude | approx. 20 subjects per exp. group & high potential for exp. misclassification |  |  |  |  |  |  |  |  |  |  |  |  |  | exclude |
| [Das et al. (2014)](#_ENREF_93) | exclude | no data for exposure-response |  |  |  |  |  |  |  |  |  |  |  |  |  | exclude |
| [Dauphiné et al. (2011)](#_ENREF_96) | consider | need raw data, but breakdown in paper is 0–250 µg/L compared to >800 µg/L before age 10 yrs. | S | LS (M) | LS (range) | S | LS (only 2 groups with 0–250 µg/L being the reference) | S | LS | LS (report peak exp. before 10, but not exp. duration | NA | S | 5 |  |  | exclude |
| [De et al. (2004)](#_ENREF_99) | exclude | lung function only measured in cases w/arsenicosis, controls apparently not incl., hair & nail measurements available for few subjects |  |  |  |  |  |  |  |  |  |  |  |  |  | exclude |
| [D'Ippoliti et al. (2015)](#_ENREF_92) | consider | no data on no. subjects per exp. stratum; requested information from study authors | LS (mortal-ity) | LS | LS (range) | S | LS (2+ref) | S | S | S | NA | S | 4 | number of subjects not reported | author provided number of subjects per exposure stratum | include |
| [Engel and Smith (1994)](#_ENREF_103) | exclude | high risk of exp. bias, likely differences between groups not accounted for, as subjects were from different areas |  |  |  |  |  |  |  |  |  |  |  |  |  | exclude |
| [Farzan et al. (2015c)](#_ENREF_112) | consider | need raw data for exposure-response | S | S | LS (per doubling) | S | LS (per doubling) | S | S | S | NA | S | 2 | no, would need raw data |  | exclude |
| [Farzan et al. (2013)](#_ENREF_111) | consider | need raw data for exposure-response | S | S (U, summed inorganic & metab-olites) | LS (regr.) | S | LS (regr.) | S | S (U) | S (mater-nal U, effects in 4- mo. olds) | NA | S | 2 | no, primarily regr. results |  | exclude |
| [Ghosh et al. (2007)](#_ENREF_128) | exclude | no data for dose- response |  |  |  |  |  |  |  |  |  |  |  |  |  | exclude |
| [Ghosh (2013)](#_ENREF_127) | exclude | evaluated respiratory effects in cases & controls w/skin lesions; no data for exposure-response |  |  |  |  |  |  |  |  |  |  |  |  |  | exclude |
| [Guo et al. (2007)](#_ENREF_149) | exclude | no data for exposure-response |  |  |  |  |  |  |  |  |  |  |  |  |  | exclude |
| [Halatek et al. (2014)](#_ENREF_152) | exclude | no data for exposure-response |  |  |  |  |  |  |  |  |  |  |  |  |  | exclude |
| [Halatek et al. (2009)](#_ENREF_153) | exclude | no data for dose- response |  |  |  |  |  |  |  |  |  |  |  |  |  | exclude |
| [Hopenhayn-Rich et al. (1996)](#_ENREF_164) | exclude | only low, medium, & high, not well defined, w/high risk of bias |  |  |  |  |  |  |  |  |  |  |  |  |  | exclude |
| [Kile et al. (2014b)](#_ENREF_205) | exclude | high risk of bias, subjects self-reported, many aware of exp. status |  |  |  |  |  |  |  |  |  |  |  |  |  | exclude |
| [Lewis et al. (1999)](#_ENREF_225) | exclude | decrease in effect, which might be related to low smoking, but cannot account for smoking |  |  |  |  |  |  |  |  |  |  |  |  |  | exclude |
| [Lubin et al. (1981)](#_ENREF_241) | exclude | exp. not adequately quantified |  |  |  |  |  |  |  |  |  |  |  |  |  | exclude |
| [Majumdar et al. (2009)](#_ENREF_250) | exclude | cross-sectional |  |  |  |  |  |  |  |  |  |  |  |  |  | exclude |
| [Guha Mazumder et al. (2000)](#_ENREF_138) | exclude | only effects evaluated were symptoms |  |  |  |  |  |  |  |  |  |  |  |  |  | exclude |
| [Guha Mazumder et al. (2005)](#_ENREF_141) | exclude | survey, nonmalignant respiratory effects reported in subjects w/skin lesions, no data for exposure-response |  |  |  |  |  |  |  |  |  |  |  |  |  | exclude |
| [Meliker et al. (2007)](#_ENREF_262) | exclude | no data for dose- response |  |  |  |  |  |  |  |  |  |  |  |  |  | exclude |
| [Milton et al. (2001)](#_ENREF_266) | exclude | survey, nonmalignant respiratory effects reported in subjects w/skin lesions, no data for exposure-response |  |  |  |  |  |  |  |  |  |  |  |  |  | exclude |
| [Milton and Rahman (2002)](#_ENREF_267) | exclude | only 44 subjects w/skin lesions considered exposed & distributed to 3 categories, lowest category <600 µg/L |  |  |  |  |  |  |  |  |  |  |  |  |  | exclude |
| [Nafees et al. (2011)](#_ENREF_283) | exclude | exp. only presented as >100 or >250 µg/L; no data for exposure-response |  |  |  |  |  |  |  |  |  |  |  |  |  | exclude |
| [Olivas-Calderon et al. (2015)](#_ENREF_289) | exclude | no adjustments; examines only percentage w/lung impairment | S | S | S | NS (not adj.) | S (3+ref) | LS (tot. in group & % reported, so no. could be calculated) | S | LS | NA | LS for obstructive, but okay for restrictive | 5 |  |  | exclude |
| [Parvez et al. (2008)](#_ENREF_293) | exclude | cross-sectional |  |  |  |  |  |  |  |  |  |  |  |  |  | exclude |
| [Parvez et al. (2010)](#_ENREF_294) | exclude | only effects evaluated were symptoms |  |  |  |  |  |  |  |  |  |  |  | no, no cases or controls; only beta coeffs. |  | exclude |
| [Parvez et al. (2013)](#_ENREF_295) | consider |  | S | S | LS (range) | S | LS (2) | S | S (U); LS (water) | S | NA | S | 2 | no, no cases or controls; only beta coeffs. |  | exclude |
| [Paul et al. (2013)](#_ENREF_296) | exclude | no data for exposure-response |  |  |  |  |  |  |  |  |  |  |  |  |  | exclude |
| [Perry et al. (1948)](#_ENREF_301) | exclude | no data for exposure-response |  |  |  |  |  |  |  |  |  |  |  |  |  | exclude |
| [Pesola et al. (2012)](#_ENREF_303) | exclude | cross-sectional |  |  |  |  |  |  |  |  |  |  |  |  |  | exclude |
| [Rahman et al. (2011)](#_ENREF_312) | consider |  | S (7-da morbidity recall) | S (U, summed inorganic & metab-olites) | LS (range) | LS (women stated not to smoke, but paternal smoking not addressed) | S | LS (reported as no. episodes & no. wks. of recall) | S (U) | S (maternal U) | NA | S (no. episodes) | 3 | data not provided in format for exposure-response modeling |  | exclude |
| [Raqib et al. (2009)](#_ENREF_327) | consider |  | S (2-wk. recall) | S (U, summed inorganic & metab-olites) | LS (regr.) | LS | LS (regr.) | LS | S (U) | S (matern-al urine) | NA | LS | 5 |  |  | exclude |
| [Recio-Vega et al. (2015)](#_ENREF_328) | exclude | no adjustments; but Table 4 provides spirometry results (possibly for Tier 2 if raw data available for adjustments); possibly use gender information in characteristic table because results were for children |  |  |  |  |  |  |  |  |  |  |  |  |  | exclude |
| [Smith et al. (1998)](#_ENREF_344) | exclude | no data for exposure-response |  |  |  |  |  |  |  |  |  |  |  |  |  | exclude |
| [Smith et al. (2006)](#_ENREF_346) | exclude | no data for exposure-response |  |  |  |  |  |  |  |  |  |  |  |  |  | exclude |
| [Smith et al. (2011)](#_ENREF_347) | exclude | no data for exposure-response |  |  |  |  |  |  |  |  |  |  |  |  |  | exclude |
| [Smith et al. (2012)](#_ENREF_345) | exclude | no data for exposure-response |  |  |  |  |  |  |  |  |  |  |  |  |  | exclude |
| [Smith et al. (2013)](#_ENREF_348) | consider | consider for asthma, based on a specific symptoms question-naire; possibly for lung function (no significant effect), but likely would need raw data | S | S | LS (range) | S | S  (asthma, 4); LS (lung function, 2) | LS | LS | S | NA | LS (no. not specified) | 4 | no cases & controls data |  | exclude |
| [Stocks (1960)](#_ENREF_357) | exclude | no data for exposure-response |  |  |  |  |  |  |  |  |  |  |  |  |  | exclude |
| [Tsai et al. (1999)](#_ENREF_364) | exclude | no data for exposure-response |  |  |  |  |  |  |  |  |  |  |  |  |  | exclude |
| [von Ehrenstein et al. (2005)](#_ENREF_377) | exclude | exp. based only on presence/absence of skin lesions |  |  |  |  |  |  |  |  |  |  |  |  |  | exclude |
| [Welch et al. (1982)](#_ENREF_391) | consider | inhalation study w/poor exp. characterization | LS | LS (W) | LS (range) | S | S (3) | S | LS (had TWA & ceiling) | S | NA | S | 4 | only mortality ratios provided |  | exclude |
| [Yoshikawa et al. (2008)](#_ENREF_407) | consider |  | LS | LS | LS | LS | S | S | LS | LS | NA | S | 6 |  |  | exclude |

S = suitable; LS = less suitable; NS = not suitable

1. Qualifiers for Exposure Ascertainment:

M = municipal or village water average

KR = kriged average

U = urinary arsenic

toes = toenail arsenic

blood = blood arsenic

Inh = inhalation with no assessment of other routes of exposure

W = work history + estimated mg/m^3^ levels for different jobs and timeframes

2. Qualifiers for Exposure Uncertainty

(ranges) = exposure presented as ranges (numbers indicate numbers of strata)

SD, SE = standard deviation, standard error

3. Does not adjust for gender

4. Results might be affected by blackfoot disease incidence

5. Risks as a function of cumulative exposure only for total urinary cancer (not urothelial carcinoma)

6. Total creatinine-adjusted urinary arsenic reported, but no speciation and no documentation of fish consumption (to determine arsenobetaine and arsenocholine levels are not abnormally high)

7. Number of deaths, but not number of subjects, given for each exposure stratum

8. Averages reported but no measures of dispersion

9. Death rates not adjusted for effect of smoking due to lack of smoking information

#### Pregnancy Outcomes

Table S-10. Pregnancy outcomes exposure-response study selection

| **Study** | **Initial Screen. Rec.** | **Rationale for Initial Exclusion** | **Endpoint (I, M)** | **Exposure** | | **Est. Adj. (Smoking, Gender, Age)** | **Number** | | **Exposure** | | **Ref. Grp. Represent.** | **Sufficient Cases (Nos.)** | **Mark-downs** | **All Data  Available  for DR?** | **Author Provided Data?** | **Final Rec.** |
| --- | --- | --- | --- | --- | --- | --- | --- | --- | --- | --- | --- | --- | --- | --- | --- | --- |
|  |  |  |  | **Ascertain.** | **Uncertainty** |  | **Exp. Grps.** | **Subj., Cases Rept’d.** | **Metric** | **Timing, Dur.** |  |  |  |  |  |  |
| [Aelion et al. (2012)](#_ENREF_3) | exclude | ecological |  |  |  |  |  |  |  |  |  |  |  |  |  | exclude |
| [Ahamed et al. (2006b)](#_ENREF_5) | exclude | cross-sectional |  |  |  |  |  |  |  |  |  |  |  |  |  | exclude |
| [Ahmad et al. (2001)](#_ENREF_7) | exclude | cross-sectional |  |  |  |  |  |  |  |  |  |  |  |  |  | exclude |
| [Aschengrau et al. (1989)](#_ENREF_18) | consider |  | S spontan-eous abortion | NS (drinking-water sample) | NS reported as range; town avg.; women matched to town during preg. | S (specifics assumed – reported in another paper) | NS 3 groups (0.0014–0.0019, 0.0008–0.0013, UD µg/L) | S  153 exp., 690 contr. | S drink. Water | S  chronic; 79% of samples collect-ed w/i 4 mos. of concept-tion | S | S | 6 | number of subjects not reported | author provided number of subjects per exposure stratum | exclude |
| [Bloom et al. (2014)](#_ENREF_34) | consider | NA | S spontan-eous abortion | LS  recon-structed drinking-water exp. | LS mean, median, range; 3 indicators:  avg. in up to 2 sources, peak, daily exp. resi-dential water sources; 0.0–175.1 µg/L | S (modest interaction between As & smoking or vitamin use) | LS analyzed as tertiles but data not shown | S  150 cases, 150 controls | S drink. Water | S  avg. & peak | LS cases older than controls & less likely to use vitamins | S  150 cases, 150 controls | 4 | no, no. subjects per exp. stratum not provided |  | exclude |
| [Bloom et al. (2015)](#_ENREF_33) | consider | NA | S | S | LS (called tertile only) | LS (no adj. for infant sex) | LS (2+ref) | LS | S urine | S chronic during preg. | NA | S | 4 | no, regr. only |  | exclude |
| [Bloom et al. (2016)](#_ENREF_33) | consider | NA | S | S | LS | LS | LS | LS | S | S | NA | S | 4 | no, regr. only |  | exclude |
| [Chakraborti et al. (2003)](#_ENREF_44) | exclude | cross-sectional |  |  |  |  |  |  |  |  |  |  |  |  |  | exclude |
| [Cherry et al. (2008)](#_ENREF_77) | exclude | ecological |  |  |  |  |  |  |  |  |  |  |  |  |  | exclude |
| [Cherry et al. (2010)](#_ENREF_76) | exclude | ecological |  |  |  |  |  |  |  |  |  |  |  |  |  | exclude |
| [Davis et al. (2015)](#_ENREF_98) | exclude | cross-sectional |  |  |  |  |  |  |  |  |  |  |  |  |  | exclude |
| [El-Baz et al. (2015)](#_ENREF_102) | consider | NA | S  intra-uterine growth retard-ation | S | LS (regr.) | LS (not reported) | LS (regr.) | S | S urine | LS | LS | LS (only 60, but not categorized) | 6 | only beta provided |  | exclude |
| [Gardner et al. (2013)](#_ENREF_125) | consider | NA | S size at age 5 yrs. | S (individ-ual meas. graphed but not tabulated) | S (individual exp. & outcome data; sum of iAs & methylated metabolites adj. by specific gravity) | S evaluated Cd, SES & nutritional supplementa-tion, related to maternal nutrition | NS  2 | S  1,505 mother-child pairs | S urine | S chronic during preg. & before | S | S | 2 | no, only two dose groups |  | exclude |
| [Gelmann et al. (2013)](#_ENREF_126) | exclude | cross-sectional |  |  |  |  |  |  |  |  |  |  |  |  |  | exclude |
| Gilbert-Diamond et al. (2016) | consider | NA | S | S | S | LS | LS | S | S | S | S | S | 2 | No, results provided per unit increase in ln(As) and not stratified into exposure groups |  | exclude |
| [Guan et al. (2012)](#_ENREF_134) | exclude | blood as exp. metric |  |  |  |  |  |  |  |  |  |  |  |  |  | exclude |
| [Hopenhayn-Rich et al. (1999)](#_ENREF_167) | exclude | ecological |  |  |  |  |  |  |  |  |  |  |  | exp. reported by city of residence (ecological) |  | exclude |
| [Hopenhayn et al. (2003)](#_ENREF_168) | consider | NA | S  LBW | LS citywide avg. DW As | S (19 women/city urinary As subsample to confirm drinking-water levels/each per city) | S (including chemical coexp. & maternal stress) | NS compare unexp. city & exp. city | 424 infants Antofagasta (40 µg/L); 420 infants Valparaiso <1 µg/L | S drink. Water | S chronic during preg. & before | S | S | 3 | exp. reported by city of residence (ecological) |  | exclude |
| [Hopenhayn-Rich et al. (2000)](#_ENREF_166) | exclude | ecological |  |  |  |  |  |  |  |  |  |  |  |  |  | exclude |
| [Hu et al. (2015)](#_ENREF_179) | exclude | ecological |  |  |  |  |  |  |  |  |  |  |  |  |  | exclude |
| [Huyck et al. (2007)](#_ENREF_185) | consider | NA | S  LBW | LS  (mult. bio-markers & well-water samples; associa-tion w/hair & toenails but not water conc. >409 µg/L vs. unexp. <10 µg/L | LS (some inconsisten-cies in exp.) pop. exp. levels given as quartiles, range; individ. water for each pregnant woman assumed but not stated; As chemistry not spec-ified; water sample 6 mos. before. | S yes | S (quintiles w/ranges & linear regr. between biomarker & LBW) | LS 52 pregnant women; 49 live births | S drink. Water | S chronic during preg. & before | S | S | 3 | no, no risk estimates by exp. stratum |  | exclude |
| [Ihrig et al. (1998)](#_ENREF_186) | consider | NA | S  stillbirth | LS (air- dispersion modeling linked to maternal residence) | LS  modeled data | LS not smoking, but discusses historical smoking data to suggest it is not controlling factor | S (individ-ual est., but reported as quartiles incl. control | S  19,568 | S air | LS  not speci-fied | S | s | 3 |  |  | include |
| [Kippler et al. (2012)](#_ENREF_208) | exclude | Primarily cadmium exposure |  |  |  |  |  |  |  |  |  |  |  |  |  | exclude |
| [Kwok et al. (2006)](#_ENREF_214) | exclude | cross-sectional |  |  |  |  |  |  |  |  |  |  |  |  |  | exclude |
| [Laine et al. (2015)](#_ENREF_219) | exclude | cross-sectional |  |  |  |  |  |  |  |  |  |  |  |  |  | exclude |
| [Mcdermott et al. (2014)](#_ENREF_256) | exclude | soil as exp. measure |  |  |  |  |  |  |  |  |  |  |  |  |  | exclude |
| [Milton et al. (2005)](#_ENREF_268) | exclude | cross-sectional |  |  |  |  |  |  |  |  |  |  |  |  |  | exclude |
| [Mukherjee et al. (2005)](#_ENREF_278) | exclude | cross-sectional |  |  |  |  |  |  |  |  |  |  |  |  |  | exclude |
| [Myers et al. (2010)](#_ENREF_280) | exclude | ecological |  |  |  |  |  |  |  |  |  |  |  |  |  | exclude |
| [Rahman et al. (2005a)](#_ENREF_324) | exclude | cross-sectional |  |  |  |  |  |  |  |  |  |  |  |  |  | exclude |
| [Rahman et al. (2007)](#_ENREF_313) | consider | NA | S  stillbirth & infant mortality | LS (exp., individual pregnan-cies from As in tube well used during preg. & historic areawide monitor-ing) | LS group median & quintile intervals | S yes | S (9 exp. groups betw. <1 & >50 µg/L), grouped into quintiles; ~5,000 live births/ quintile | S  29,134 pregnan-cies; 2,444 fetal loss; 1,096 induced abortions; 850 neonatal deaths; 523 infant deaths | S drink. Water | S chronic during preg. & poten-tially early life | S | S | 2 |  |  | include |
| [Rahman et al. (2009)](#_ENREF_314) | consider | NA | S  LBW & size | S (urinary biomark-ers avg. 2 samples collected 8 & 30 wks.) | S (sum of iAs, methylated metabolites adj. by specific gravity; extensive well sampling in well-studied area – avg. well-water 78 µg/L (1–410 µg/L); food contrib. likely substantial; most infants breastfed, assume prenatal exp.) | S yes | S (linear regr. individual meas.; Gardner et al. (2013) graphs, does not tabulate individual exp. meas. | S  1,578 mother-infant pairs | S urine | S chronic during preg. & poten-tially early life | S | S | 0 | no, no risk estimates provided by exp. stratum |  | exclude |
| [Rahman et al. (2010)](#_ENREF_311) | consider | NA | S  spontan-eous abortion, stillbirth & infant mortality | S (exp. based on avg. individual, 2 urine samples collected 8 & 30 wks.) | S (as above for Rahman et al. 2009) | S water Mn not measured | S quintiles ~550 pregnan-cy/group for spontan-eous abortion | S  2,924 preg. associated w/275 spontan-eous abortions, 88 induced abortions, 52 stillbirths & 98 infant deaths | S urine | S chronic during preg. & poten-tially early life | NS | S | 2 |  |  | include |
| [Saha et al. (2012)](#_ENREF_334) | consider | NA | S  low birth size | S (U) As of mothers 8- & 30- wk gesta-tion & infants 18 mos.; U ~80 µg/L (25–400) mothers & 34 µg/L (12–159) children | S (as above for Rahman et al. 2009) | S (adj. assoc. betw. weight & length by U quintiles (linear regr.) by maternal age & BMI, SES, sex; assoc. markedly attenuated after adj. but significant) | S median U quintiles (lowest is reference group) | S  2372 infants | S urine | S chronic during preg. & before | S | S | 0 | no, no. subjects per exp. stratum not provided |  | exclude |
| [Sen and Chaudhuri (2008)](#_ENREF_337) | exclude | cross-sectional |  |  |  |  |  |  |  |  |  |  |  |  |  | exclude |
| [Thomas et al. (2015)](#_ENREF_362) | exclude | blood arsenic (& other metals); some urinary measurements, but most analyses compare 2 categories & results complicated by potential exp. via fish (as measured by arsenobetaine) |  |  |  |  |  |  | S urine |  |  |  |  |  |  | exclude |
| [Vall et al. (2012)](#_ENREF_374) | exclude | cross-sectional |  |  |  |  |  |  |  |  |  |  |  |  |  | exclude |
| [von Ehrenstein et al. (2006)](#_ENREF_376) | exclude | cross-sectional |  |  |  |  |  |  |  |  |  |  |  |  |  | exclude |
| [Yang et al. (2003)](#_ENREF_401) | exclude | ecological |  |  |  |  |  |  |  |  |  |  |  |  |  | exclude |

S = suitable; LS = less suitable; NS = not suitable

1. Qualifiers for Exposure Ascertainment:

M = municipal or village water average

KR = kriged average

U = urinary arsenic

toes = toenail arsenic

blood = blood arsenic

Inh = inhalation with no assessment of other routes of exposure

W = work history + estimated mg/m^3^ levels for different jobs and timeframes

2. Qualifiers for Exposure Uncertainty

(ranges) = exposure presented as ranges (numbers indicate numbers of strata)

SD, SE = standard deviation, standard error

3. Does not adjust for gender

4. Results might be affected by blackfoot disease incidence

5. Risks as a function of cumulative exposure only for total urinary cancer (not urothelial carcinoma)

6. Total creatinine-adjusted urinary arsenic reported, but no speciation and no documentation of fish consumption (to determine arsenobetaine and arsenocholine levels are not abnormally high)

7. Number of deaths, but not number of subjects, given for each exposure stratum

8. Averages reported but no measures of dispersion

9. Death rates not adjusted for effect of smoking due to lack of smoking information

#### Renal Cancer

Table S-11. Renal cancer exposure-response study selection

| **Study** | **Initial Screen. Rec.** | **Rationale for Initial Exclusion** | **Endpoint (I, M)** | **Exposure** | | **Est. Adj. (Smoking, Gender, Age)** | **Number** | | **Exposure** | | **Ref. Grp. Represent.** | **Sufficient Cases (Nos.)** | **Mark-downs** | **All Data  Available  for DR?** | **Author Provided Data?** | **Final Rec.** |
| --- | --- | --- | --- | --- | --- | --- | --- | --- | --- | --- | --- | --- | --- | --- | --- | --- |
|  |  |  |  | **Ascertain.** | **Uncertainty** |  | **Exp. Grps.** | **Subj., Cases Rept’d.** | **Metric** | **Timing, Dur.** |  |  |  |  |  |  |
| [Baastrup et al. (2008)](#_ENREF_20) | consider |  | S | LS (munici-pal water) | LS (regr.) | LS (no adj. for gender, age) | NA | LS (IRR) | S (cum. DW) | S | NA | S | 4 | continuous measure of exposure |  | exclude |
| [Besuschio et al. (1980)](#_ENREF_28) | exclude | ecological |  |  |  |  |  |  |  |  |  |  |  |  |  | exclude |
| [Boffetta et al. (2011)](#_ENREF_35) | exclude | no data for exposure-response (only compares exposed to not-exposed |  |  |  |  |  |  |  |  |  |  |  |  |  | exclude |
| [Buchet and Lison (1998)](#_ENREF_40) | exclude | ecological |  |  |  |  |  |  |  |  |  |  |  |  |  | exclude |
| [Chen and Wang (1990)](#_ENREF_55) | consider |  | LS | LS (munici-pal water) | LS (regr.) | LS (no adj. for gender, smoking) | NA | LS | LS | LS | NA | S | 7 |  |  | exclude |
| [Chen et al. (1992)](#_ENREF_48) | consider |  | LS | LS | LS | LS (no adj. for smoking) | S (4) | LS (4 or fewer in high dose group) | LS | S | S | LS | 7 |  |  | exclude |
| [Chen et al. (1985)](#_ENREF_54) | exclude | ecological |  |  |  |  |  |  |  |  |  |  |  |  |  | exclude |
| [Chen et al. (1988)](#_ENREF_52) | exclude | ecological |  |  |  |  |  |  |  |  |  |  |  |  |  | exclude |
| [Chen et al. (2014)](#_ENREF_60) | exclude | relative risk for cancer mortality based on 1‑ppm increase in soil As |  |  |  |  |  |  |  |  |  |  |  |  |  | exclude |
| [D'Ippoliti et al. (2015)](#_ENREF_92) | consider | note: no data on no. subjects per exp. stratum; requested information from study authors | LS (mortal-ity) | LS | LS (range) | S | LS (2+ref) | S | S | S | NA | S | 4 | number of subjects not reported | author provided number of subjects per exposure stratum | include |
| [Enterline et al. (1995)](#_ENREF_104) | consider |  | LS | LS | LS | LS | LS | LS | LS | LS | LS | LS | 10 |  |  | exclude |
| [Ferreccio et al. (2013a)](#_ENREF_120) | consider |  | S | LS | LS | S | S | S | S | S | S | S | 2 |  |  | include |
| [García-Esquinas et al. (2013)](#_ENREF_123) | consider |  | LS | S | LS (regr.) | S | S | NA | S | LS | NA | S | 3 |  |  | include |
| [Guo et al. (1997)](#_ENREF_145) | consider |  | S | LS (wells) | LS (ranges) | LS (adj. for smoking but by using cigarettes sold per capita) | S | LS (regr.) | LS | LS | NA | S | 6 |  |  | exclude |
| [Han et al. (2009)](#_ENREF_155) | exclude | ecological |  |  |  |  |  |  |  |  |  |  |  |  |  | exclude |
| [Hinwood et al. (1999)](#_ENREF_162) | exclude | ecological |  |  |  |  |  |  |  |  |  |  |  |  |  | exclude |
| [Hopenhayn-Rich et al. (1998)](#_ENREF_165) | exclude | ecological |  |  |  |  |  |  |  |  |  |  |  |  |  | exclude |
| [Huang et al. (2011)](#_ENREF_180) | exclude | no exp.-response data |  |  |  |  |  |  |  |  |  |  |  |  |  | exclude |
| [Huang et al. (2012)](#_ENREF_181) | consider |  | S | LS | LS (ranges) | S | S | S | S (U) | S | S | S | 2 |  |  | include |
| [Kurttio et al. (1999)](#_ENREF_211) | consider |  | S | LS | LS (ranges) | S | S | S | S (conc. in water; daily dose; cum. dose) | S | S | S | 2 | no, tot. no. subjects per exp. stratum not provided |  | exclude |
| [Lewis et al. (1999)](#_ENREF_225) | exclude | standardized mortality ratio by exp. group |  |  |  |  |  |  |  |  |  |  |  |  |  | exclude |
| [Meliker et al. (2007)](#_ENREF_262) | exclude | ecological |  |  |  |  |  |  |  |  |  |  |  |  |  | exclude |
| [Mikoczy et al. (1996)](#_ENREF_265) | exclude | only 2 exp. groups, referent & >0.5 & very few cases (only 6 tot. kidney cancer cases) |  |  |  |  |  |  |  |  |  |  |  |  |  | exclude |
| [Mostafa and Cherry (2013)](#_ENREF_276) | consider |  | S | LS | LS (ranges) | S | S | S | S | S | S | S | 2 |  |  | include |
| [Rivara et al. (1997)](#_ENREF_330) | exclude | no exp.-response data |  |  |  |  |  |  |  |  |  |  |  |  |  | exclude |
| [Sawada et al. (2013)](#_ENREF_335) | consider |  | S | LS | S (median) | S | S | S | S | S | NA | S | 1 |  |  | include |
| [Smith et al. (1998)](#_ENREF_344) | exclude | ecological |  |  |  |  |  |  |  |  |  |  |  |  |  | exclude |
| [Smith et al. (2012)](#_ENREF_345) | exclude | ecological |  |  |  |  |  |  |  |  |  |  |  |  |  | exclude |
| [Tsai et al. (1999)](#_ENREF_364) | exclude | no exp.-response data |  |  |  |  |  |  |  |  |  |  |  |  |  | exclude |
| [Wu et al. (1989)](#_ENREF_397) | consider |  | LS | LS | LS (ranges) | LS | S | NA | LS | S | S | LS | 6 |  |  | exclude |
| [Yang et al. (2004)](#_ENREF_403) | exclude | ecological |  |  |  |  |  |  |  |  |  |  |  |  |  | exclude |
| [Yuan et al. (2010)](#_ENREF_409) | exclude | no exp.-response data |  |  |  |  |  |  |  |  |  |  |  |  |  | exclude |

S = suitable; LS = less suitable; NS = not suitable

1. Qualifiers for Exposure Ascertainment:

M = municipal or village water average

KR = kriged average

U = urinary arsenic

toes = toenail arsenic

blood = blood arsenic

Inh = inhalation with no assessment of other routes of exposure

W = work history + estimated mg/m^3^ levels for different jobs and timeframes

2. Qualifiers for Exposure Uncertainty

(ranges) = exposure presented as ranges (numbers indicate numbers of strata)

SD, SE = standard deviation, standard error

3. Does not adjust for gender

4. Results might be affected by blackfoot disease incidence

5. Risks as a function of cumulative exposure only for total urinary cancer (not urothelial carcinoma)

6. Total creatinine-adjusted urinary arsenic reported, but no speciation and no documentation of fish consumption (to determine arsenobetaine and arsenocholine levels are not abnormally high)

7. Number of deaths, but not number of subjects, given for each exposure stratum

8. Averages reported but no measures of dispersion

9. Death rates not adjusted for effect of smoking due to lack of smoking information

## Author-Provided Data

As part of the RRB analysis study selection process, authors were contacted for additional data whenever the study had fewer than 5 markdowns but not all data needed for the exposure-response analysis was available. Whether or not data were provided is shown in the study selection tables, in the “Author Provided Data?” column. In this appendix section in the following tables, the data supplied by the authors is documented.

Table S-12. Data provided for [Argos et al. (2007)](#_ENREF_16)

| [Argos et al. (2007)](#_ENREF_16) | Well water arsenic concentration (μg/L) | Cases | | | Non-Cases | Notes |  |
| --- | --- | --- | --- | --- | --- | --- | --- |
| Skin Lesions (Owns Land) | <7 | 18 | | | 839 | Cases and Non-Cases provided by author | |
| Hero ID: 627505 | 7–38 | 20 | | | 772 |  |  |
|  | 39–90 | 46 | | | 807 |  |  |
|  | 91–177 | 38 | | | 774 |  |  |
|  | >177 | 51 | | | 664 |  |  |
| [Argos et al. (2007)](#_ENREF_16) | Well water arsenic concentration (μg/L) | Cases | | | Non-Cases | Notes |  |
| Skin Lesions (Does not own land) | <7 | 39 | | | 1,309 | Cases and Non-Cases provided by author |  |
| Hero ID: 627505 | 7–38 | 76 | | | 1,503 |  |  |
|  | 39–90 | 91 | | | 1,398 |  |  |
|  | 91–177 | 114 | | | 1,442 |  |  |
|  | >177 | 188 | | | 1,577 |  |  |
| [**Argos et al. (2007)**](#_ENREF_16) | Urinary As Concentration (μg/g-creatinine) | | Cases | | Non-Cases | Notes |  |
| Skin Lesions (Owns land) | ≤35 | | 38 | | 1,018 | Cases and Non-Cases provided by author |  |
| Hero ID: 627505 | 36–66 | | 50 | | 1,156 |  |  |
|  | 67–114 | | 48 | | 1,072 |  |  |
|  | 115–204 | | 90 | | 1,089 |  |  |
|  | >204 | | 82 | | 1,021 |  |  |
| [**Argos et al. (2007)**](#_ENREF_16) | Urinary As Concentration (μg/g-creatinine) | | Cases | | Non-Cases | Notes |  |
| Skin Lesions (Does not own land) | ≤35 | | 36 | | 1,101 | Cases and Non-Cases provided by author |  |
| Hero ID: 627505 | 36–66 | | 49 | | 1,050 |  |  |
|  | 67–114 | | 70 | | 1,079 |  |  |
|  | 115–204 | | 78 | | 1,039 |  |  |
|  | >204 | | 125 | | 1,149 |  |  |
| [**Argos et al. (2007)**](#_ENREF_16) | Cumulative As exposure (mg) | | Cases | Non-Cases | | Notes |  |
| Skin Lesions (Owns land) | ≤62 | | 34 | 1,208 | | Cases and Non-Cases provided by author |  |
| Hero ID: 627505 | 62–224 | | 37 | 933 | |  |  |
|  | 225–583 | | 54 | 1,096 | |  |  |
|  | 584–1490 | | 74 | 1,023 | |  |  |
|  | >1490 | | 110 | 1,049 | |  |  |
| [**Argos et al. (2007)**](#_ENREF_16) | Cumulative As exposure (mg) | | Cases | Non-Cases | | Notes |  |
| Skin Lesions (Does not own land) | ≤62 | | 27 | 1,169 | | Cases and Non-Cases provided by author |  |
| Hero ID: 627505 | 62–224 | | 35 | 940 | |  |  |
|  | 225–583 | | 65 | 1,070 | |  |  |
|  | 584–1490 | | 84 | 1,136 | |  |  |
|  | >1490 | | 148 | 1,095 | |  |  |

Table S-13. Data provided for [Aschengrau et al. (1989)](#_ENREF_18)

| [**Aschengrau et al. (1989)**](#_ENREF_18) | Drinking water As exposure (μg/L) | Cases | Non-Cases | Notes |
| --- | --- | --- | --- | --- |
| Spontaneous abortion/miscarriage | Not detected | 128 | 701 | Cases and Non-Cases provided by author |
| Hero ID: 1032517 | 0.8–1.3 | 151 | 668 |  |
|  | 1.4–1.9 | 7 | 22 |  |

Table S-14. Data provided for [Chen et al. (2011b)](#_ENREF_68)

| [**Chen et al. (2011b)**](#_ENREF_68) | Drinking water As concentration- by mean (μg/L) | Total N | Notes |
| --- | --- | --- | --- |
| Hero ID: 1015960 | 3.7 | 2,982 | Total N per exposure level provided by author |
|  | 35.9 | 2,943 |  |
|  | 102.5 | 2,886 |  |
|  | 265.7 | 2,935 |  |
| [**Chen et al. (2011b)**](#_ENREF_68) | Urinary Arsenic Concentration - by mean (μg/g creatinine) | Total N | Notes |
| Hero ID: 1015960 | 68.5 | 2,793 | Total N per exposure level provided by author |
|  | 150.6 | 2,829 |  |
|  | 264.9 | 2,805 |  |
|  | 641.5 | 2,797 |  |

Table S-15. Data provided for [D'Ippoliti et al. (2015)](#_ENREF_92)

| [**D'Ippoliti et al. (2015)**](#_ENREF_92) | Average As during first year of residence (μg/L) | Total N | Notes |
| --- | --- | --- | --- |
| Males | <10 | 21,997 | Total N per exposure level provided by author |
| Hero ID: 3005297 | 10–20 | 20,533 |  |
|  | >20 | 26,228 |  |
| [**D'Ippoliti et al. (2015)**](#_ENREF_92) | Average As during first year of residence (μg/L) | Total N | Notes |
| Females | <10 | 22,347 | Total N per exposure level provided by author |
| Hero ID: 3005297 | 10–20 | 20,946 |  |
|  | >20 | 26,749 |  |
| [**D'Ippoliti et al. (2015)**](#_ENREF_92) | Cumulative As Dose (mg) | Total N | Notes |
| Males | <204.9 | 26,944 | Total N per exposure level and unit provided by author |
| Hero ID: 3005297 | 204.9–804.0 | 44,537 |  |
|  | >804 | 21,636 |  |
| [**D'Ippoliti et al. (2015)**](#_ENREF_92) | Cumulative As Dose (mg) | Total N | Notes |
| Females | <204.9 | 26,459 | Total N per exposure level and unit provided by author |
| Hero ID: 3005297 | 204.9–804.0 | 44,702 |  |
|  | >804 | 23,175 |  |

Table S-16. Data provided for [Gilbert-Diamond et al. (2013)](#_ENREF_129)

| [**Gilbert-Diamond et al. (2013)**](#_ENREF_129) | Total Urinary Arsenic (μg/L) | Cases | Controls | Notes |
| --- | --- | --- | --- | --- |
| Hero ID: 1797805 | <3.36 | 165 | 141 | Cases and controls provided by author |
|  | 3.36–<5.31 | 145 | 161 |  |
|  | >=5.31 | 137 | 168 |  |
| [**Gilbert-Diamond et al. (2013)**](#_ENREF_129) | Inorganic urinary As ( μg/L) | Cases | Controls | Notes |
| Hero ID: 1797805 | <0.23 | 156 | 153 | Cases and controls provided by author |
|  | 0.23–<0.45 | 149 | 153 |  |
|  | >=0.45 | 142 | 164 |  |

Table S-17. Data provided for [James et al. (2015)](#_ENREF_190)

| [**James et al. (2015)**](#_ENREF_190) | As TWA exposure (μg g/L-yr) | Mean As TWA exposure (μg g/L-yr) | Cases | Non- Cases | Notes |
| --- | --- | --- | --- | --- | --- |
| coronary heart disease | 1–20 | 7.31 | 58 | 370 | Means and cases provided by author and non-cases calculated against total n presented in paper |
| Hero ID: 2822189 | 20–30 | 25.1 | 18 | 68 |  |
|  | 30–45 | 36.6 | 16 | 17 |  |
|  | 45–88 | 50.2 | 4 | 4 |  |

Table S-18. Data provided for [Moon et al. (2013)](#_ENREF_270)

| [**Moon et al. (2013)**](#_ENREF_270) | Urinary As concentration (μg /g-creatinine) | Mean Urinary As concentration  (μg/g-creatinine) | Cases | Non-Cases | Notes |
| --- | --- | --- | --- | --- | --- |
| coronary heart disease incidence | <5.8 | 4.10 | 202 | 694 | Means and non-cases provided by author |
| Hero ID: 2064267 | 5.8–9.7 | 7.60 | 206 | 687 |  |
|  | 9.8–15.7 | 12.5 | 197 | 695 |  |
|  | >15.7 | 26.3 | 241 | 653 |  |
| [**Moon et al. (2013)**](#_ENREF_270) | Urinary As concentration (μg/g-creatinine) | Mean Urinary As concentration  (μg /g-creatinine) | Cases | Non-Cases | Notes |
| coronary heart disease mortality | <5.8 | 4.10 | 68 | 828 | Means and non-cases provided by author |
| Hero ID: 2064267 | 5.8–9.7 | 7.60 | 67 | 826 |  |
|  | 9.8–15.7 | 12.5 | 87 | 805 |  |
|  | >15.7 | 26.3 | 119 | 775 |  |
| [**Moon et al. (2013)**](#_ENREF_270) | Urinary As concentration (μg/g-creatinine) | Mean Urinary As concentration  (μg/g-creatinine) | Cases | Non-Cases | Notes |
| cardiovascular disease incidence | <5.8 | 4.10 | 265 | 631 | Means and non-cases provided by author |
| Hero ID: 2064267 | 5.8–9.7 | 7.60 | 297 | 596 |  |
|  | 9.8–15.7 | 12.5 | 291 | 601 |  |
|  | >15.7 | 26.3 | 331 | 563 |  |
| [**Moon et al. (2013)**](#_ENREF_270) | Urinary As concentration (μg/g-creatinine) | Mean Urinary As concentration  (μg/g-creatinine) | Cases | Non-Cases | Notes |
| cardiovascular disease mortality | <5.8 | 4.10 | 68 | 828 | Means and non-cases provided by author |
| Hero ID: 2064267 | 5.8–9.7 | 7.60 | 67 | 826 |  |
|  | 9.8–15.7 | 12.5 | 87 | 805 |  |
|  | >15.7 | 26.3 | 119 | 775 |  |
| [**Moon et al. (2013)**](#_ENREF_270) | Urinary As concentration (μg/g-creatinine) | Mean Urinary As concentration  (μg/g-creatinine) | Cases | Non-Cases | Notes |
| stroke incidence | <5.8 | 4.10 | 55 | 841 | Means and non-cases provided by author |
| Hero ID: 2064267 | 5.8–9.7 | 7.60 | 75 | 818 |  |
|  | 9.8–15.7 | 12.5 | 62 | 830 |  |
|  | >15.7 | 26.3 | 72 | 822 |  |
| [**Moon et al. (2013)**](#_ENREF_270) | Urinary As concentration (μg/g-creatinine) | Mean Urinary As concentration  (μg/g-creatinine) | Cases | Non-Cases | Notes |
| stroke mortality | <5.8 | 4.10 | 6 | 890 | Means and non-cases provided by author |
| Hero ID: 2064267 | 5.8–9.7 | 7.60 | 17 | 876 |  |
|  | 9.8–15.7 | 12.5 | 13 | 879 |  |
|  | >15.7 | 26.3 | 18 | 876 |  |

Table S-19. Data provided for [Sohel et al. (2009)](#_ENREF_349)

| [**Sohel et al. (2009)**](#_ENREF_349) | Average Historic Arsenic exposure (μg/L) | Mean Historic Arsenic exposure (μg/L) | Notes |
| --- | --- | --- | --- |
| Hero ID: 710822 | <10 | 1.40 | Means provided by author |
|  | 10–49 | 31.1 |  |
|  | 50–149 | 97.0 |  |
|  | 150–299 | 209 |  |
|  | >=300 | 403 |  |

Table S-20. Data provided for [Tseng et al. (2003)](#_ENREF_367)

| [**Tseng et al. (2003)**](#_ENREF_367) | Total Urinary Arsenic (μg/L) | Cases | Non-Cases | Notes |
| --- | --- | --- | --- | --- |
| Hero ID: 628705 | 0 | 4 | 73 | Cases and non-cases provided by author |
|  | 0.1–14.9 | 15 | 123 |  |
|  | >=15 | 35 | 110 |  |

Table S-21. Data provided for [Wade et al. (2009)](#_ENREF_379)

| [**Wade et al. (2009)**](#_ENREF_379) | Drinking water As concentration (μg/L) | Mean drinking water As concentration (μg/L) | Notes |
| --- | --- | --- | --- |
| Hero ID: 628466 | 0–5 | 1.61 | Means provided by author |
|  | 5.1–20 | 12.0 |  |
|  | 20.1–100 | 38.9 |  |
|  | 100.1–300 | 168 |  |
|  | >300 | 421 |  |

Table S-22. Data provided for [Wade et al. (2015)](#_ENREF_378)

| [**Wade et al. (2015)**](#_ENREF_378) | Water As concentration (μg/L) | Mean water As concentration (μg/L) | Notes |
| --- | --- | --- | --- |
| Hero ID: 2854656 | <10 | 3.02 | Means provided by author |
|  | 10–39 | 20.9 |  |
|  | >=40 | 78.8 |  |

Table S-23. Data provided for [Wasserman et al. (2004)](#_ENREF_390)

| [Wasserman et al. (2004)](#_ENREF_390) | Drinking water As concentration (μg/L) | Total N | Full IQ score mean | SE | Notes | |
| --- | --- | --- | --- | --- | --- | --- |
| Hero ID: 180230 | 0.1–5.5 | 50 | 76.56 | 2.87 | Total N per exposure level and Full IQ score mean (SE) provided by author |  |
|  | 5.6–50.0 | 50 | 71.81 | 2.89 |  |  |
|  | 50.1–176 | 50 | 68.80 | 2.99 |  |  |
|  | 177–790 | 51 | 65.25 | 2.89 |  |  |
| [Wasserman et al. (2004)](#_ENREF_390) | Drinking water As concentration (μg/L) | Total N | Performance IQ score mean | SE | Notes |  |
| Hero ID: 180230 | 0.1–5.5 | 50 | 59.51 | 2.44 | Total N per exposure level and Performance IQ score mean (SE) provided by author |  |
|  | 5.6–50.0 | 50 | 54.41 | 2.46 |  |  |
|  | 50.1–176 | 50 | 52.23 | 2.55 |  |  |
|  | 177–790 | 51 | 49.77 | 2.46 |  |  |
| [Wasserman et al. (2004)](#_ENREF_390) | Drinking water As concentration (μg/L) | Total N | Verbal IQ score mean | SE | Notes |  |
| Hero ID: 180230 | 0.1–5.5 | 50 | 17.05 | 0.74 | Total N per exposure level and Verbal IQ score mean (SE) provided by author |  |
|  | 5.6–50.0 | 50 | 17.40 | 0.75 |  |  |
|  | 50.1–176 | 50 | 16.56 | 0.78 |  |  |
|  | 177–790 | 51 | 15.47 | 0.75 |  |  |

Table S-24. Data provided for [Wu et al. (2012b)](#_ENREF_395)

| [Wu et al. (2012b)](#_ENREF_395) | Well water As (μg/L) | MMP-9 mean (ng/mL) | SD | Notes |
| --- | --- | --- | --- | --- |
| Hero ID: 1070384 | 0.10–2.00 | 105.90 | 59.30 | MMP-9 mean (SD) provided by author |
|  | 2.01–23.13 | 101.20 | 78.70 |  |
|  | 23.14–73.46 | 104.10 | 62.20 |  |
|  | 73.47–500.62 | 107.00 | 70.10 |  |
| [Wu et al. (2012b)](#_ENREF_395) | Well water As (μg/L) | MPO (ng/mL) | SD | Notes |
| Hero ID: 1070384 | 0.10–2.00 | 21.00 | 16.30 | MPO mean (SD) provided by author |
|  | 2.01–23.13 | 20.30 | 14.10 |  |
|  | 23.14–73.46 | 20.20 | 14.70 |  |
|  | 73.47–500.62 | 20.30 | 14.00 |  |
| [Wu et al. (2012b)](#_ENREF_395) | Well water As (μg/L) | sE-selectin (ng/mL) | SD | Notes |
| Hero ID: 1070384 | 0.10–2.00 | 35.30 | 14.60 | sE-selectin mean (SD) provided by author |
|  | 2.01–23.13 | 36.30 | 17.00 |  |
|  | 23.14–73.46 | 34.80 | 14.30 |  |
|  | 73.47–500.62 | 35.70 | 17.10 |  |
| [Wu et al. (2012b)](#_ENREF_395) | Well water As (μg/L) | PAI-1 (ng/mL) | SD | Notes |
| Hero ID: 1070384 | 0.10–2.00 | 72.60 | 14.60 | PAI-1 mean (SD) provided by author |
|  | 2.01–23.13 | 70.90 | 17.00 |  |
|  | 23.14–73.46 | 68.80 | 14.30 |  |
|  | 73.47–500.62 | 81.70 | 17.10 |  |
| [Wu et al. (2012b)](#_ENREF_395) | Well water As (μg/L) | sICAM-1 (ng/mL) | SD | Notes |
| Hero ID: 1070384 | 0.10–2.00 | 160.70 | 145.00 | sICAM-1 mean (SD) provided by author |
|  | 2.01–23.13 | 134.30 | 125.50 |  |
|  | 23.14–73.46 | 136.00 | 94.60 |  |
|  | 73.47–500.62 | 156.30 | 125.00 |  |
| [Wu et al. (2012b)](#_ENREF_395) | Well water As (μg/L) | sVCAM-1 (ng/mL) | SD | Notes |
| Hero ID: 1070384 | 0.10–2.00 | 1,001.70 | 325.10 | sVCAM-1 mean (SD) provided by author |
|  | 2.01–23.13 | 1,054.70 | 331.20 |  |
|  | 23.14–73.46 | 1,109.50 | 312.40 |  |
|  | 73.47–500.62 | 1,117.90 | 344.10 |  |
| [Wu et al. (2012b)](#_ENREF_395) | Urinary As (μg/g creatinine) | MMP-9 mean (ng/mL) | SD | Notes |
| Hero ID: 1070384 | 12.05–88.21 | 116.00 | 74.30 | MMP-9 mean (SD) provided by author |
|  | 88.22–141.69 | 99.50 | 63.30 |  |
|  | 141.7–275.63 | 97.40 | 63.00 |  |
|  | 275.64-1,869.57 | 105.00 | 65.90 |  |
| [Wu et al. (2012b)](#_ENREF_395) | Urinary As (μg/g creatinine) | MPO (ng/mL) | SD | Notes |
| Hero ID: 1070384 | 12.05–88.21 | 22.60 | 16.90 | MPO mean (SD) provided by author |
|  | 88.22–141.69 | 18.90 | 12.10 |  |
|  | 141.7–275.63 | 20.50 | 15.40 |  |
|  | 275.64–1,869.57 | 19.80 | 14.10 |  |
| [Wu et al. (2012b)](#_ENREF_395) | Urinary As (μg/g creatinine) | sE-selectin (ng/mL) | SD | Notes |
| Hero ID: 1070384 | 12.05–88.21 | 37.40 | 16.30 | sE-selectin mean (SD) provided by author |
|  | 88.22–141.69 | 35.00 | 15.10 |  |
|  | 141.7–275.63 | 33.70 | 14.60 |  |
|  | 275.64–1,869.57 | 35.50 | 16.40 |  |
| [**Wu et al. (2012b)**](#_ENREF_395) | Urinary As (μg/g creatinine) | PAI-1 (ng/mL) | SD | Notes |
| Hero ID: 1070384 | 12.05–88.21 | 74.10 | 34.70 | PAI-1 mean (SD) provided by author |
|  | 88.22–141.69 | 71.30 | 39.50 |  |
|  | 141.7–275.63 | 68.20 | 31.90 |  |
|  | 275.64-1,869.57 | 80.70 | 48.00 |  |
| [**Wu et al. (2012b)**](#_ENREF_395) | Urinary As (μg/g creatinine) | sICAM-1 (ng/mL) | SD | Notes |
| Hero ID: 1070384 | 12.05–88.21 | 149.60 | 139.70 | sICAM-1 mean (SD) provided by author |
|  | 88.22–141.69 | 142.20 | 114.50 |  |
|  | 141.7–275.63 | 154.00 | 144.70 |  |
|  | 275.64–1,869.57 | 147.20 | 92.30 |  |
| [**Wu et al. (2012b)**](#_ENREF_395) | Urinary As (μg/g creatinine) | sVCAM-1 (ng/mL) | SD | Notes |
| Hero ID: 1070384 | 12.05–88.21 | 1,010.90 | 320.90 | sVCAM-1 mean (SD) provided by author |
|  | 88.22–141.69 | 1,053.50 | 345.90 |  |
|  | 141.7–275.63 | 1,120.80 | 331.60 |  |
|  | 275.64–1,869.57 | 1,126.60 | 314.70 |  |

## Additional Graphs and Tables

#### Bladder Cancer Exposure-Response Modeling Results

The analysis of arsenic exposure response on bladder cancer outcomes evaluated 76 datasets from 20 peer reviewed studies that included endpoints such as urothelial carcinoma and urinary transitional cell carcinoma. A summary of datasets modeled identifying the study design, location, exposure metric and outcome domain is provided in Table S-25. A breakdown of the exposure levels and RRE_20_ estimates are provided for each exposure metric in Figure S-1–Figure S-7. Finally, RRE_20_ summary tables for all exposures are provided in Table S-26.


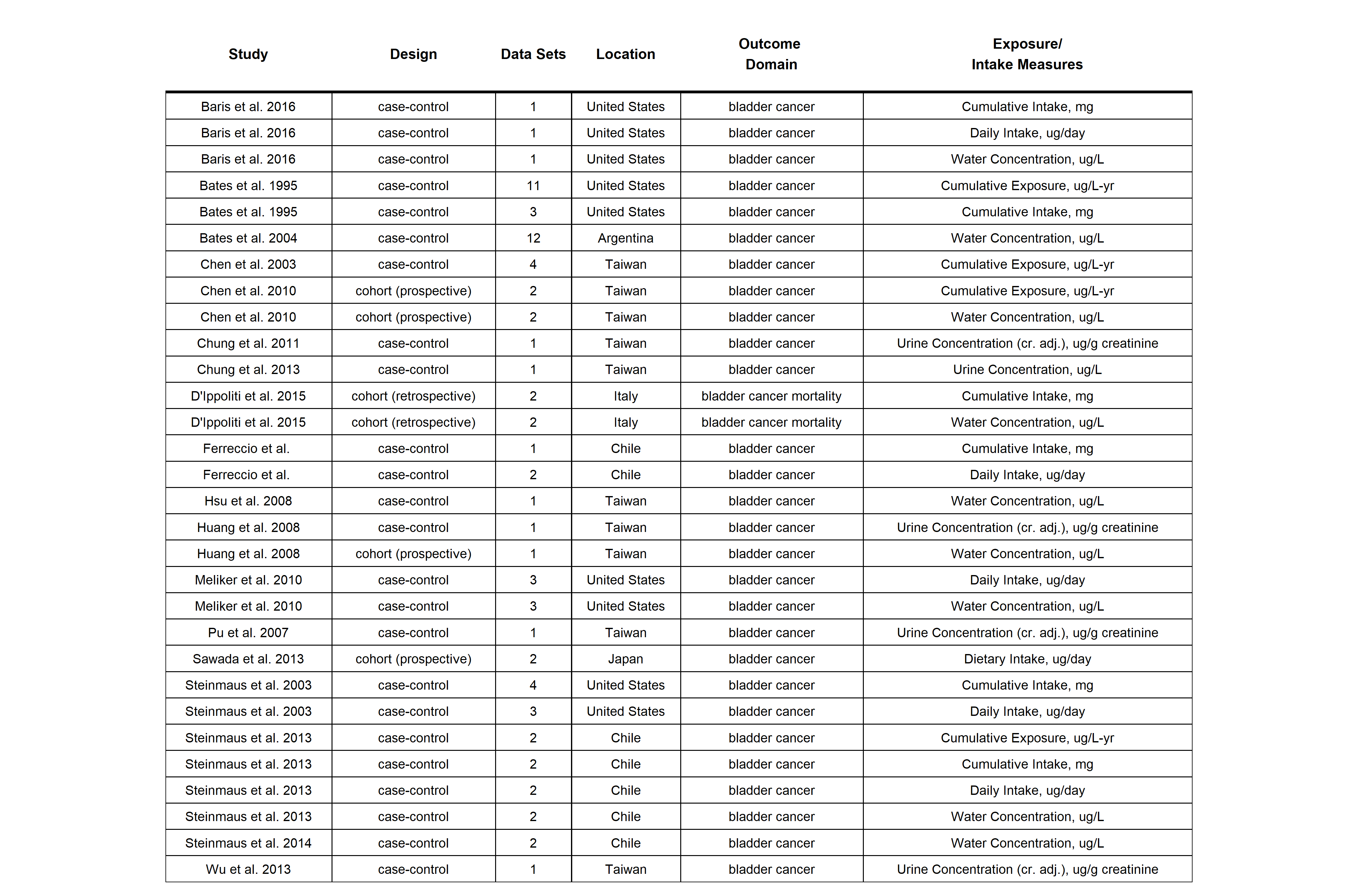
Table S-25. Summary of datasets considered in bladder cancer exposure response RRB analysis by exposure metric


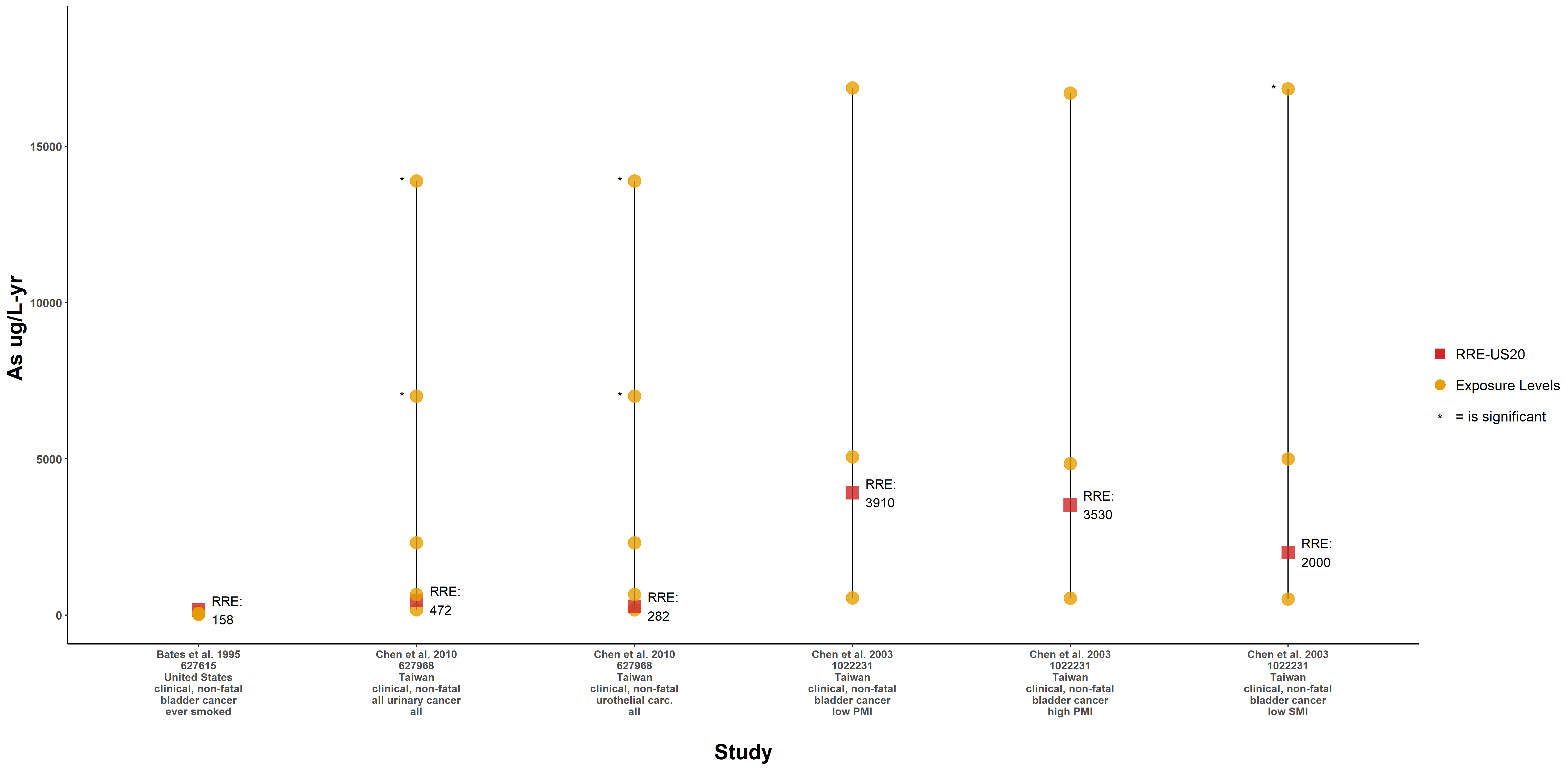


Figure S-1A. Exposure levels and RRE-US_20_ for bladder cancer using cumulative exposure.


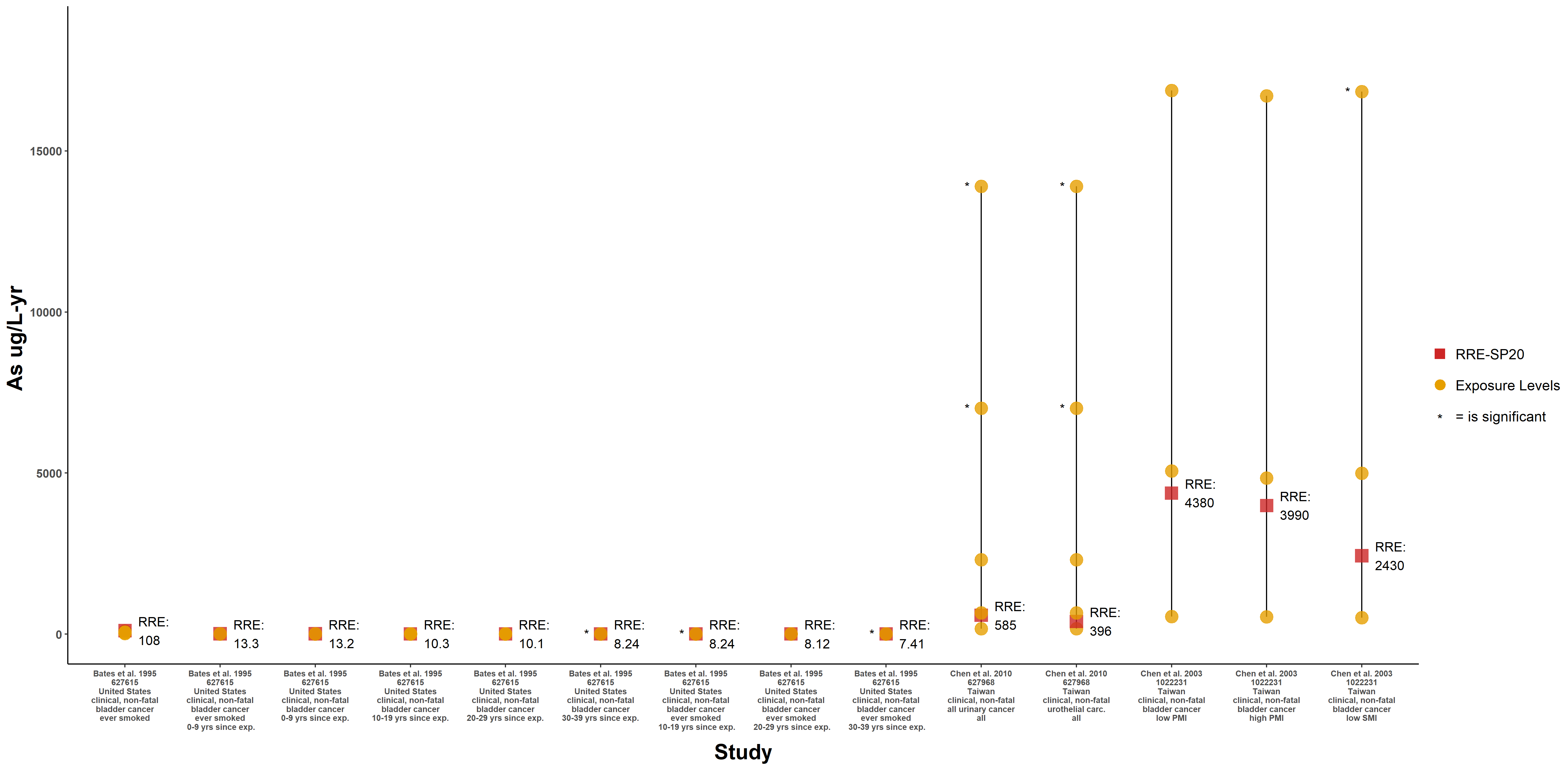


Figure S-1B. Exposure levels and RRE-SP_20_ for bladder cancer using cumulative exposure.


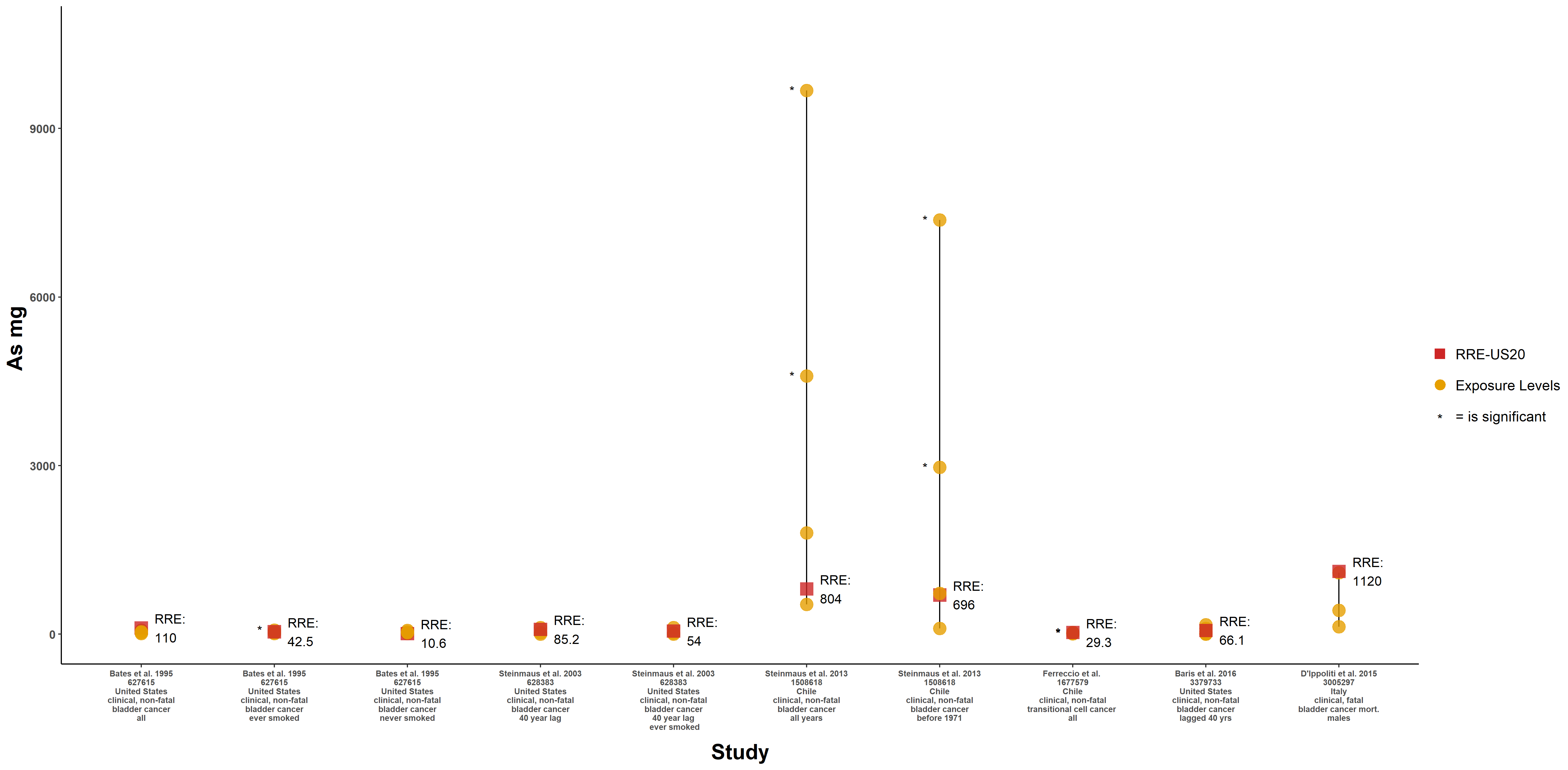


Figure S-2A. Exposure levels and RRE-US_20_ for bladder cancer using cumulative intake.


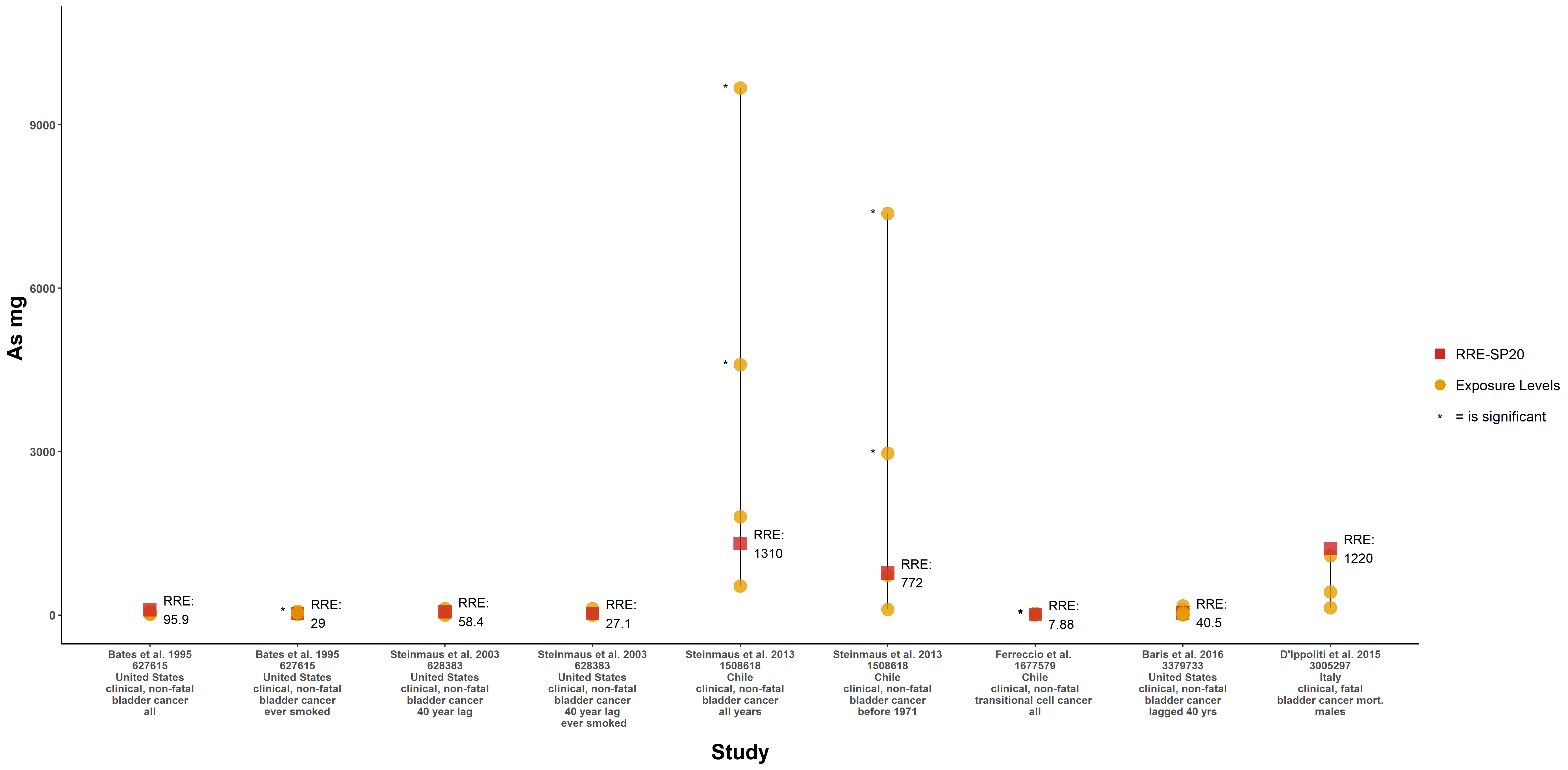


Figure S-2B. Exposure levels and RRE-SP_20_ for bladder cancer using cumulative intake.


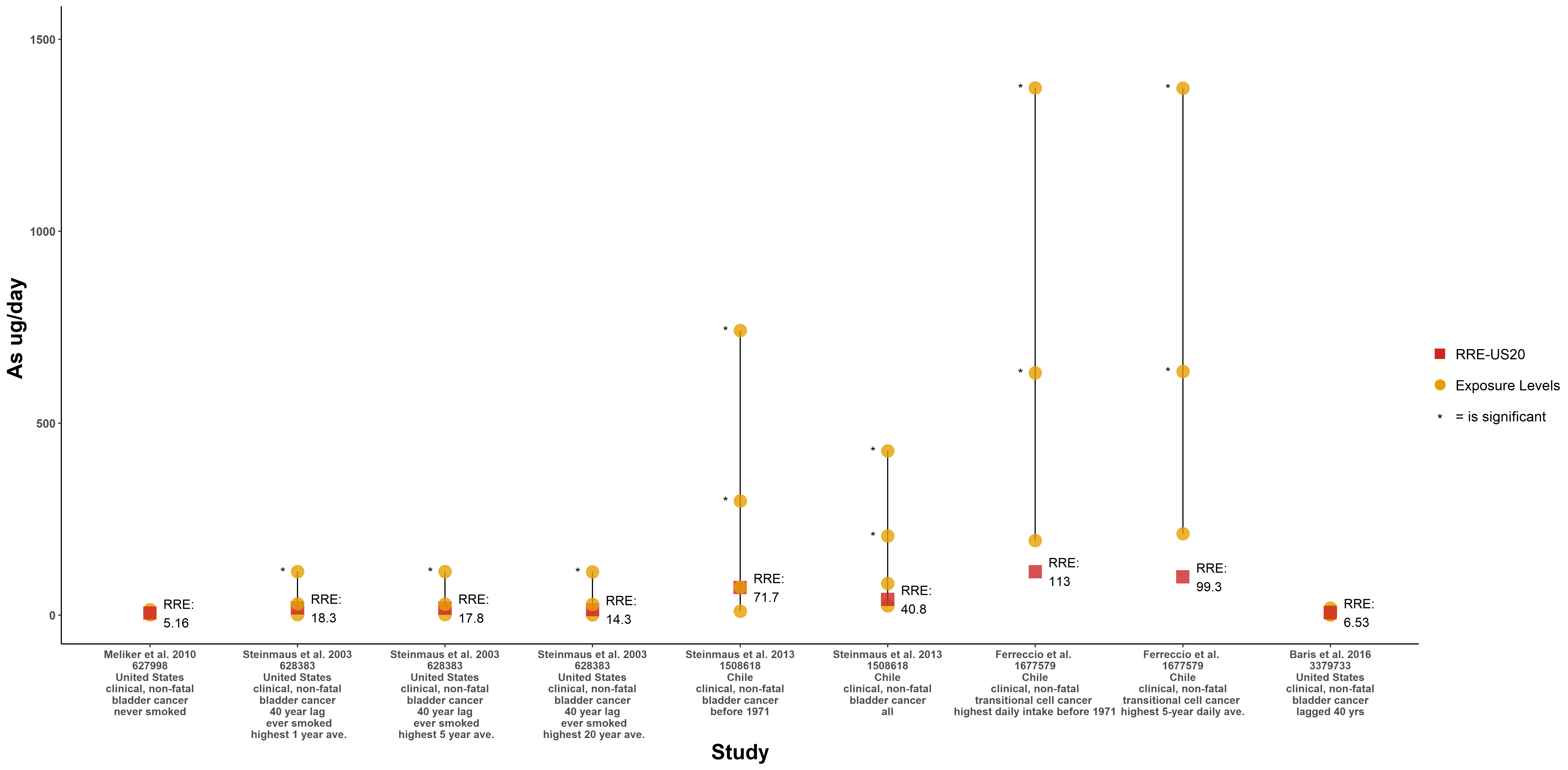


Figure S-3A. Exposure levels and RRE-US_20_ for bladder cancer using daily intake.


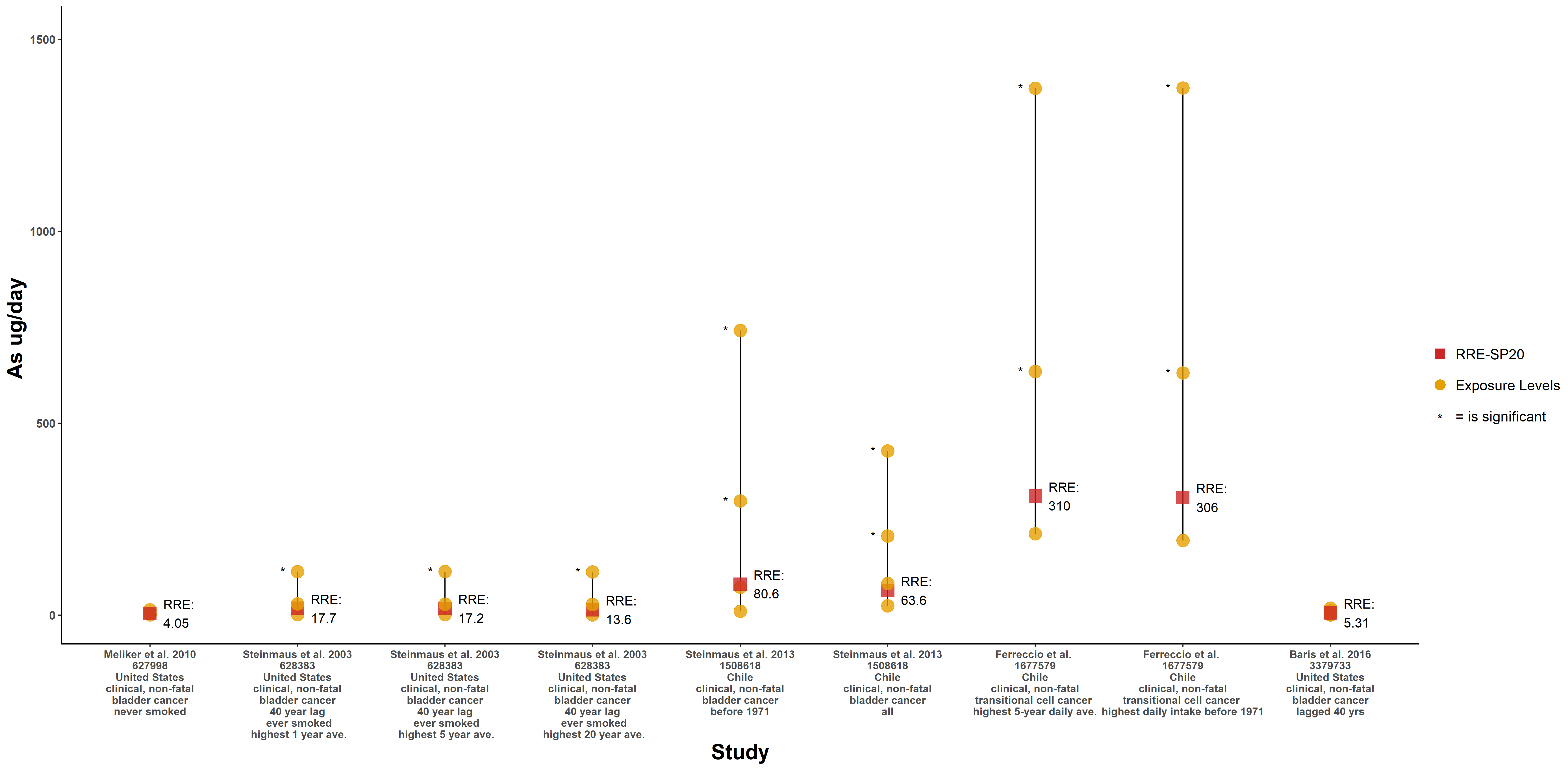


Figure S-3B. Exposure levels and RRE-SP_20_ for bladder cancer using daily intake.


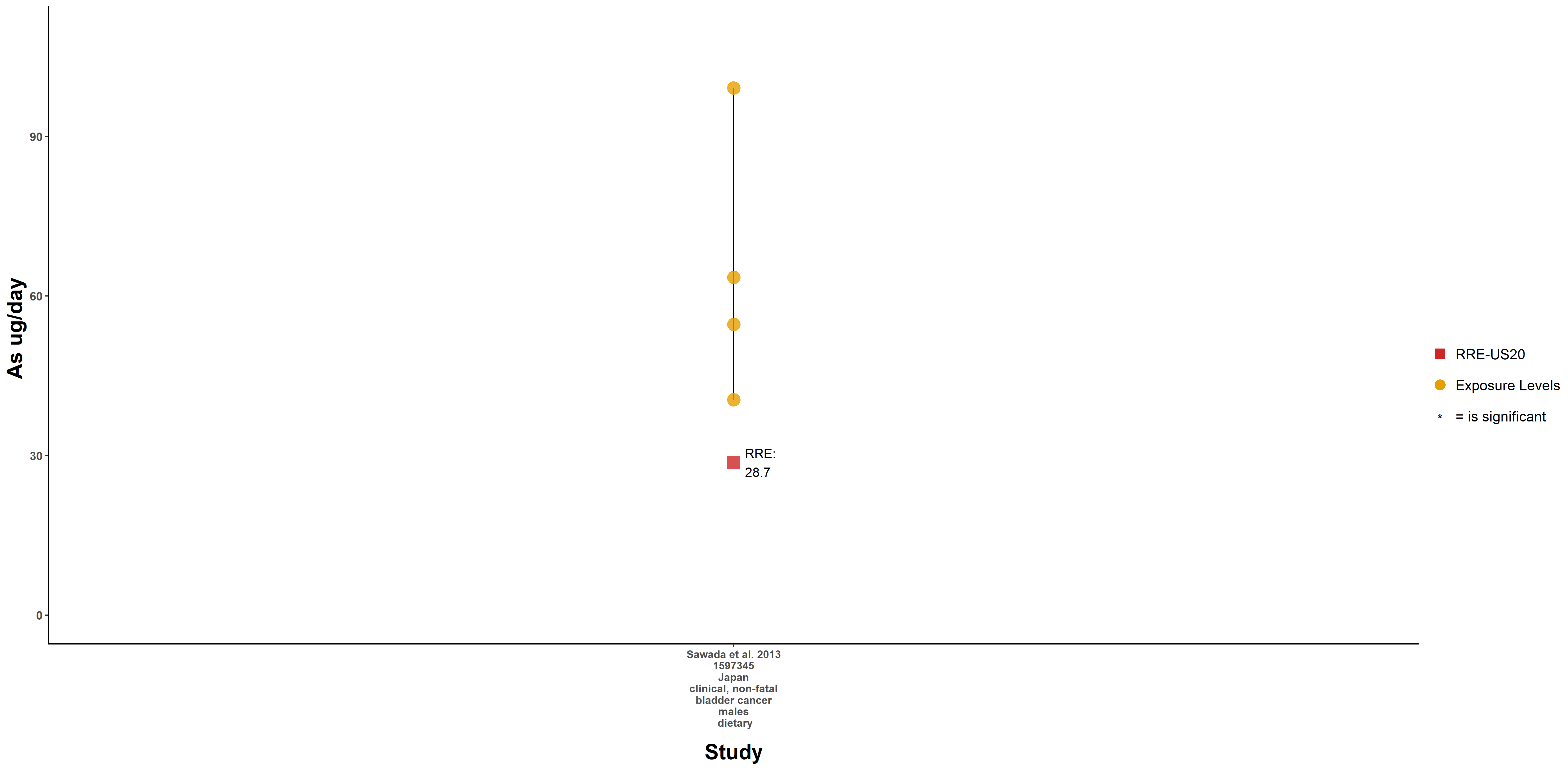


Figure S-4A. Exposure levels and RRE-US_20_ for bladder cancer using dietary intake.


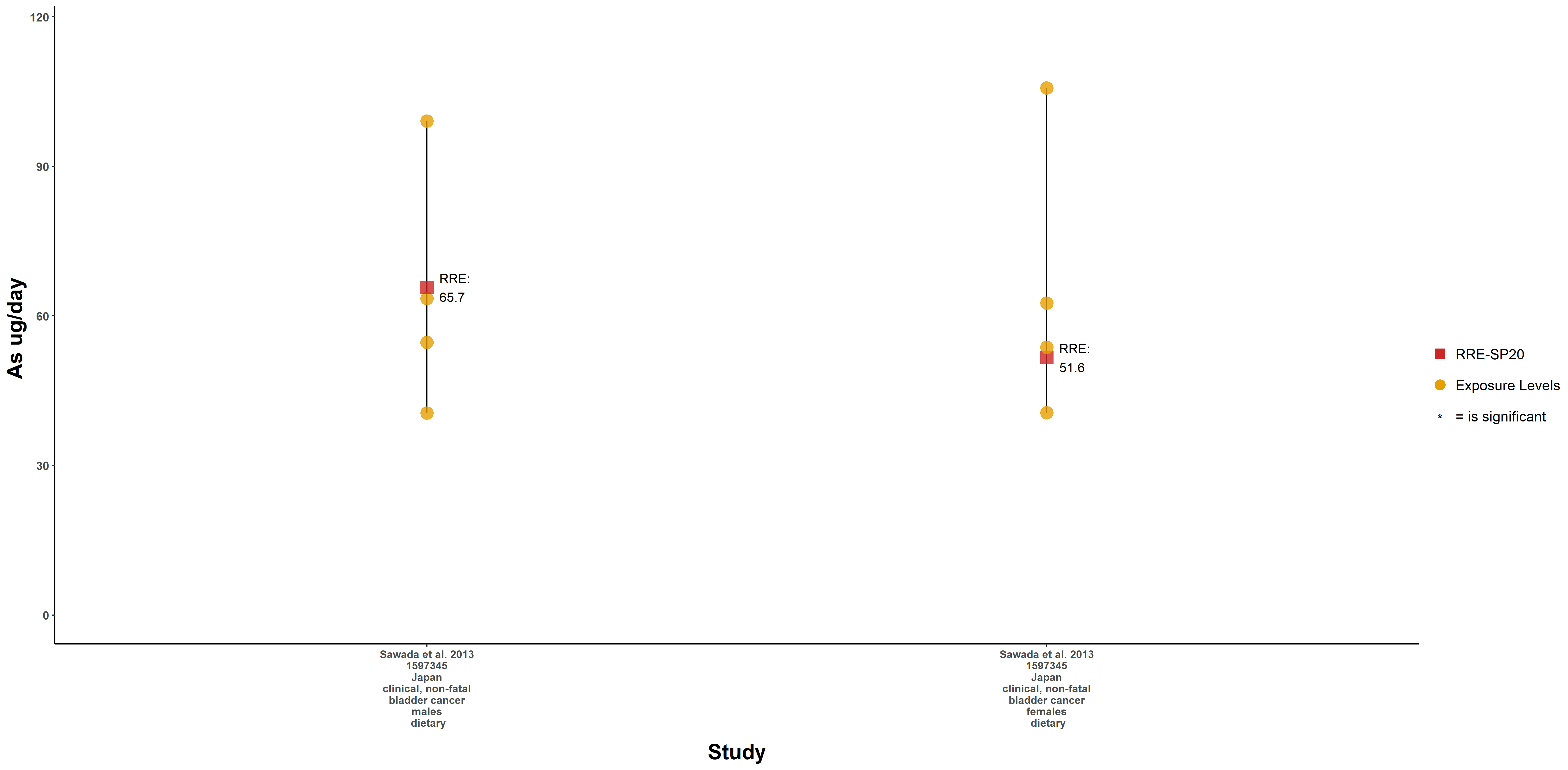


Figure S-4B. Exposure levels and RRE-SP_20_ for bladder cancer using dietary intake.


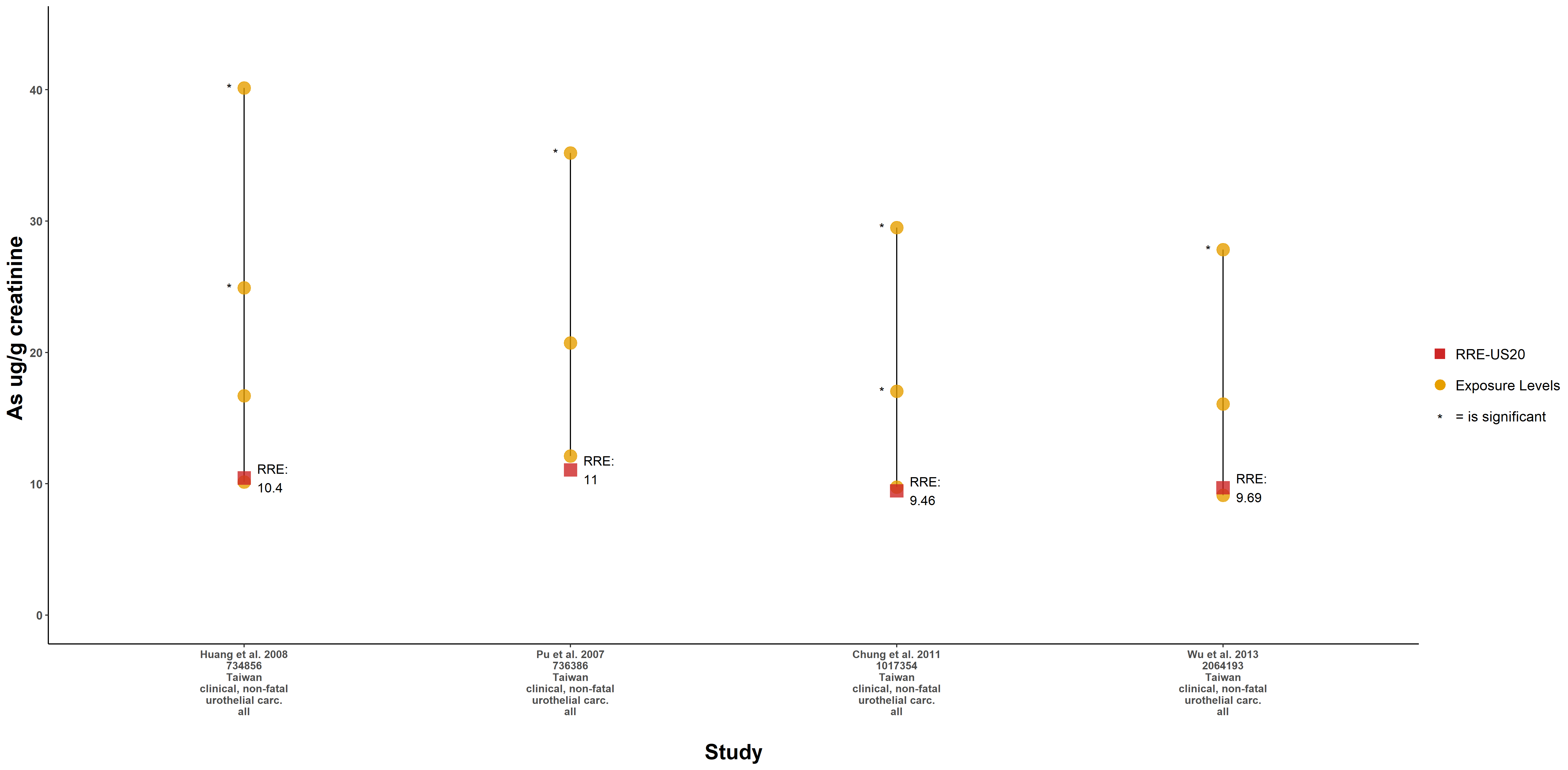


Figure S-5A. Exposure levels and RRE-US_20_ for bladder cancer using creatinine adjusted urine concentration.


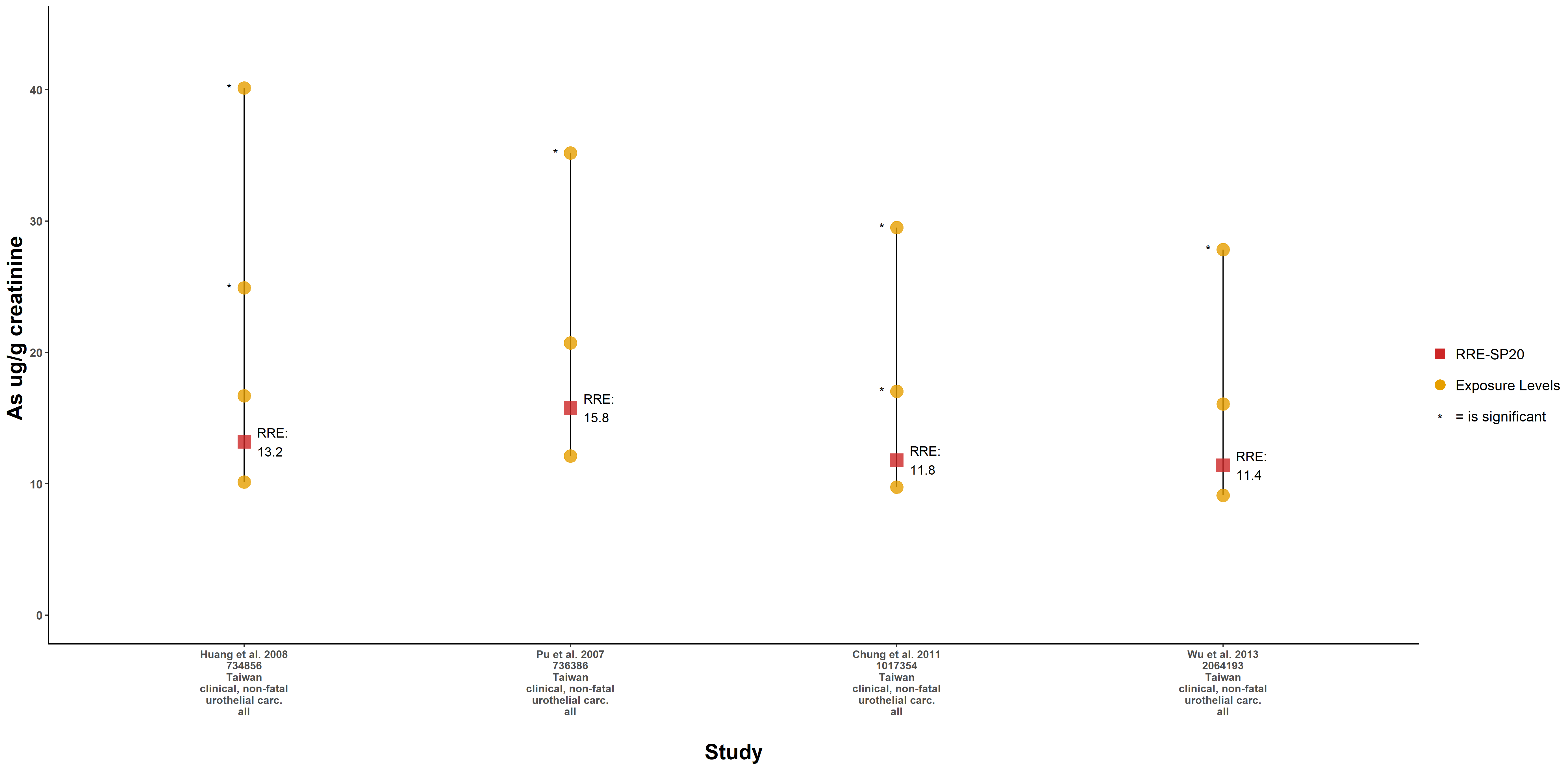


Figure S-5B. Exposure levels and RRE-SP_20_ for bladder cancer using creatinine adjusted urine concentration.


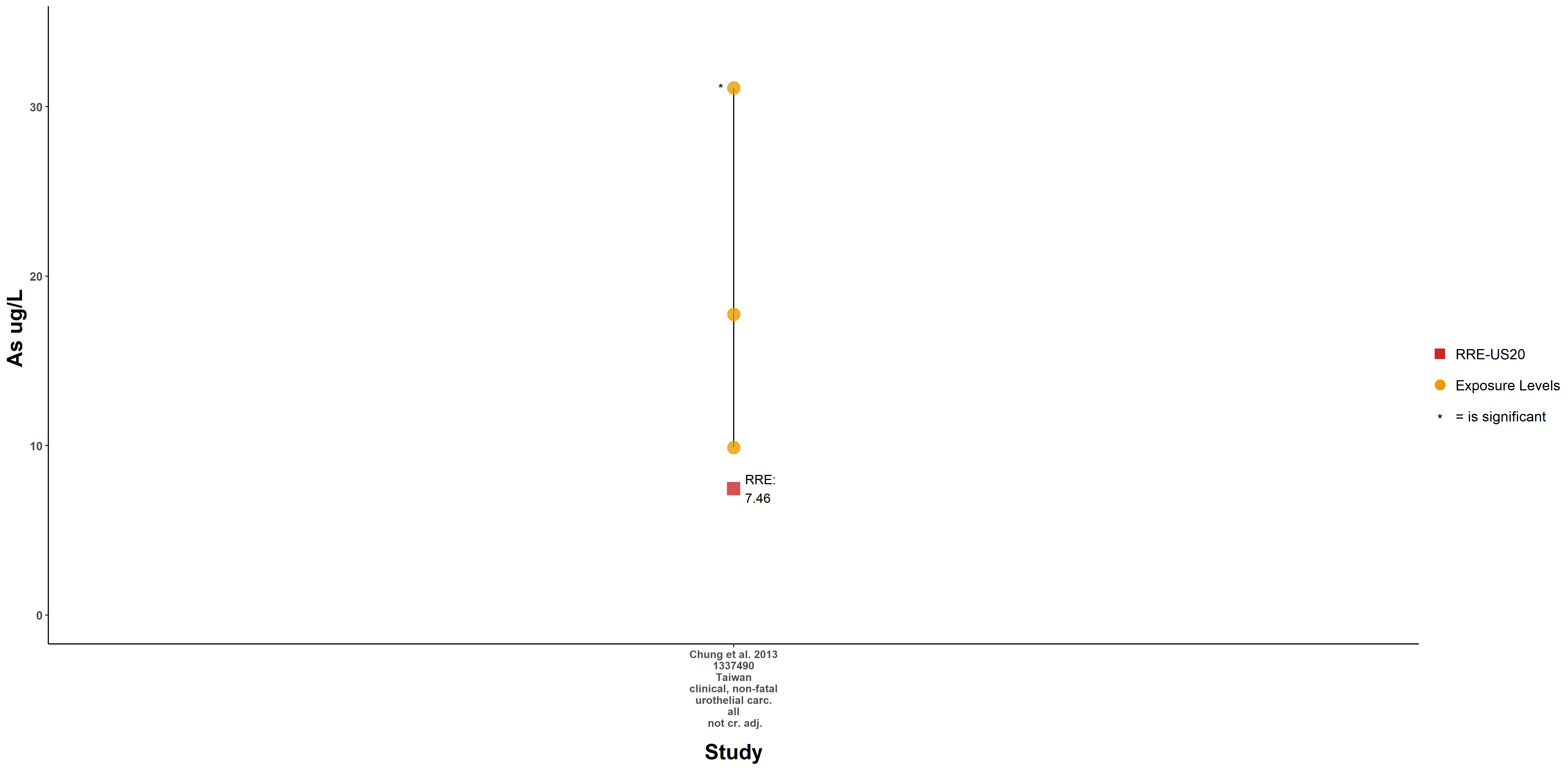


Figure S-6A. Exposure levels and RRE-US_20_ for bladder cancer using urine concentration.


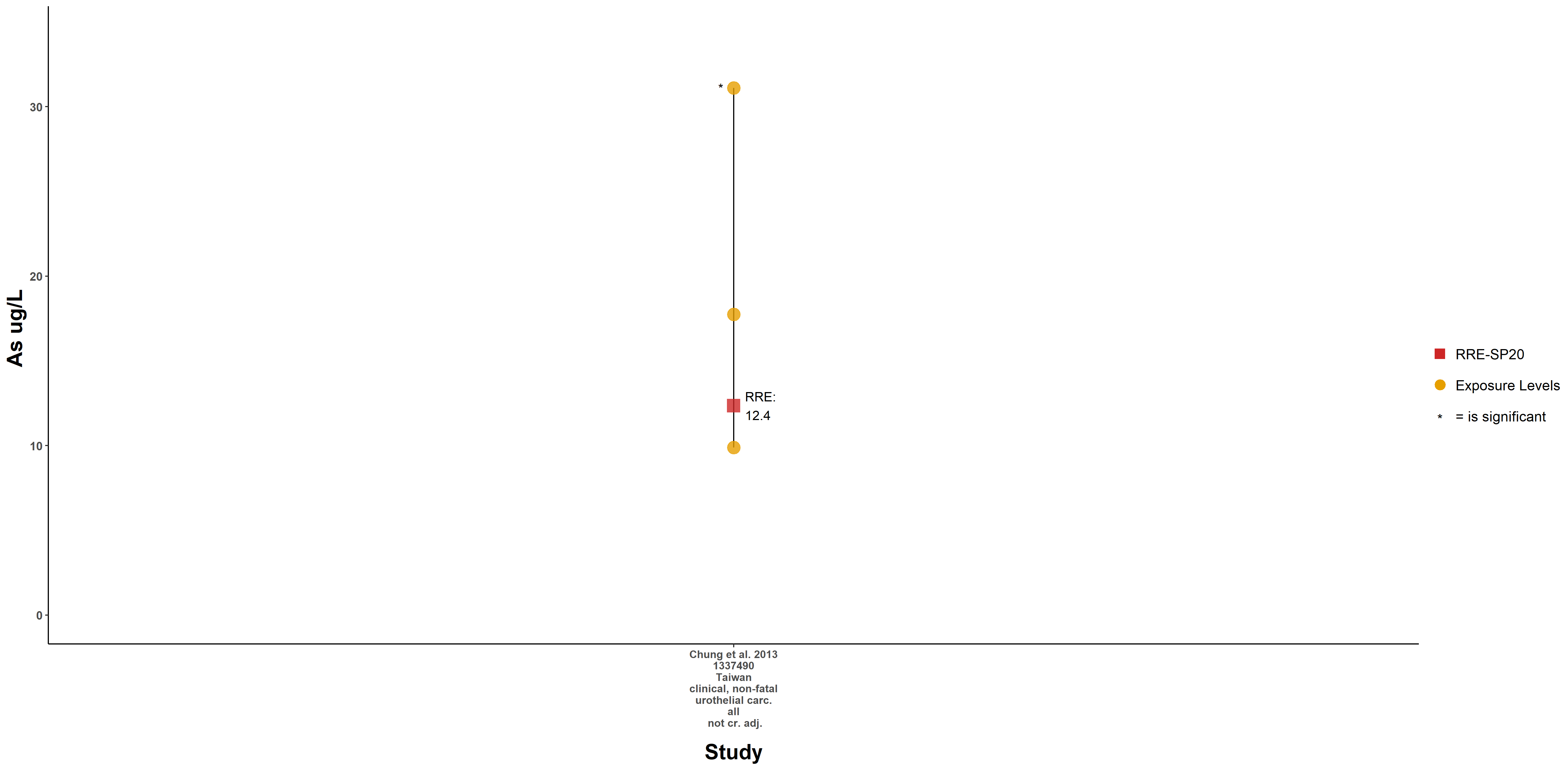


Figure S-6B. Exposure levels and RRE-SP_20_ for bladder cancer using urine concentration.


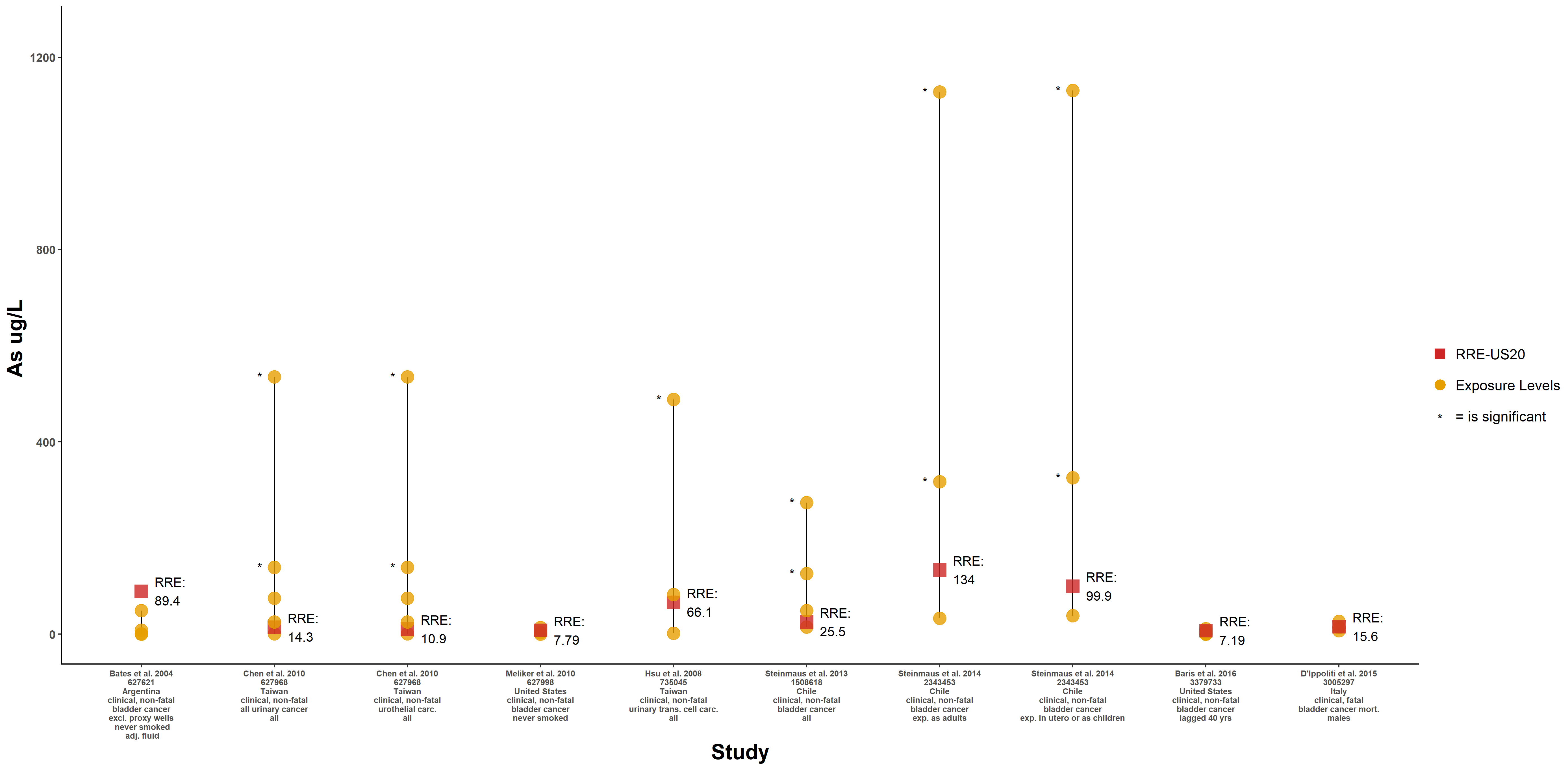


Figure S-7A. Exposure levels and RRE-US_20_ for bladder cancer using water concentration.


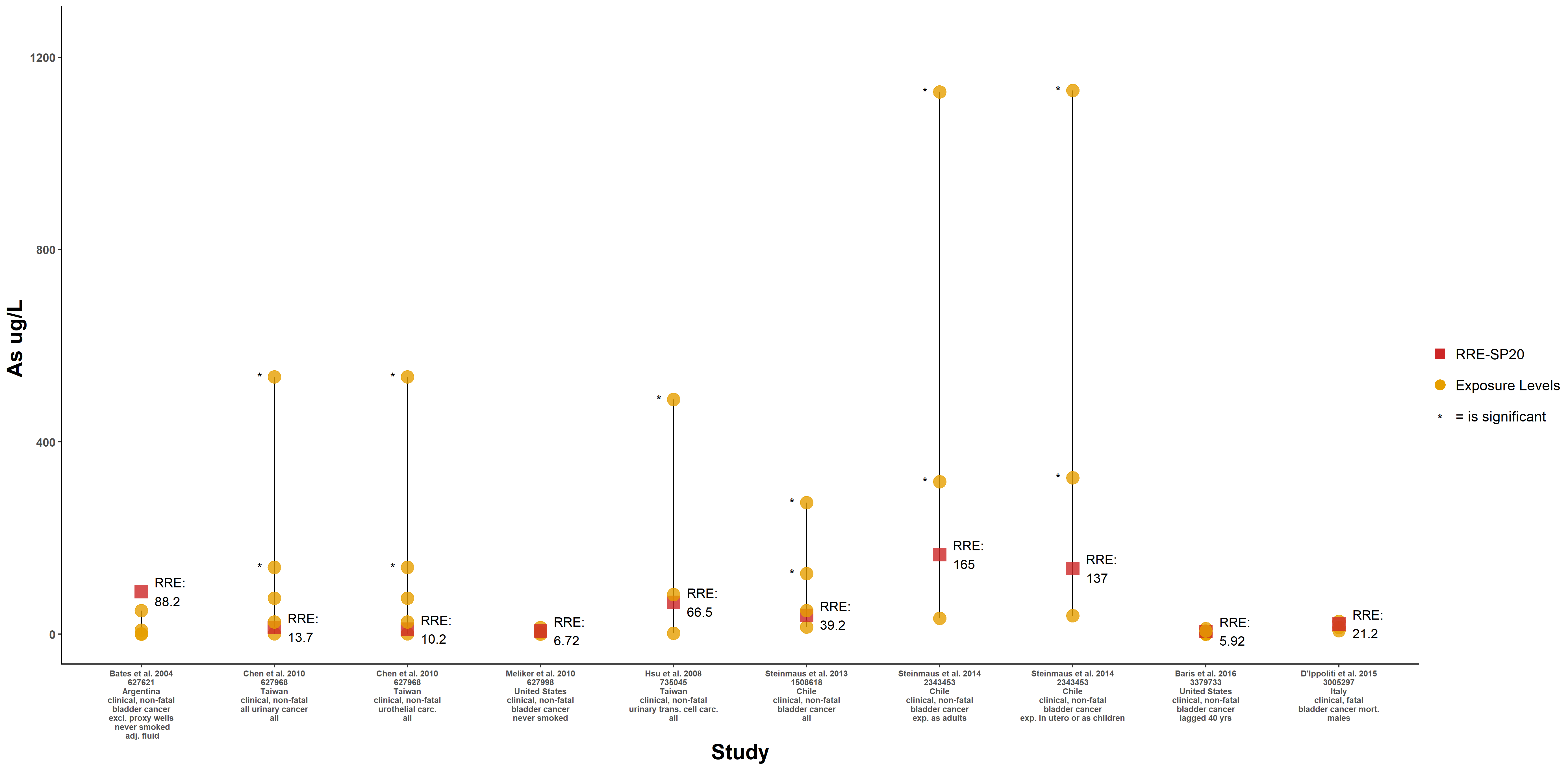


Figure S-7B. Exposure levels and RRE-SP_20_ for bladder cancer using water concentration.

Table S-26A. Summary of RRE-US_20_ and RRB-US for bladder cancer studies


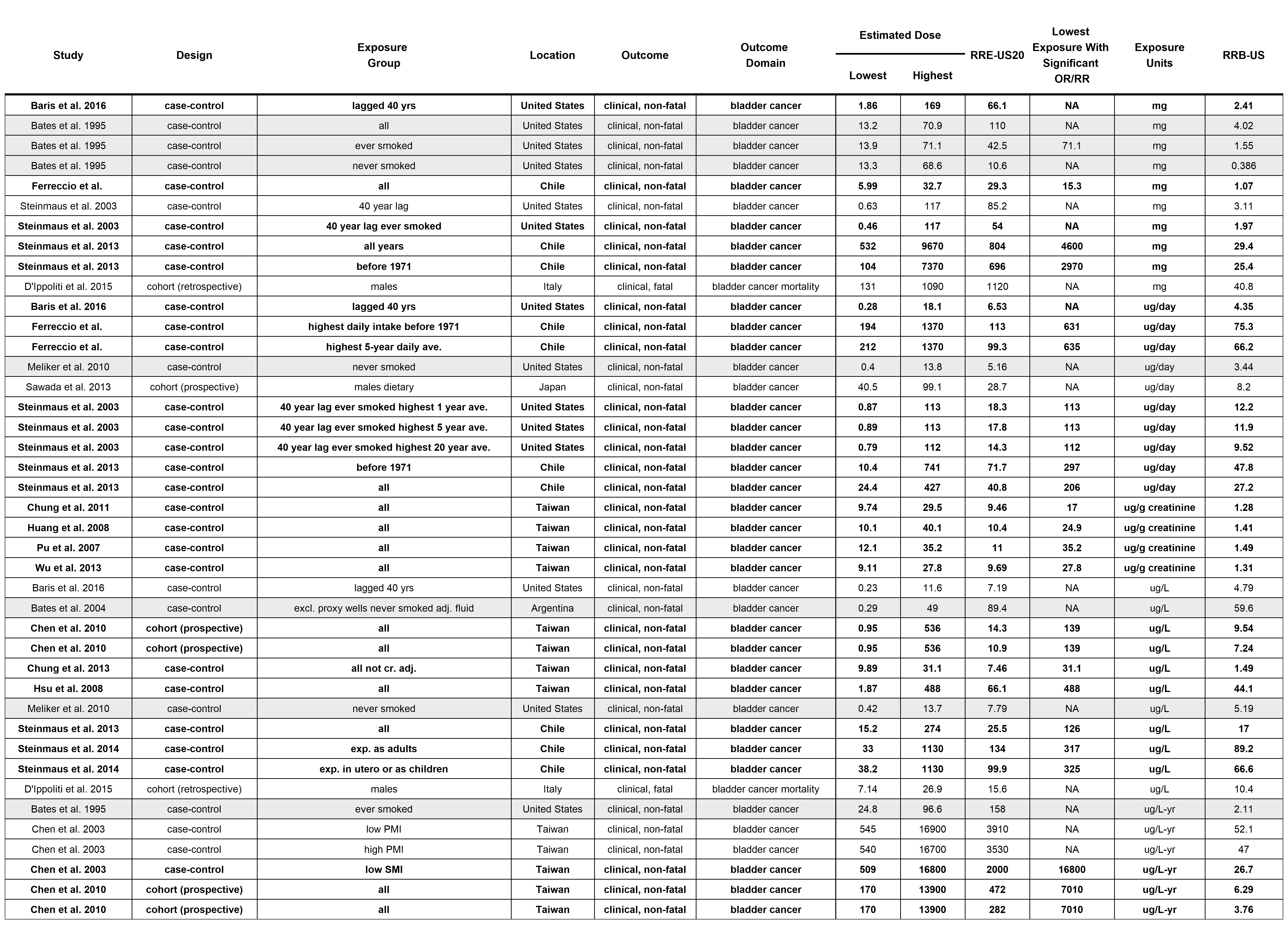


RRB-US refers to the ratio of RRE-US_20_ to an estimated U.S. background exposure level. Shaded cells indicate that authors did not report exposure-response trends. Bold rows indicate that authors reported a significant exposure-response trend (*p* <0.05). Bates et al 1995, never smoked dataset was dropped from RRB analysis due to a non-positive dose response.

Table S-26B. Summary of RRE-SP_20_ and RRB-SP for bladder cancer studies


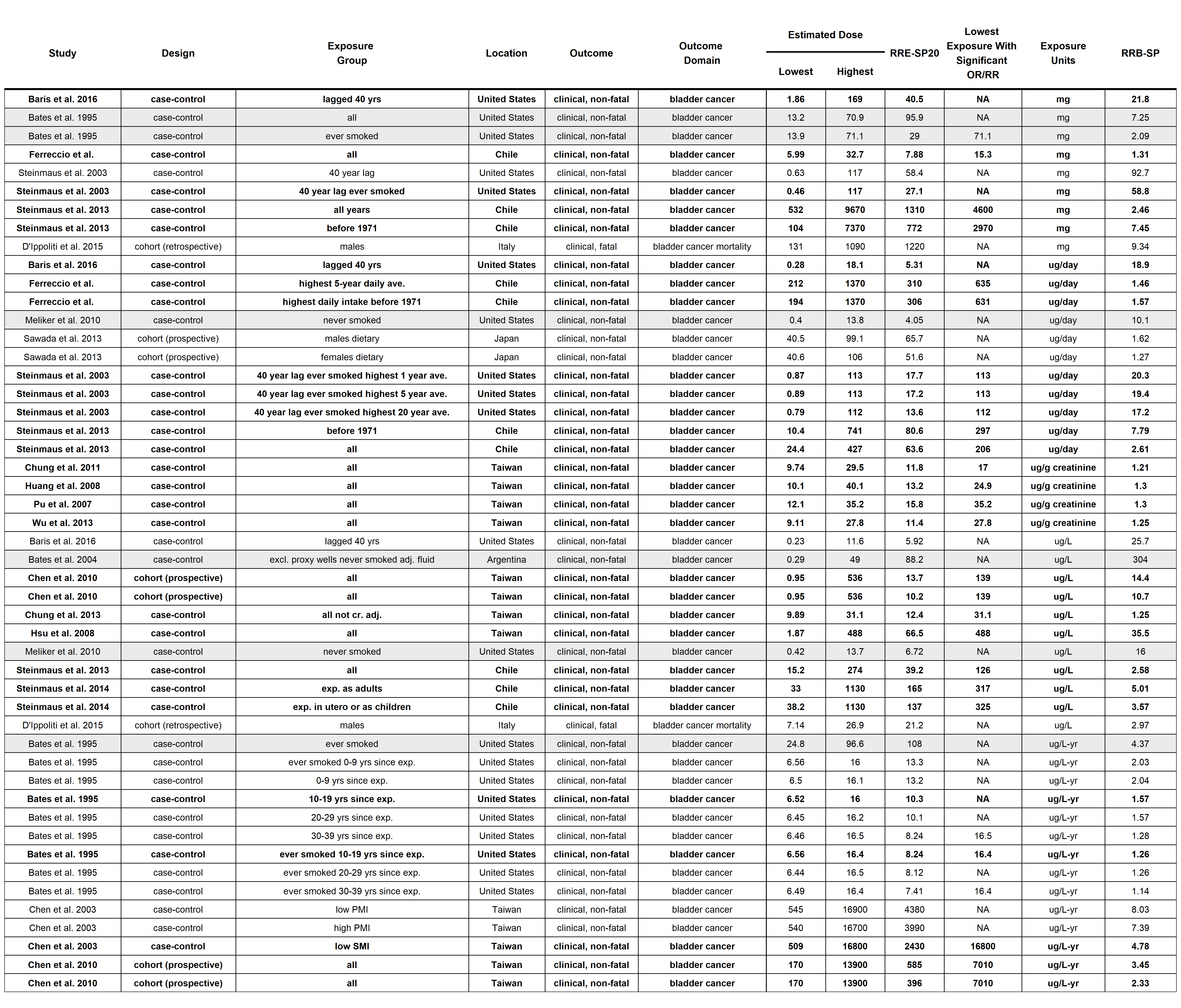


RRB-SP refers to the ratio of RRE-SP_20_ to the reported or estimated background exposure level for the study referent group. Shaded cells indicate that authors did not report exposure-response trends. Bold rows indicate that authors reported a significant exposure-response trend (*p* <0.05)

#### Diabetes Exposure-Response Modeling Results

The analysis of arsenic exposure response in diabetes outcomes consisted of 9 datasets from 4 peer reviewed studies. A summary of datasets modeled in identifying the study design, location, exposure metric and outcome domain is provided in Table S-27 below. A breakdown of the exposure levels and RRE_20_ estimates are provided for each exposure metric in Figure S-8–Figure S-11. Finally, RRE_20_ summary tables for all exposures are provided in Table S-28.

Table S-27. Summary of datasets considered in diabetes exposure-response RRB analysis by exposure metric


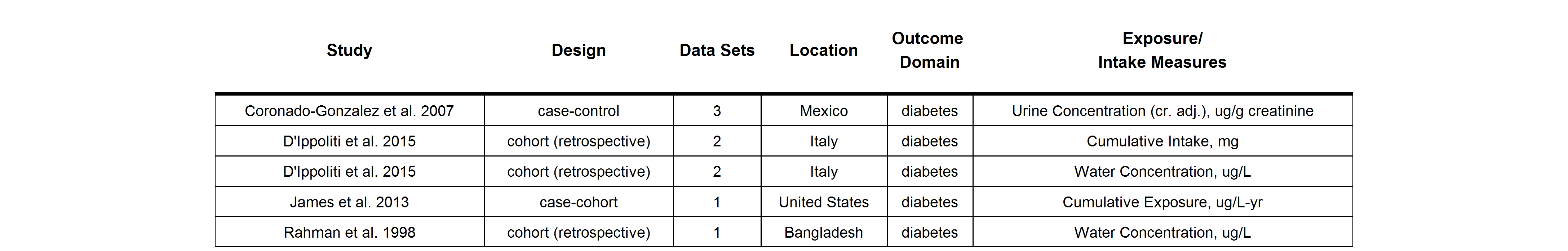


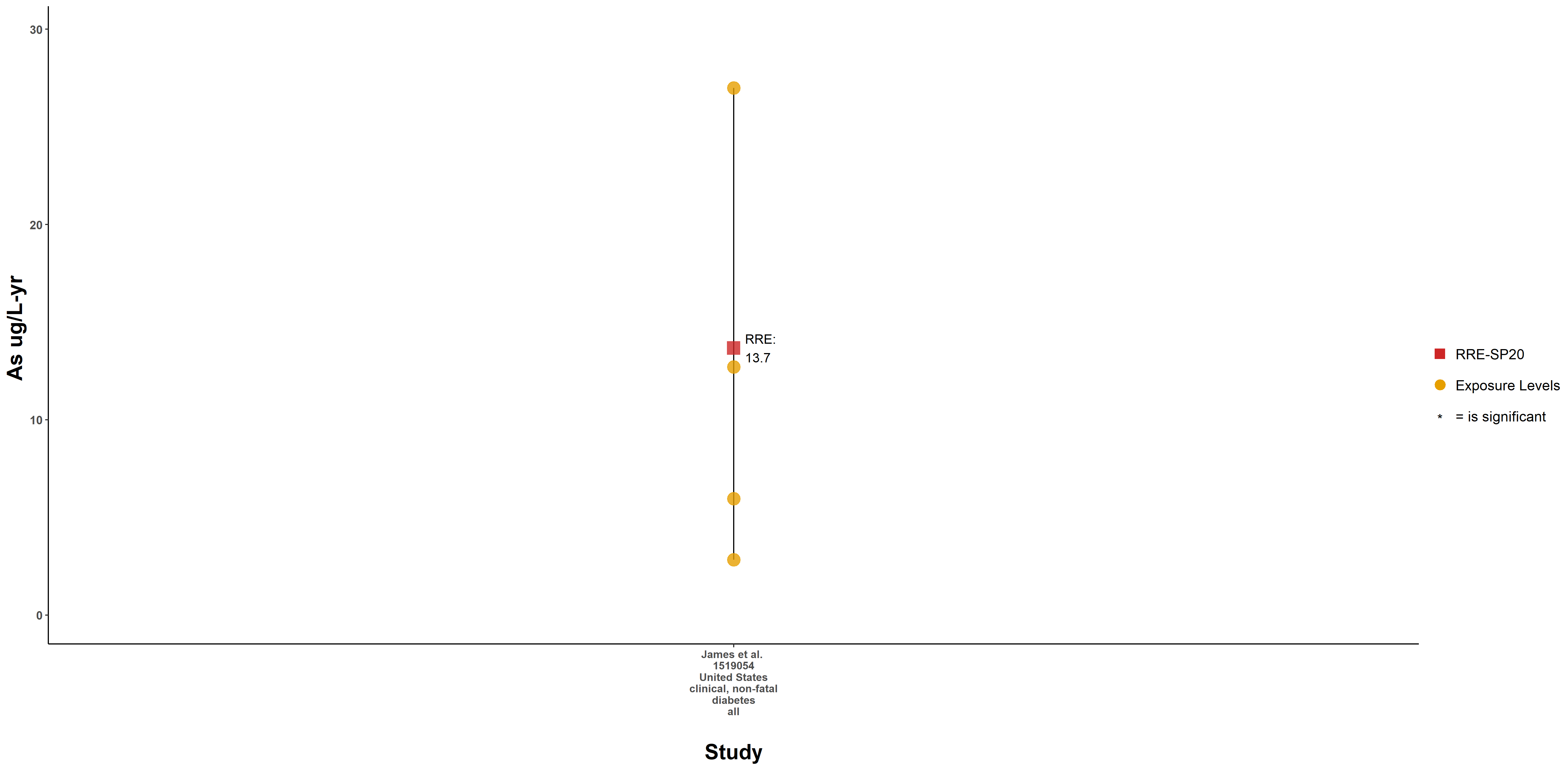


Figure S-8. Exposure levels and RRE-SP_20_ for diabetes using cumulative exposure.


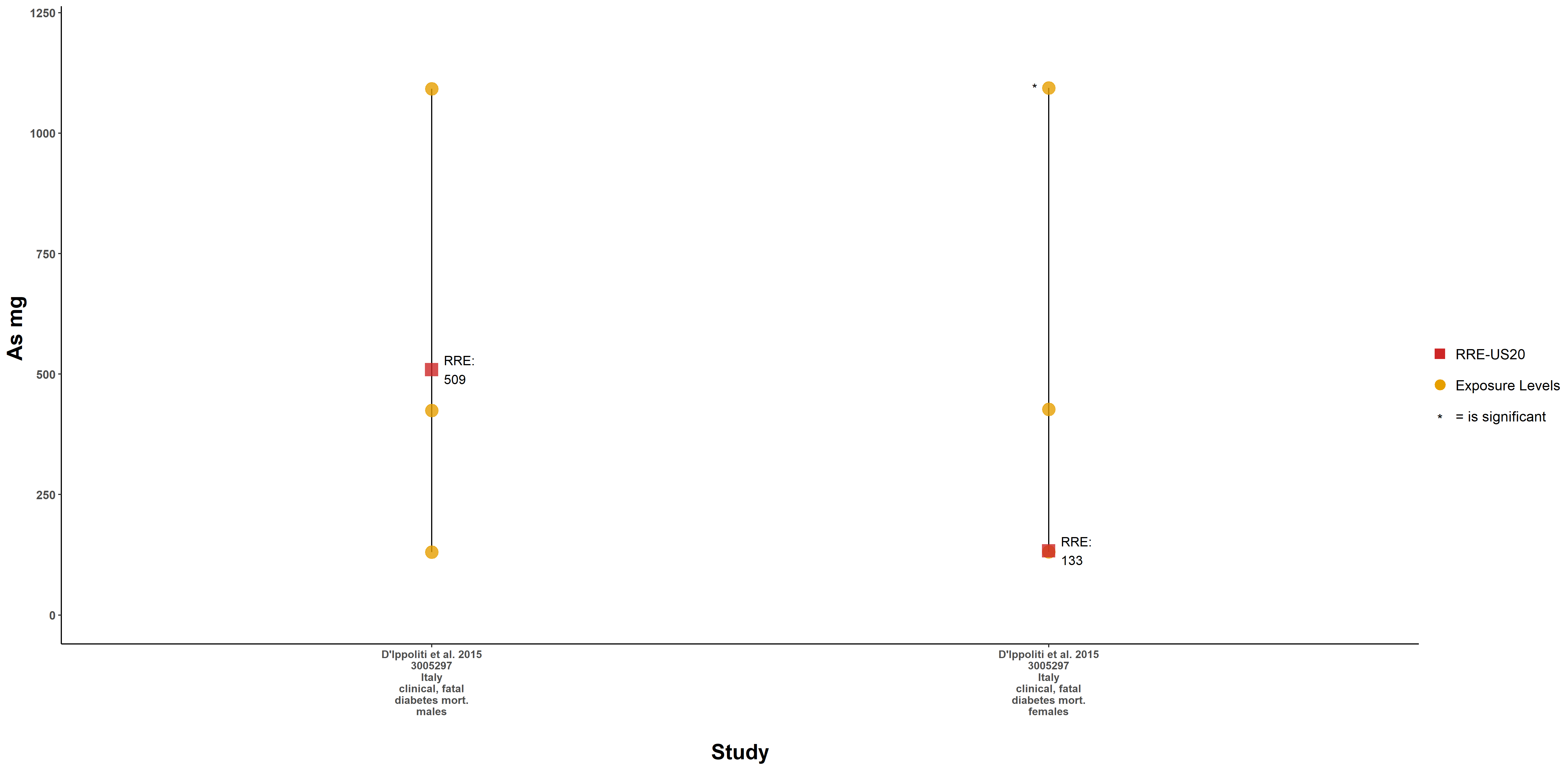


Figure S-9A. Exposure levels and RRE-US_20_ for diabetes using cumulative intake.


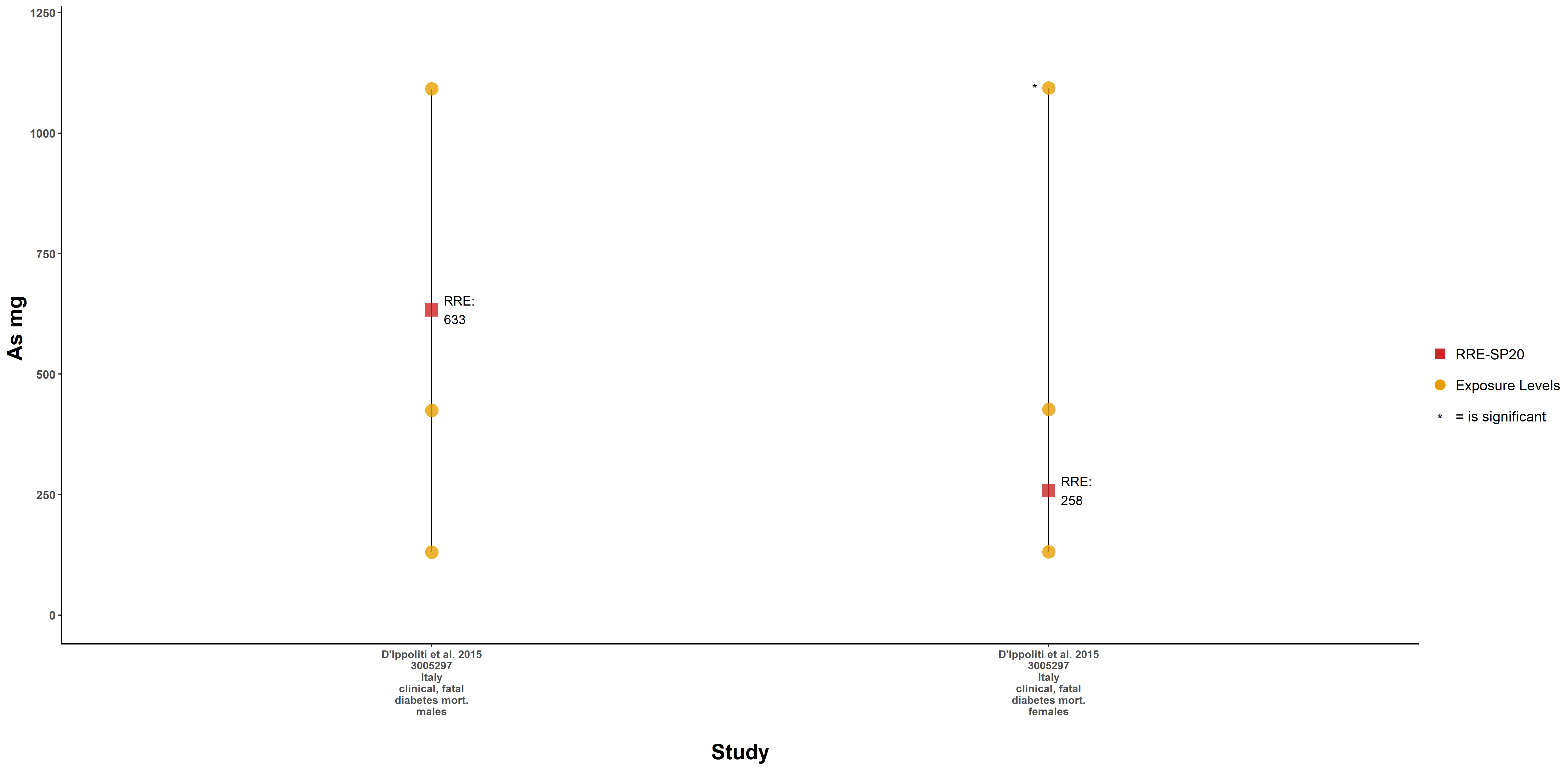


Figure S-9B. Exposure levels and RRE-SP_20_ for diabetes using cumulative intake.


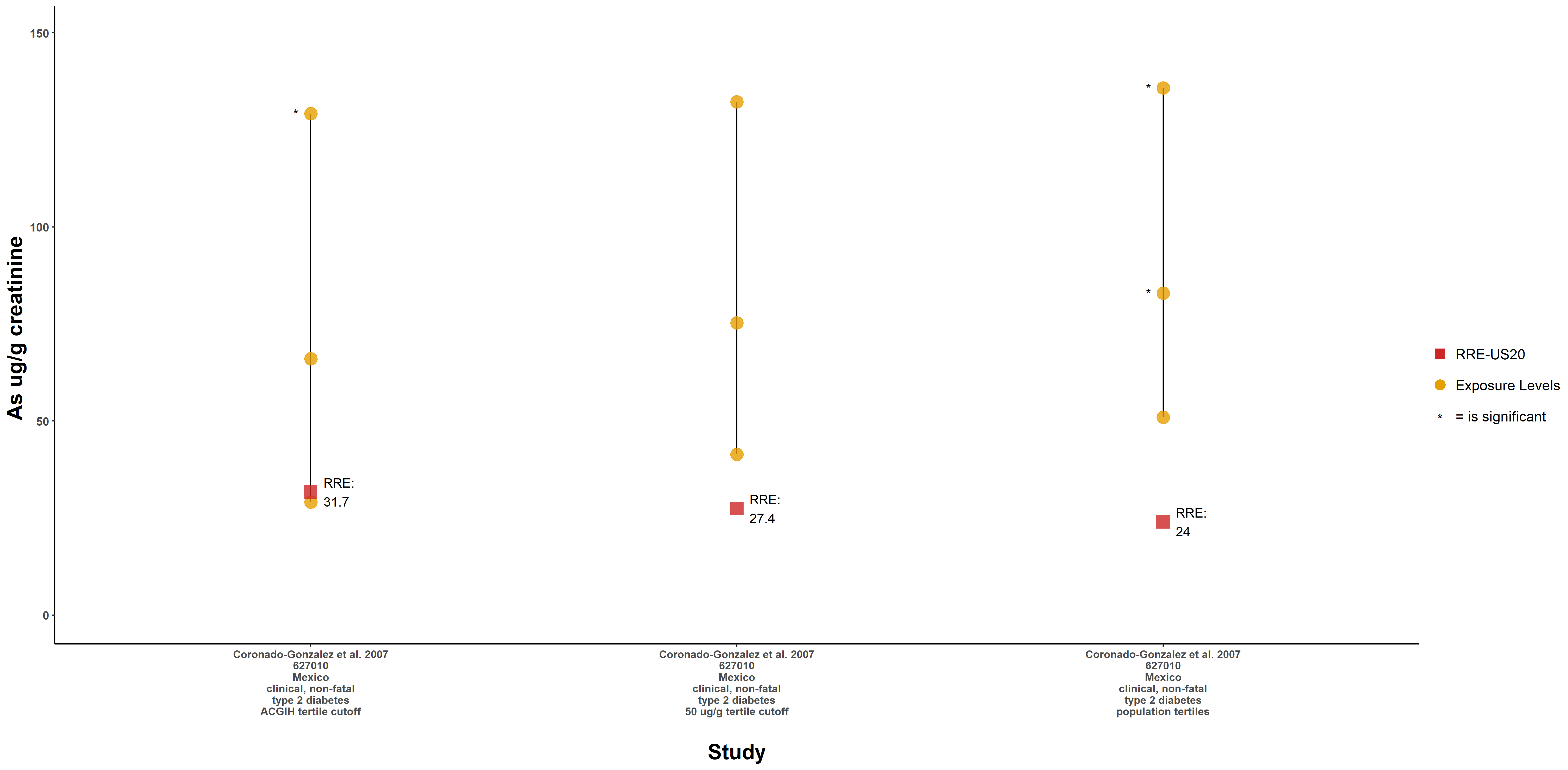


Figure S-10A. Exposure levels and RRE-US_20_ for diabetes using creatinine adjusted urine concentration.


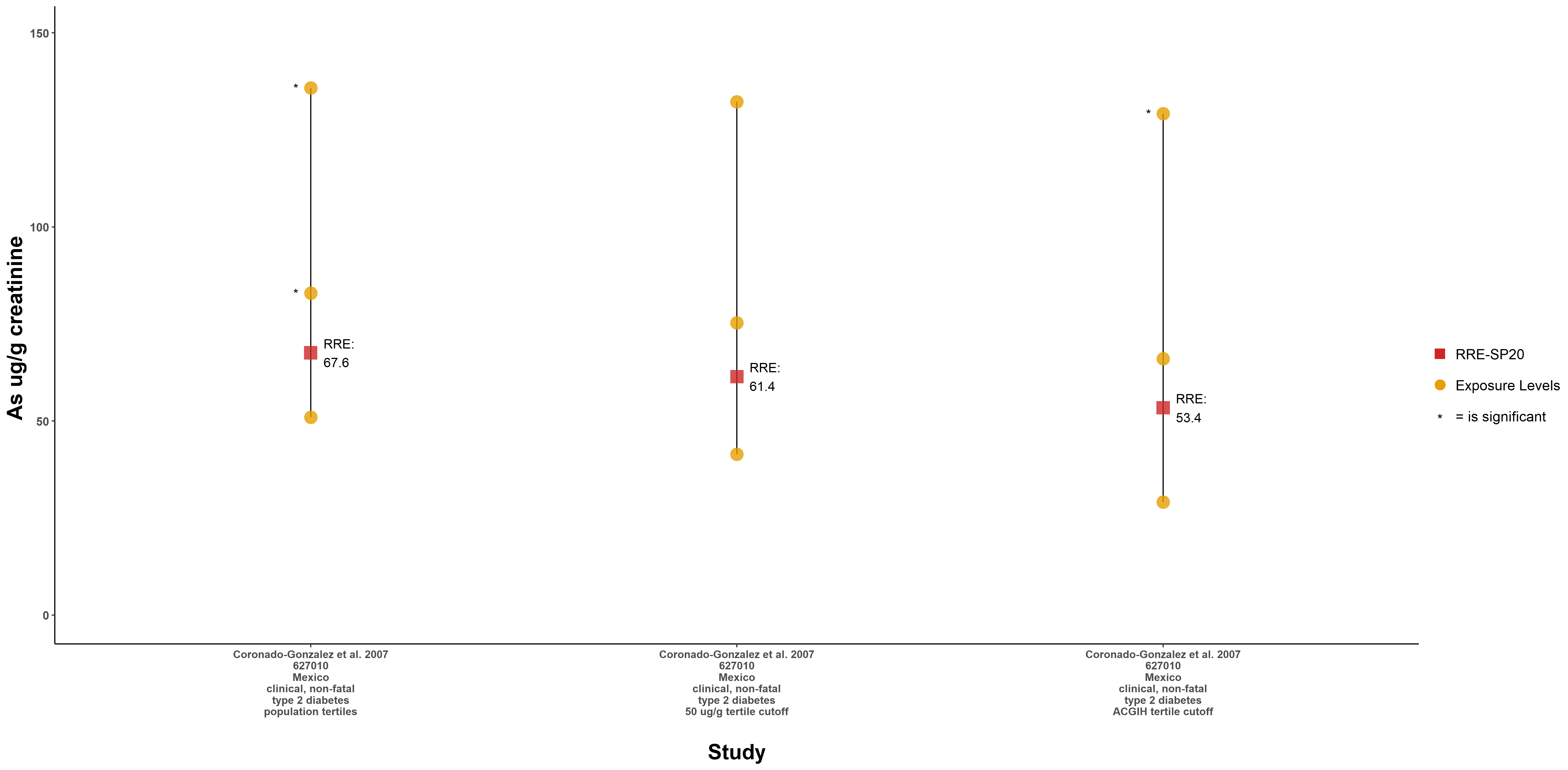


Figure S-10B. Exposure levels and RRE-SP_20_ for diabetes using creatinine adjusted urine concentration.


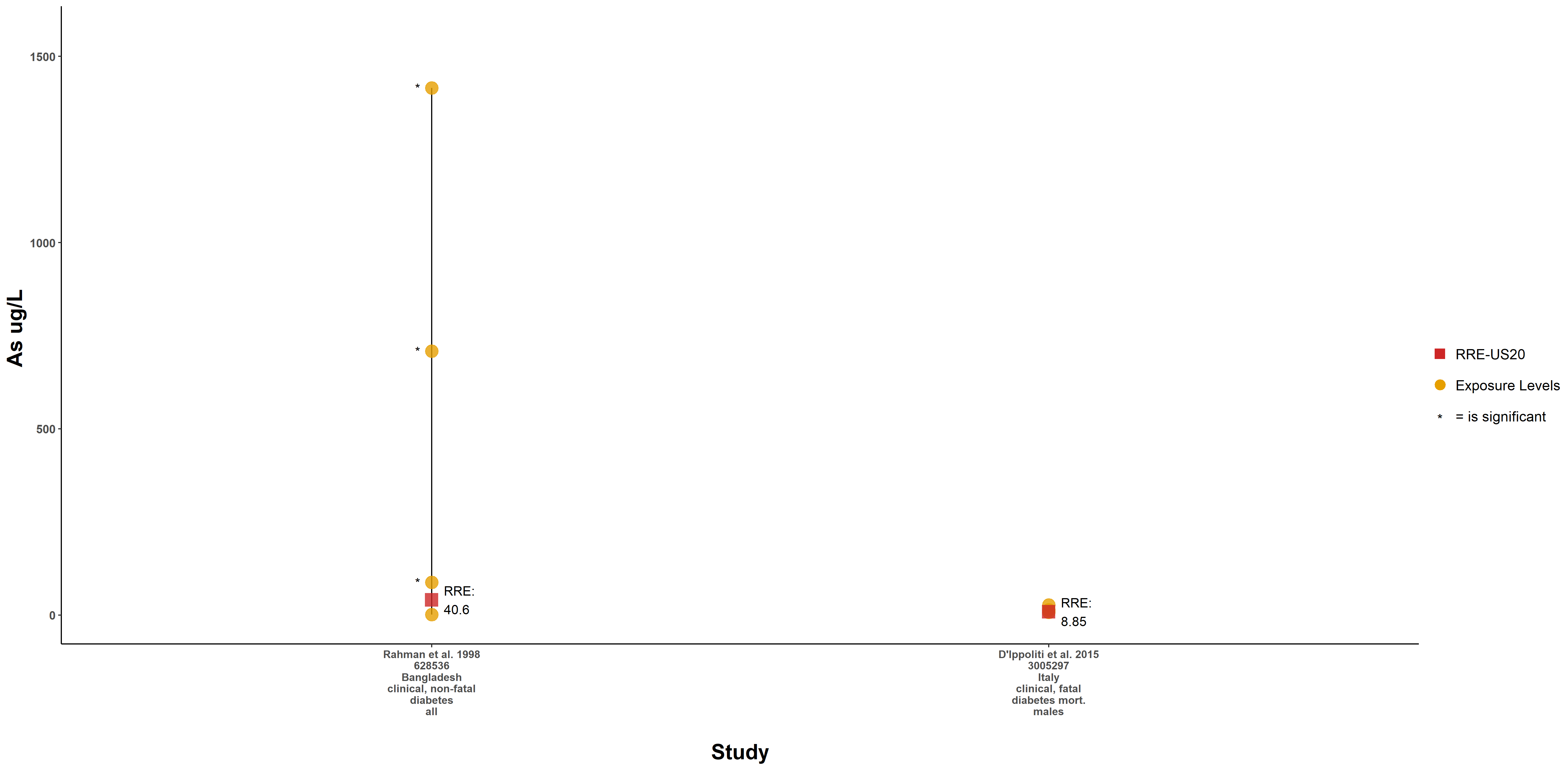


Figure S-11A. Exposure levels and RRE-US_20_ for diabetes using water concentration.


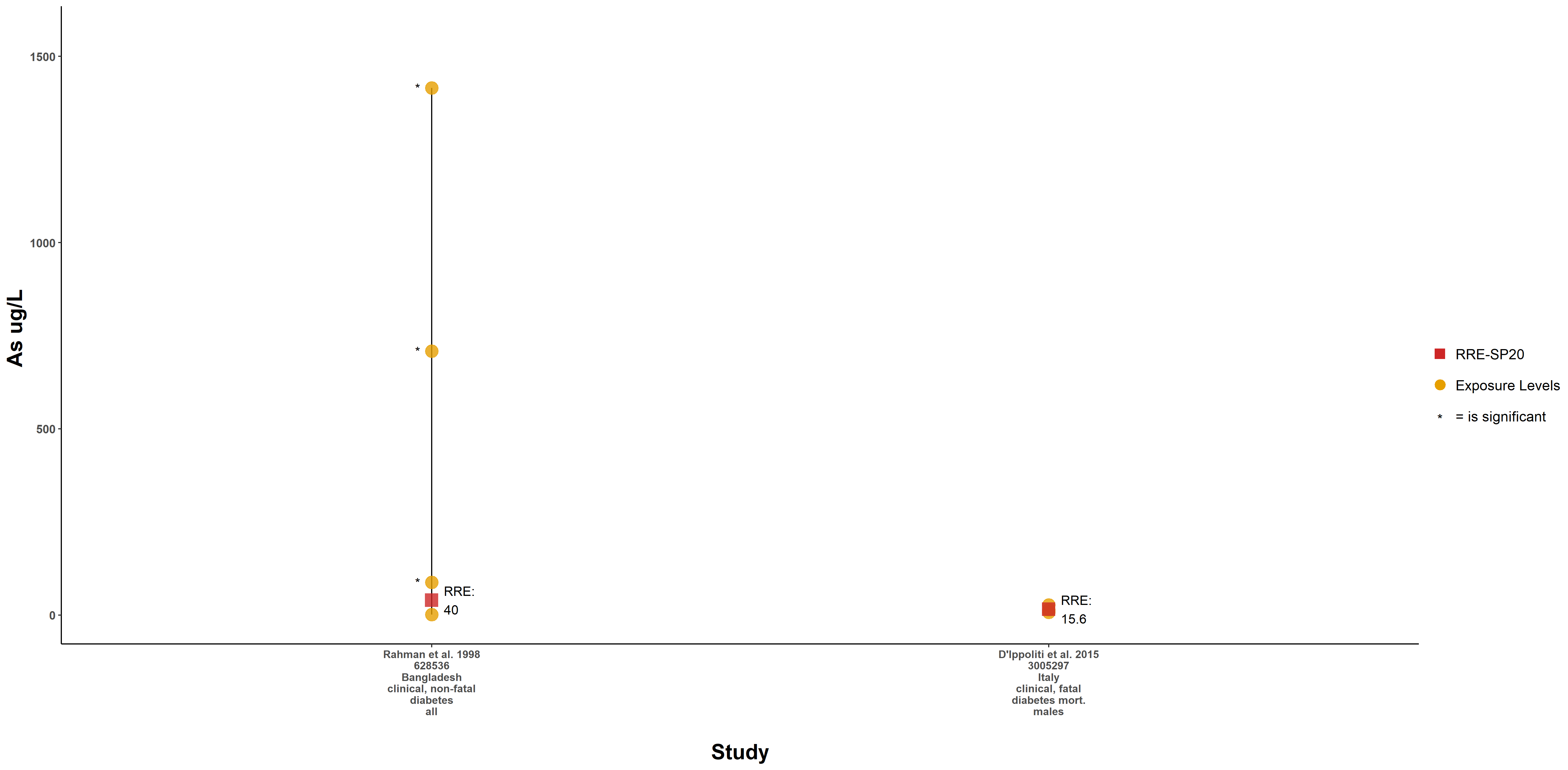


Figure S-11B. Exposure levels and RRE-SP_20_ for diabetes using water concentration.


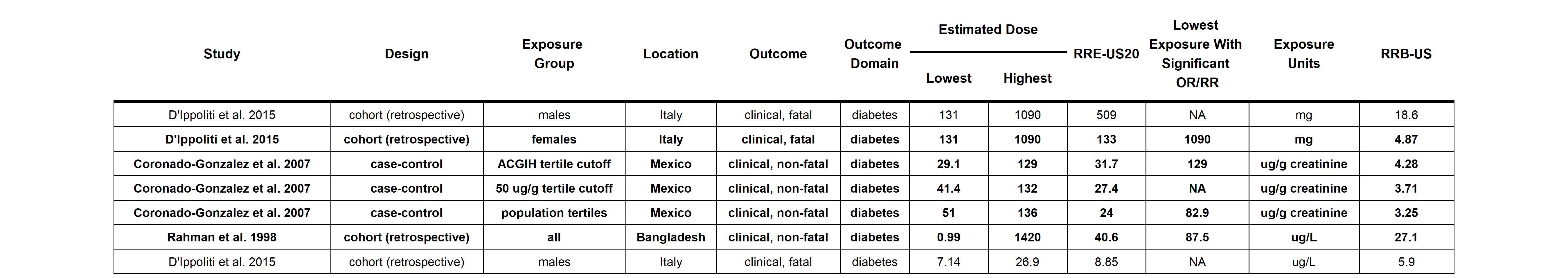
Table S-28A. Summary of RRE-US_20_s and RRB-US for diabetes studies

RRB-US refers to the ratio of RRE-US_20_ to an estimated U.S. background exposure level. Shaded cells indicate that authors did not report exposure-response trends. Bold rows indicate that authors reported a significant exposure-response trend (*p* <0.05)

Table S-28B. Summary of RRE-SP_20_s and RRB-SP for diabetes studies


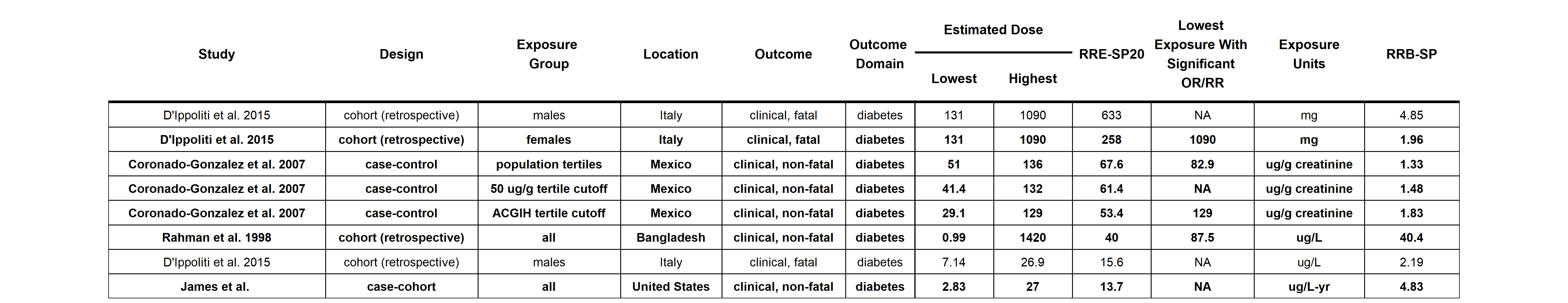


RRB-SP refers to the ratio of RRE-SP_20_ to the reported or estimated background exposure level for the study referent group. Shaded cells indicate that authors did not report exposure-response trends. Bold rows indicate that authors reported a significant exposure-response trend (*p* <0.05)

#### Disease of the Circulatory System Exposure-Response Modeling Results

The analysis of arsenic exposure response on diseases of the circulatory system evaluated 69 datasets from 14 peer reviewed studies that included endpoints such as hypertension, atherosclerosis, and stroke. A summary of datasets modeled identifying the study design, location, exposure metric and outcome domain are provided in Table S-29 below. A breakdown of the exposure levels and RRE_20_ estimates are provided for each exposure metric in Figure S-12–Figure S-17. Finally, RRE_20_ summary tables for all exposures are provided in Table S-30.

Table S-29. Summary of datasets considered in diseases of the circulatory system exposure-response RRB by exposure metric


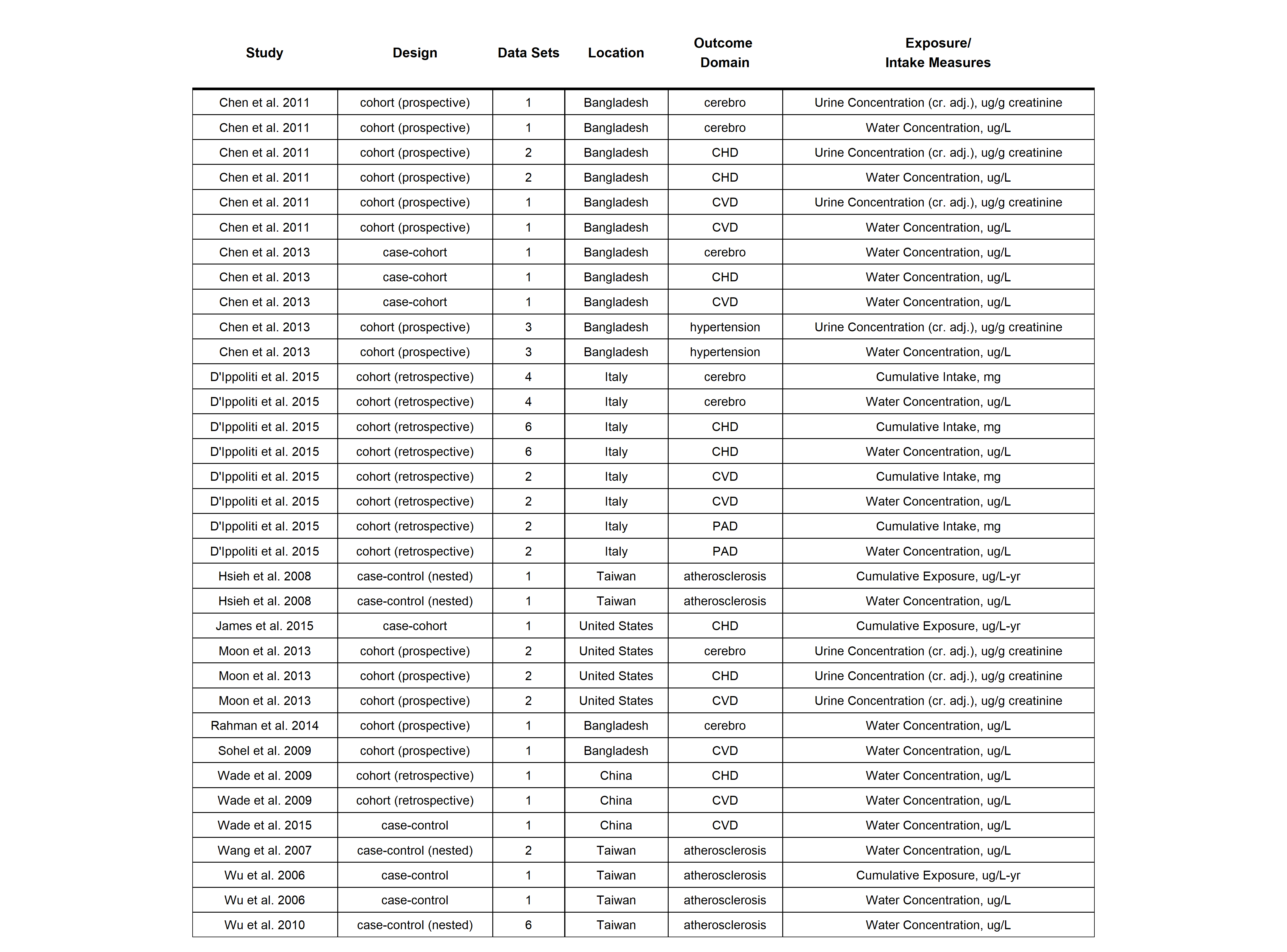


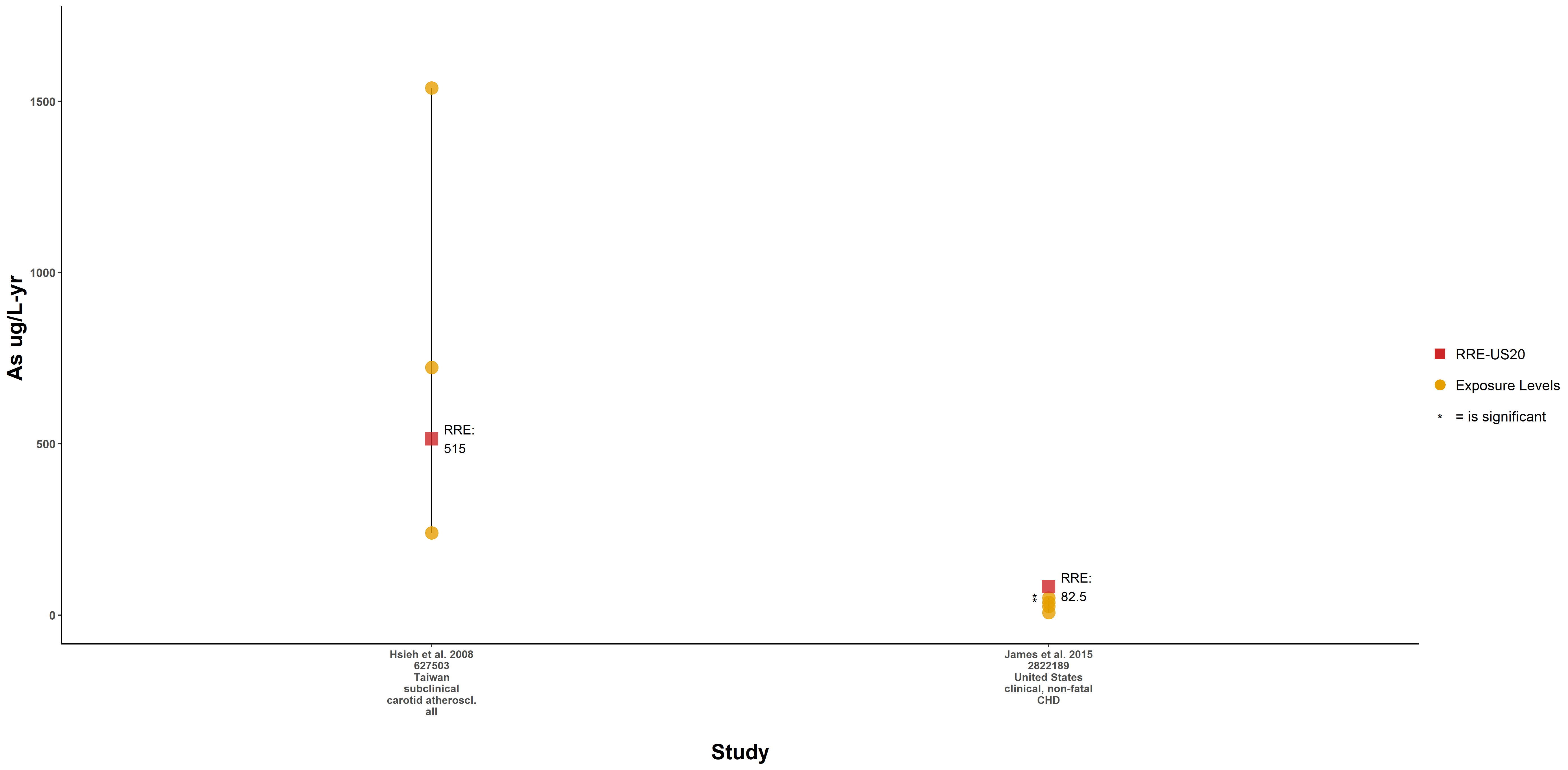


Figure S-12A. Exposure levels and RRE-US_20_ for diseases of the circulatory system using cumulative exposure.


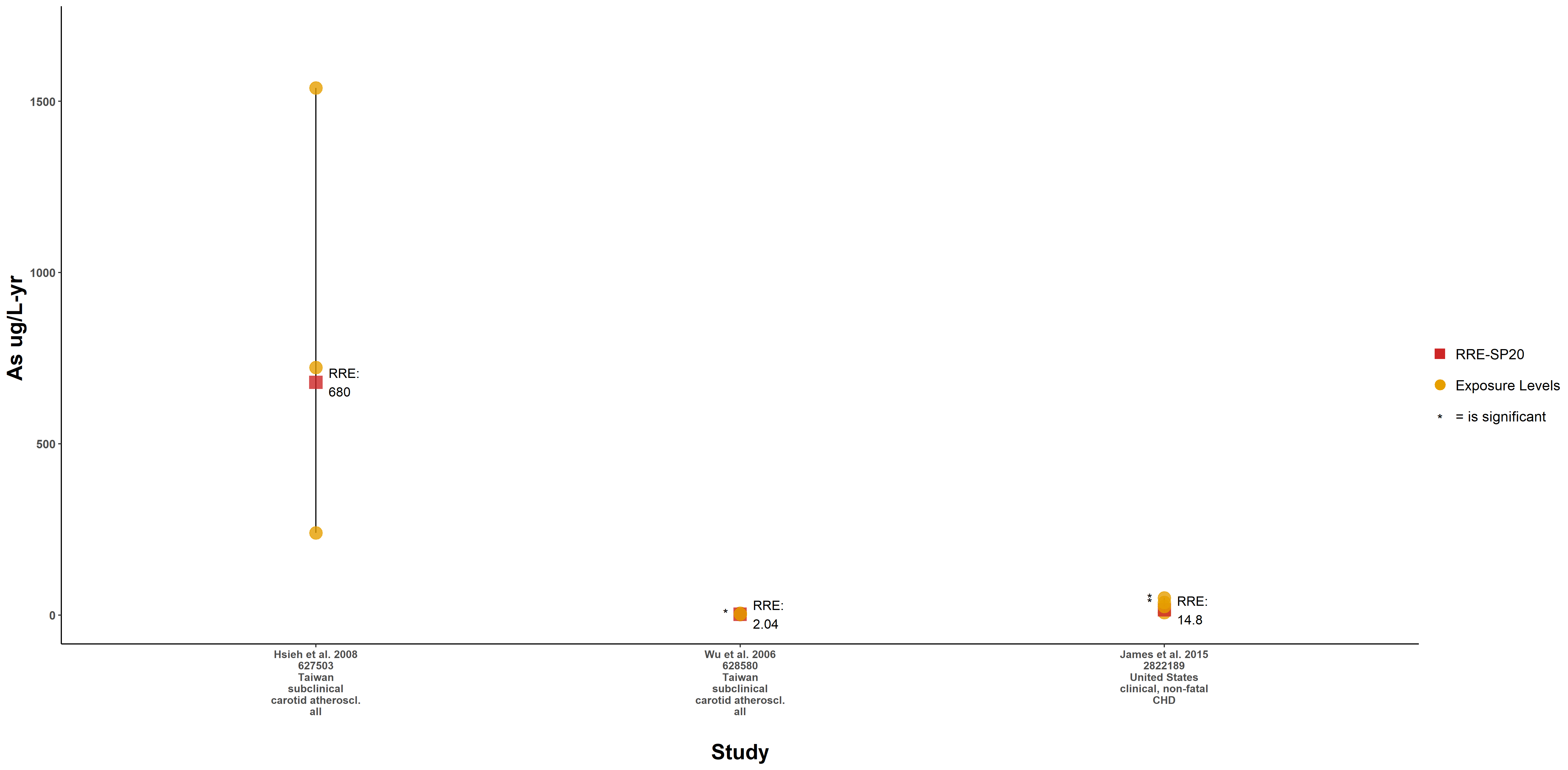


Figure S-12B. Exposure levels and RRE-SP_20_ for diseases of the circulatory system using cumulative exposure.


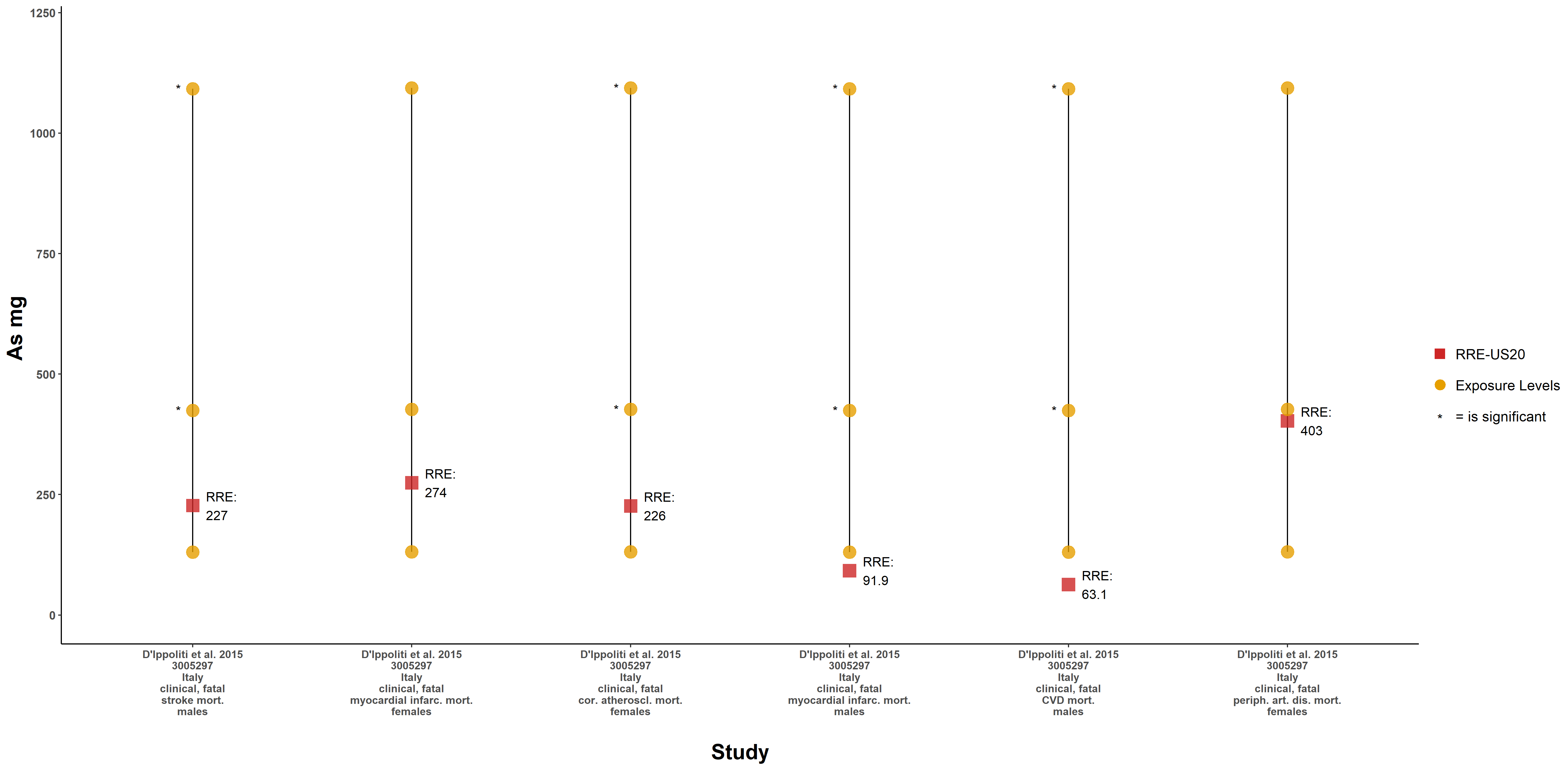


Figure S-13A. Exposure levels and RRE-US_20_ for diseases of the circulatory system using cumulative intake.


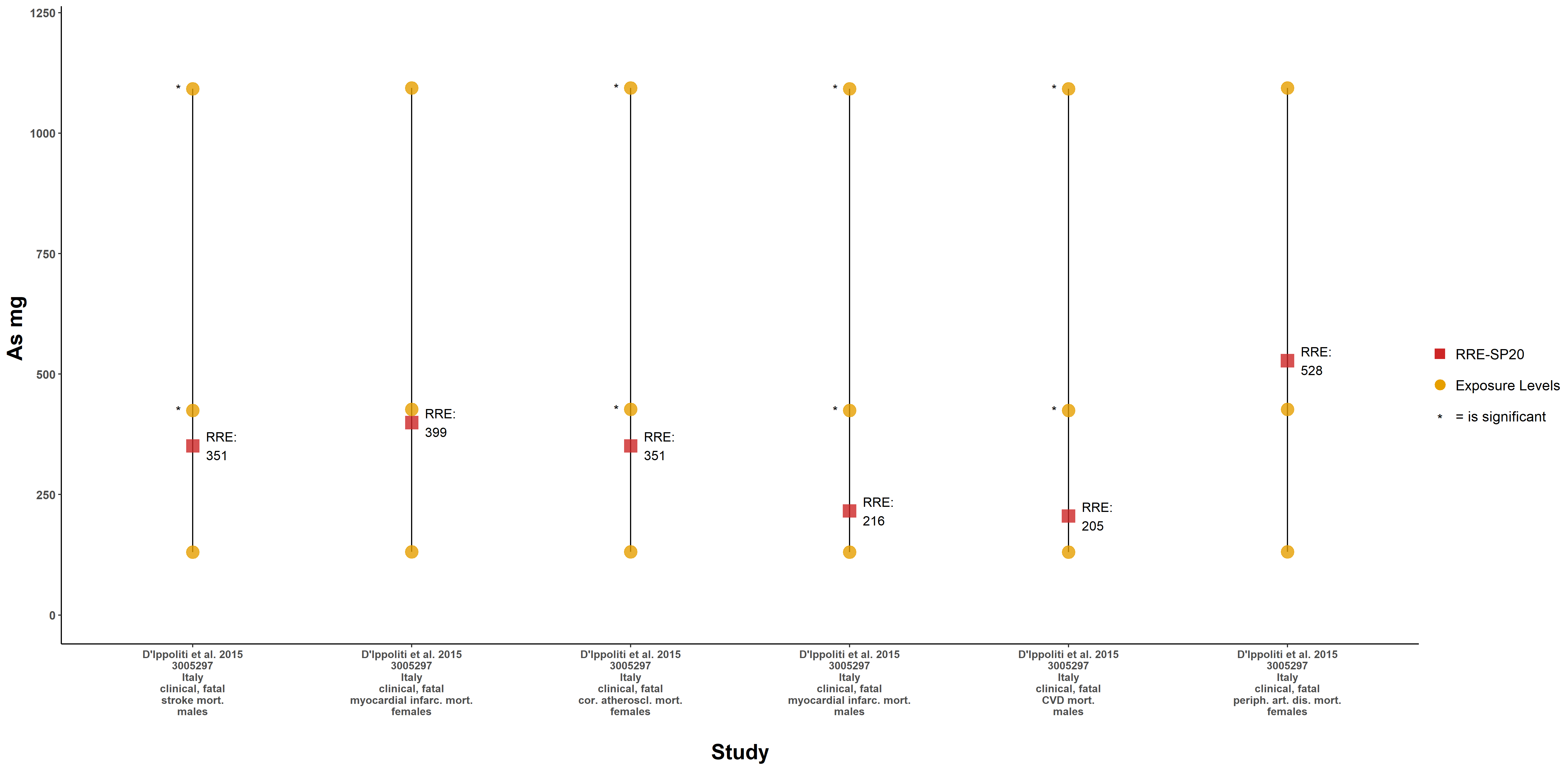


Figure S-13B. Exposure levels and RRE-SP_20_ for diseases of the circulatory system using cumulative intake.


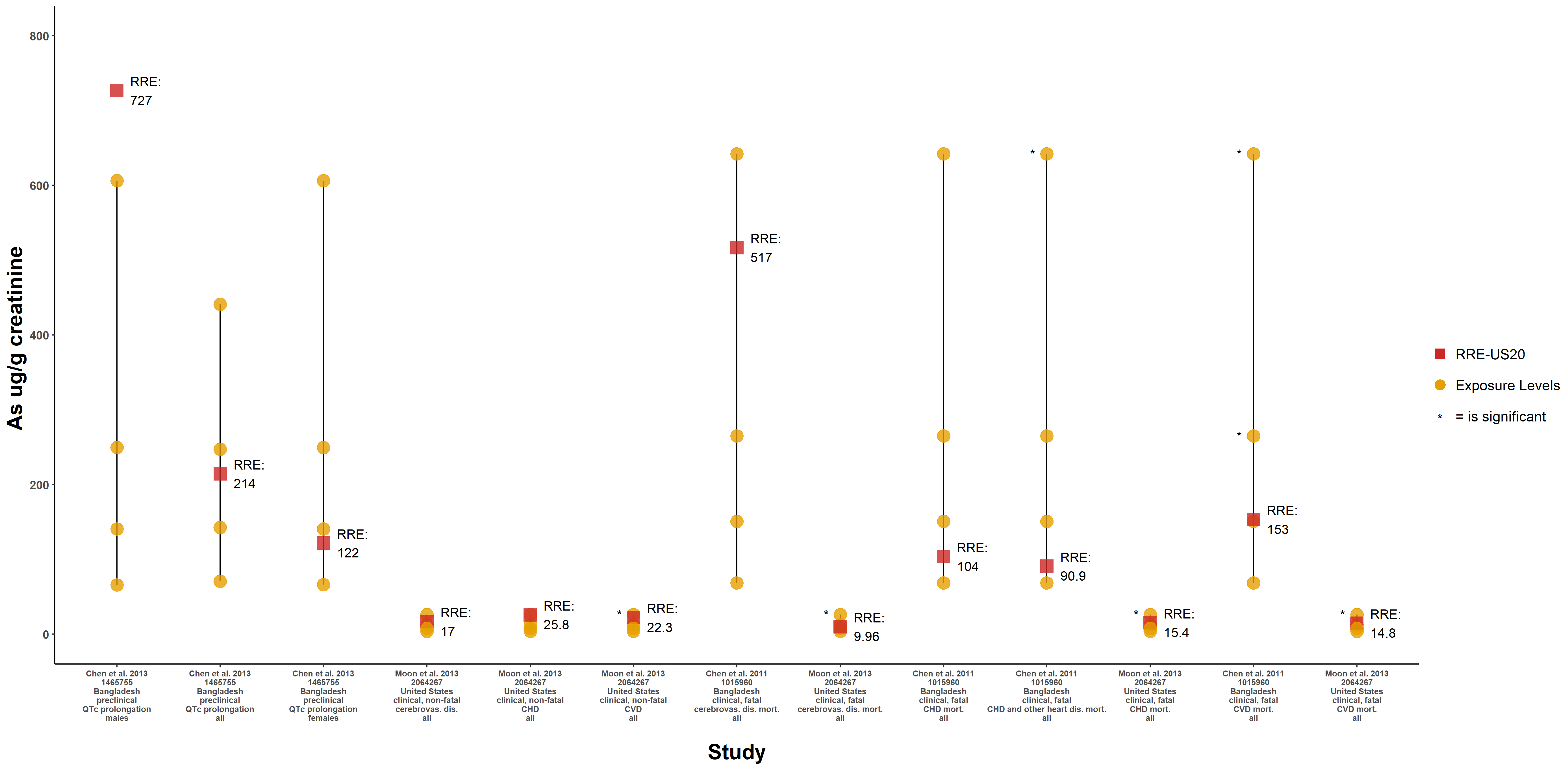


Figure S-14A. Exposure levels and RRE-US_20_ for diseases of the circulatory system using creatinine adjusted urine concentration.


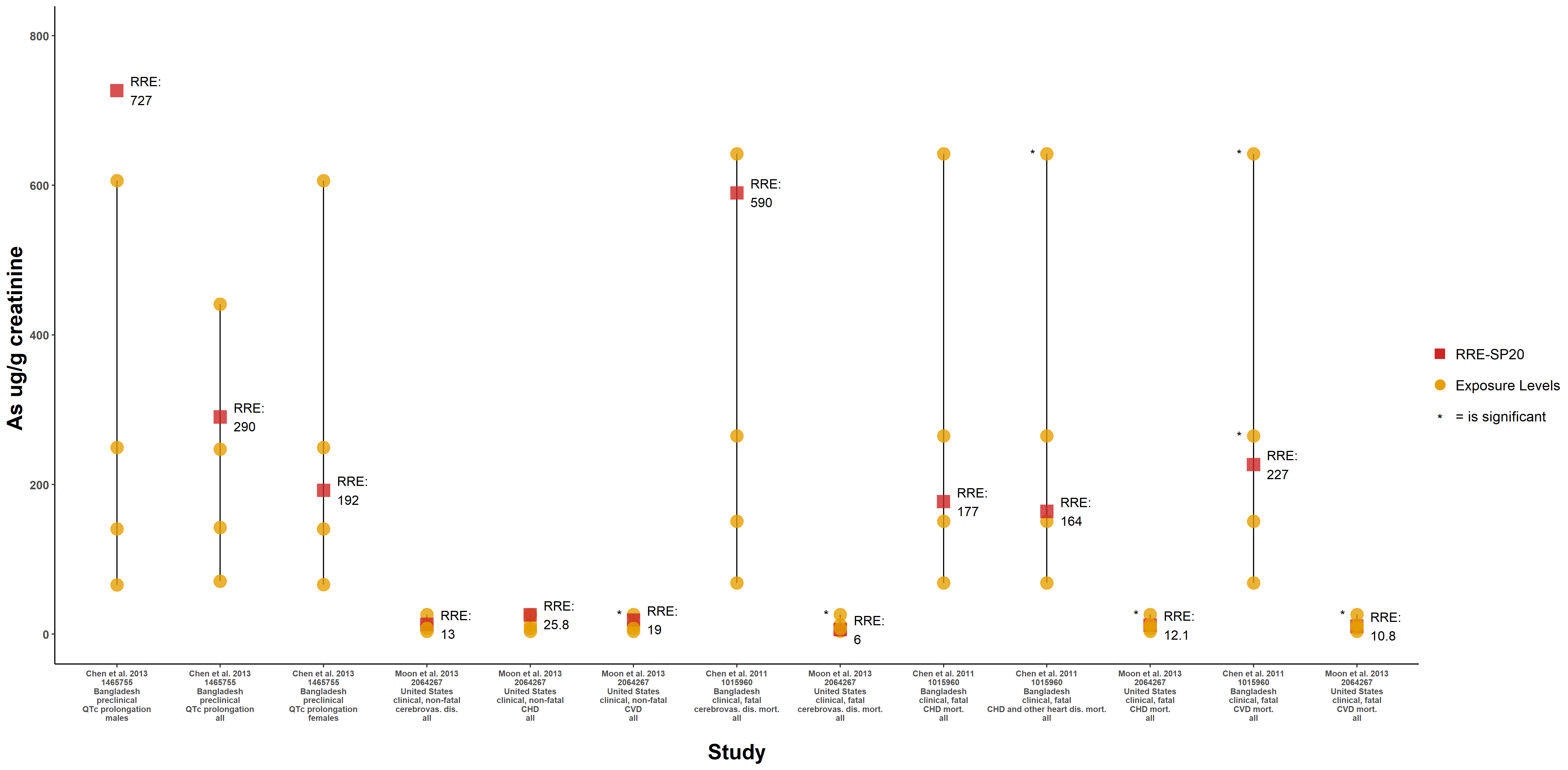


Figure S-14B. Exposure levels and RRE-SP_20_ for diseases of the circulatory system using creatinine adjusted urine concentration.


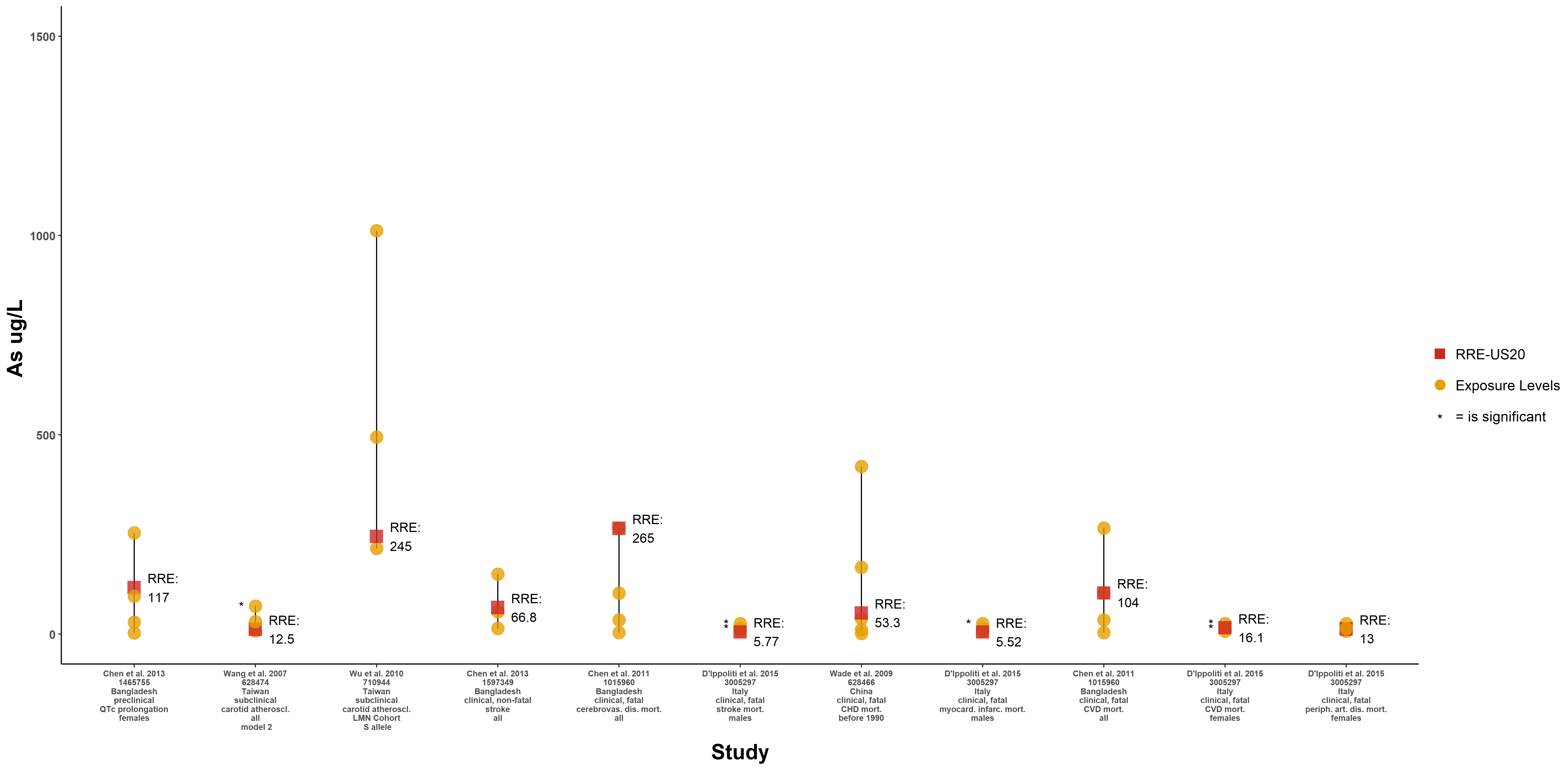


Figure S-15A. Exposure levels and RRE-US_20_ for diseases of the circulatory system using water concentration
Part 1 of 3.


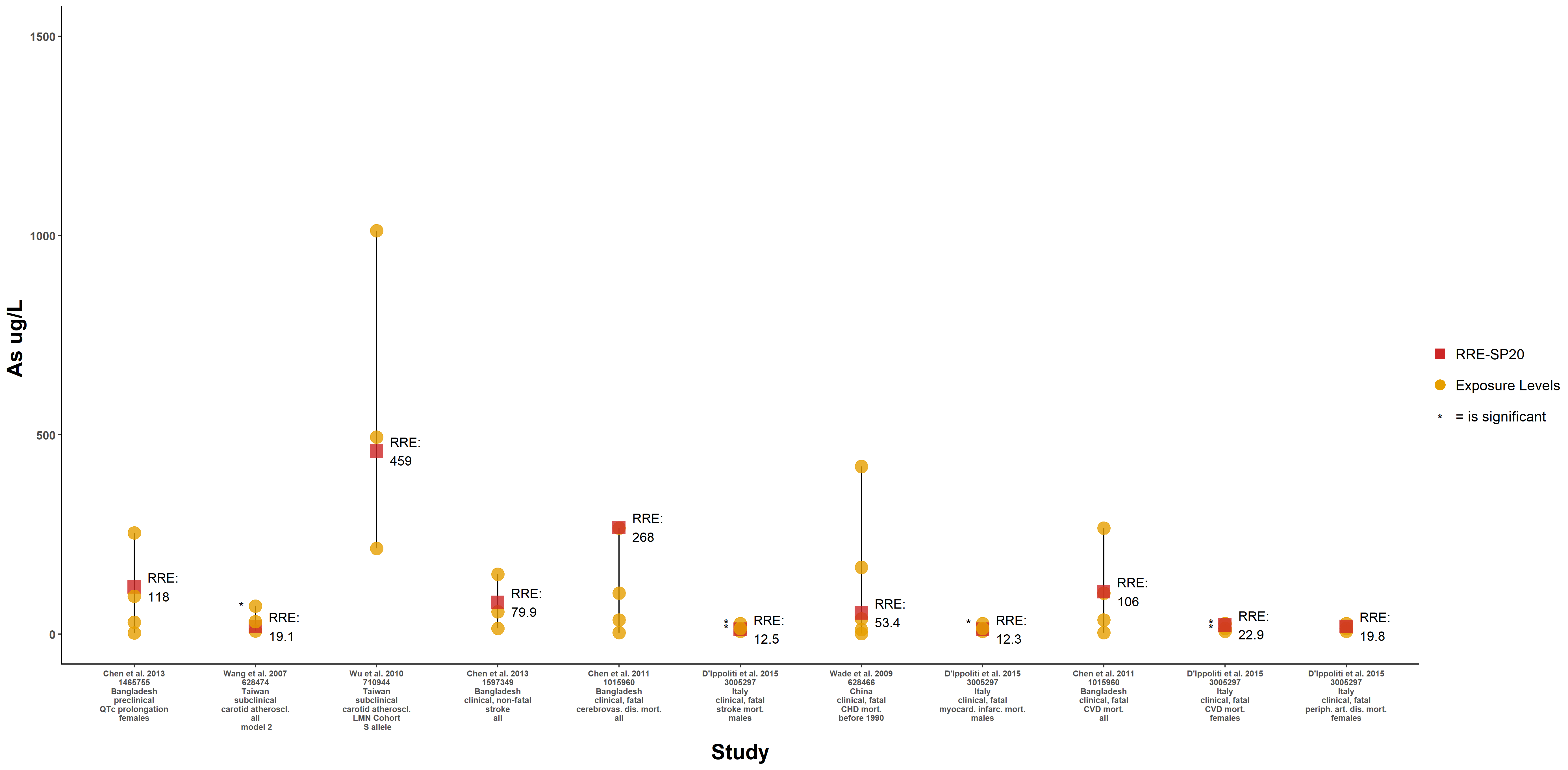


Figure S-15B. Exposure levels and RRE-SP_20_ for diseases of the circulatory system using water concentration
Part 1 of 3.


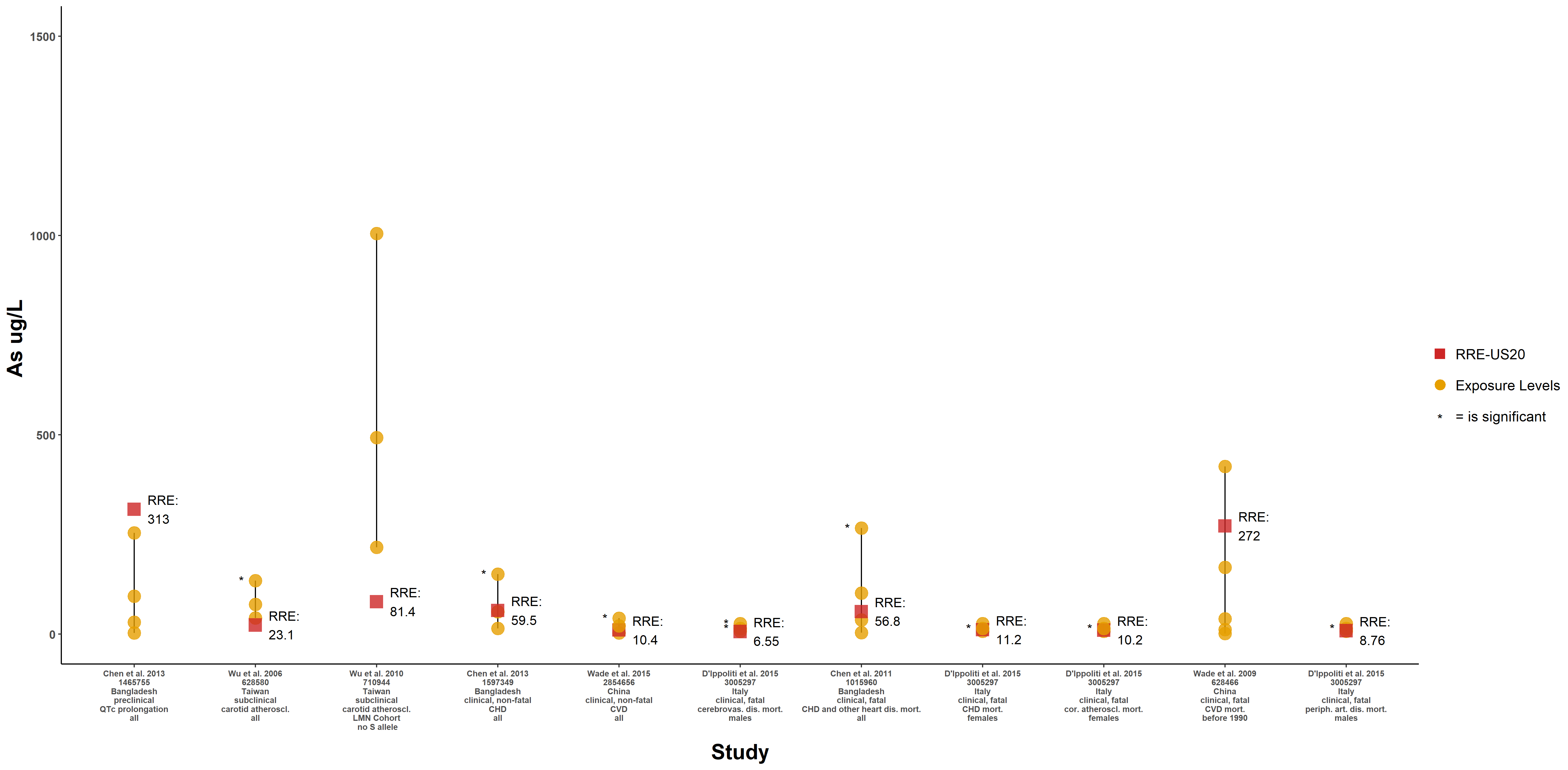


Figure S-16A. Exposure levels and RRE-US_20_ for diseases of the circulatory system using water concentration
Part 2 of 3.


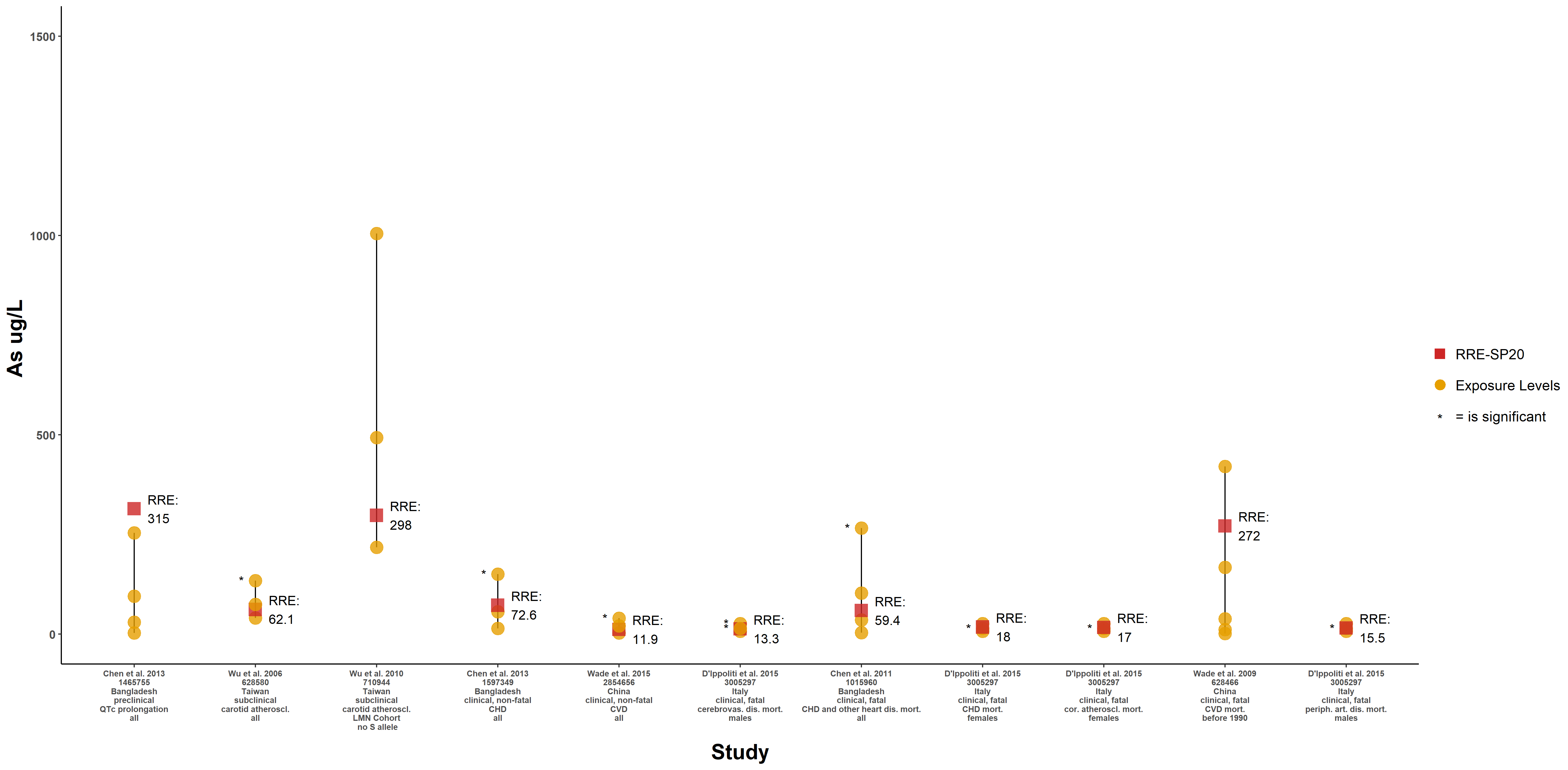


Figure S-16B. Exposure levels and RRE-SP_20_ for diseases of the circulatory system using water concentration
Part 2 of 3.


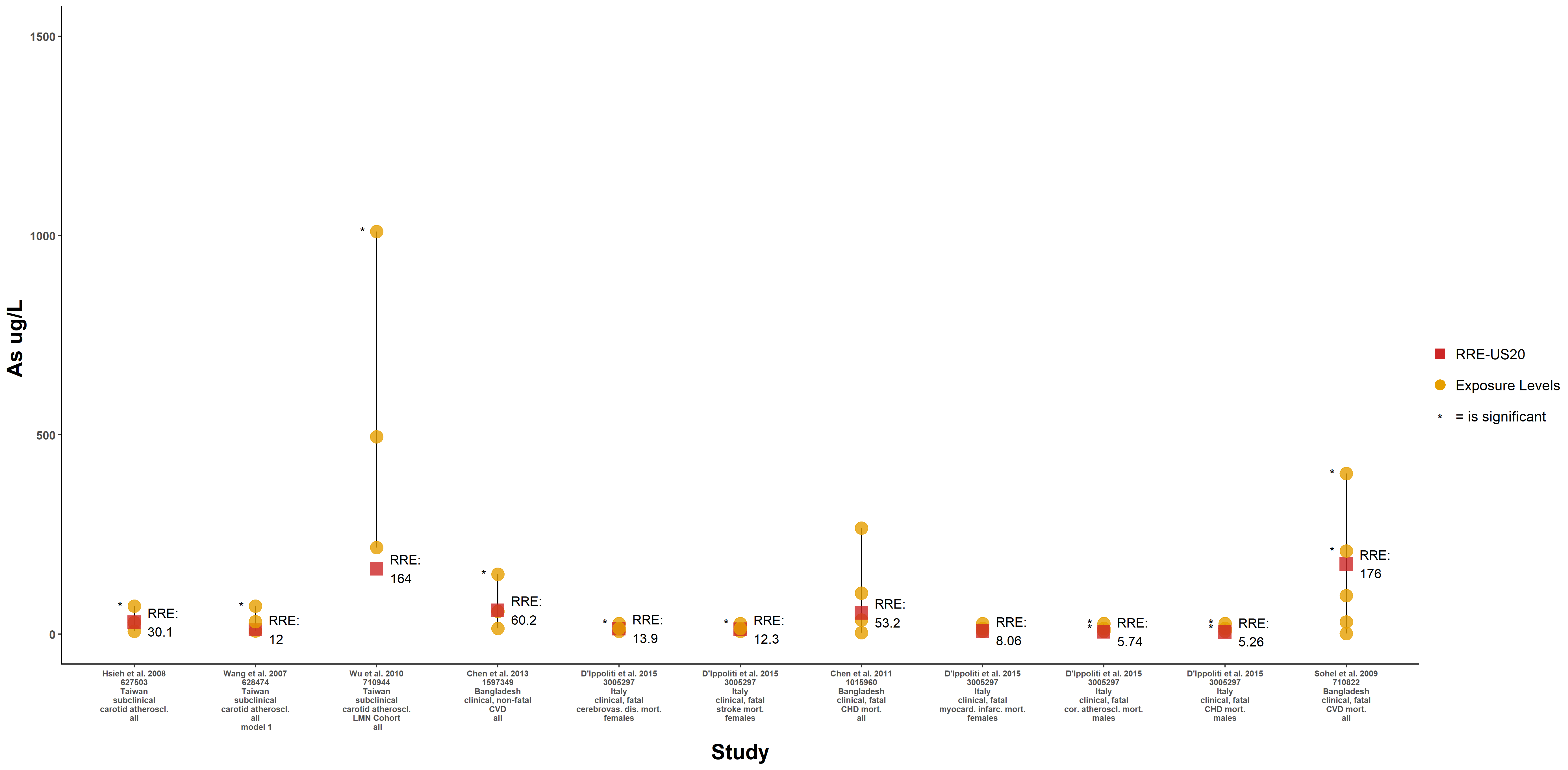


Figure S-17A. Exposure levels and RRE-US_20_ for diseases of the circulatory system using water concentration
Part 3 of 3.


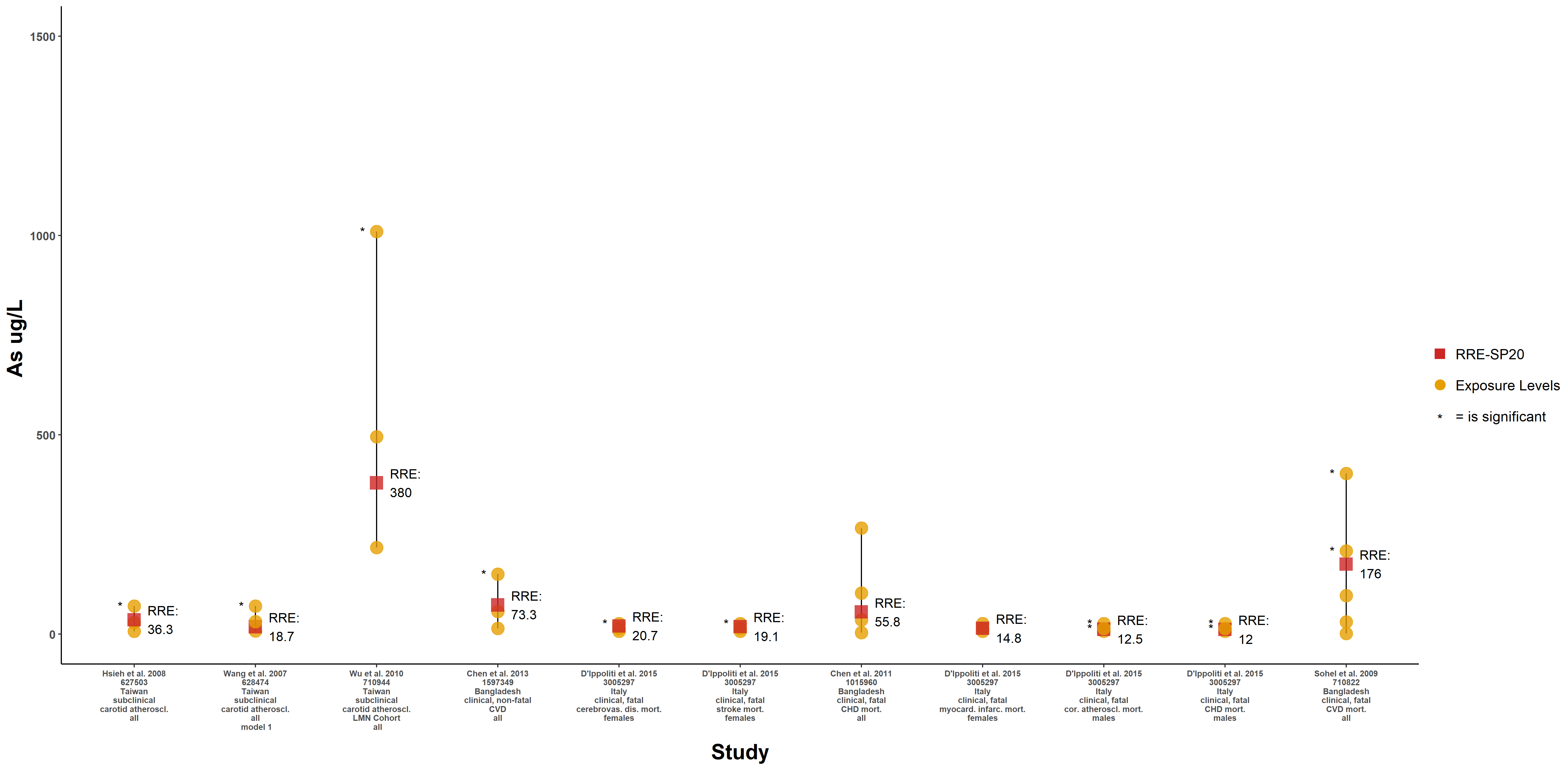


Figure S-17B. Exposure levels and RRE-SP_20_ for diseases of the circulatory system using water concentration
Part 3 of 3.

Table S-30A. Summary of RRE-US_20_s and RRB-US for diseases of the circulatory system studies


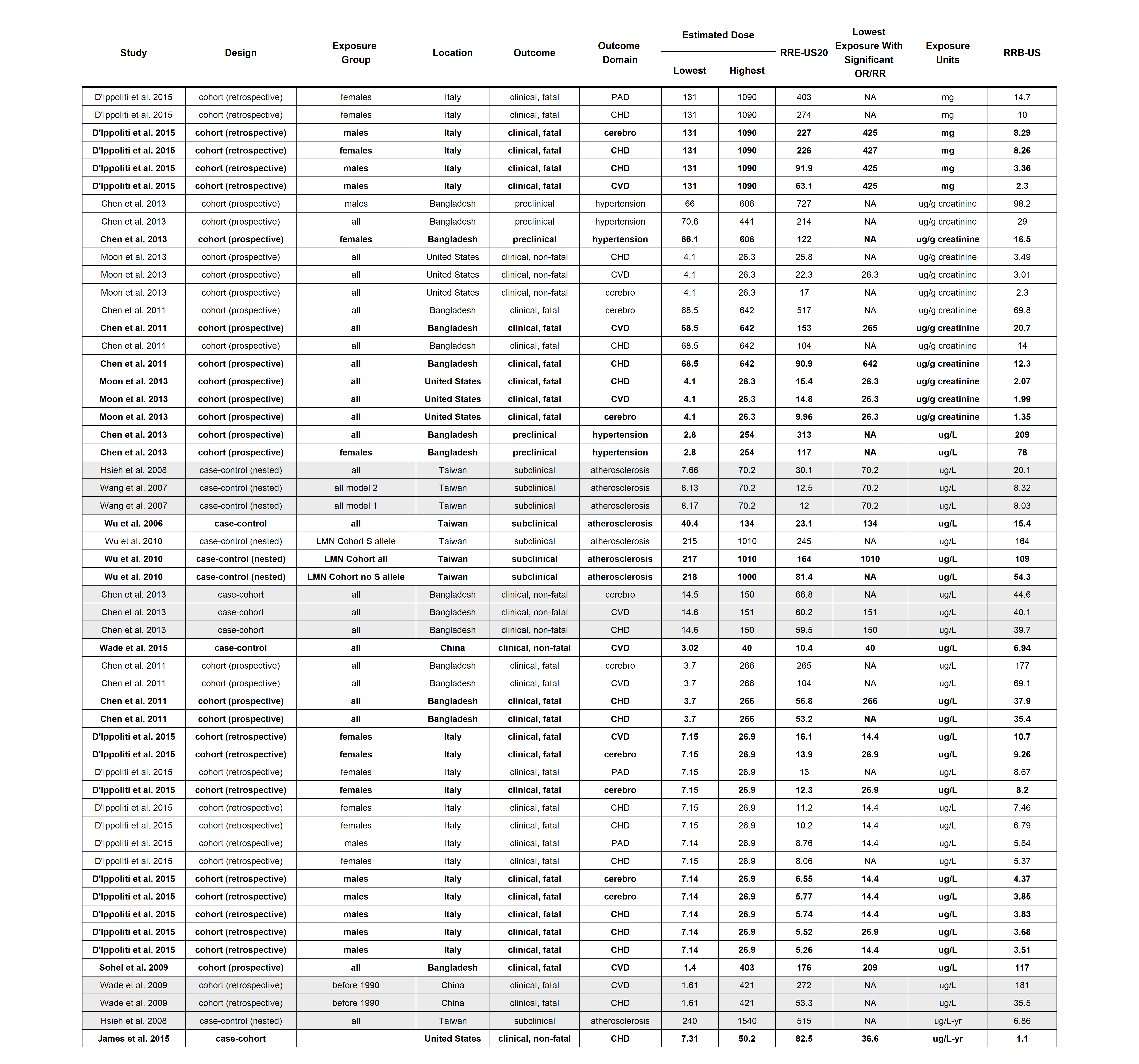


RRB-US refers to the ratio of RRE-US_20_ to an estimated U.S. background exposure level. Shaded cells indicate that authors did not report exposure-response trends. Bold rows indicate that authors reported a significant exposure-response trend (*p* <0.05)

Table S-30B. Summary of RRE-SP_20_s and RRB-SP for diseases of the circulatory system studies


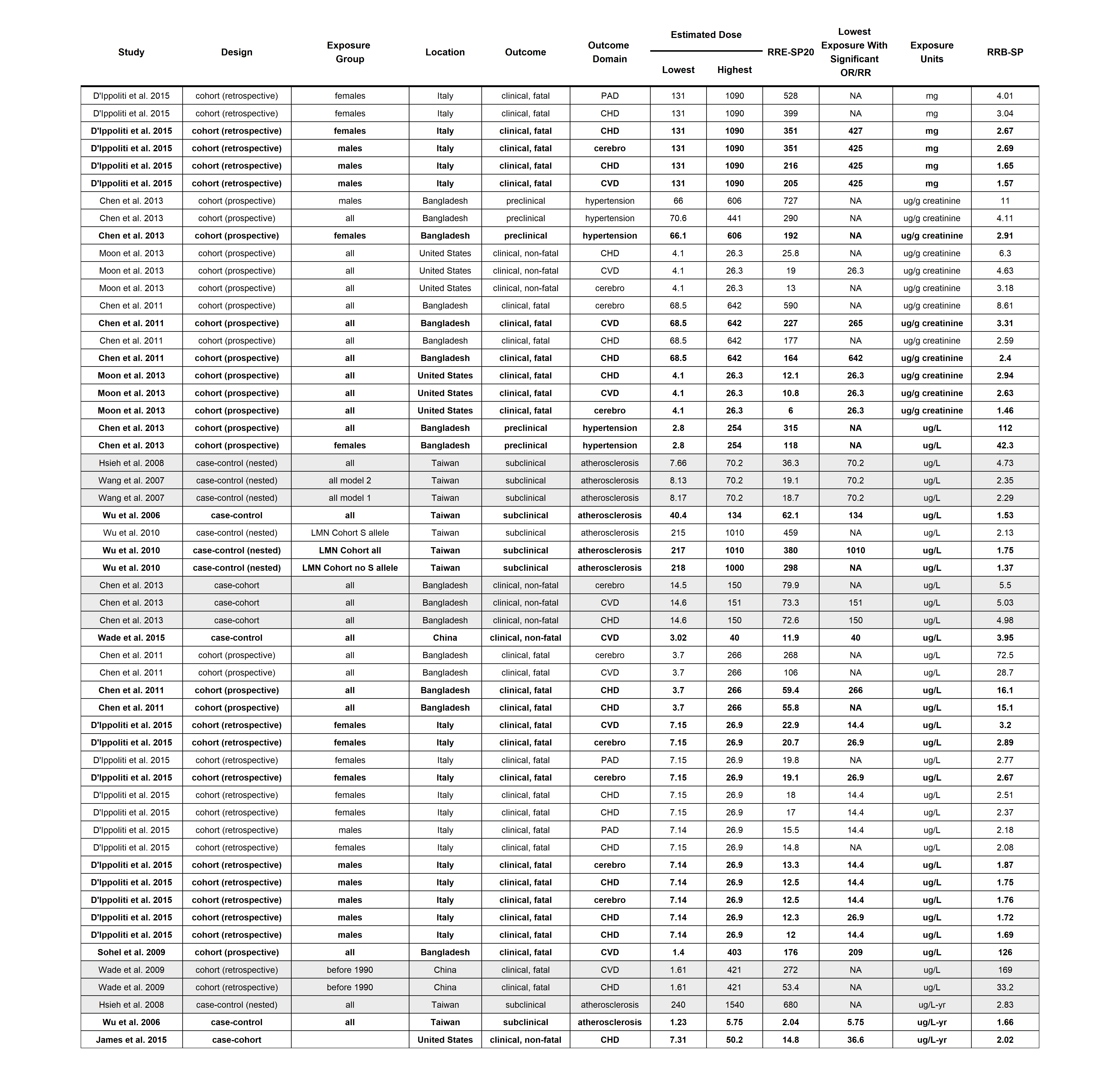


RRB-SP refers to the ratio of RRE-SP_20_ to the reported or estimated background exposure level for the study referent group. Shaded cells indicate that authors did not report exposure-response trends. Bold rows indicate that authors reported a significant exposure-response trend (*p* <0.05)

#### Liver Cancer Exposure-Response Modeling Results

The analysis of arsenic exposure response on liver cancer outcomes evaluated 7 datasets from 3 peer reviewed studies that included endpoints such as liver, gallbladder, and bile duct cancers. A summary of datasets modeled identifying the study design, location, exposure metric and outcome domain are provided in Table S-31 below. A breakdown of the exposure levels and RRE_20_ estimates are provided for each exposure metric in Figure S-18–Figure S-21. Finally, RRE_20_ summary tables for all exposures are provided in Table S-32.

Table S-31. Summary of datasets considered in liver cancer exposure-response RRB analysis by exposure metric


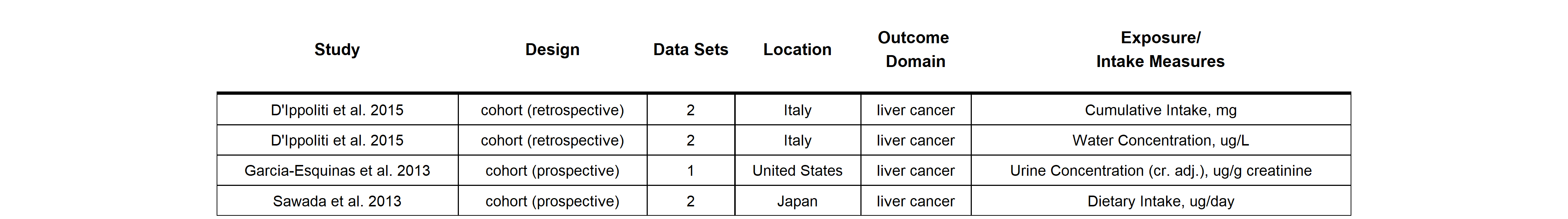


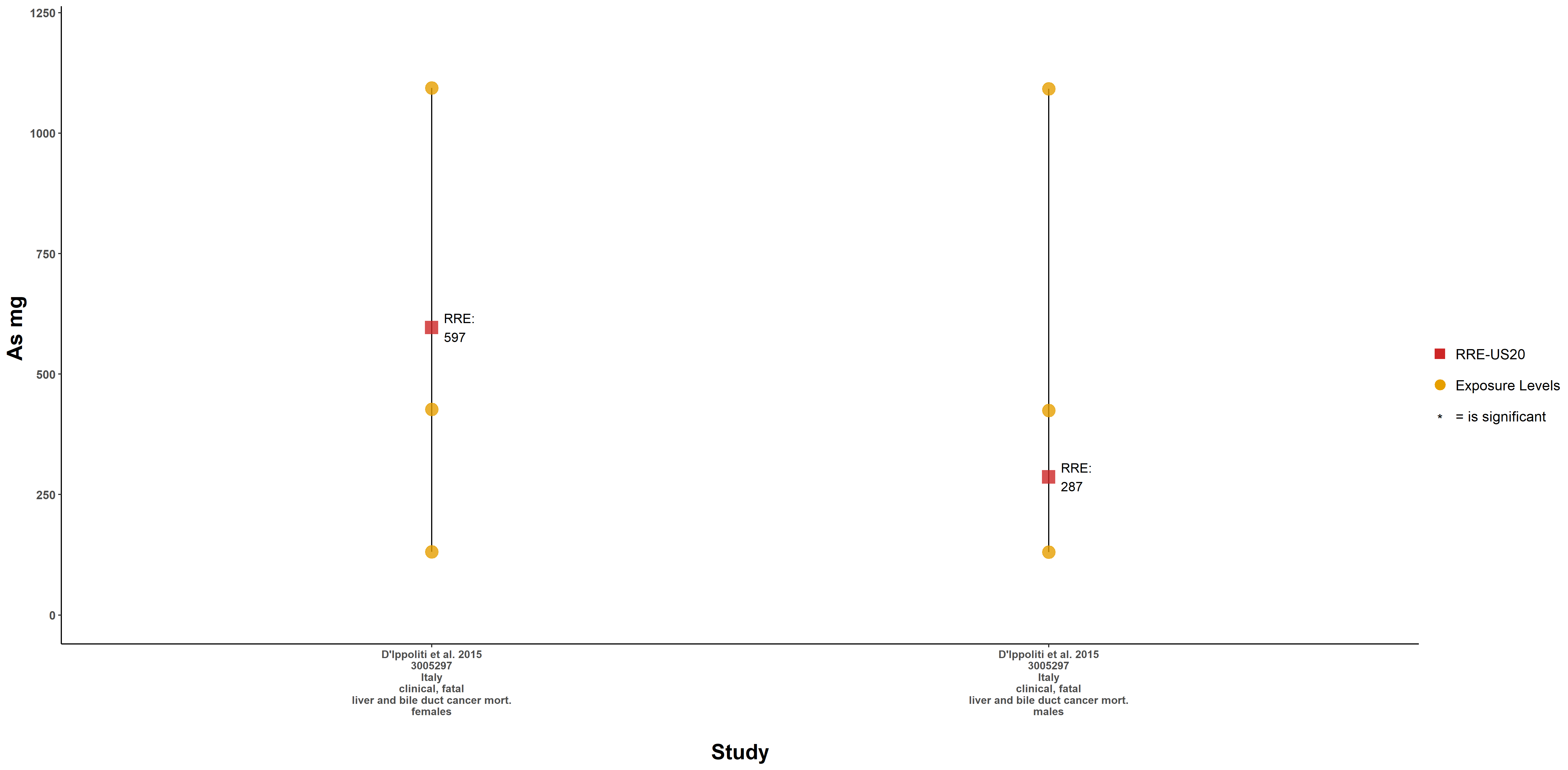


Figure S-18A. Exposure levels and RRE-US_20_ for liver cancer using cumulative intake.


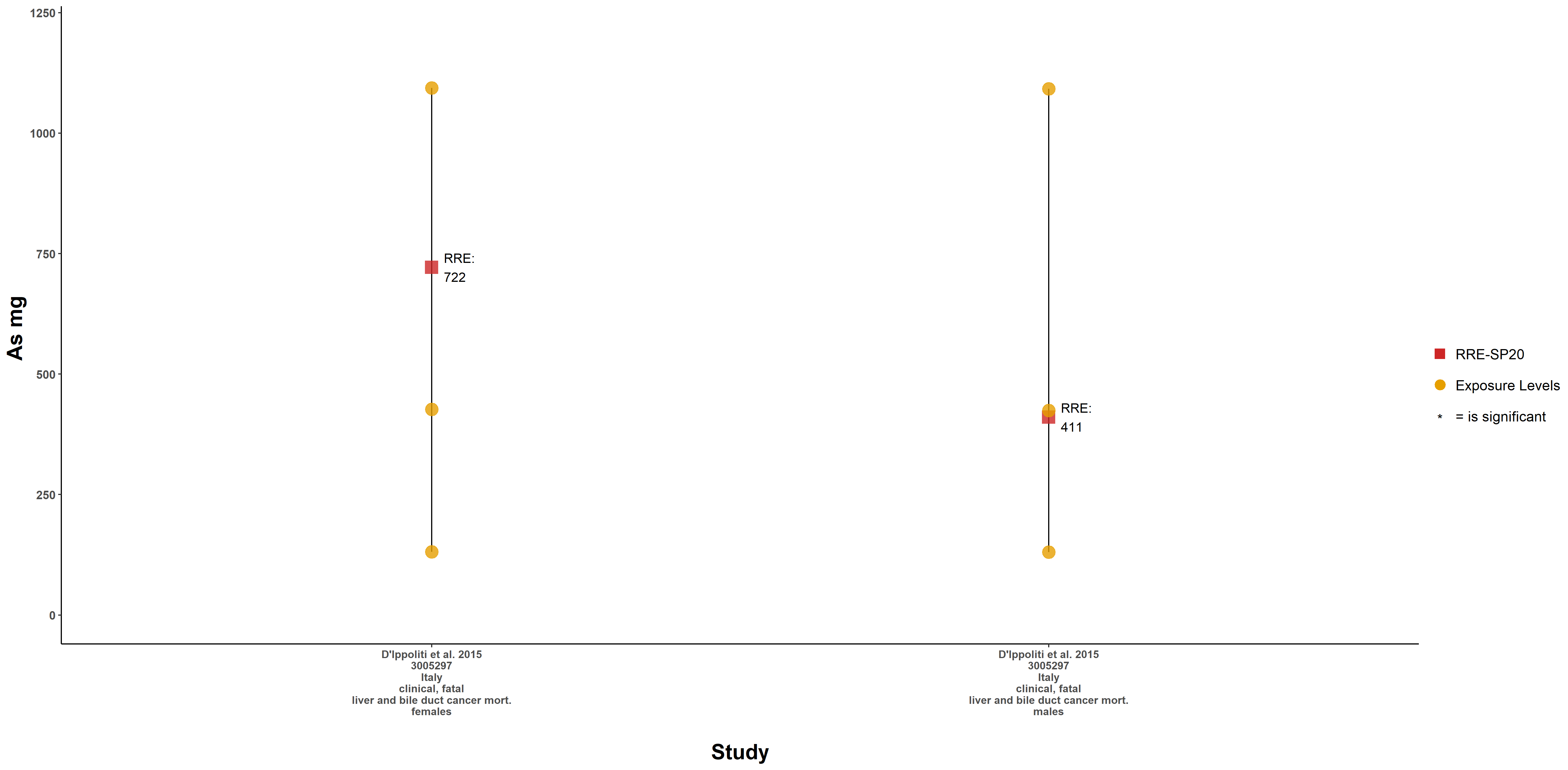


Figure S-18A. Exposure levels and RRE-SP_20_ for liver cancer using cumulative intake.


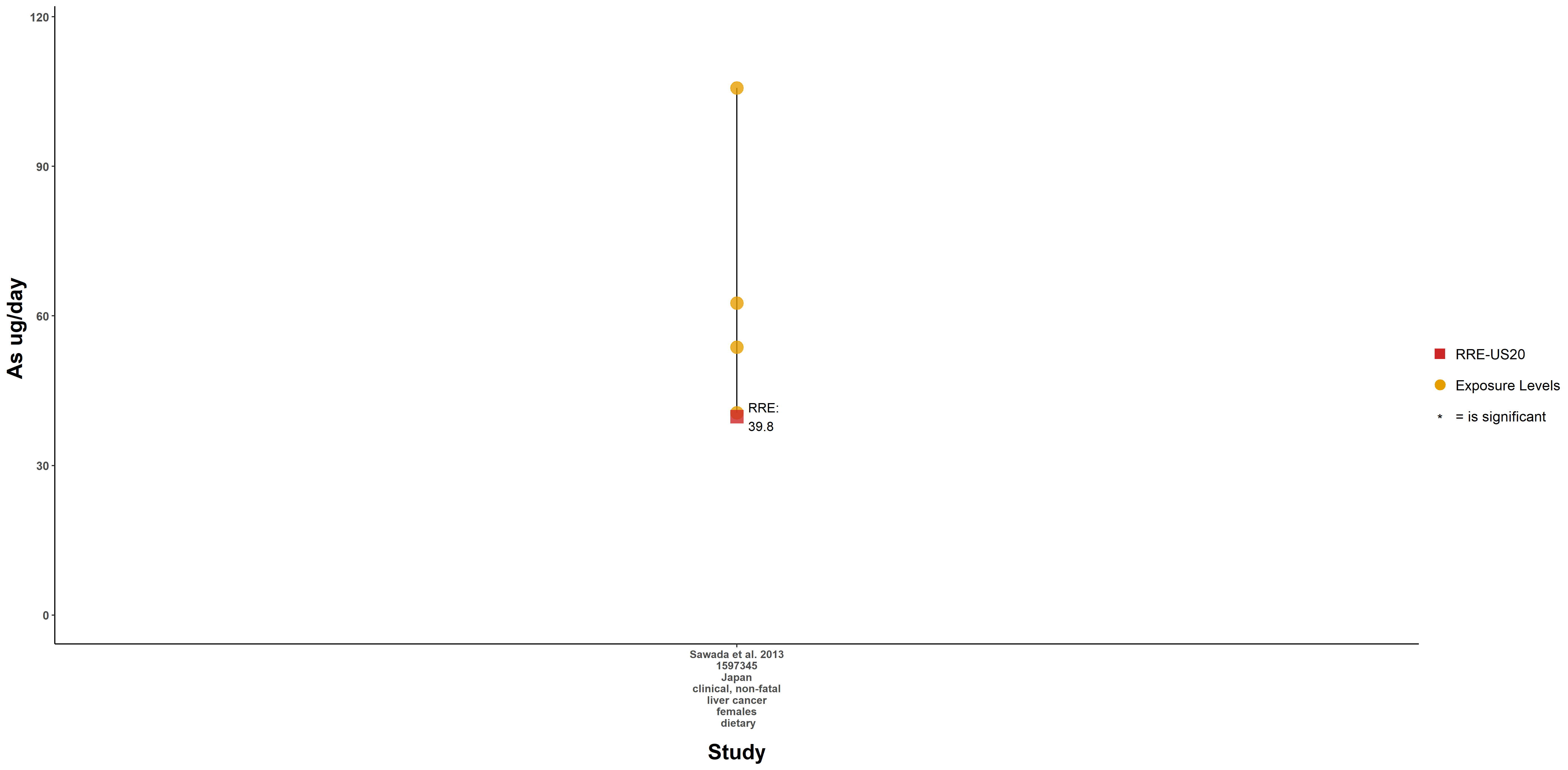


Figure S-19A. Exposure levels and RRE-US_20_ for liver cancer using dietary intake.


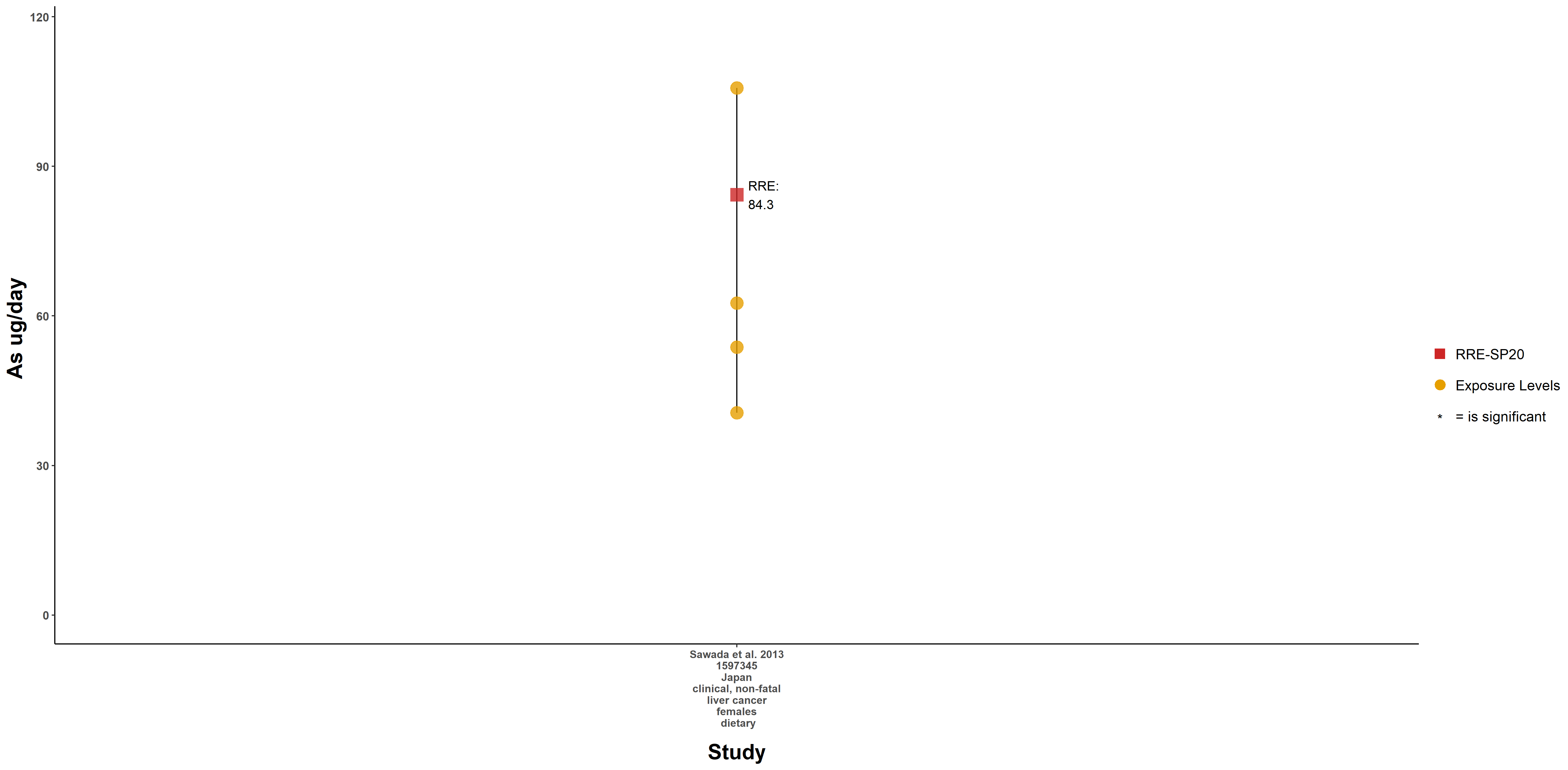


Figure S-19B. Exposure levels and RRE-SP_20_ for liver cancer using dietary intake.


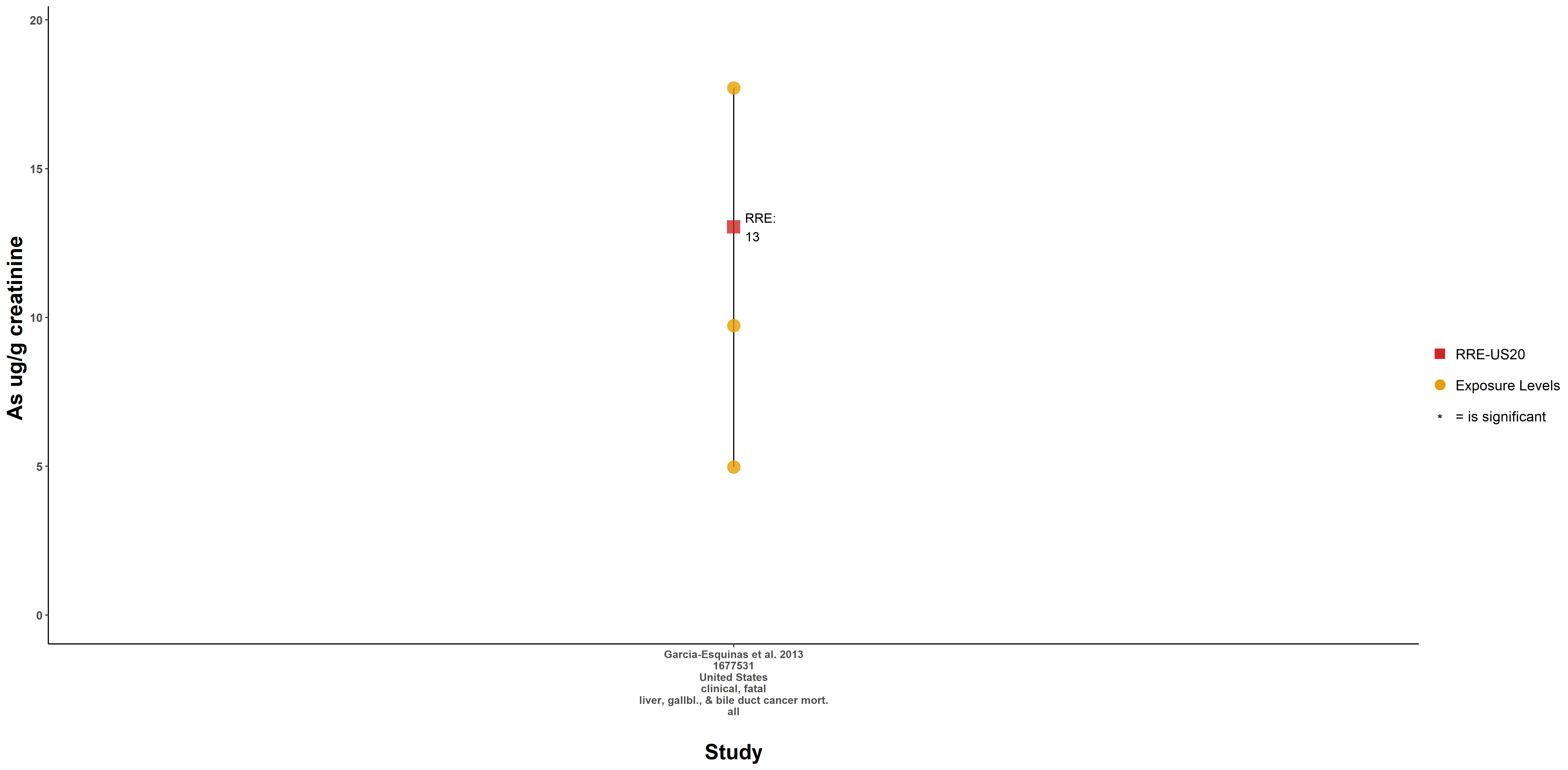


Figure S-20A. Exposure levels and RRE-US_20_ for liver cancer using creatinine adjusted urine concentration.


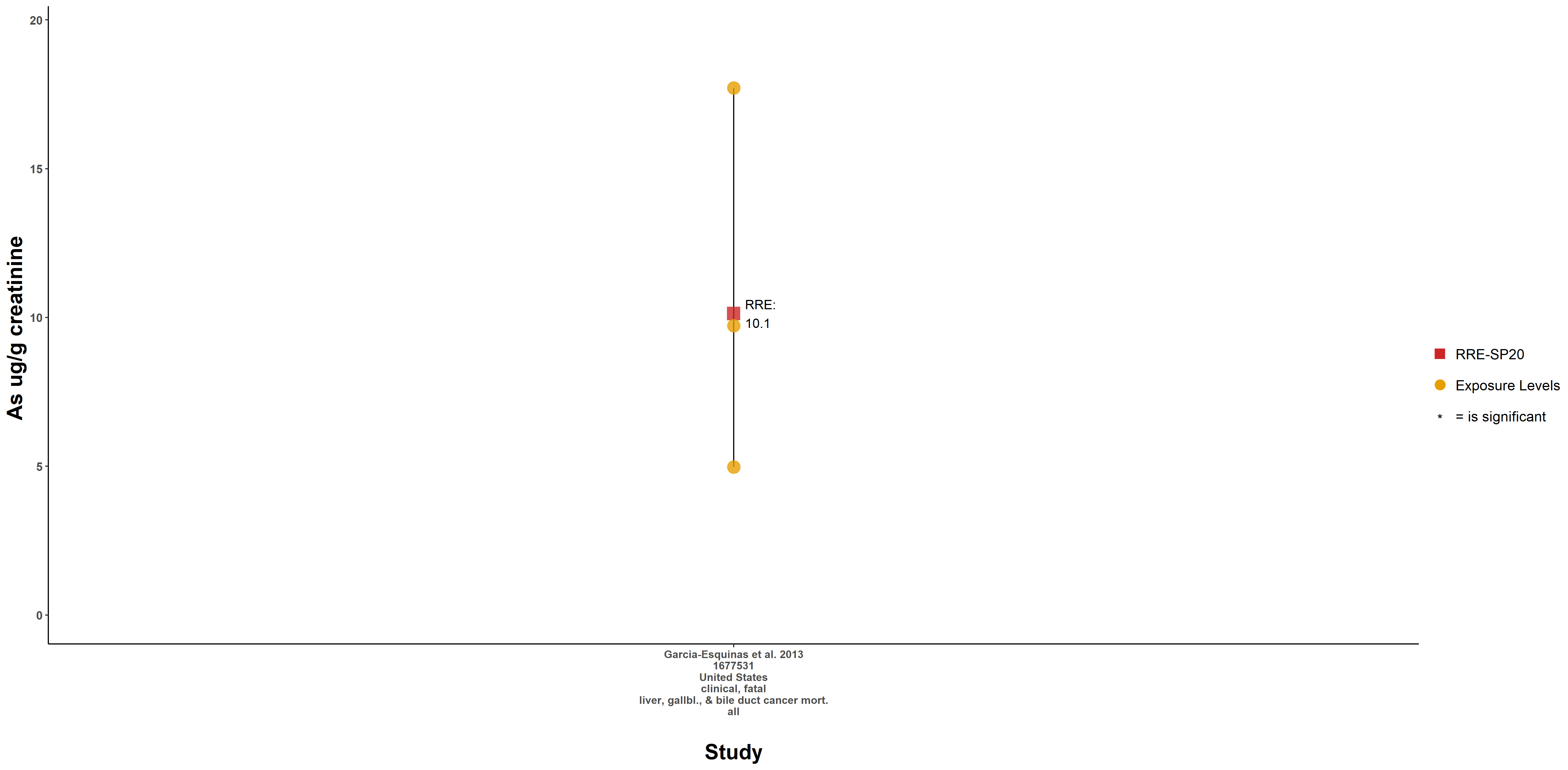


Figure S-20B. Exposure levels and RRE-SP_20_ for liver cancer using creatinine adjusted urine concentration.


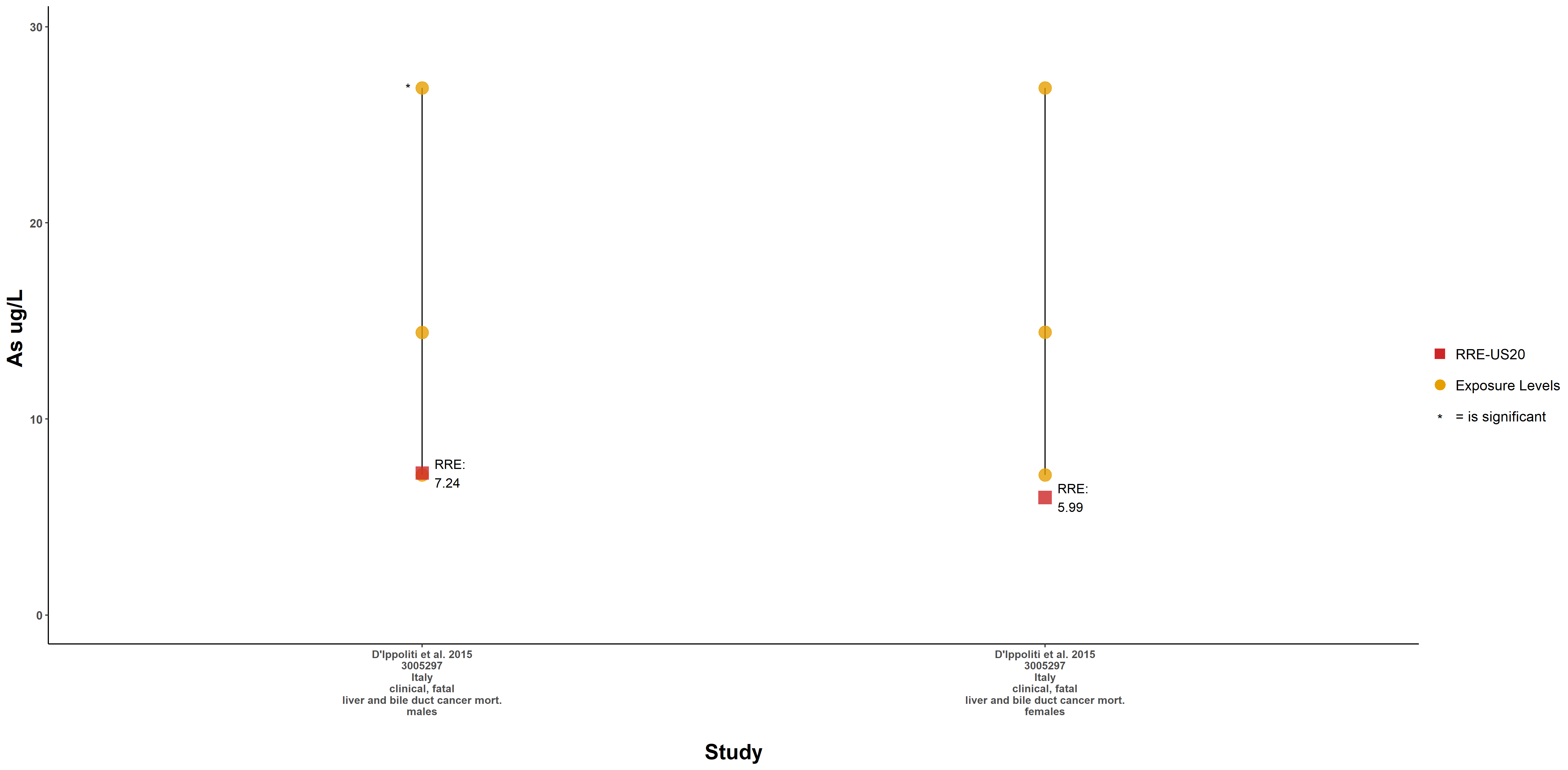


Figure S-21A. Exposure levels and RRE-US_20_ for liver cancer using water concentration.


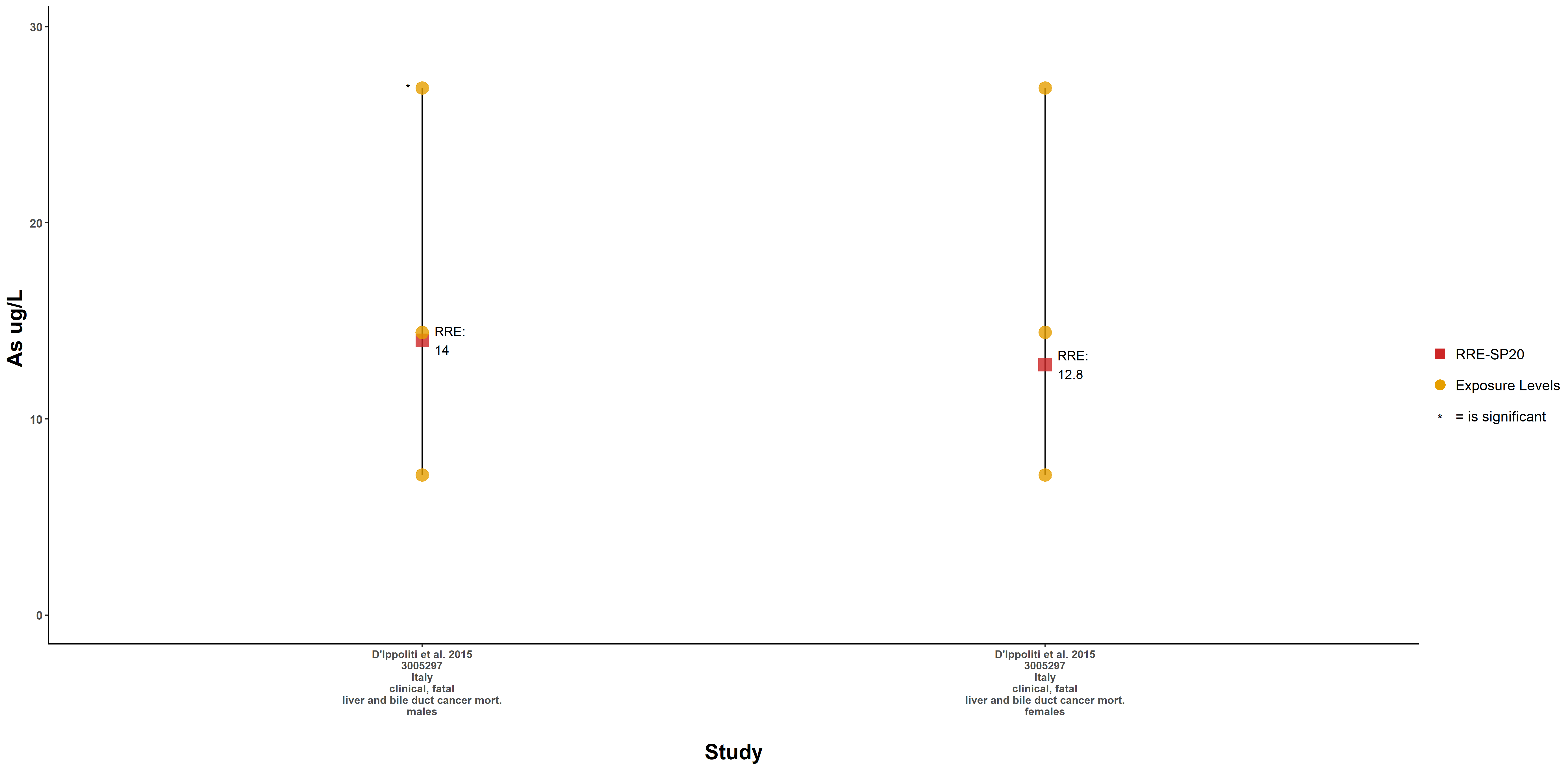


Figure S-21B. Exposure levels and RRE-SP_20_ for liver cancer using water concentration.

Table S-32A. Summary of RRE-US_20_s and RRB-US for liver cancer studies


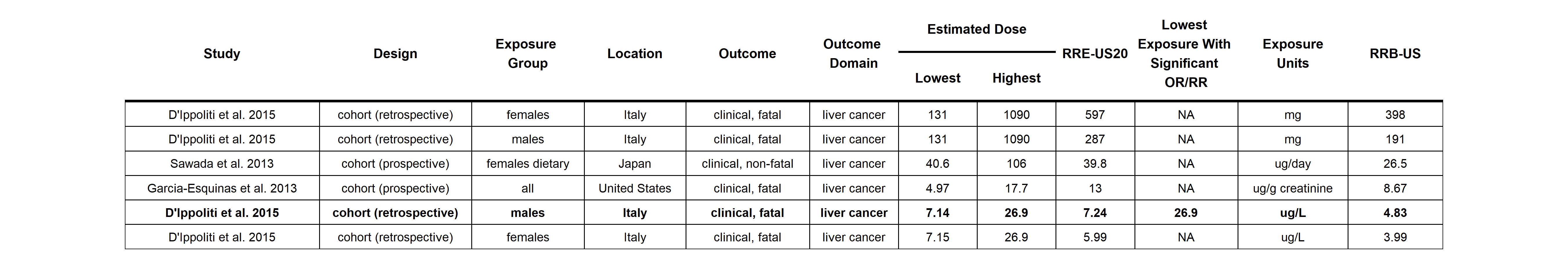


RRB-US refers to the ratio of RRE_US_20_ to an estimated U.S. background exposure level. Shaded cells indicate that authors did not report exposure-response trends. Bold rows indicate that authors reported a significant exposure-response trend (*p* <0.05)

Table S-32B. Summary of RRE-SP_20_s and RRB-SP for liver cancer studies


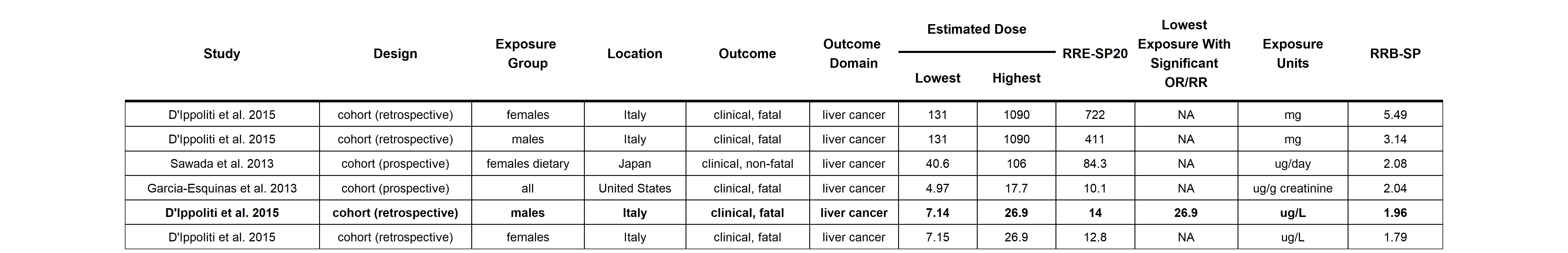


RRB-SP refers to the ratio of RRE-SP_20_ to the reported or estimated background exposure level for the study referent group. Shaded cells indicate that authors did not report exposure-response trends. Bold rows indicate that authors reported a significant exposure-response trend (*p* <0.05)

#### Lung Cancer Exposure-Response Modeling Results

The analysis of arsenic exposure response on lung cancer outcomes evaluated 35 datasets from 16 peer reviewed studies that included endpoints such as lung adenocarcinoma and squamous cell carcinoma as well as cancers of the trachea and bronchus. A summary of datasets modeled identifying the study design, location, exposure metric and outcome domain is provided in Table S-33 below. This analysis generated 29 models that met our initial model selection criteria. Of the 29 selected models, 28 models had RRE_20_ estimates ranging within a factor of three of the central estimates for the lowest or highest dose group and are presented in the results below. A breakdown of the exposure levels and RRE_20_ estimates is provided for each exposure metric in Figure S-21–Figure I‑27. Finally, an RRE_20_ summary table for all exposures is provided in Table S-34.

Table S-33. Summary of datasets considered in lung cancer exposure-response RRB analysis by exposure metric


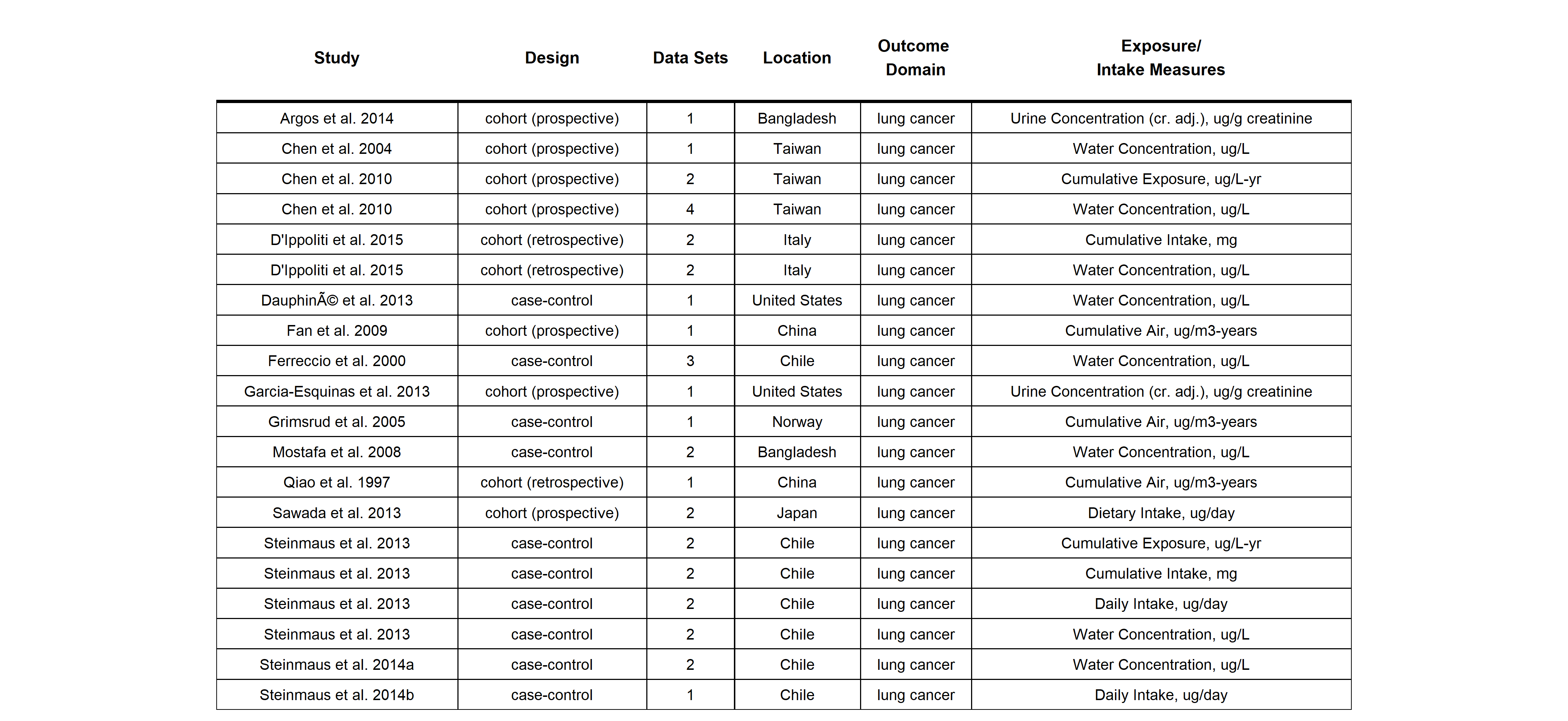


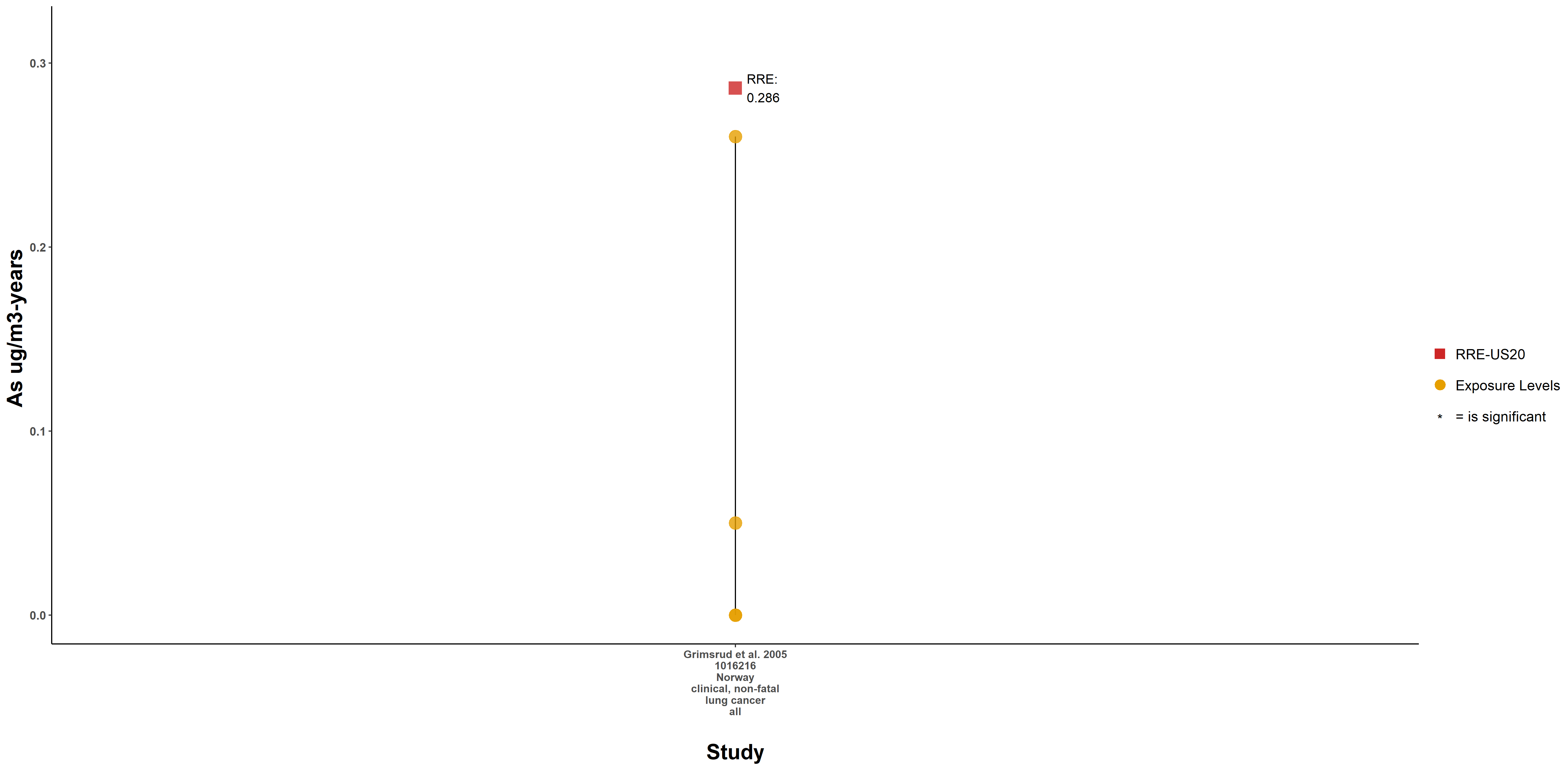


Figure S-22A. Exposure levels and RRE-US_20_ for lung cancer using cumulative air exposure.


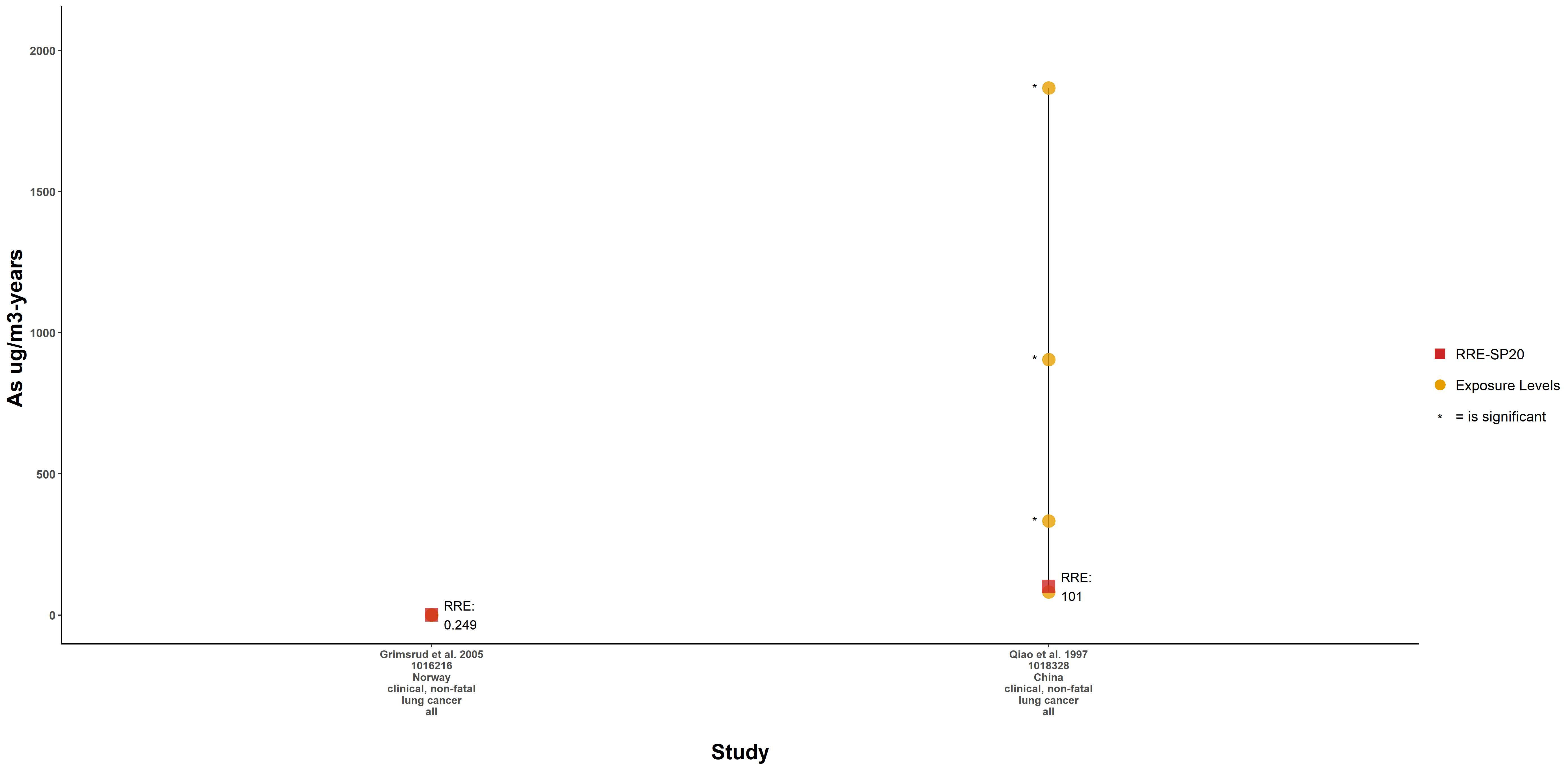


Figure S-22B. Exposure levels and RRE-SP_20_ for lung cancer using cumulative air exposure.


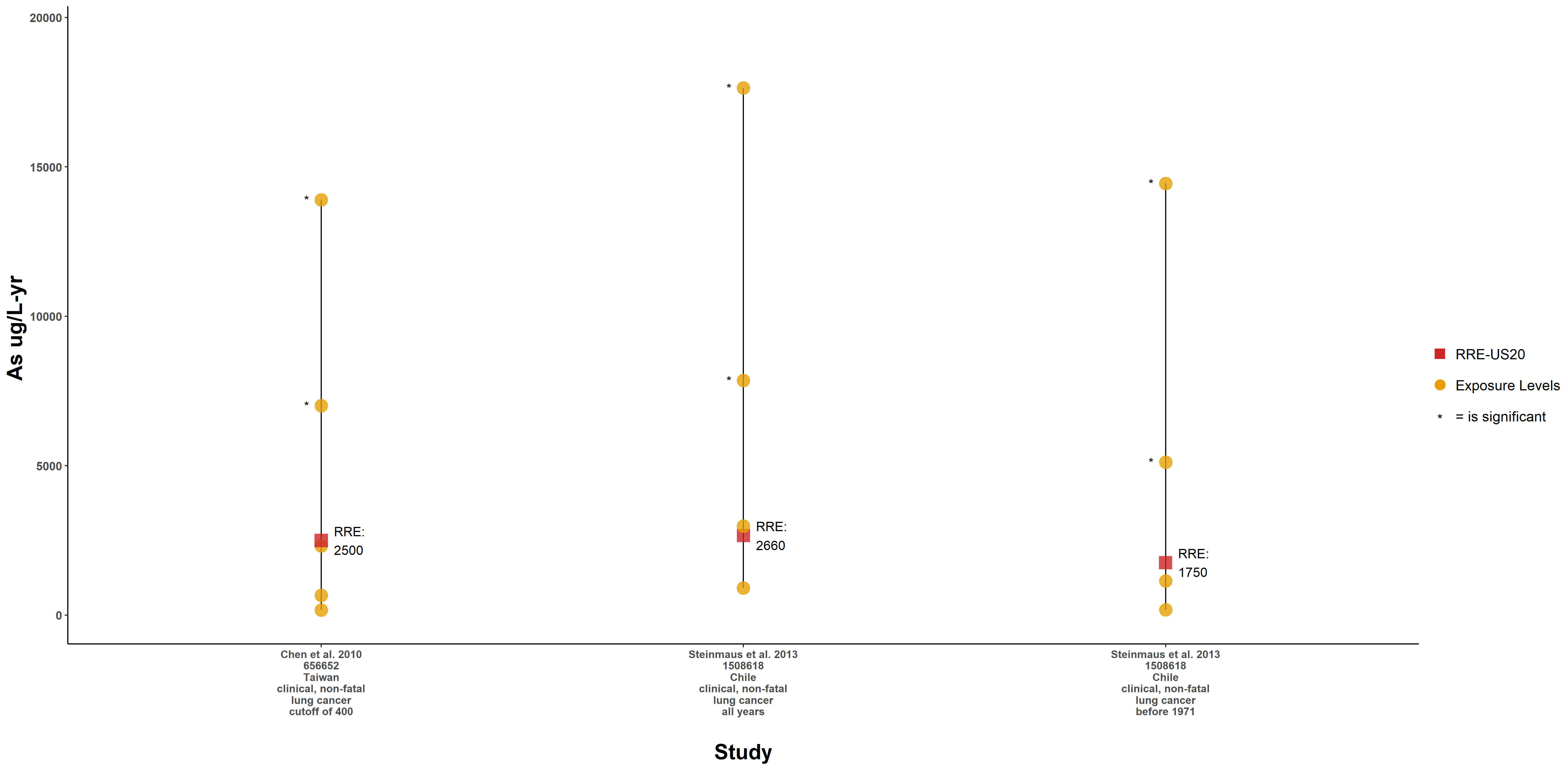


Figure S-23A. Exposure levels and RRE-US_20_ for lung cancer using cumulative exposure.


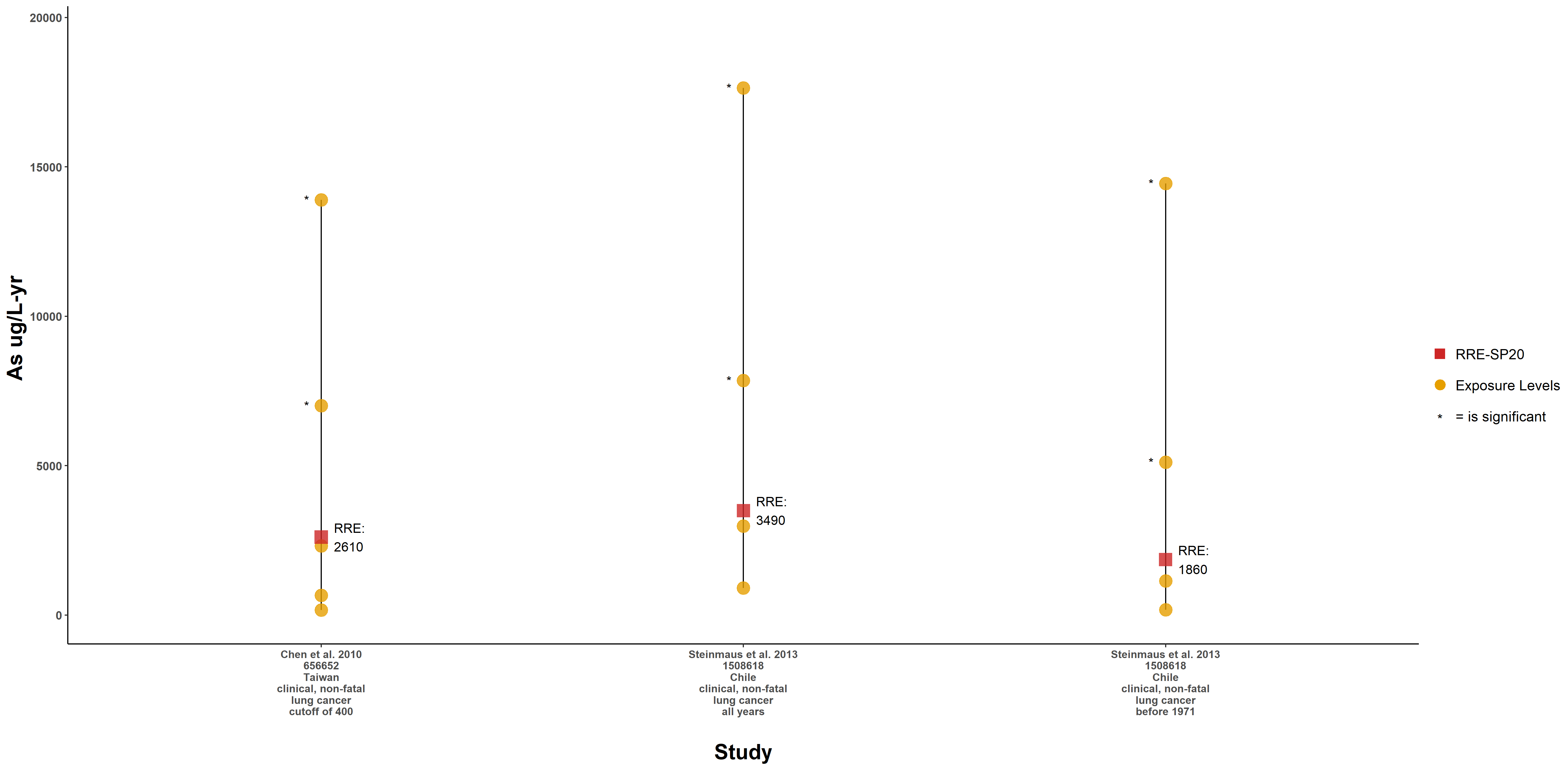


Figure S-23B. Exposure levels and RRE-SP_20_ for lung cancer using cumulative exposure.


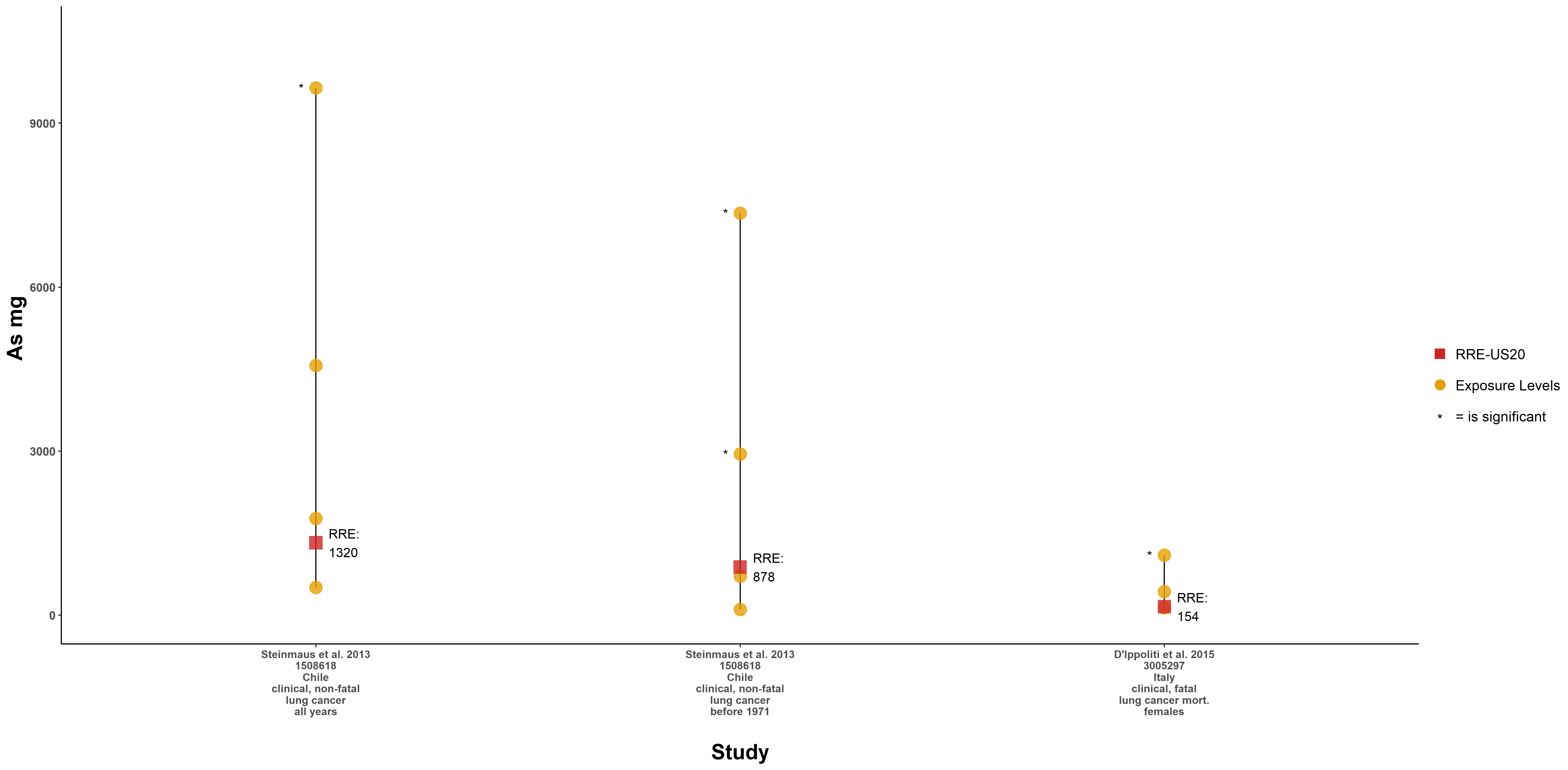


Figure S-24A. Exposure levels and RRE-US_20_ for lung cancer using cumulative intake.


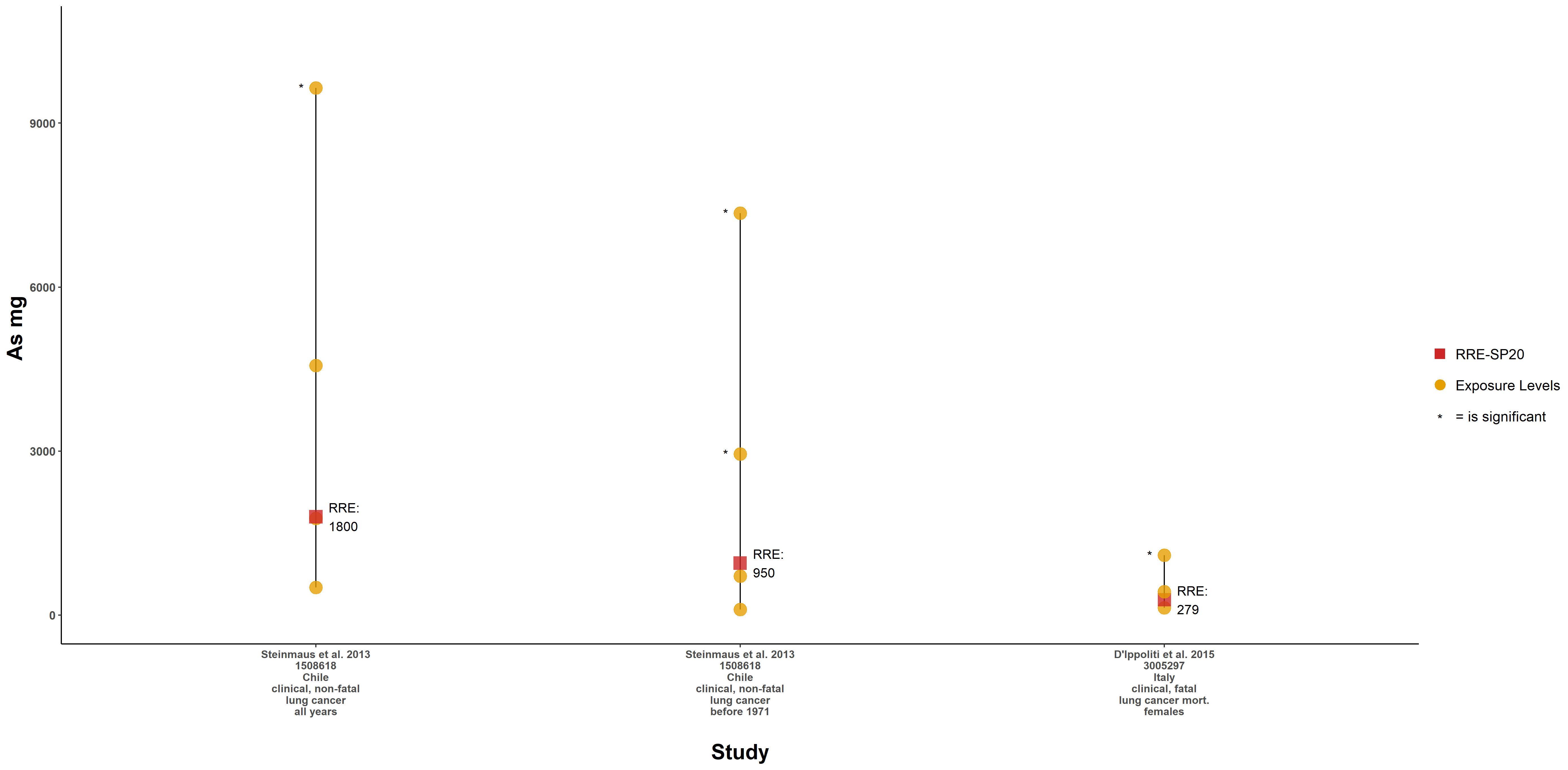


Figure S-24B. Exposure levels and RRE-SP_20_ for lung cancer using cumulative intake.


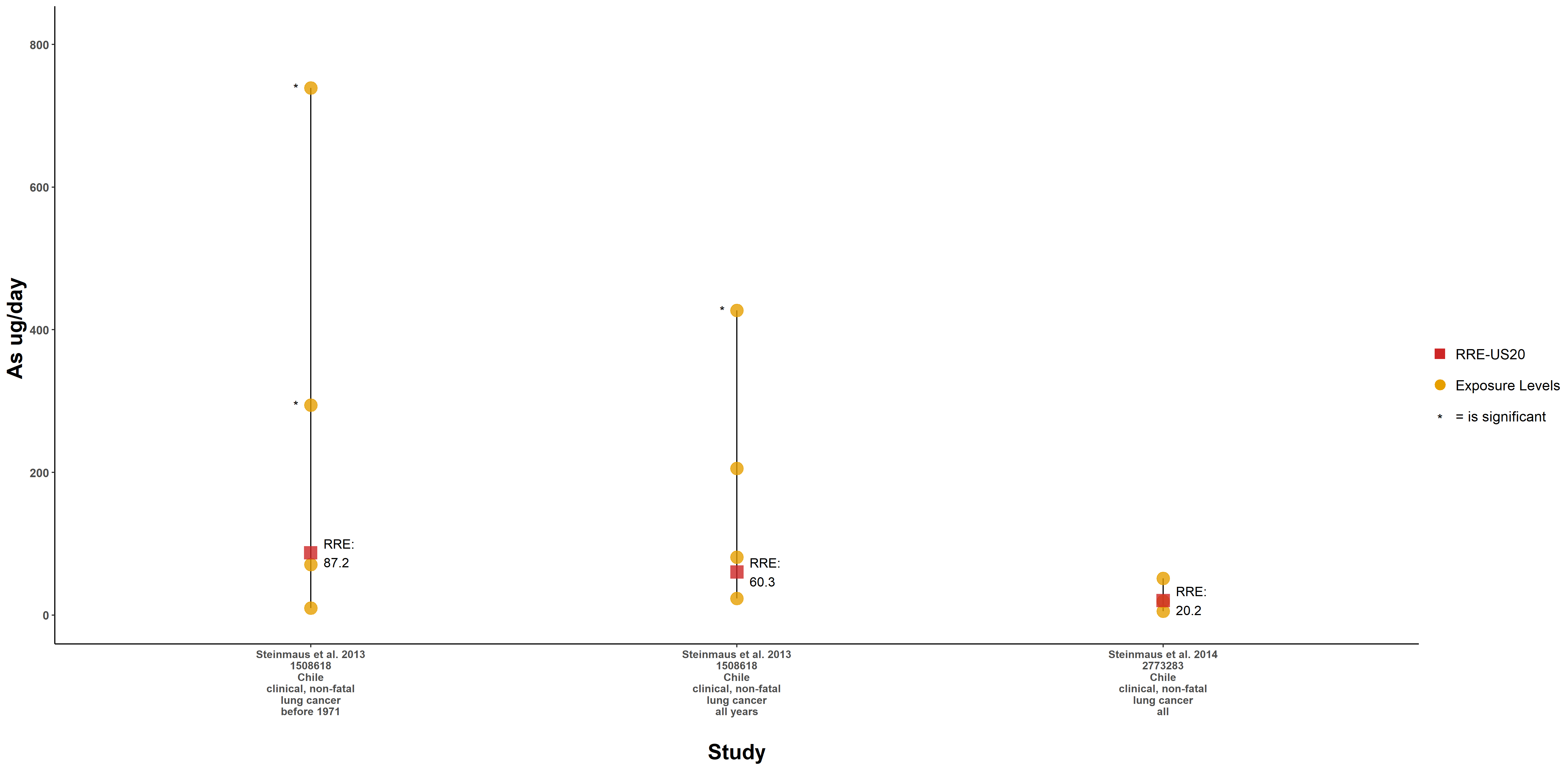


Figure S-25A. Exposure levels and RRE-US_20_ for lung cancer using daily intake.


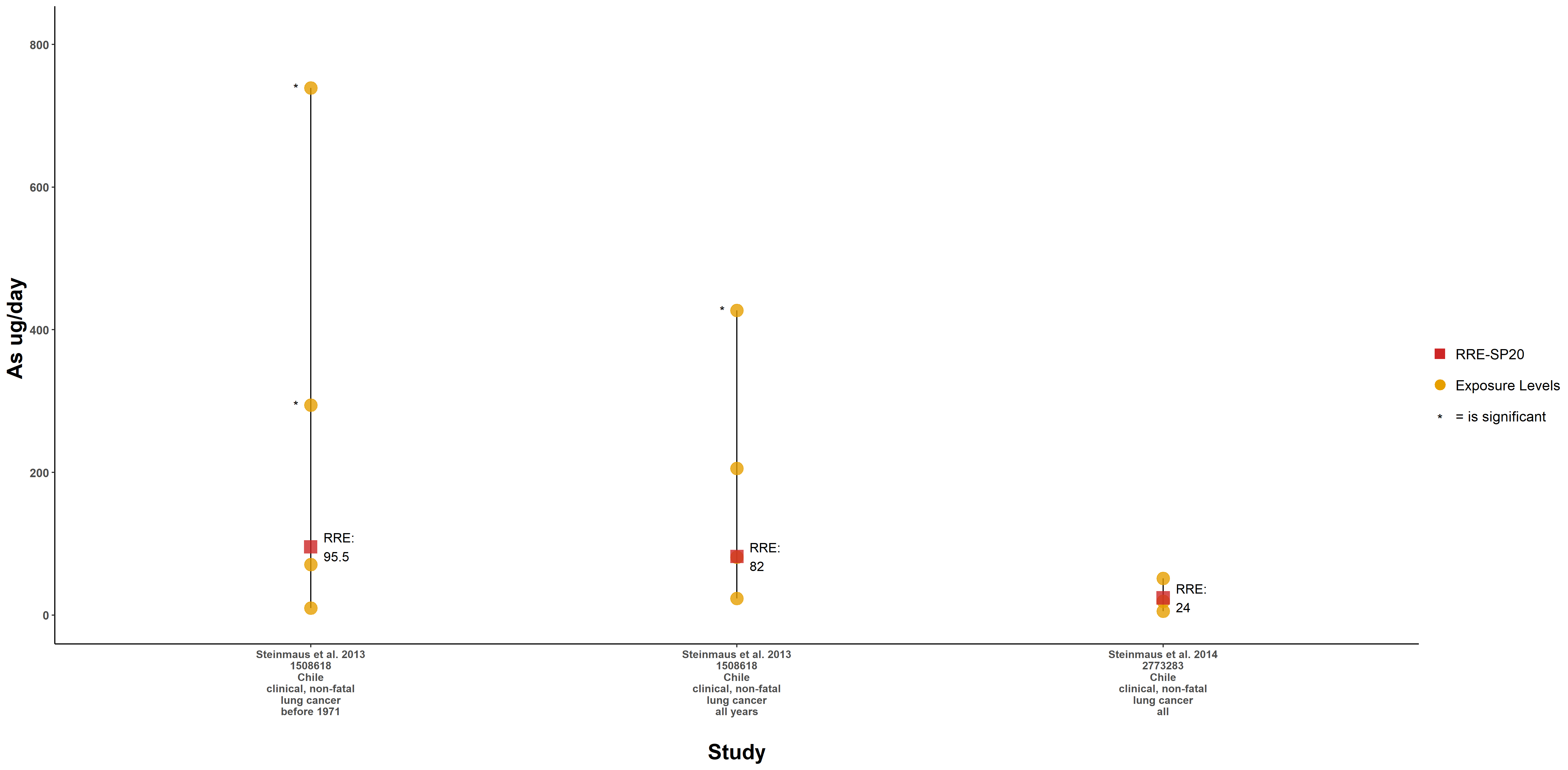


Figure S-25B. Exposure levels and RRE-SP_20_ for lung cancer using daily intake.


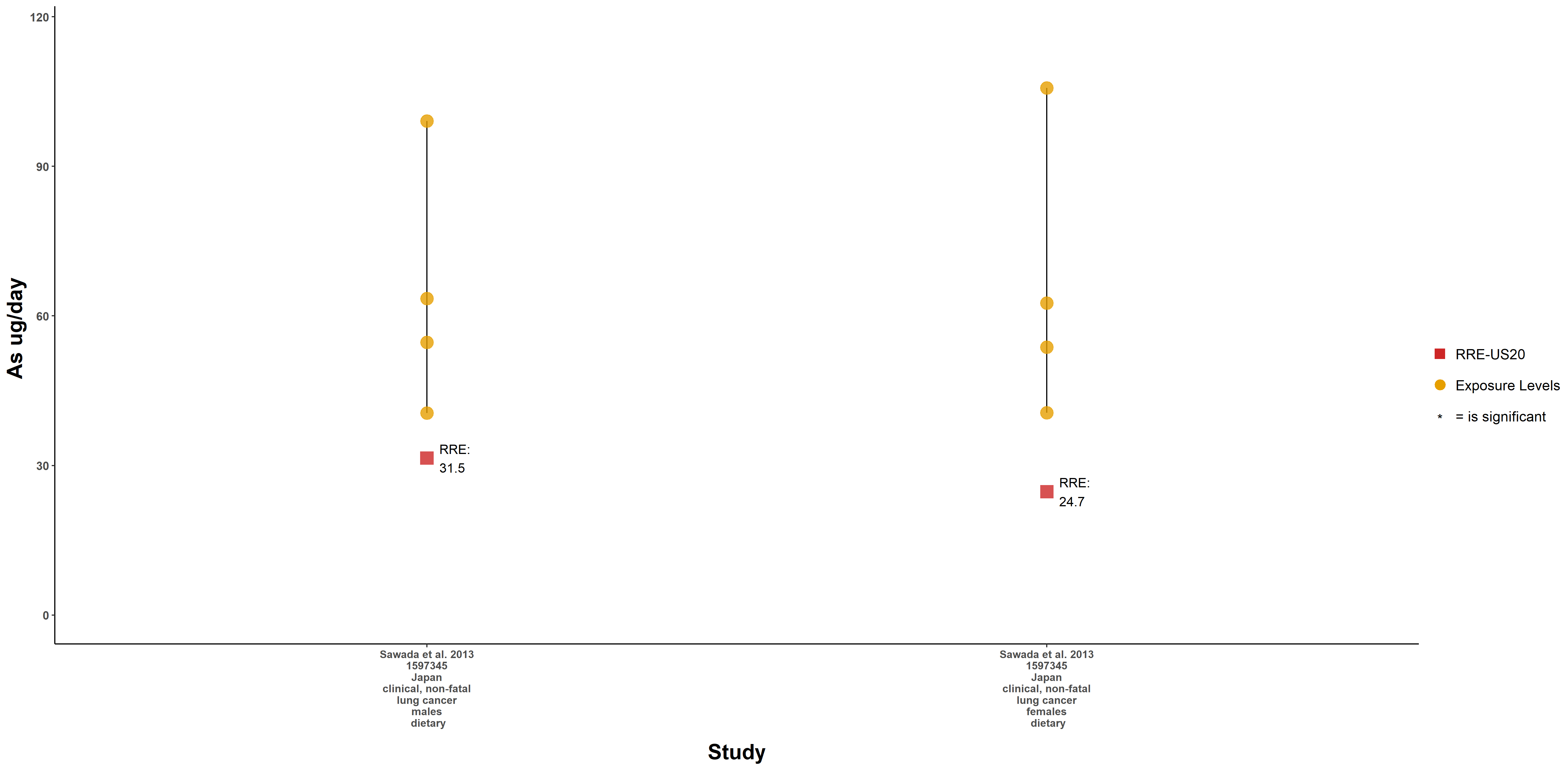


Figure S-26A. Exposure levels and RRE-US_20_ for lung cancer using dietary intake.


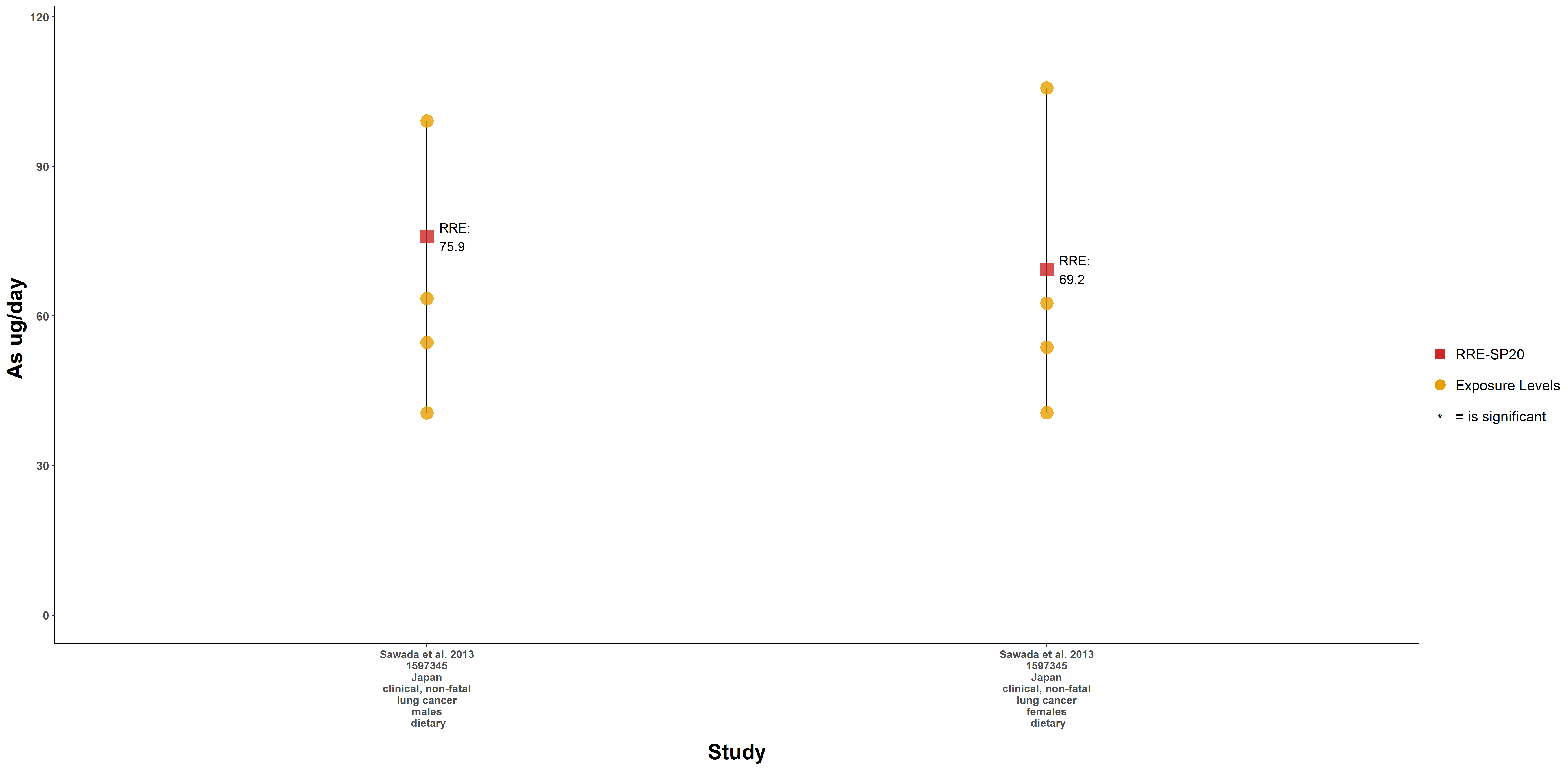


Figure S-26B. Exposure levels and RRE-SP_20_ for lung cancer using dietary intake.


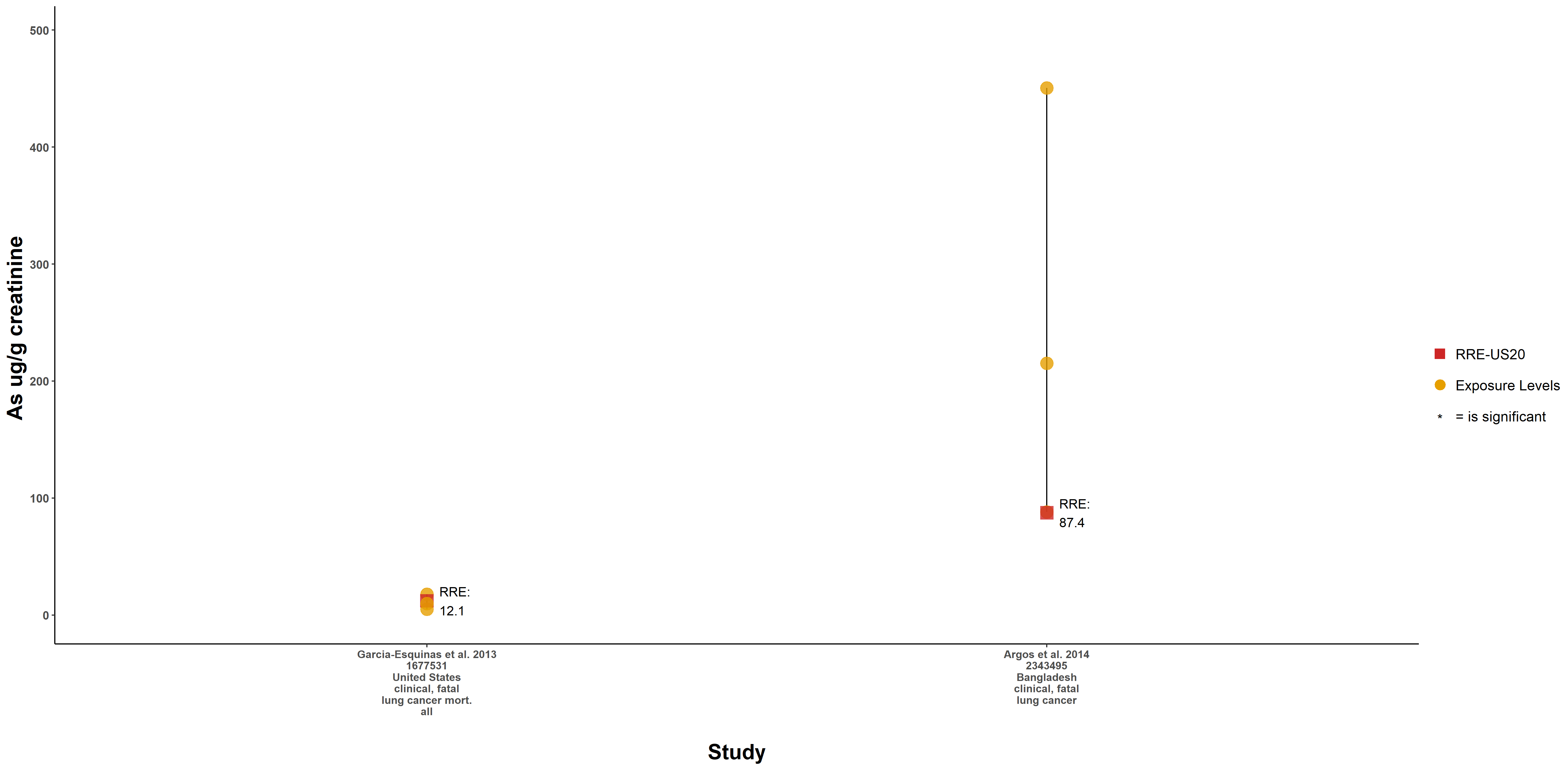


Figure S-27A. Exposure levels and RRE-US_20_ for lung cancer using creatinine adjusted urine concentration.


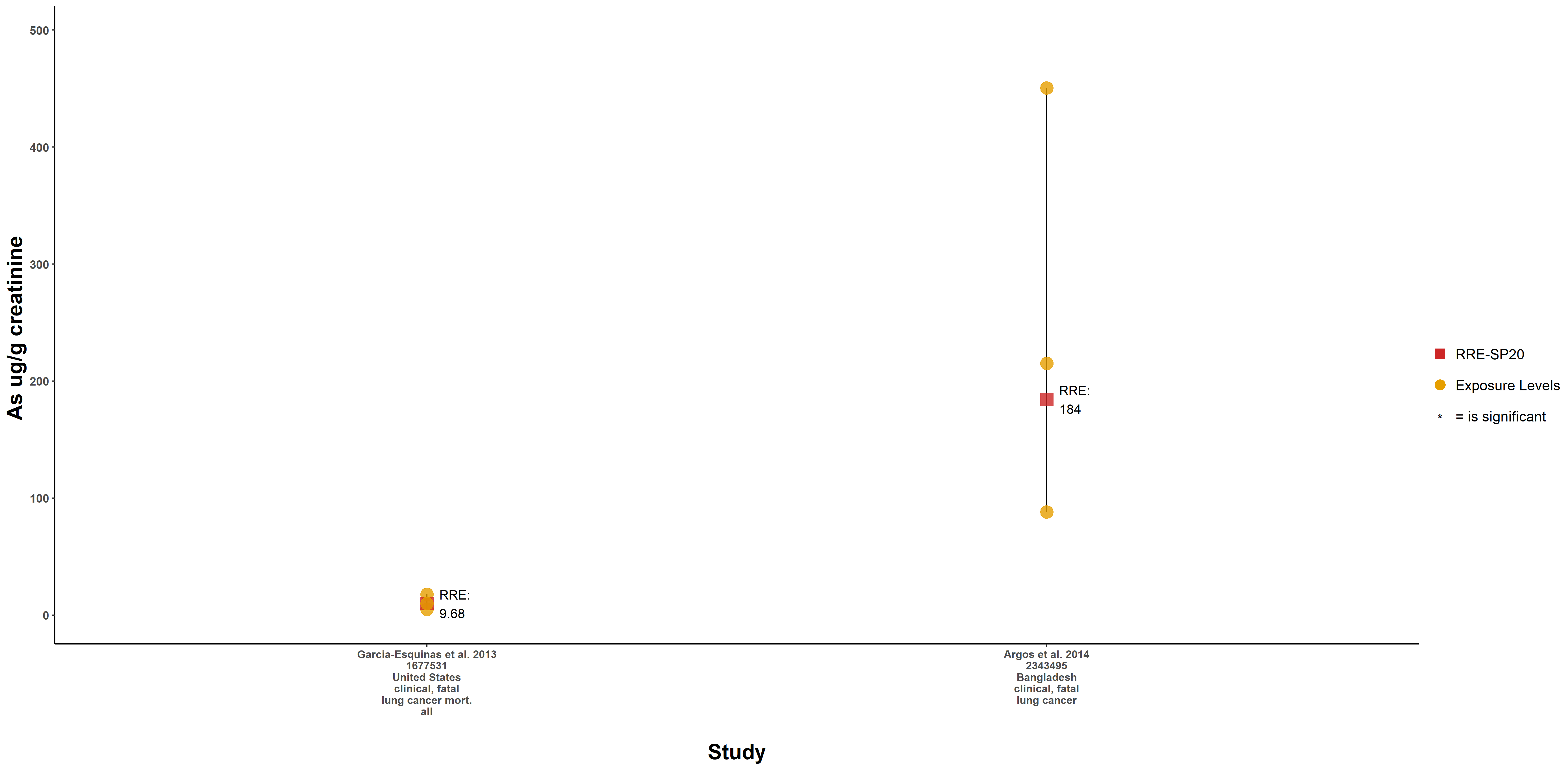


Figure S-27B. Exposure levels and RRE-SP_20_ for lung cancer using creatinine adjusted urine concentration.


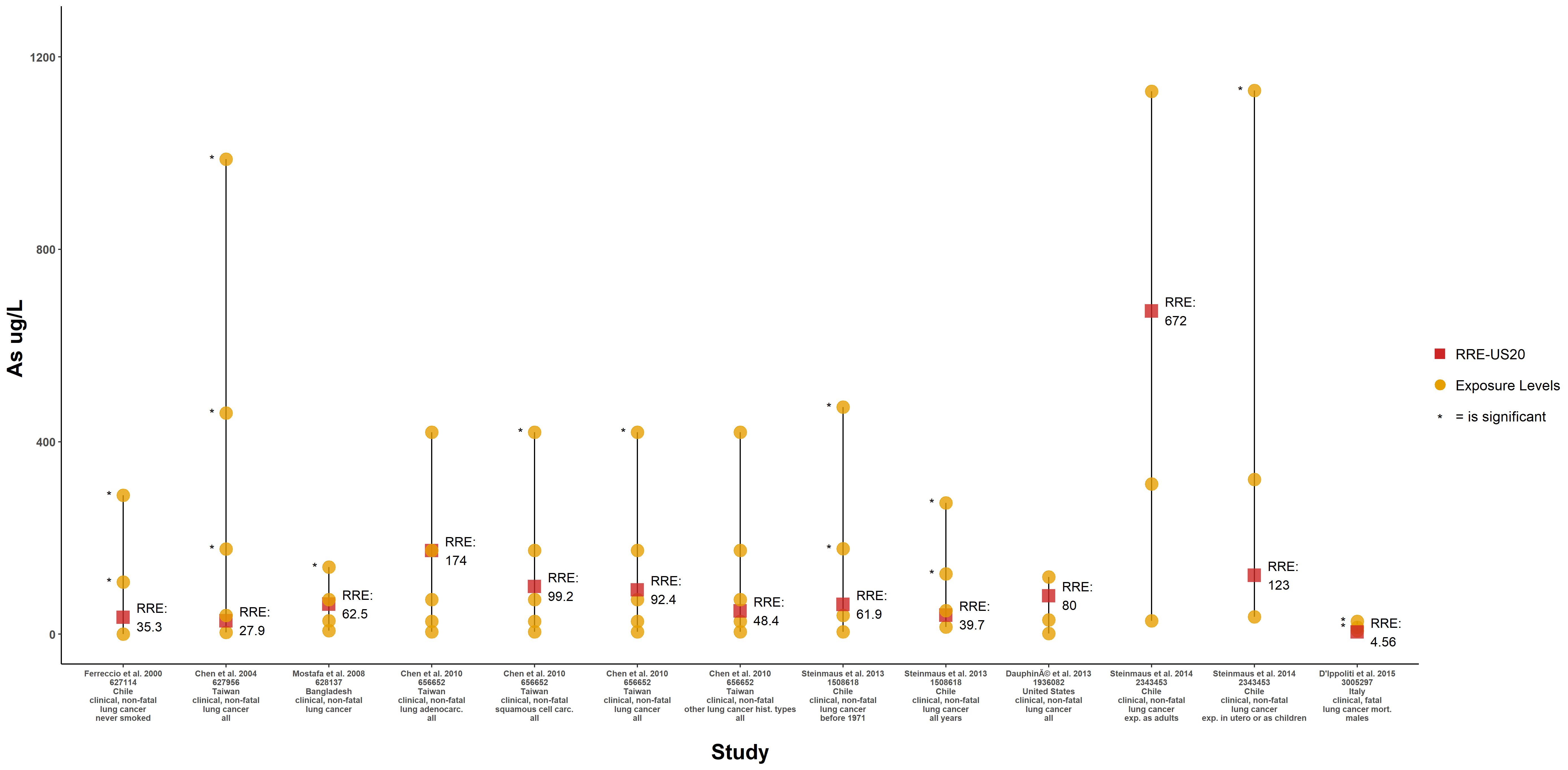


Figure S-28A. Exposure levels and RRE-US_20_ for lung cancer using water concentration.


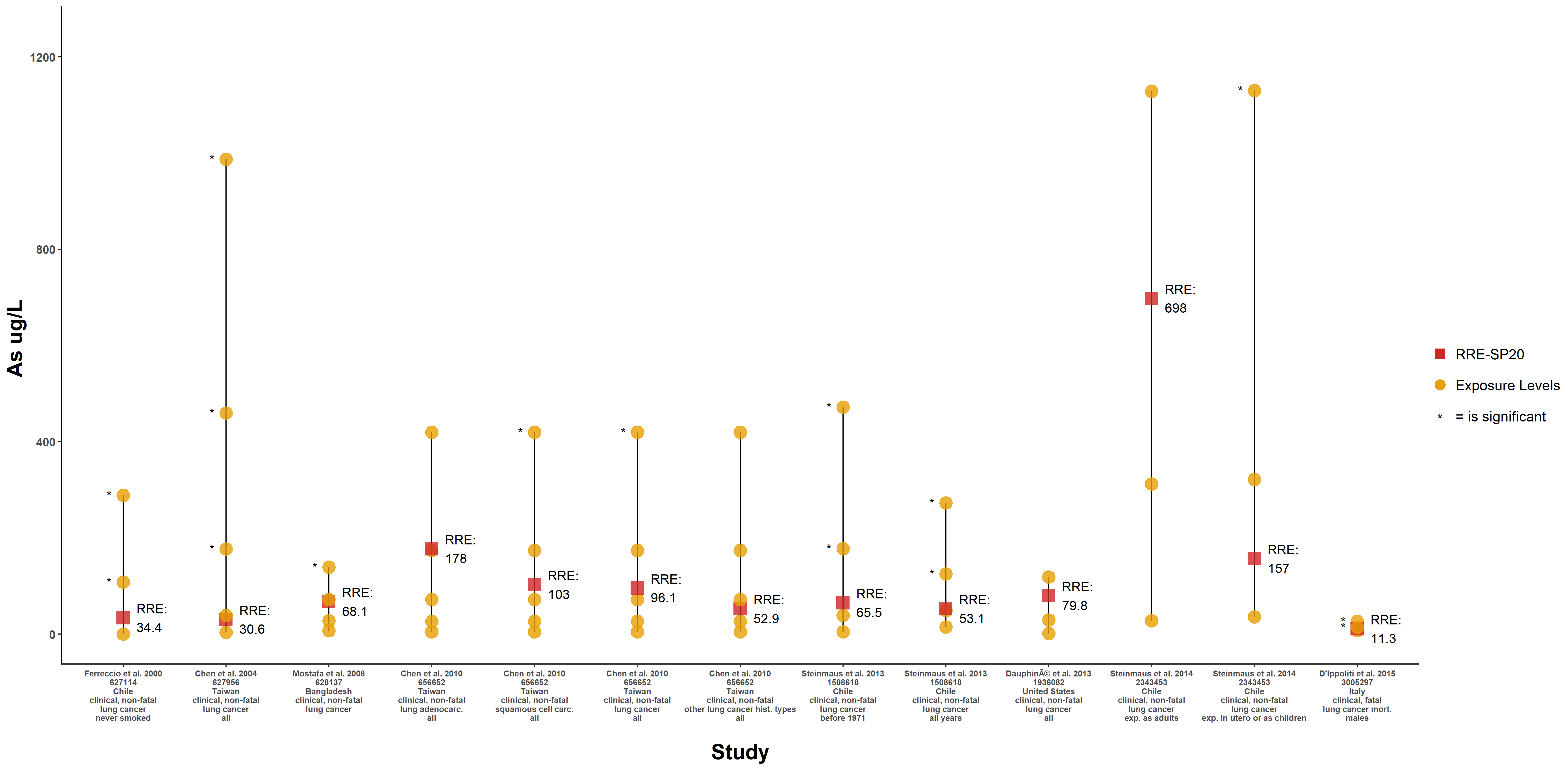


Figure S-28B. Exposure levels and RRE-SP_20_ for lung cancer using water concentration.

Table S-34A. Summary of RRE-US_20_s and RRB-US for lung cancer studies


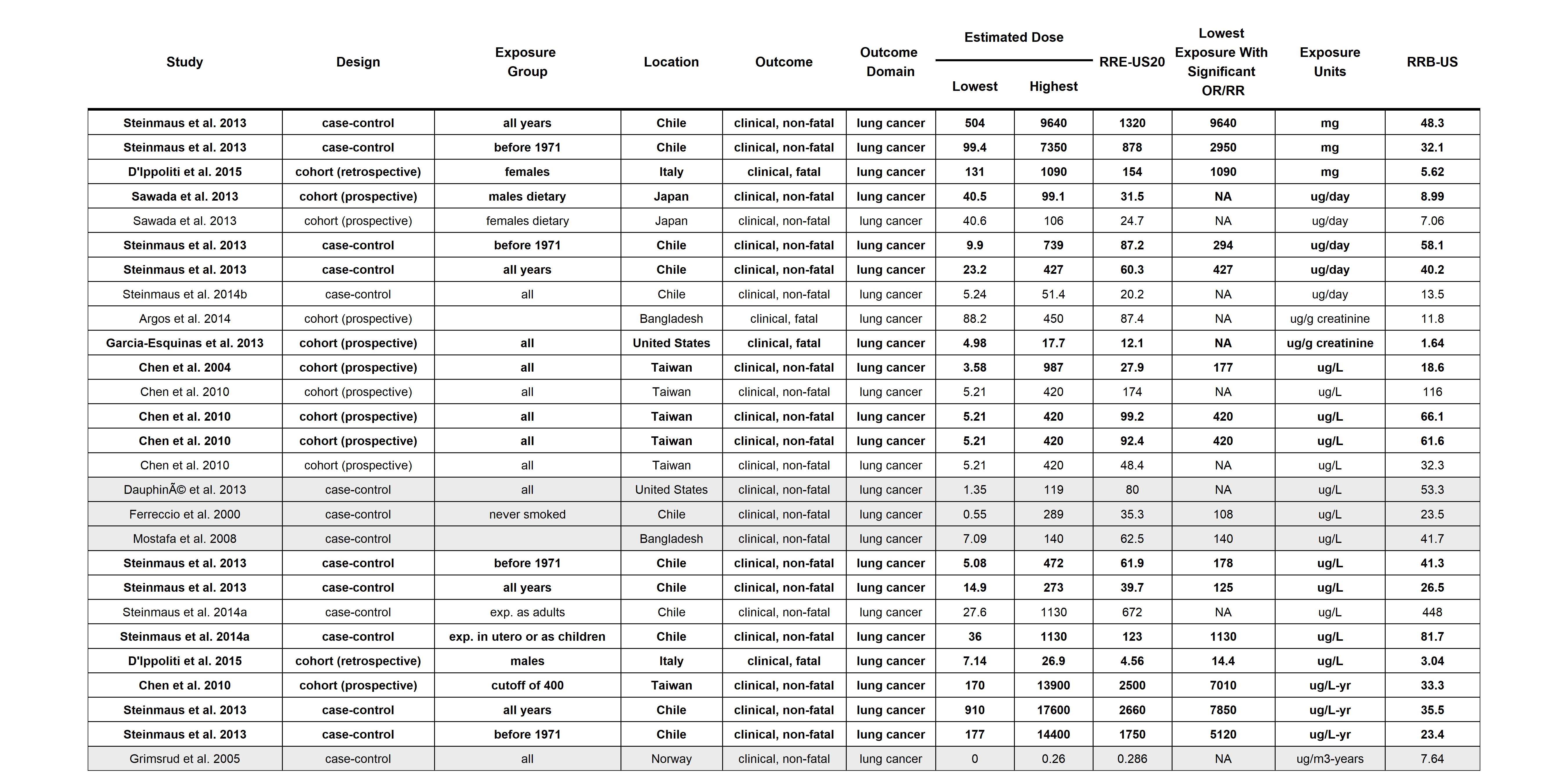


RRB-US refers to the ratio of RRE-US_20_ to an estimated U.S. background exposure level. Shaded cells indicate that authors did not report exposure-response trends. Bold rows indicate that authors reported a significant exposure-response trend (*p* <0.05)

Table S-34B. Summary of RRE-SP_20_s and RRE-SP for lung cancer studies


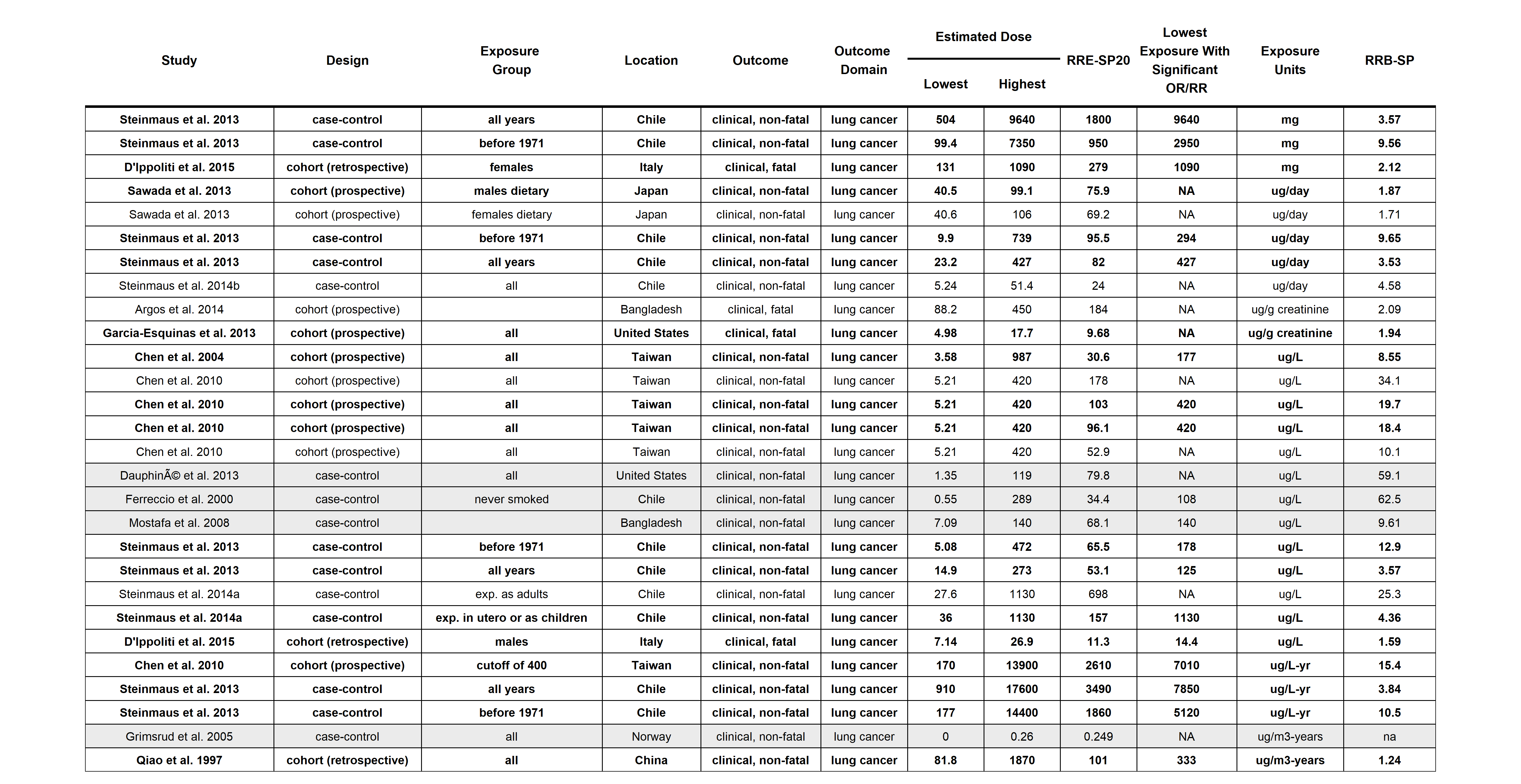


RRB-SP refers to the ratio of RRE-SP_20_ to the reported or estimated background exposure level for the study referent group. Shaded cells indicate that authors did not report exposure-response trends. Bold rows indicate that authors reported a significant exposure-response trend (p<0.05)

#### Nonmalignant Respiratory Disease Exposure-Response Modeling Results

The analysis of arsenic exposure response on nonmalignant respiratory disease evaluated 5 datasets from two studies by [D'Ippoliti et al. (2015)](#_ENREF_92) and [Argos et al. (2014)](#_ENREF_17) that evaluated chronic obstructive pulmonary disease (COPD) mortality and arsenic exposure. A summary of datasets modeled identifying the study design, location, and exposure metric is provided in Table S-35 below. A breakdown of the exposure levels and RRE_20_ estimates are provided for each exposure metric in Figure S-29 - Figure S-31. Finally, RRE_20_ summary tables for all exposures are provided in Table S-36.


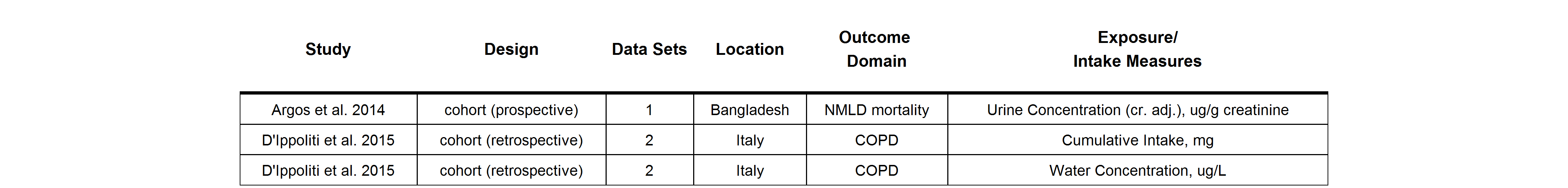
Table S-35. Summary of datasets considered in nonmalignant respiratory disease exposure-response RRB analysis by exposure metric


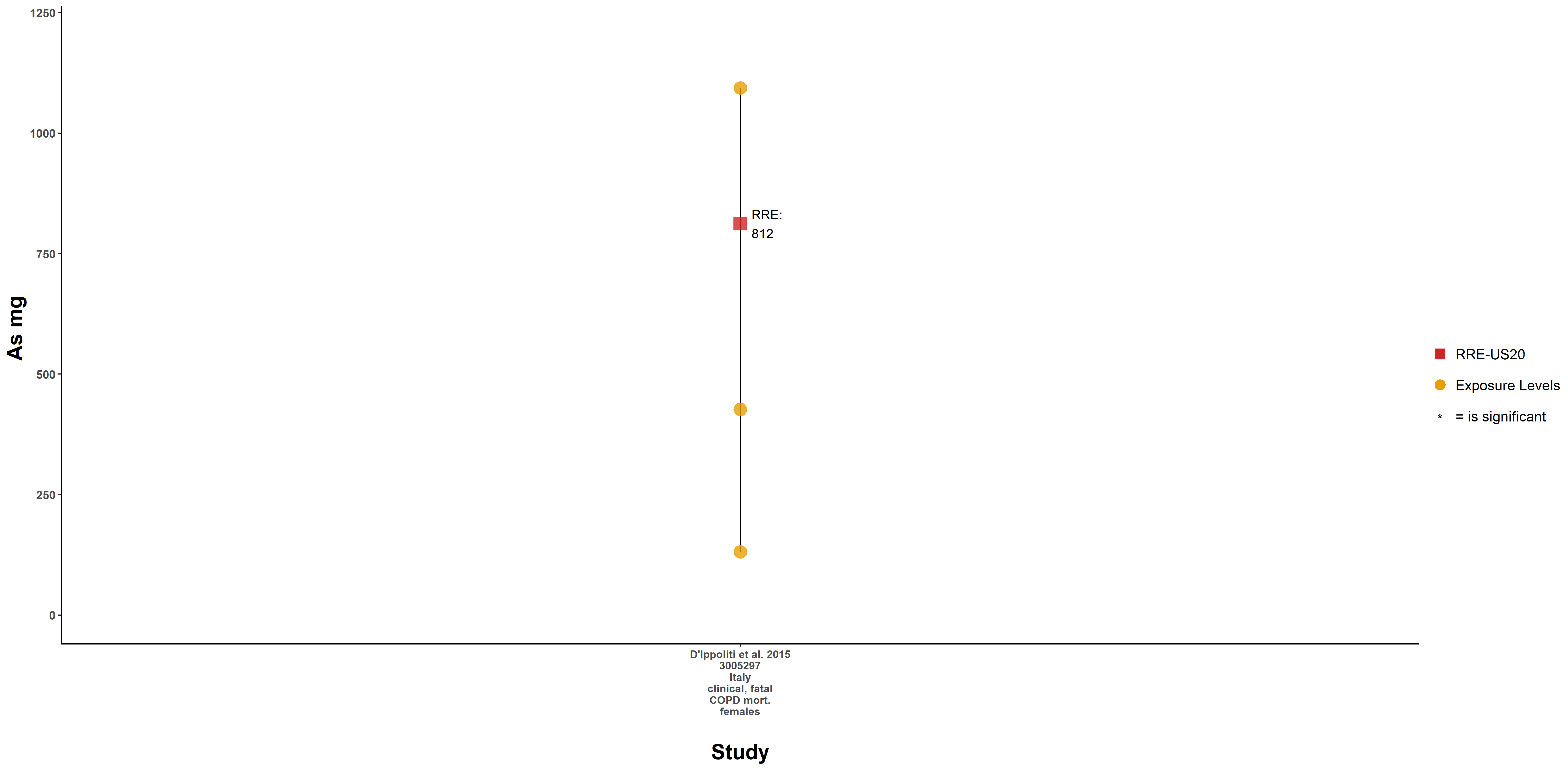


Figure S-29A. Exposure levels and RRE-US_20_ for nonmalignant respiratory disease using cumulative intake.


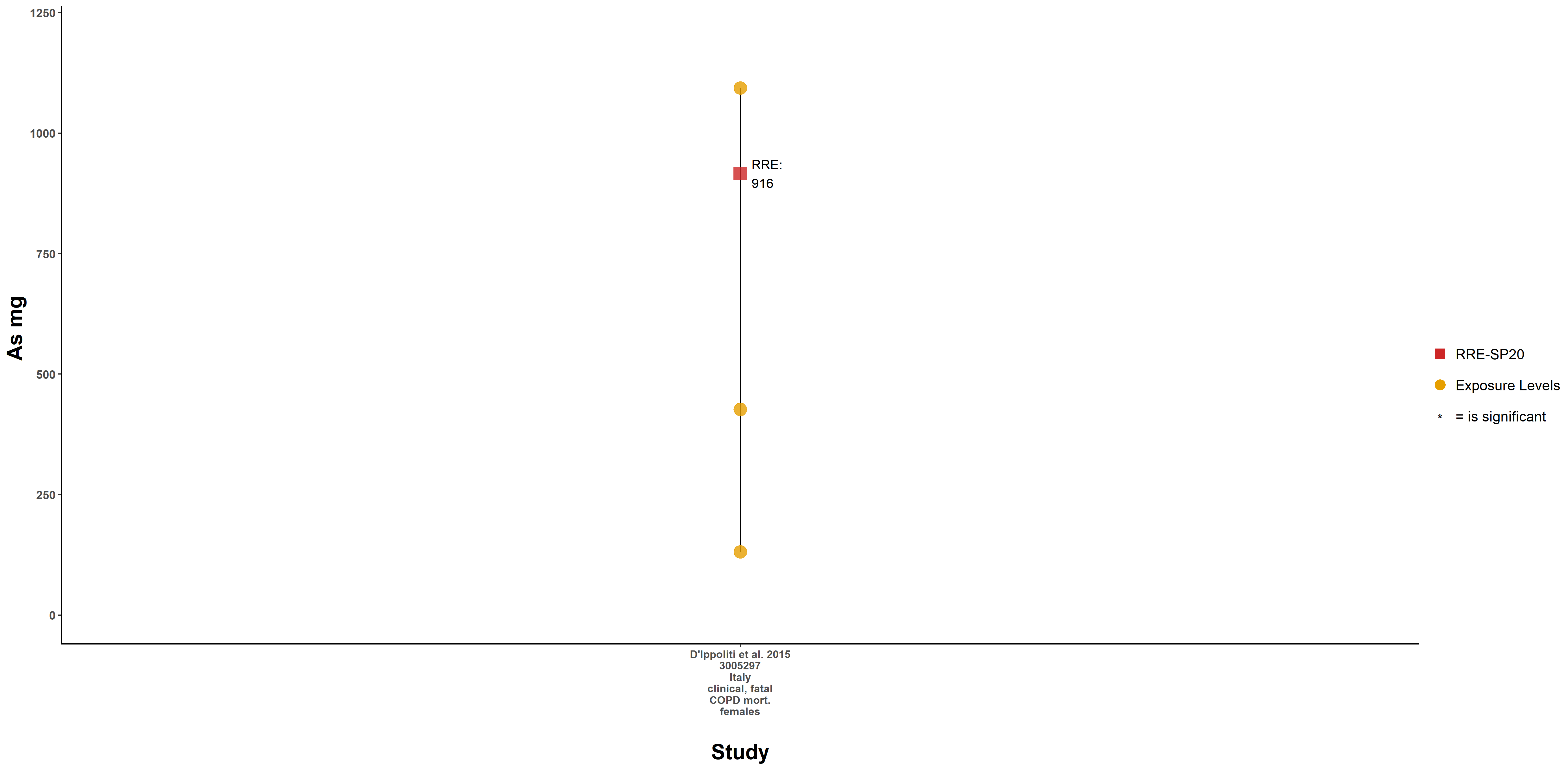


Figure S-29B. Exposure levels and RRE-SP_20_ for nonmalignant respiratory disease using cumulative intake.


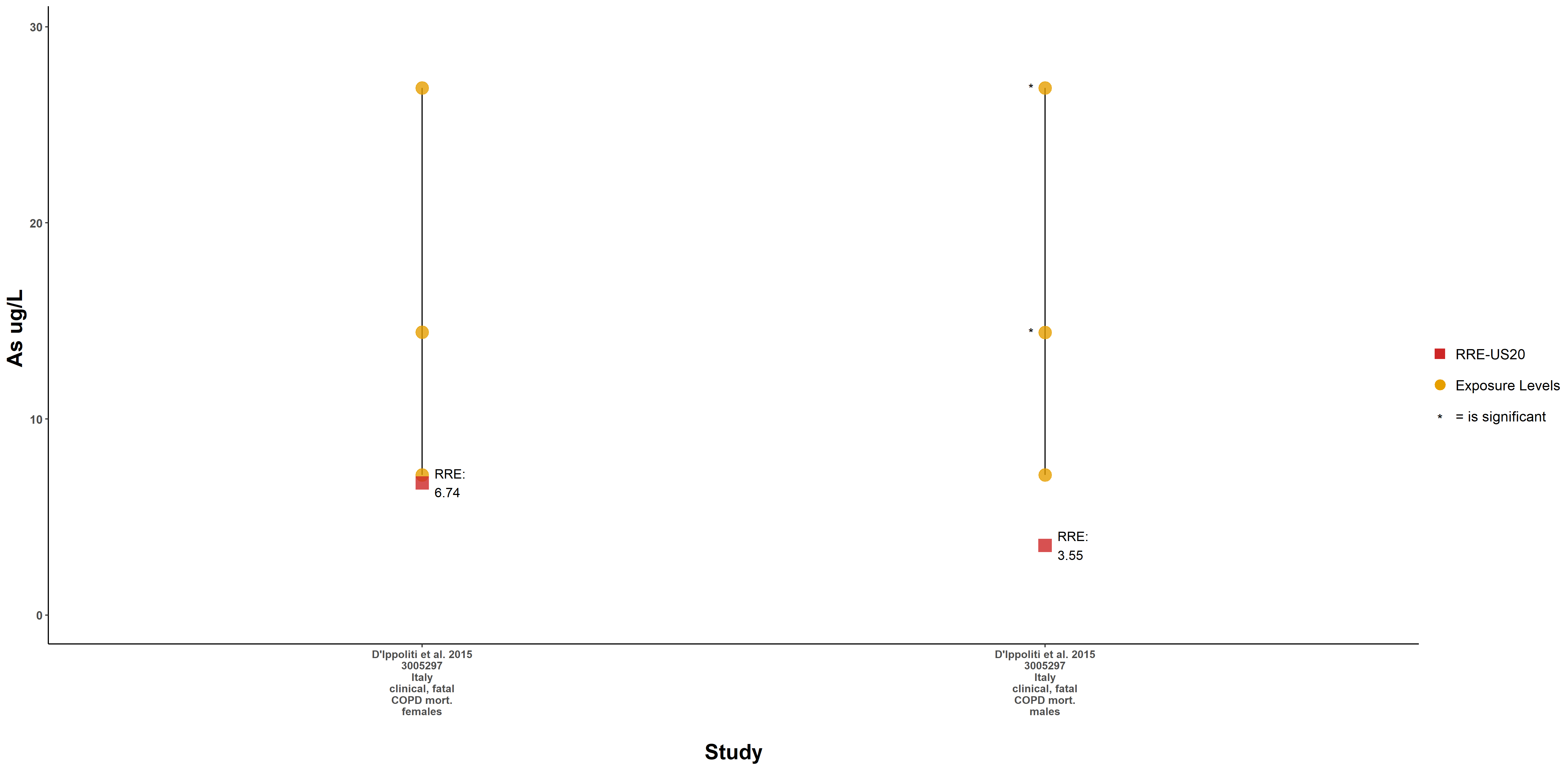


Figure S-30A. Exposure levels and RRE-US_20_ for nonmalignant respiratory disease using water concentration.


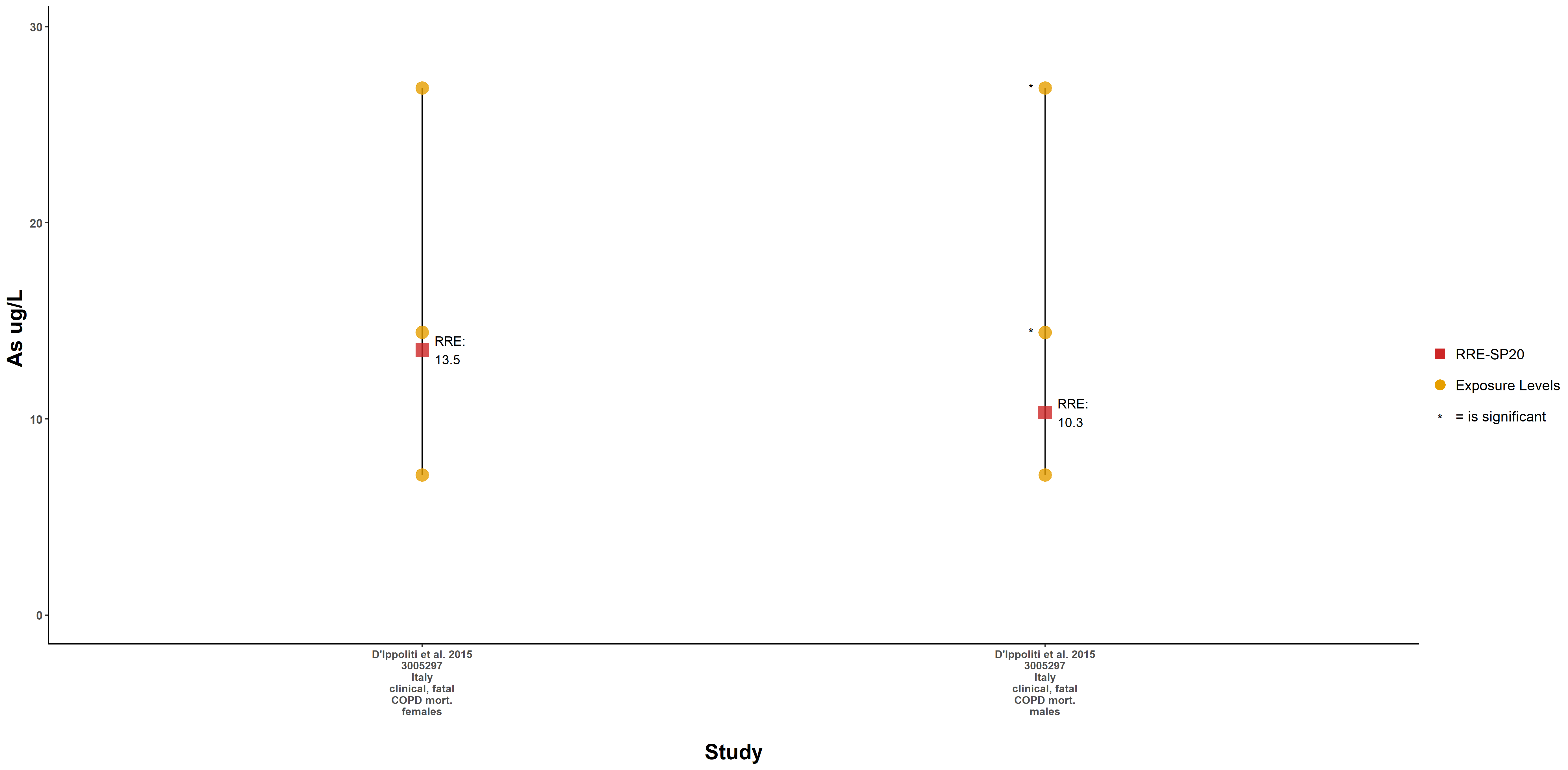


Figure S-30B. Exposure levels and RRE-SP_20_ for nonmalignant respiratory disease using water concentration.


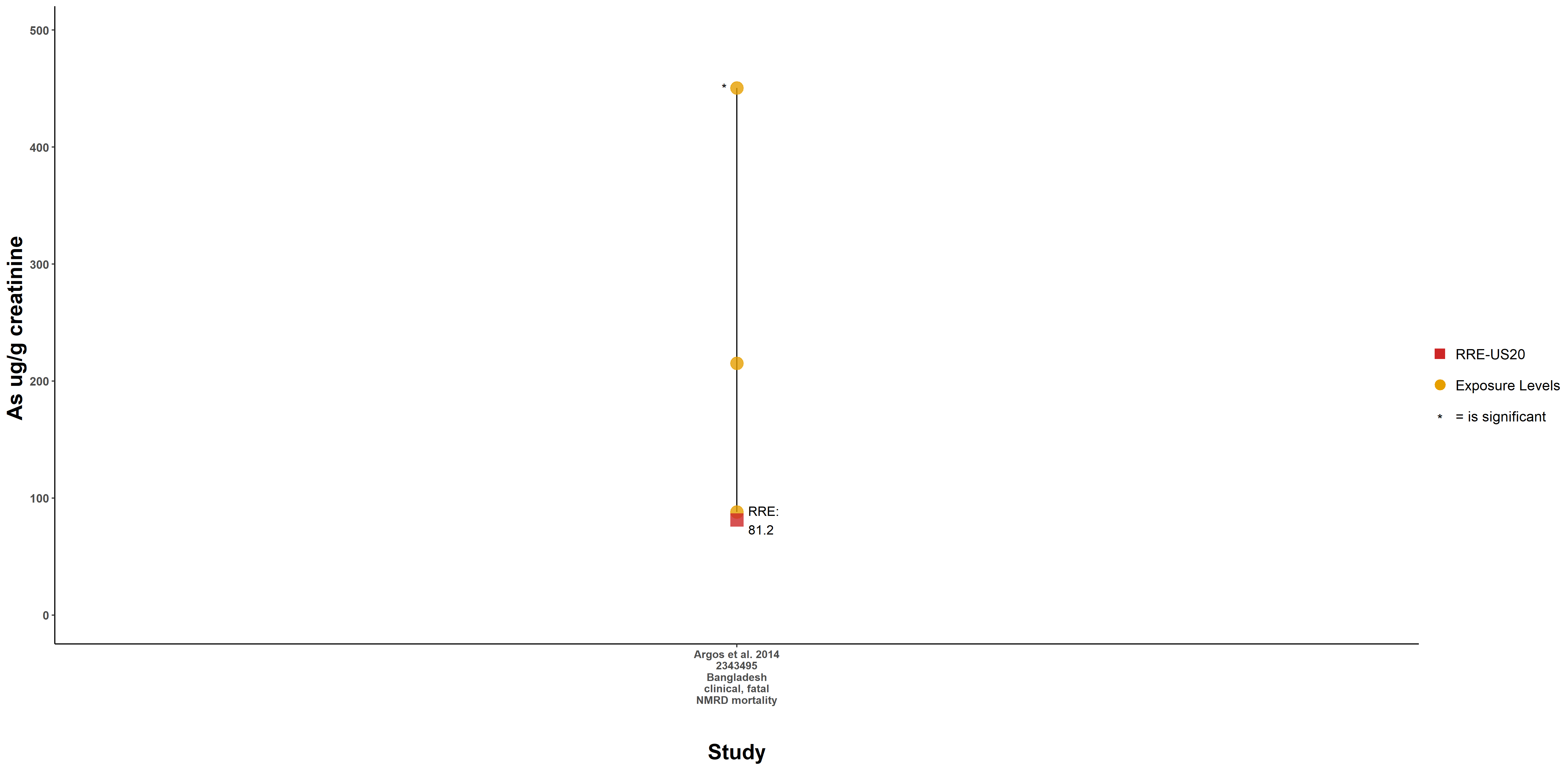


Figure S-31A. Exposure levels and RRE-US_20_ for nonmalignant respiratory disease using creatinine adjusted urine concentrations.


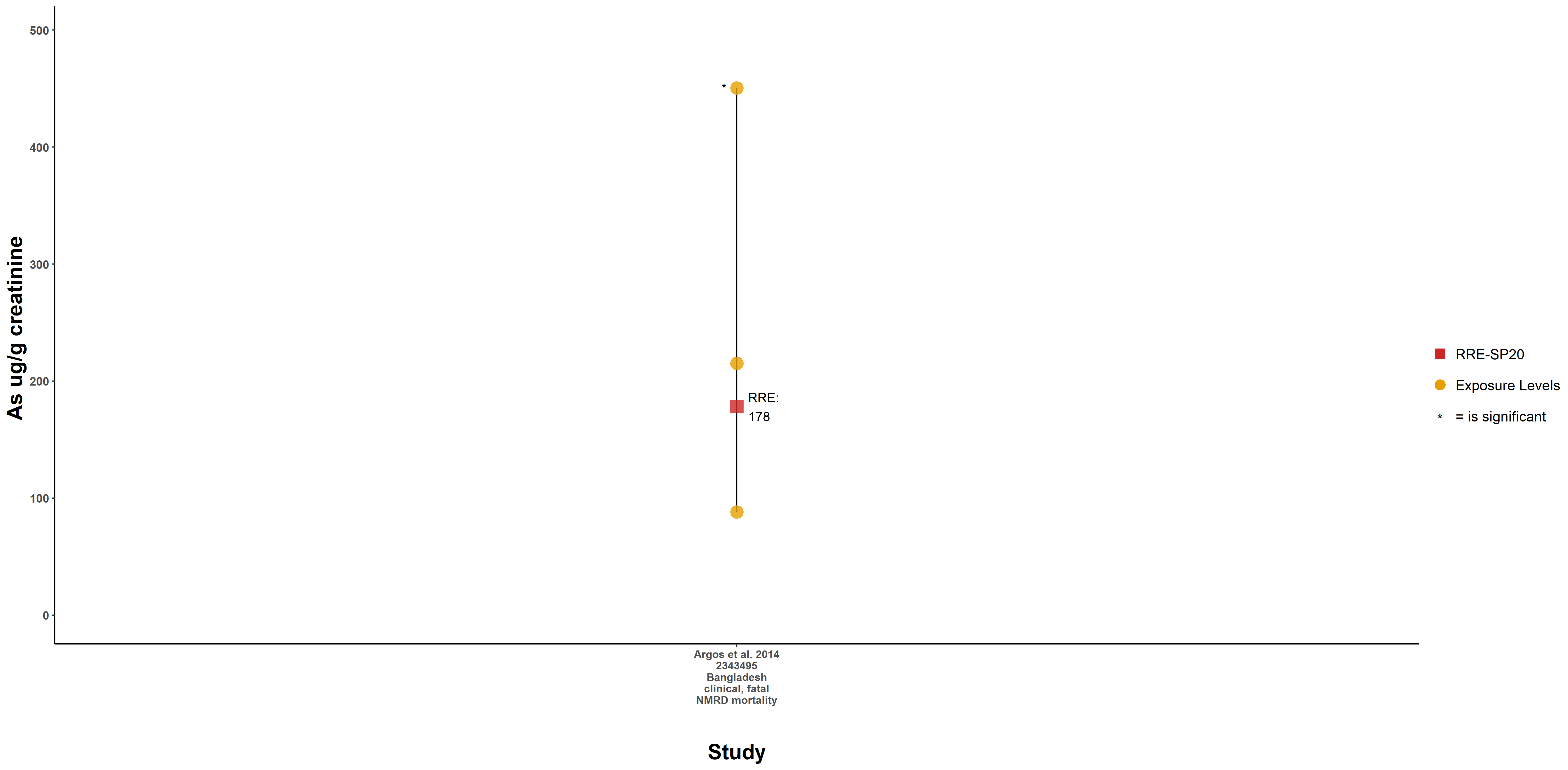


Figure S-31B. Exposure levels and RRE-SP_20_ for nonmalignant respiratory disease using creatinine adjusted urine concentrations.

Table S-36A. Summary of RRE-US_20_s and RRB-US for nonmalignant respiratory disease studies


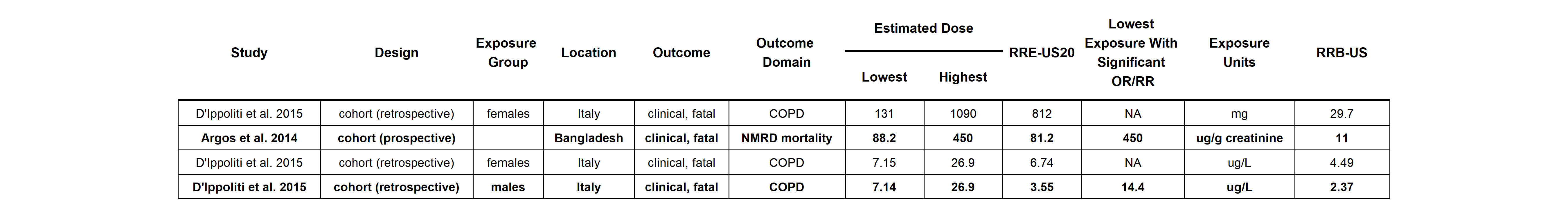


RRB-US refers to the ratio of RRE-US_20_ to an estimated U.S. background exposure level. Shaded cells indicate that authors did not report exposure-response trends. Bold rows indicate that authors reported a significant exposure-response trend (*p* <0.05)

Table S-36B. Summary of RRE-SP_20_s and RRB-SP for nonmalignant respiratory disease studies


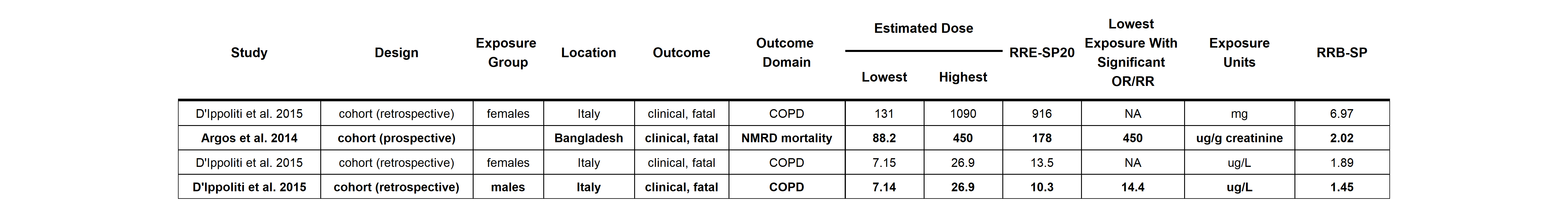


RRB-SP refers to the ratio of RRE-SP_20_ to the reported or estimated background exposure level for the study referent group. Shaded cells indicate that authors did not report exposure-response trends . Bold rows indicate that authors reported a significant exposure-response trend (*p* <0.05)

#### Pregnancy Outcomes Exposure-Response Modeling Results

The analysis of arsenic exposure response on pregnancy outcomes evaluated 6 datasets from 3 peer reviewed studies that included endpoints such as miscarriage, stillbirths, fetal loss, and infant mortality. A summary of datasets modeled identifying the study design, location, exposure metric and outcome domain is provided in Table S-37. All datasets generated models that met our initial model selection criteria. Five of the selected models had RRE_20_ estimates ranging within a factor of three of the central estimates for the lowest or highest dose group and are presented in the results below. A breakdown of the exposure levels and RRE_20_ estimates is provided for each exposure metric in Figure S-30–Figure S-32. Finally, an RRE_20_ summary table for all exposures is provided in Table S-38.

Table S-37. Summary of datasets considered in pregnancy outcomes exposure-response RRB analysis by exposure metric


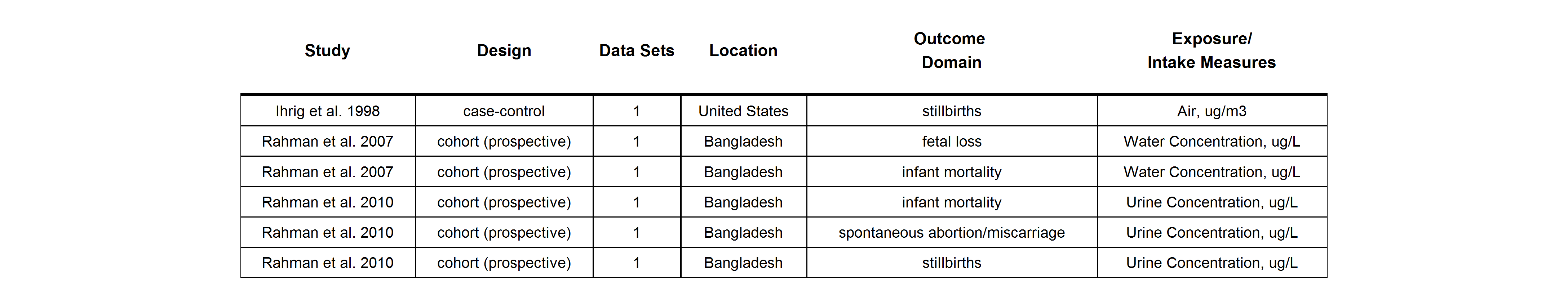


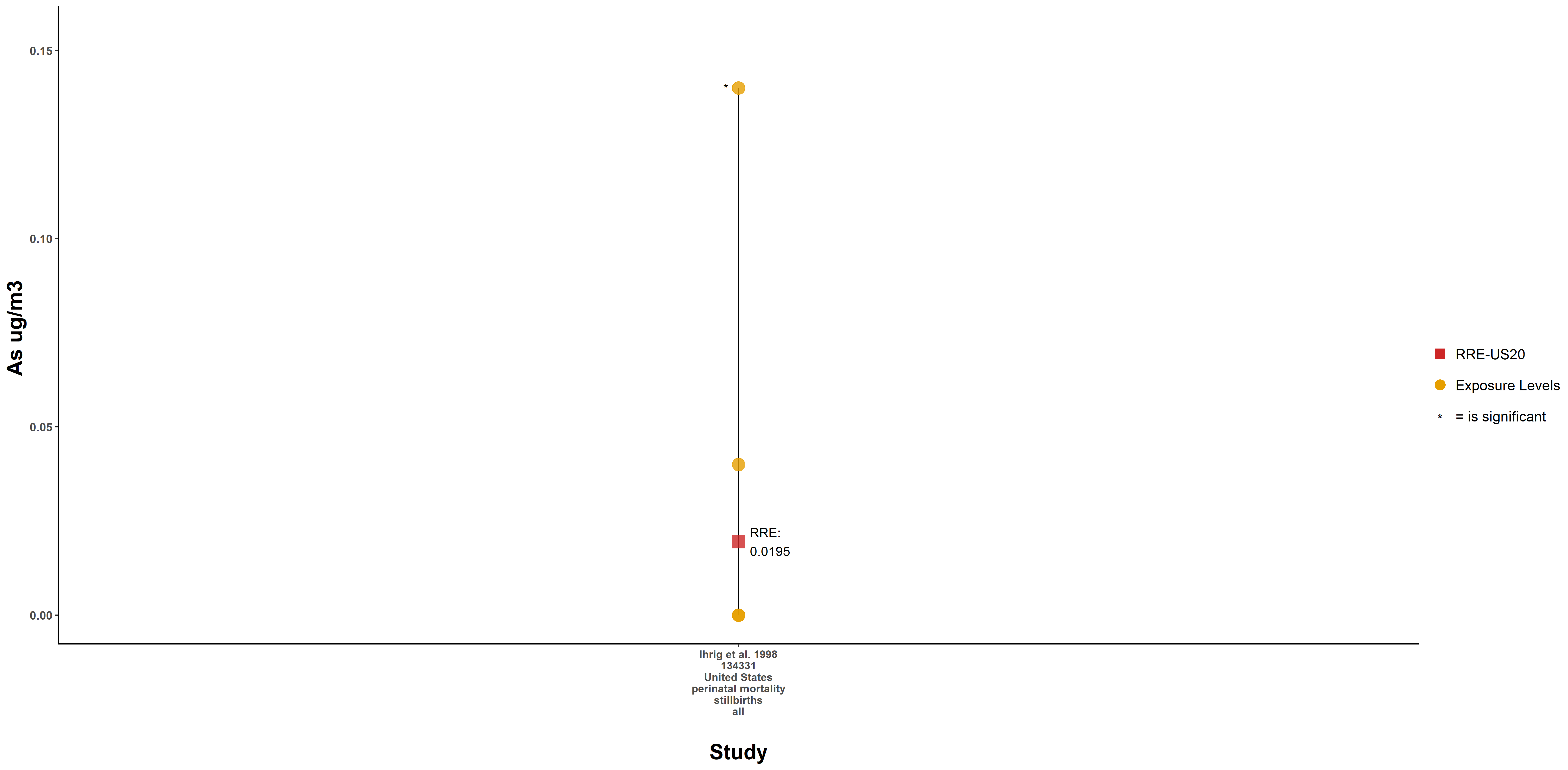


Figure S-32A. Exposure levels and RRE-US_20_ for pregnancy outcomes using air concentration.


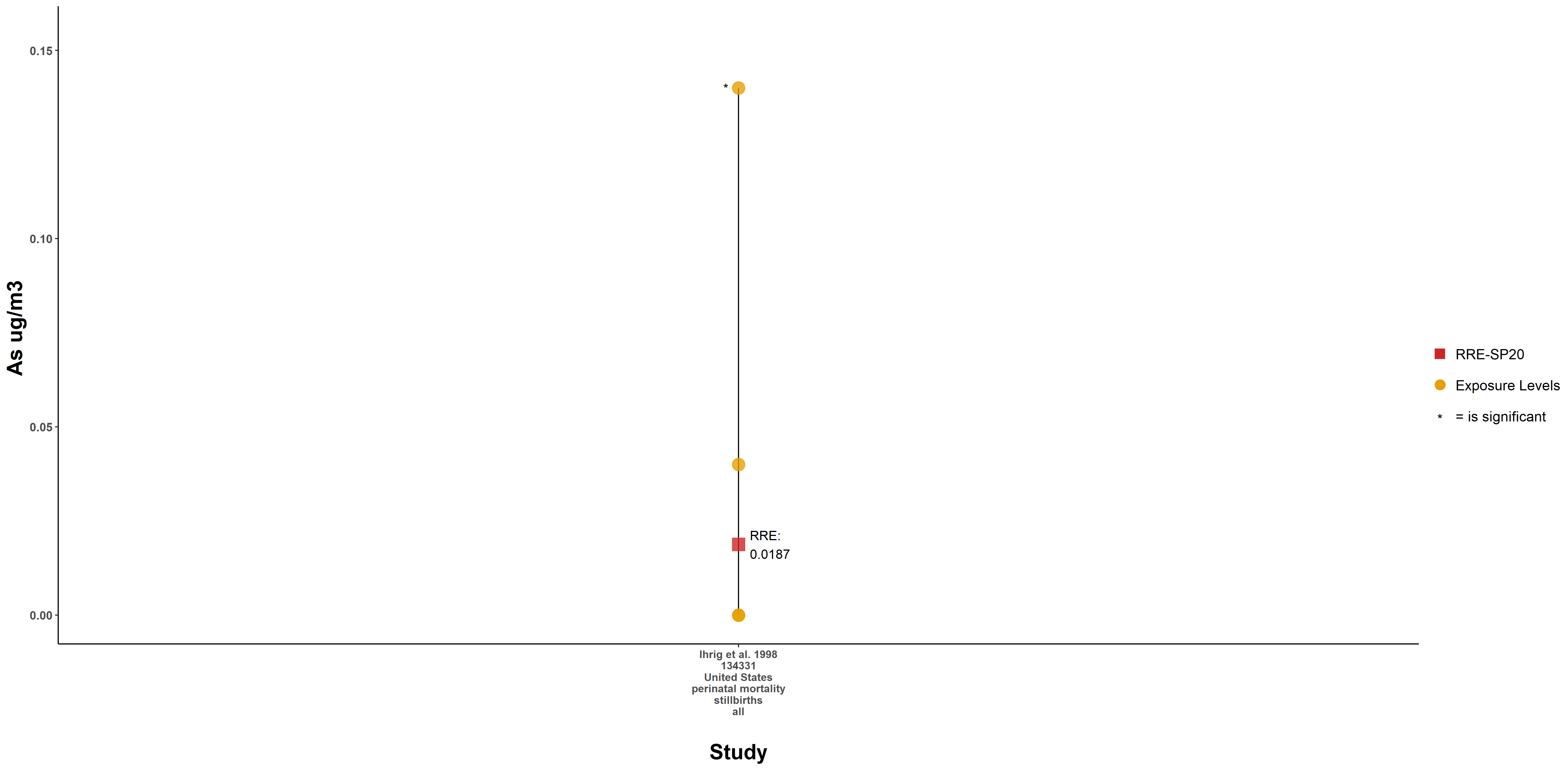


Figure S-32B. Exposure levels and RRE-SP_20_ for pregnancy outcomes using air concentration.


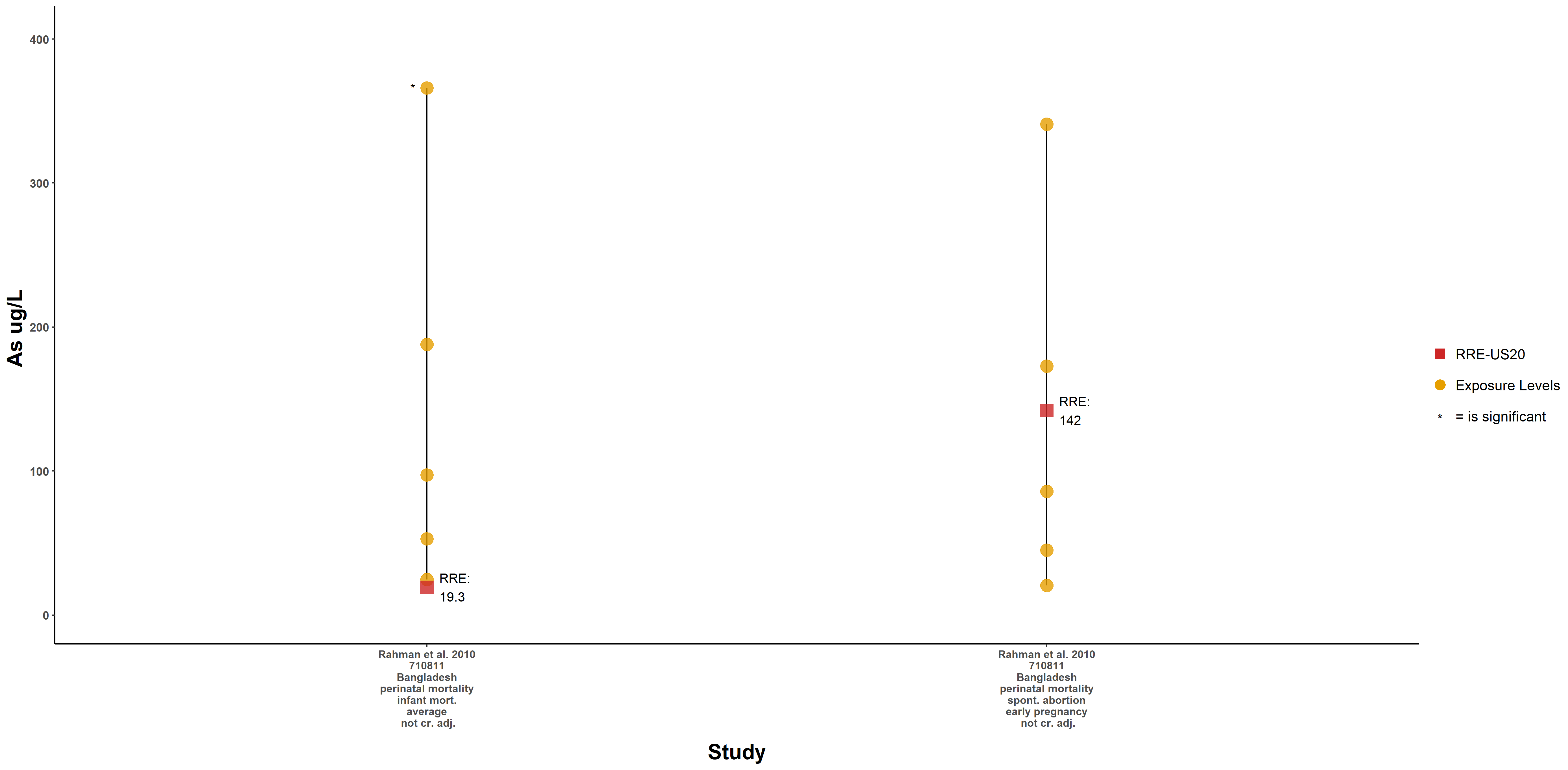


Figure S-33A. Exposure levels and RRE-US_20_ for pregnancy outcomes using urine concentration.


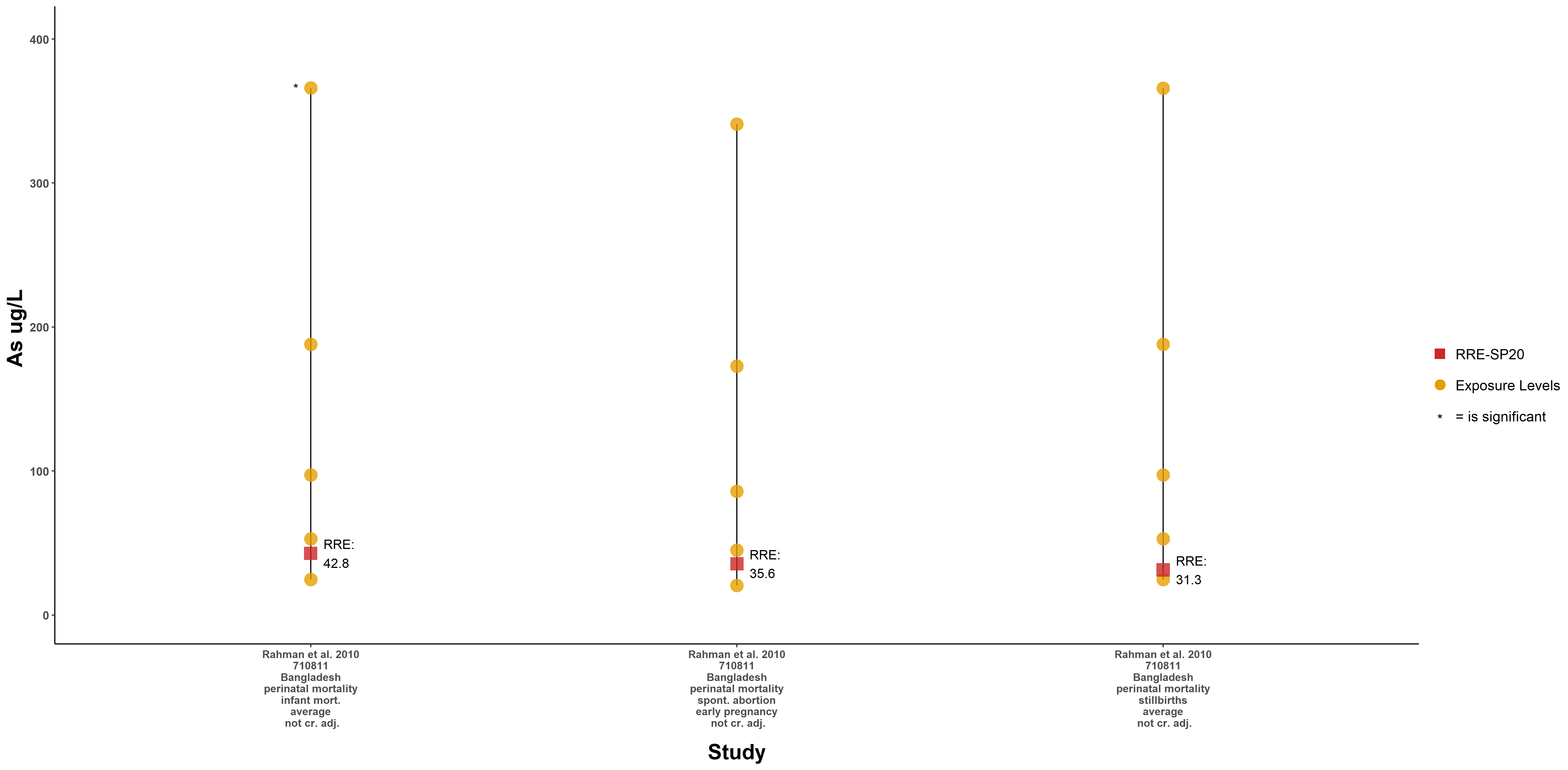


Figure S-33B. Exposure levels and RRE-SP_20_ for pregnancy outcomes using urine concentration.


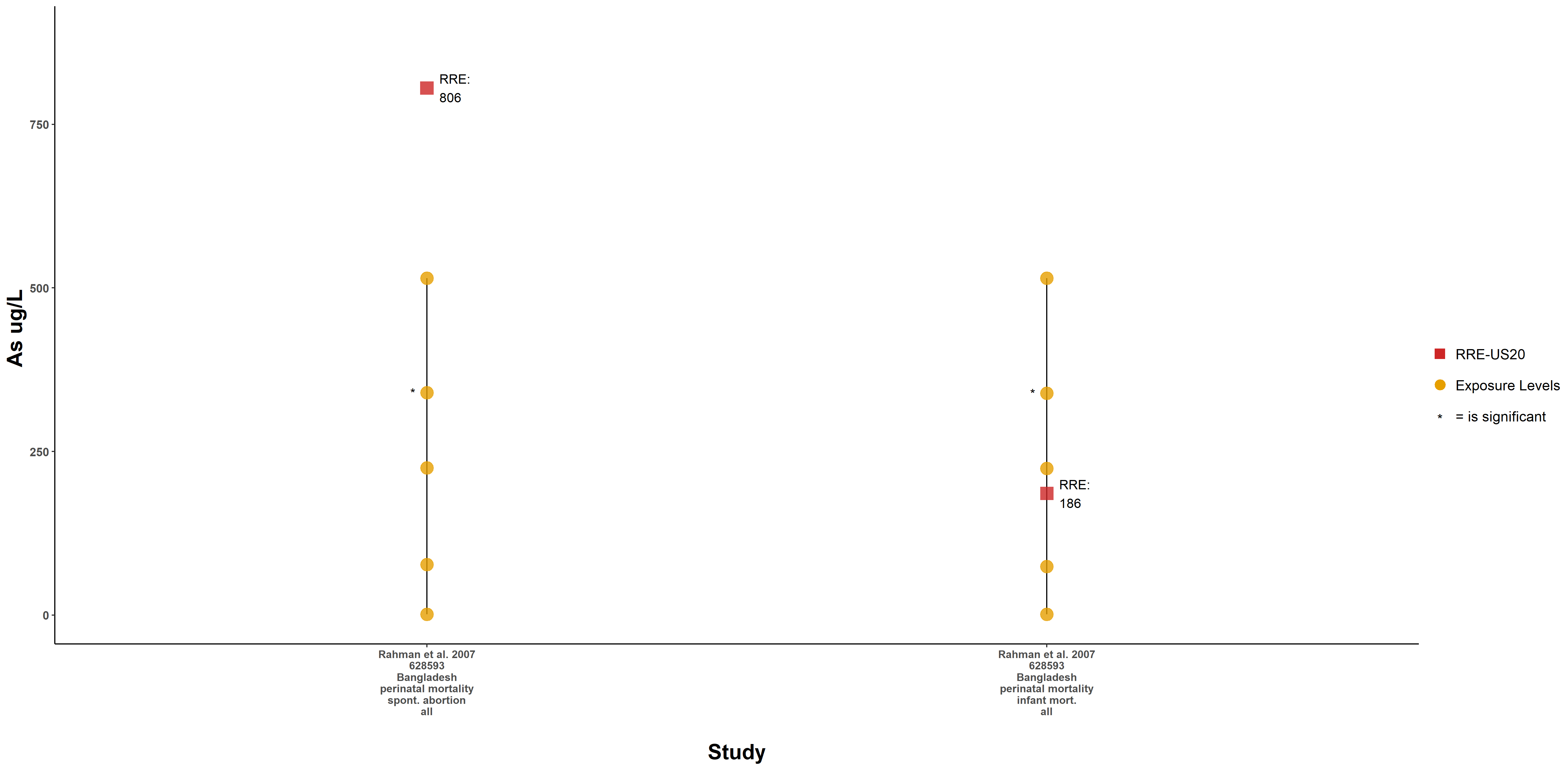


Figure S-34A. Exposure levels and RRE-US_20_ for pregnancy outcomes using water concentration.


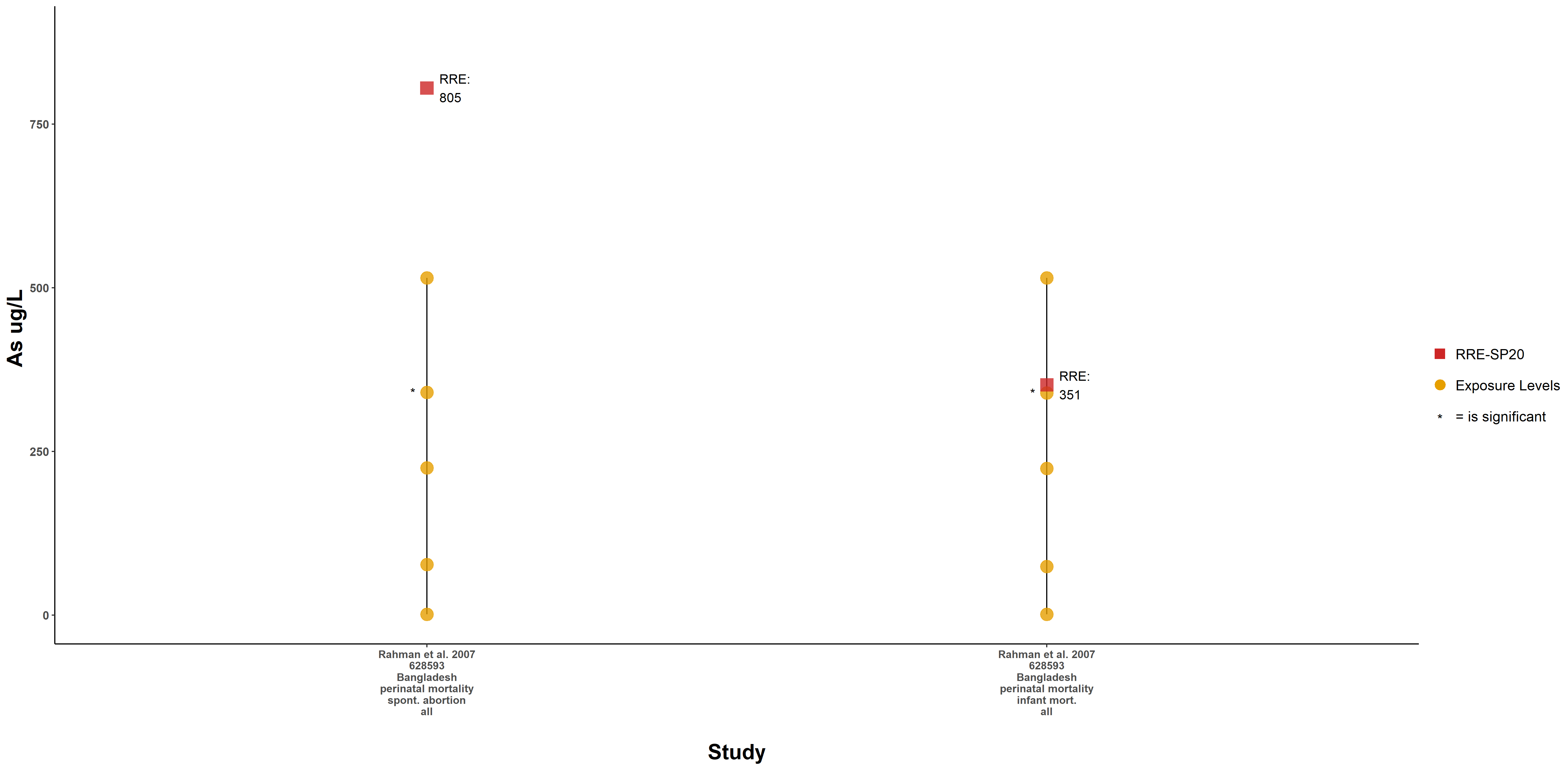


Figure S-34B. Exposure levels and RRE-SP_20_ for pregnancy outcomes using water concentration.

Table S-38A. Summary of RRE-US_20_s and RRB-US for pregnancy outcomes studies


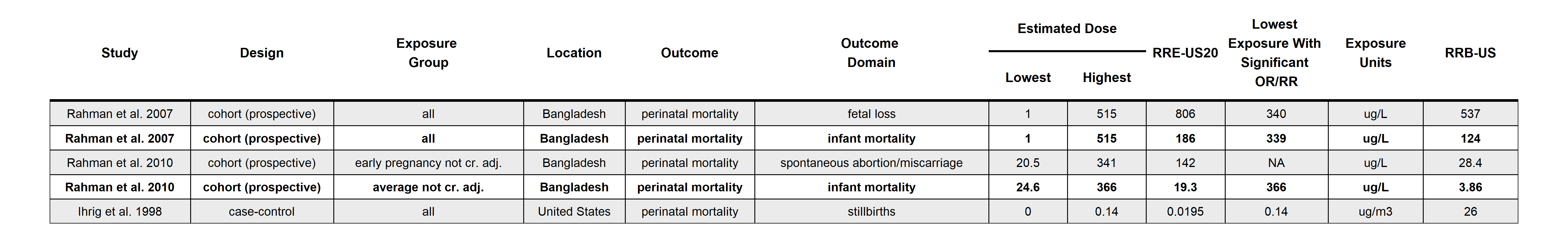


RRB-US refers to the ratio of RRE-US_20_ to an estimated U.S. background exposure level. Shaded cells indicate that authors did not report exposure-response trends. Bold rows indicate that authors reported a significant exposure-response trend (*p* < 0.05)

Table S-38B. Summary of RRE-SP_20_s and RRB-SP for pregnancy outcomes studies


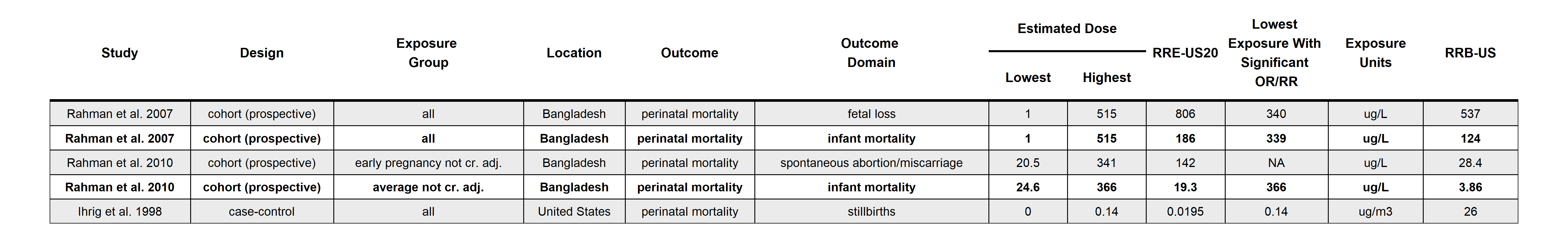


RRB-SP refers to the ratio of RRE-SP_20_ to the reported or estimated background exposure level for the study referent group. Shaded cells indicate that authors did not report exposure-response trends. Bold rows indicate that authors reported a significant exposure-response trend (*p* < 0.05)

#### Renal Cancer Exposure-Response Modeling Results

The analysis of arsenic exposure response on renal cancer evaluated 19 datasets from 6 peer reviewed studies that included endpoints such as transitional cell carcinomas of the renal pelvis and ureter. A summary of datasets modeled identifying the study design, location, exposure metric and outcome domain is provided in Table S-39 below. A breakdown of the exposure levels and RRE_20_ estimates are provided for each exposure metric in Figure S-35–Figure S-38. Finally, an RRE_20_ summary table for all exposures are provided in Table S-40.

Table S-39. Summary of datasets considered in renal cancer exposure-response RRB analysis by exposure metric


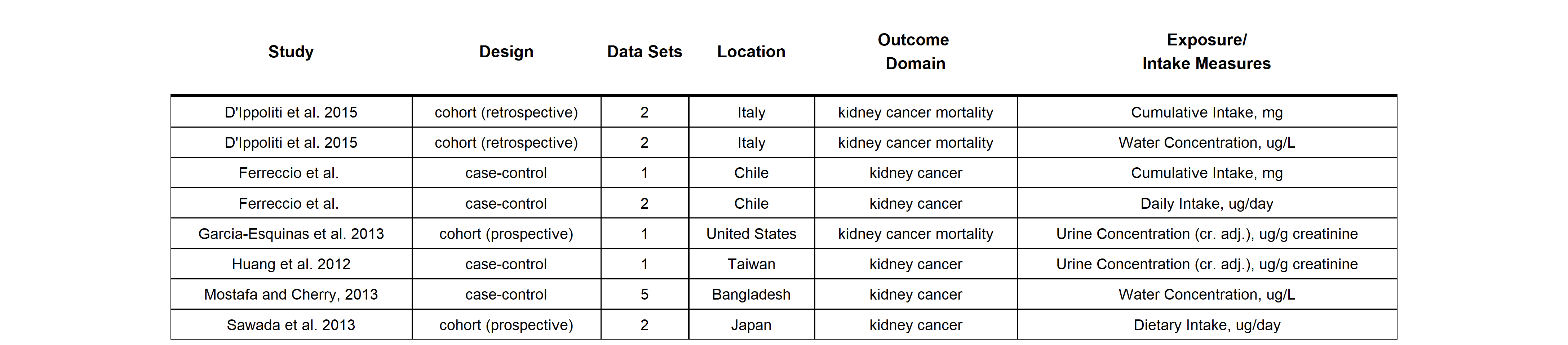


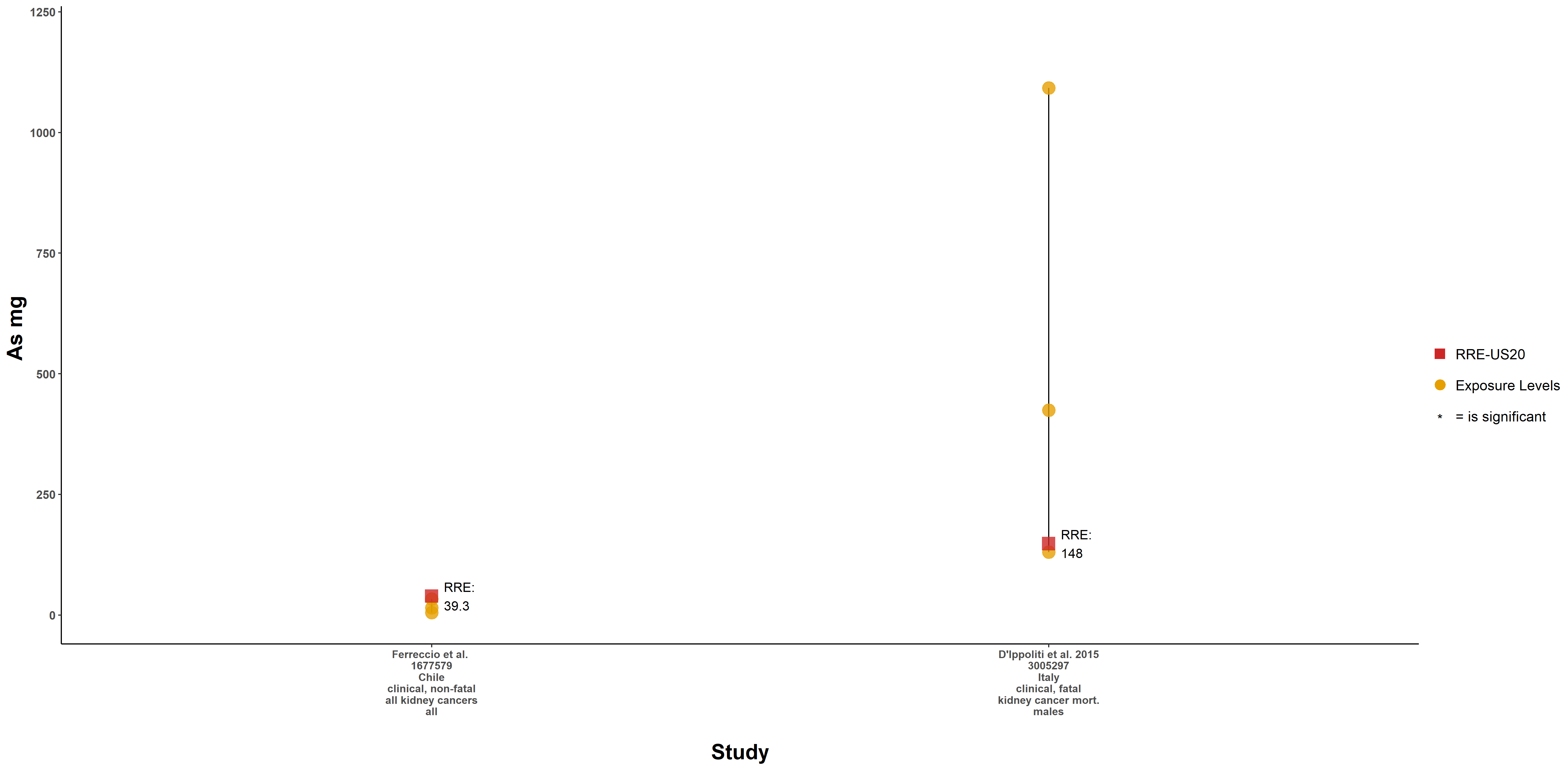


Figure S-35A. Exposure levels and RRE-US_20_ for renal cancer using cumulative intake.


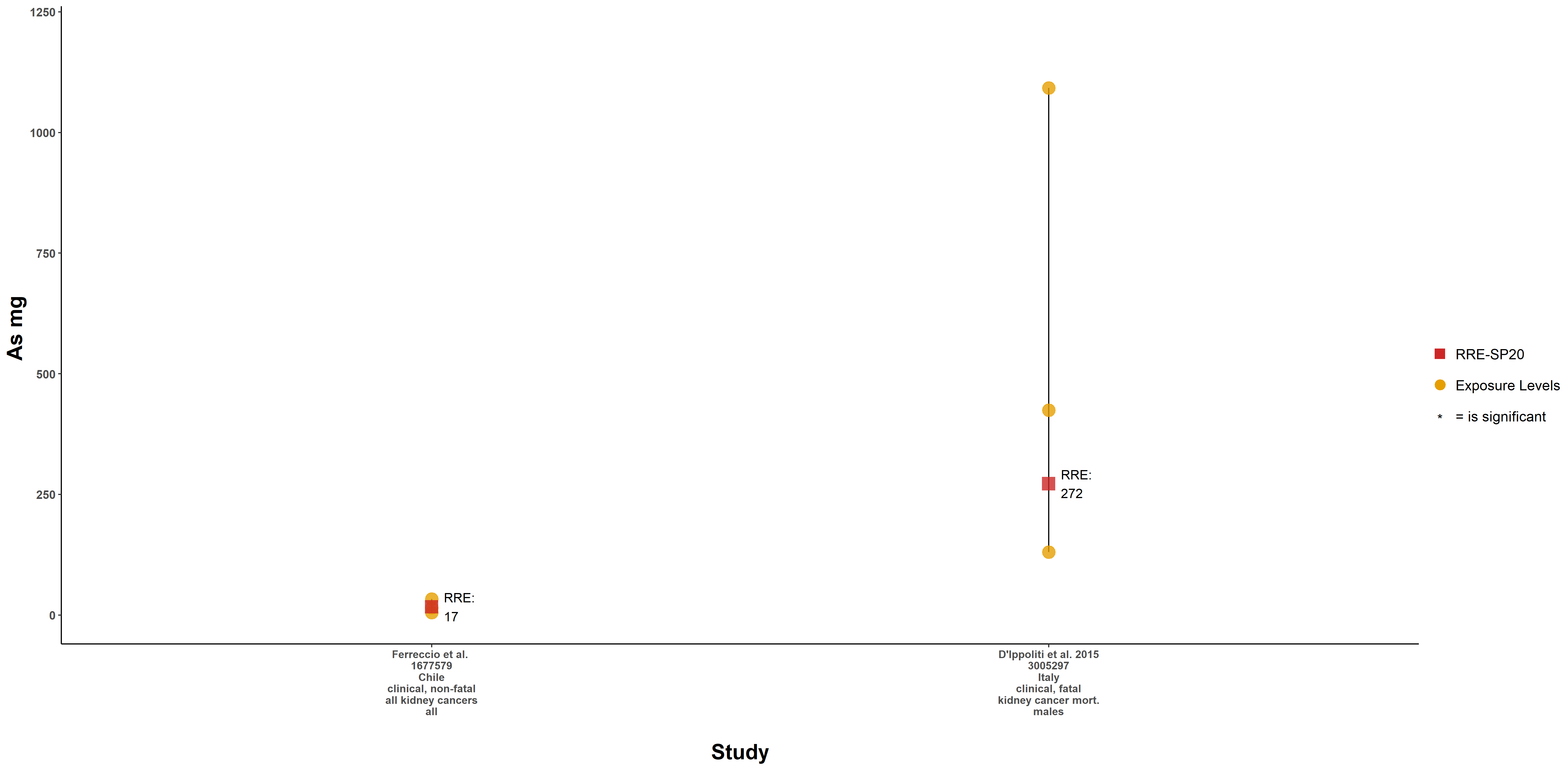


Figure S-35B. Exposure levels and RRE-SP_20_ for renal cancer using cumulative intake.


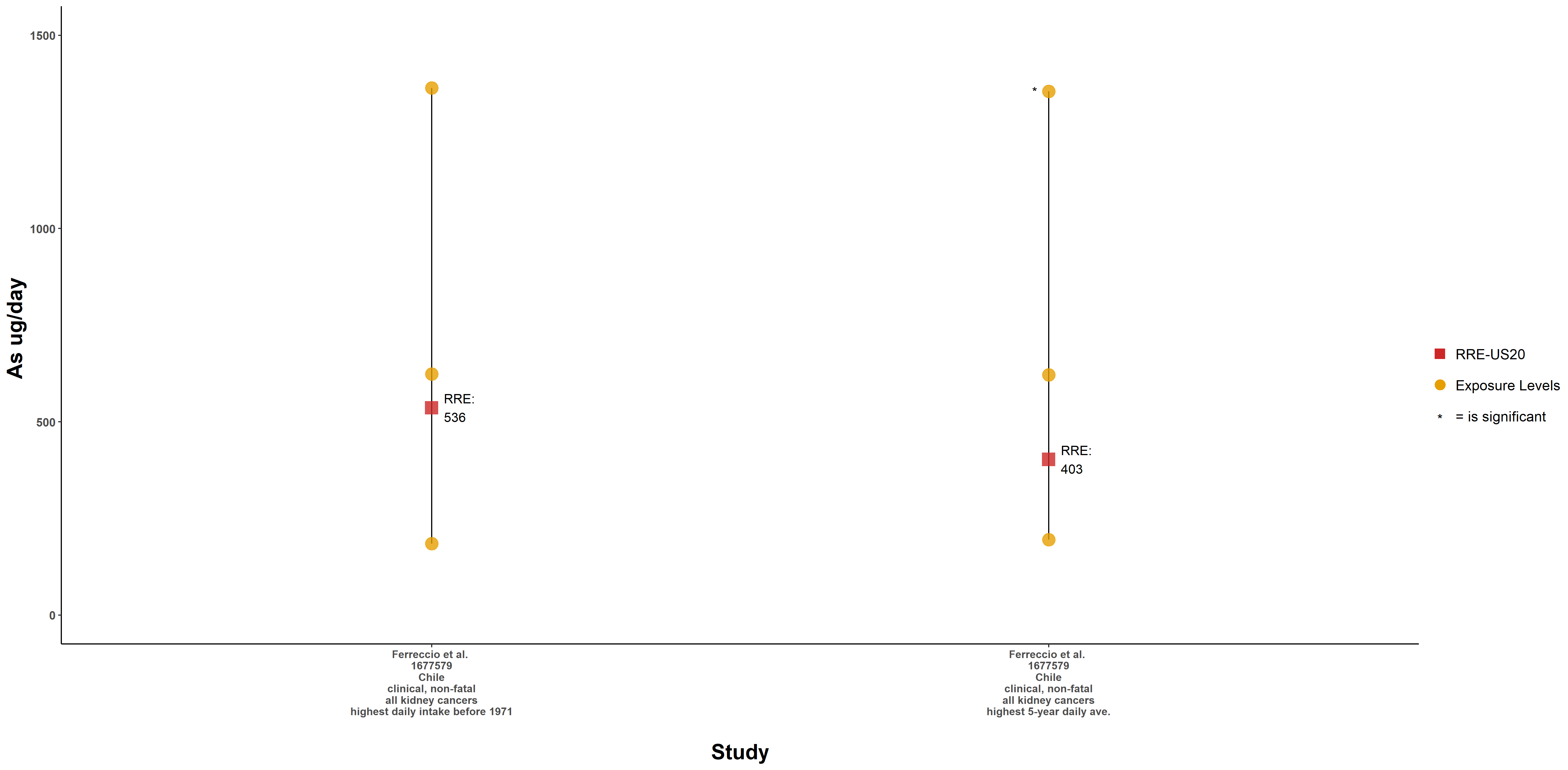


Figure S-36A. Exposure levels and RRE-US_20_ for renal cancer using daily intake.


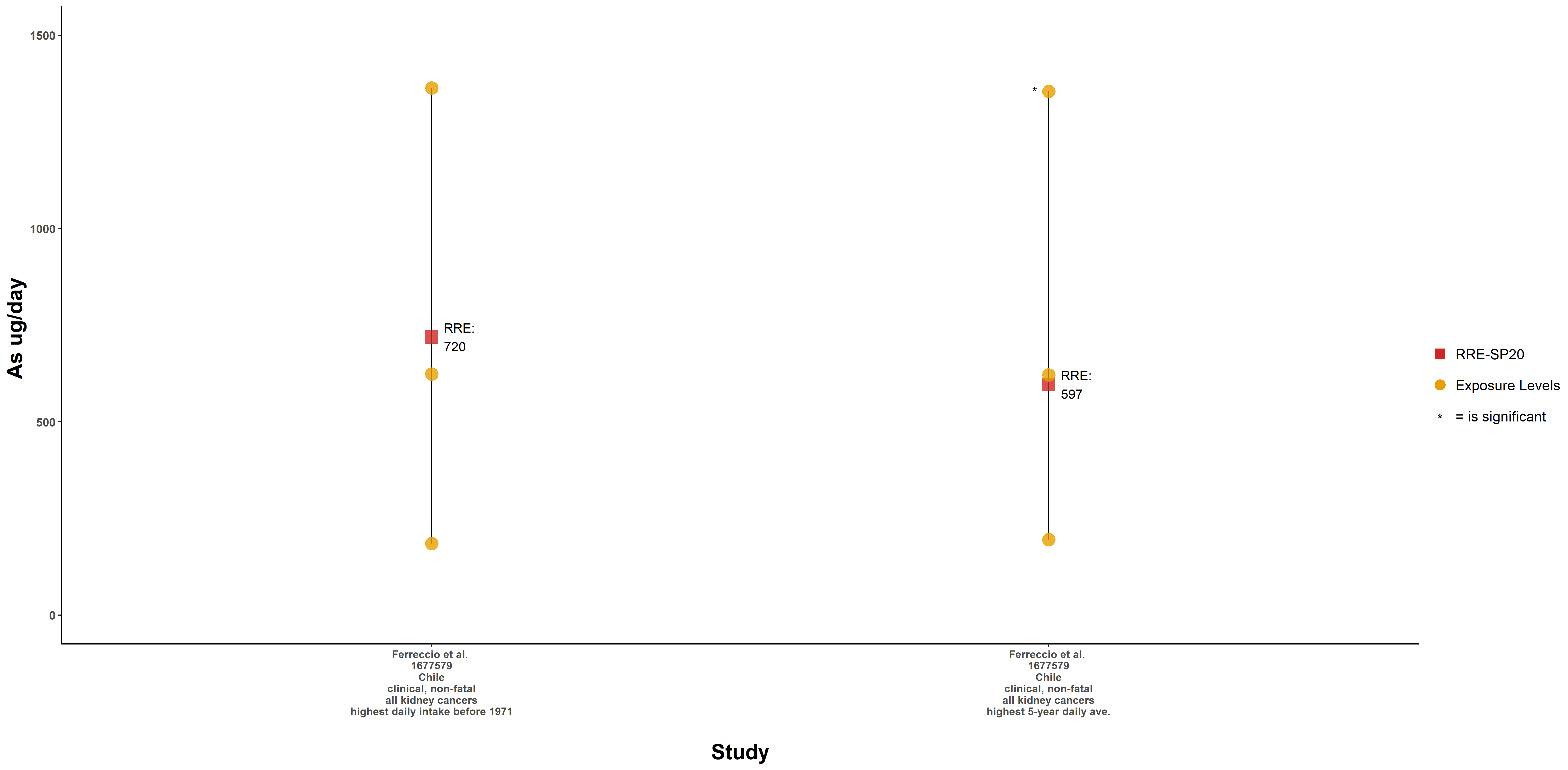


Figure S-36B. Exposure levels and RRE-SP_20_ for renal cancer using daily intake.


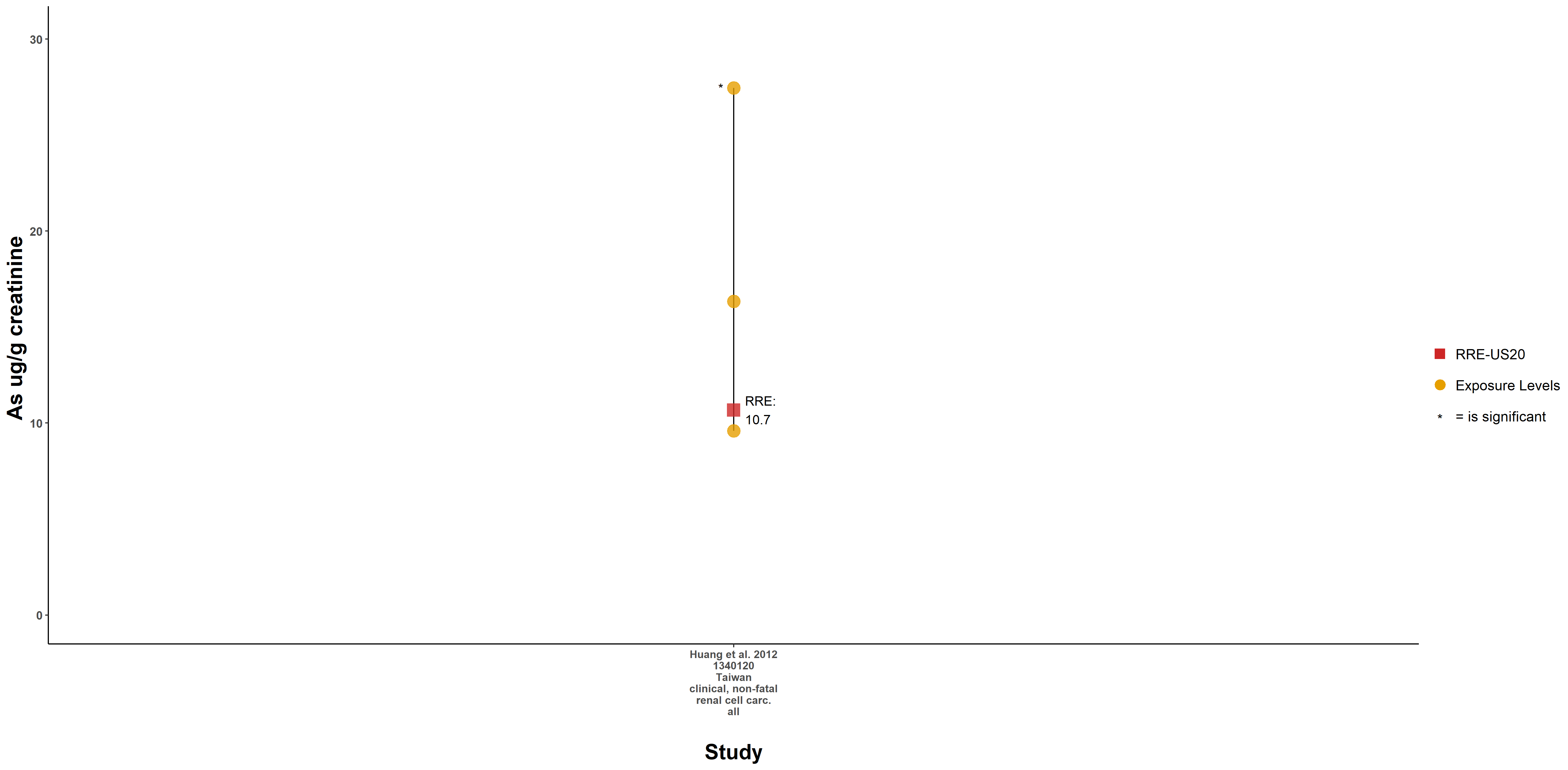


Figure S-37A. Exposure levels and RRE-US_20_ for renal cancer using creatinine adjusted urine concentration.


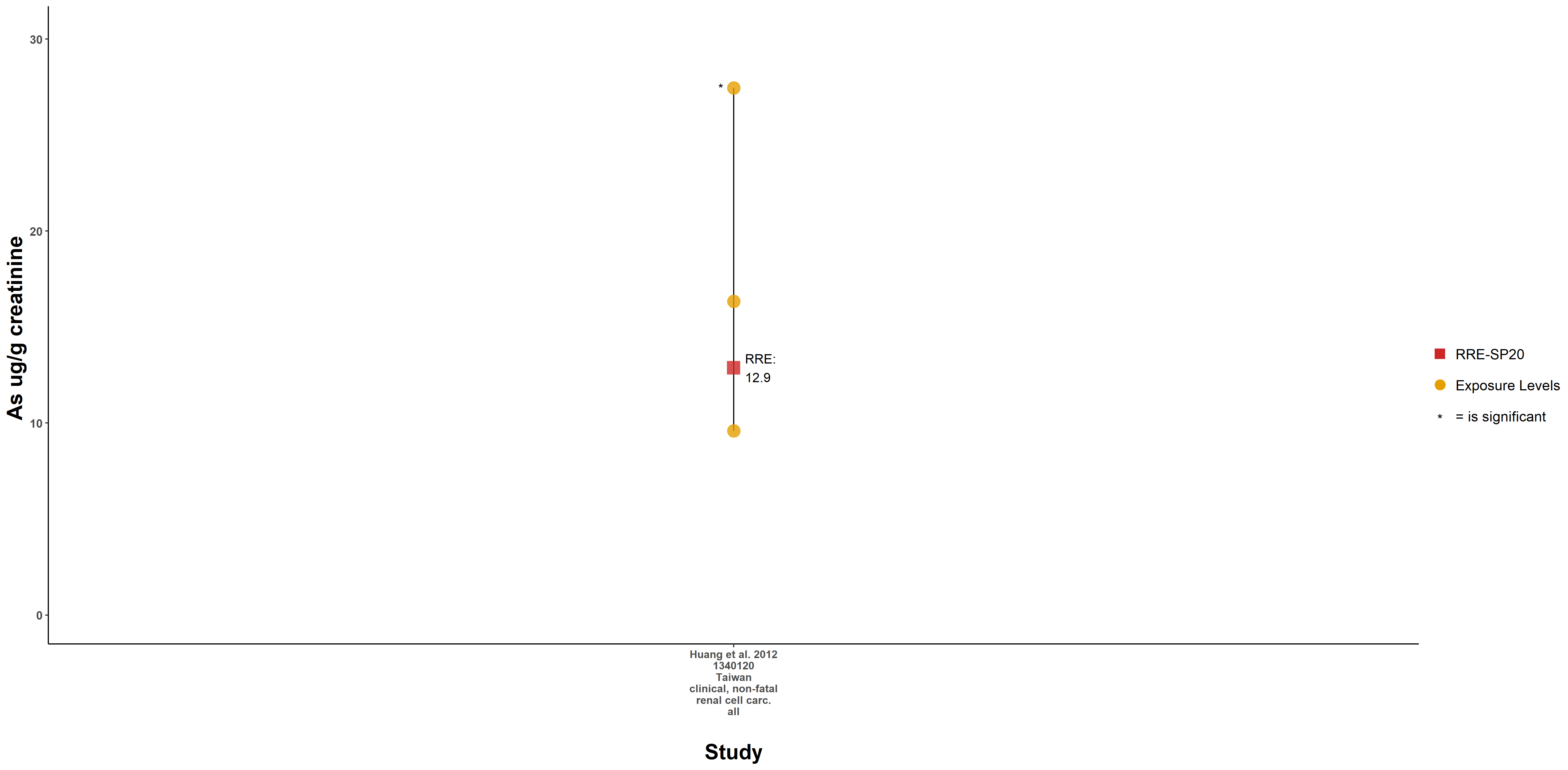


Figure S-37B. Exposure levels and RRE-SP_20_ for renal cancer using creatinine adjusted urine concentration.


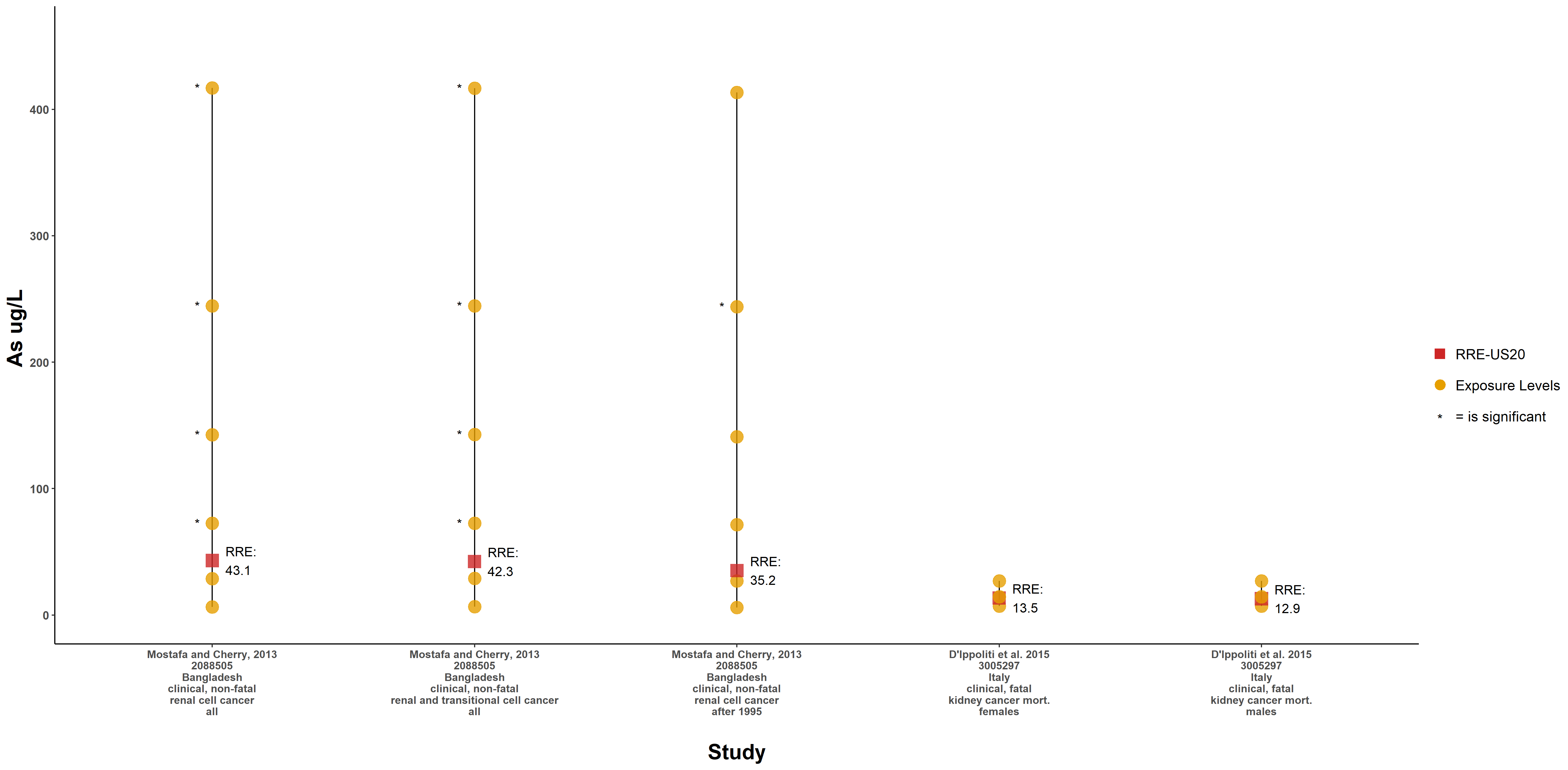


Figure S-38A. Exposure levels and RRE-US_20_ for renal cancer using water concentration.


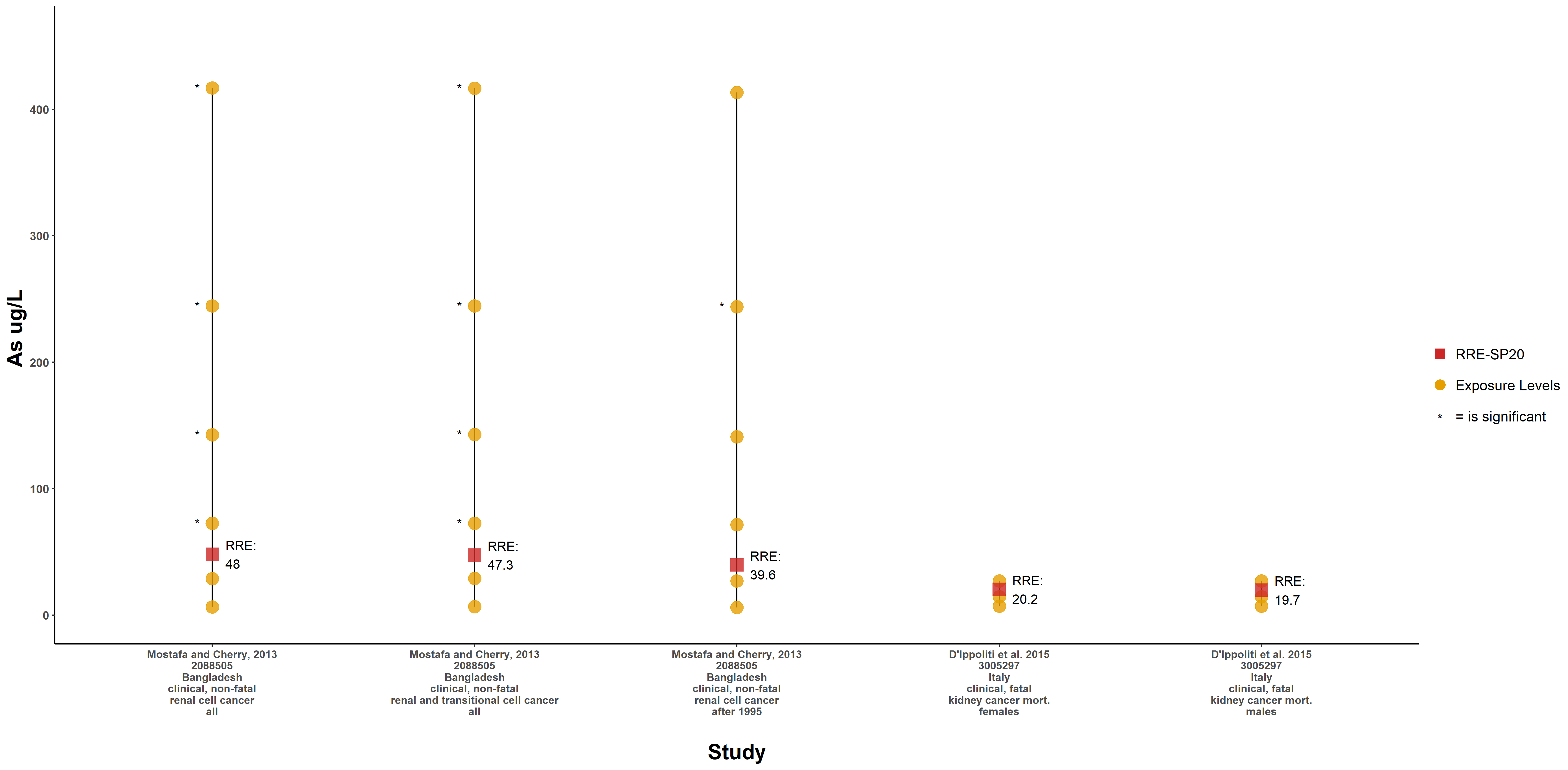


Figure S-38B. Exposure levels and RRE-SP_20_ for renal cancer using water concentration.

Table S-40A. Summary of RRE-US_20_s and RRB-US for renal cancer studies


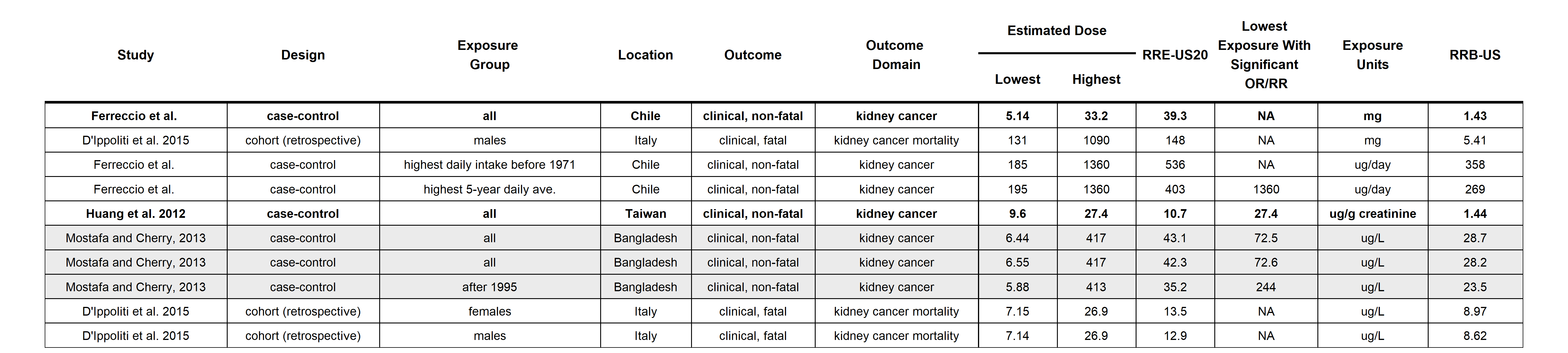


RRB-US refers to the ratio of RRE_20_ to an estimated U.S. background exposure level. Shaded cells indicate that authors did not report exposure-response trends. Bold rows indicate that authors reported a significant exposure-response trend (*p* < 0.05)

Table S-40B. Summary of RRE-SP_20_s and RRB-SP for renal cancer studies


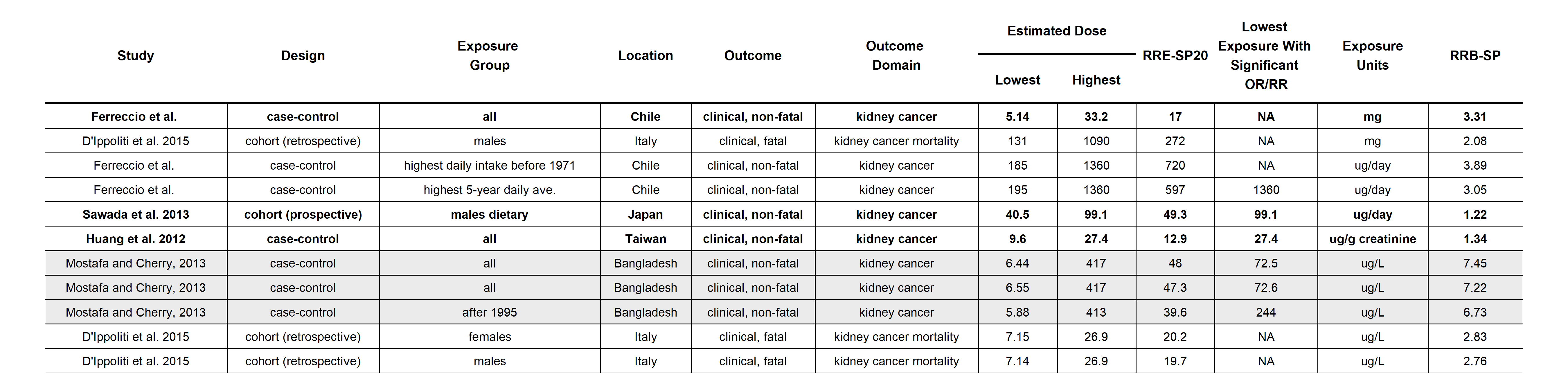


RRB-SP refers to the ratio of RRE-SP_20_ to the reported or estimated background exposure level for the study referent group. Shaded cells indicate that authors did not report exposure-response trends. Bold rows indicate that authors reported a significant exposure-response trend (*p* < 0.05)

#### Skin Cancer Exposure-Response Modeling Results

The analysis of arsenic exposure response on skin cancer outcomes evaluated 7 datasets from 3 peer reviewed studies that included endpoints such as squamous cell carcinoma and basal cell carcinoma. A summary of datasets modeled identifying the study design, location, exposure metric and outcome domain are provided in Table S-41. A breakdown of the exposure levels and RRE_20_ estimates is provided for each exposure metric in Figure S-39 and Figure S-40. Finally, RRE_20_ summary tables for all exposures are provided in Table S-42.

Table S-41. Summary of datasets considered in skin cancer exposure-response RRB analysis by exposure metric


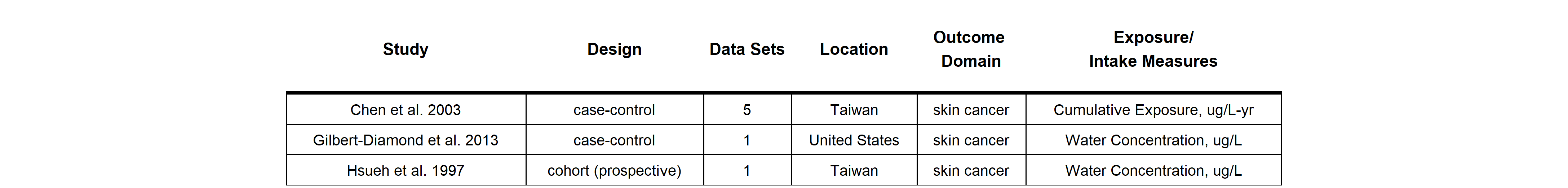


Figure S-39A. Exposure levels and RRE-US_20_ for skin cancer using cumulative exposure.

Figure S-39B. Exposure levels and RRE-SP_20_ for skin cancer using cumulative exposure.

Figure S-40A. Exposure levels and RRE-US_20_ for skin cancer using water concentration.

Figure S-40B. Exposure levels and RRE-SP_20_ for skin cancer using water concentration.

Table S-42A. Summary of RRE-US_20_s and RRB-US for skin cancer studies

RRB-US refers to the ratio of RRE-US_20_ to an estimated U.S. background exposure level. Shaded cells indicate that authors did not report exposure-response trends. Bold rows indicate that authors reported a significant exposure-response trend (*p* < 0.05)

Table S-42B. Summary of RRE-SP_20_s and RRB-SP for skin cancer studies

RRB-SP refers to the ratio of RRE-SP_20_ to the reported or estimated background exposure level for the study referent group. Bold rows indicate that authors reported a significant exposure-response trend (*p* < 0.05)

#### Skin Lesions Exposure-Response Modeling Results

The analysis of arsenic exposure response on skin lesions evaluated 25 datasets from 10 peer reviewed studies. A summary of datasets modeled identifying the study design, location, exposure metric and outcome domain is provided in Table S-43. A breakdown of the exposure levels and RRE_20_ estimates are provided for each exposure metric in Figure S-41–Figure S-45. Finally, RRE_20_ summary tables for all exposures are provided in Table S-44.

Table S-43. Summary of datasets considered in skin lesions exposure-response RRB analysis by exposure metric

Figure S-41A. Exposure levels and RRE-US_20_ for skin lesions using cumulative exposure.

Figure S-41B. Exposure levels and RRE-SP_20_ for skin lesions using cumulative exposure.

Figure S-42A. Exposure levels and RRE-US_20_ for skin lesions using daily intake.

Figure S-42B. Exposure levels and RRE-SP_20_ for skin lesions using daily intake.

Figure S-43A. Exposure levels and RRE-US_20_ for skin lesions using creatinine adjusted urine concentration.

Figure S-43B. Exposure levels and RRE-SP_20_ for skin lesions using creatinine adjusted urine concentration.

Figure S-44A. Exposure levels and RRE-US_20_ for skin lesions using urine concentration.

Figure S-44B. Exposure levels and RRE-SP_20_ for skin lesions using urine concentration.

Figure S-45A. Exposure levels and RRE-US_20_ for skin lesions using water concentration.

Figure S-45B. Exposure levels and RRE-SP_20_ for skin lesions using water concentration.

Table S-44A. Summary of RRE-US_20_s and RRB-US for skin lesions studies

RRB-US refers to the ratio of RRE-US_20_ to an estimated U.S. background exposure. Shaded cells indicate that authors did not report exposure-response trends. Bold rows indicate that authors reported a significant exposure-response trend (p <0.05)

Table S-44B. Summary of RRE-SP_20_s and RRB-SP for skin lesions studies

RRB-SP refers to the ratio of RRE-SP_20_ to the reported or estimated background exposure level for the study referent group. Shaded cells indicate that authors did not report exposure-response trends. Bold rows indicate that authors reported a significant exposure-response trend (p <0.05)

## Examples of Exposure-Response Model Uncertainty

#### Non-Positive Exposure-Response Models

Figure S-46 Example of non-positive exposure-response from Bates et al. 1995

#### Uncertainty in Michaelis-Menton and Exponential 4 Exposure-Response Models

In the example exposure response plot, Figure S-47, only the Michaelis-Menton and Exponential 4 models provided acceptable fit and positive and finite estimates for the RRE-US20. However the 20% increase in response is detected during the superlinear response and corresponded with RRE20s that are greater than a factor of 10 below estimates from other models. In these cases, the linear model was selected if it met the acceptable fit criteria, otherwise these datasets were excluded.

Figure S-47 Example of model uncertainty with Michaelis Menton and Exponential 4 models from dataset in Rahman 2010.

**REFERENCES**

't Mannetje, A; Bencko, V; Brennan, P; Zaridze, D; Szeszenia-Dabrowska, N; Rudnai, P; Lissowska, J; Fabiánová, E; Cassidy, A; Mates, D; Foretova, L; Janout, V; Fevotte, J; Fletcher, T; Boffetta, P. (2011). Occupational exposure to metal compounds and lung cancer. Results from a multi-center case-control study in Central/Eastern Europe and UK. Cancer Causes Control 22: 1669-1680. <http://dx.doi.org/10.1007/s10552-011-9843-3>

Ades, AE; Kazantzis, G. (1988). Lung cancer in a non-ferrous smelter: The role of cadmium. Br J Ind Med 45: 435-442. <http://dx.doi.org/10.1136/oem.45.7.435>

Aelion, CM; Davis, HT; Lawson, AB; Cai, B; McDermott, S. (2012). Associations of estimated residential soil arsenic and lead concentrations and community-level environmental measures with mother-child health conditions in South Carolina. Health Place 18: 774-781. <http://dx.doi.org/10.1016/j.healthplace.2012.04.005>

Ahamed, S; Kumar Sengupta, M; Mukherjee, A; Amir Hossain, M; Das, B; Nayak, B; Pal, A; Chandra Mukherjee, S; Pati, S; Nath Dutta, R; Chatterjee, G; Mukherjee, A; Srivastava, R; Chakraborti, D. (2006a). Arsenic groundwater contamination and its health effects in the state of Uttar Pradesh (UP) in upper and middle Ganga plain, India: A severe danger. Sci Total Environ 370: 310-322. <http://dx.doi.org/10.1016/j.scitotenv.2006.06.015>

Ahamed, S; Sengupta, MK; Mukherjee, SC; Pati, S; Mukherjeel, A; Rahman, MM; Hossain, MA; Das, B; Nayakl, B; Pal, A; Zafar, A; Kabir, S; Banu, SA; Morshed, S; Islam, T; Rahman, MM; Quamruzzaman, Q; Chakraborti, D. (2006b). An eight-year study report on arsenic contamination in groundwater and health effects in Eruani village, Bangladesh and an approach for its mitigation. J Health Popul Nutr 24: 129-141.

Ahmad, SA; Khatun, F; Sayed, MHS, U; Khan, MH; Aziz, MR; Hossain, MZ; Faruquee, MH. (2006). Electrocardiographic abnormalities among arsenic-exposed persons through groundwater in Bangladesh. J Health Popul Nutr 24: 221-227.

Ahmad, SA; Sayed, MHS, U; Barua, S; Khan, MH; Faruquee, MH; Jalil, A; Hadi, SA; Talukder, HK. (2001). Arsenic in drinking water and pregnancy outcomes. Environ Health Perspect 109: 629-631. <http://dx.doi.org/10.1289/ehp.01109629>

Ahmad, SA; Sayed, MHS, U; Hadi, SA; Faruquee, MH; Khan, MH; Jalil, MA; Ahmed, R; Khan, AW. (1999). Arsenicosis in a village in Bangladesh. Int J Environ Health Res 9: 187-195. <http://dx.doi.org/10.1080/09603129973155>

Ahmed, S; Ahsan, KB; Kippler, M; Mily, A; Wagatsuma, Y; Hoque, AMW; Ngom, PT; El Arifeen, S; Raqib, R; Vahter, M. (2012). In utero arsenic exposure is associated with impaired thymic function in newborns possibly via oxidative stress and apoptosis. Toxicol Sci 129: 305-314. <http://dx.doi.org/10.1093/toxsci/kfs202>

Ahmed, S; Moore, SE; Kippler, M; Gardner, R; Hawlader, MDH; Wagatsuma, Y; Raqib, R; Vahter, M. (2014). Arsenic exposure and cell-mediated immunity in pre-school children in rural Bangladesh. Toxicol Sci 141: 166-175. <http://dx.doi.org/10.1093/toxsci/kfu113>

Ahsan, H; Chen, Y; Parvez, F; Zablotska, L; Argos, M; Hussain, I; Momotaj, H; Levy, D; Cheng, Z; Slavkovich, V; van Geen, A; Howe, GR; Graziano, JH. (2006). Arsenic exposure from drinking water and risk of premalignant skin lesions in Bangladesh: Baseline results from the Health Effects of Arsenic Longitudinal Study. Am J Epidemiol 163: 1138-1148. <http://dx.doi.org/10.1093/aje/kwj154>

Ahsan, H; Perrin, M; Rahman, A; Parvez, F; Stute, M; Zheng, Y; Milton, AH; Brandt-Rauf, P; van Geen, A; Graziano, J. (2000). Associations between drinking water and urinary arsenic levels and skin lesions in Bangladesh. J Occup Environ Med 42: 1195-1201. <http://dx.doi.org/10.1097/00043764-200012000-00016>

Ameer, SS; Engström, K; Harari, F; Concha, G; Vahter, M; Broberg, K. (2015). The effects of arsenic exposure on blood pressure and early risk markers of cardiovascular disease: Evidence for population differences. Environ Res 140: 32-36. <http://dx.doi.org/10.1016/j.envres.2015.03.010>

Applebaum, KM; Karagas, MR; Hunter, DJ; Catalano, PJ; Byler, SH; Morris, S; Nelson, HH. (2007). Polymorphisms in nucleotide excision repair genes, arsenic exposure, and non-melanoma skin cancer in New Hampshire. Environ Health Perspect 115: 1231-1236. <http://dx.doi.org/10.1289/ehp.10096>

Argos, M; Kalra, T; Pierce, BL; Chen, Y; Parvez, F; Islam, T; Ahmed, A; Hasan, R; Hasan, K; Sarwar, G; Levy, D; Slavkovich, V; Graziano, JH; Rathouz, PJ; Ahsan, H. (2011). A prospective study of arsenic exposure from drinking water and incidence of skin lesions in Bangladesh. Am J Epidemiol 174: 185-194. <http://dx.doi.org/10.1093/aje/kwr062>

Argos, M; Parvez, F; Chen, Y; Hussain, AZM, I; Momotaj, H; Howe, GR; Graziano, JH; Ahsan, H. (2007). Socioeconomic status and risk for arsenic-related skin lesions in Bangladesh. Am J Public Health 97: 825-831. <http://dx.doi.org/10.2105/AJPH.2005.078816>

Argos, M; Parvez, F; Rahman, M; Rakibuz-Zaman, M; Ahmed, A; Hore, SK; Islam, T; Chen, Y; Pierce, BL; Slavkovich, V; Olopade, C; Yunus, M; Baron, JA; Graziano, JH; Ahsan, H. (2014). Arsenic and lung disease mortality in Bangladeshi adults. Epidemiology 25: 536-543. <http://dx.doi.org/10.1097/EDE.0000000000000106>

Aschengrau, A; Zierler, S; Cohen, A. (1989). Quality of community drinking water and the occurrence of spontaneous abortion. Arch Environ Health 44: 283-290. <http://dx.doi.org/10.1080/00039896.1989.9935895>

Axelson, O; Dahlgren, E; Jansson, CD; Rehnlund, SO. (1978). Arsenic exposure and mortality: A case-referent study from a Swedish copper smelter. Br J Ind Med 35: 8-15. <http://dx.doi.org/10.1136/oem.35.1.8>

Baastrup, R; Sørensen, M; Balstrøm, T; Frederiksen, K; Larsen, CL; Tjønneland, A; Overvad, K; Raaschou-Nielsen, O. (2008). Arsenic in drinking-water and risk for cancer in Denmark. Environ Health Perspect 116: 231-237. <http://dx.doi.org/10.1289/ehp.10623>

Barati, AH; Maleki, A; Alasvand, M. (2010). Multi-trace elements level in drinking water and the prevalence of multi-chronic arsenical poisoning in residents in the west area of Iran. Sci Total Environ 408: 1523-1529. <http://dx.doi.org/10.1016/j.scitotenv.2009.12.035>

Baris, D; Waddell, R; Beane Freeman, LE; Schwenn, M; Colt, JS; Ayotte, JD; Ward, MH; Nuckols, J; Schned, A; Jackson, B; Clerkin, C; Rothman, N; Moore, LE; Taylor, A; Robinson, G; Hosain, GM; Armenti, KR; Mccoy, R; Samanic, C; Hoover, RN; Fraumeni, JF; Johnson, A; Karagas, MR; Silverman, DT. (2016). Elevated Bladder Cancer in Northern New England: The Role of Drinking Water and Arsenic. J Natl Cancer Inst 108. <http://dx.doi.org/10.1093/jnci/djw099>

Bates, MN; Rey, OA; Biggs, ML; Hopenhayn, C; Moore, LE; Kalman, D; Steinmaus, C; Smith, AH. (2004). Case-control study of bladder cancer and exposure to arsenic in Argentina. Am J Epidemiol 159: 381-389. <http://dx.doi.org/10.1093/aje/kwh054>

Bates, MN; Smith, AH; Cantor, KP. (1995). Case-control study of bladder cancer and arsenic in drinking water. 141: 523-530.

Beane Freeman, LE; Dennis, LK; Lynch, CF; Thorne, PS; Just, CL. (2004). Toenail arsenic content and cutaneous melanoma in Iowa. Am J Epidemiol 160: 679-687. <http://dx.doi.org/10.1093/aje/kwh267>

Bencko, V; Rames, J; Fabiánová, E; Pesek, J; Jakubis, M. (2009). Ecological and human health risk aspects of burning arsenic-rich coal. Environ Geochem Health 31: 239-243. <http://dx.doi.org/10.1007/s10653-008-9224-3>

Bencko, V; Symon, K; Stálnik, L; Bátora, J; Vanco, E; Svandová, E. (1980). Rate of malignant tumor mortality among coal burning power plant workers occupationally exposed to arsenic. J Hyg Epidemiol Microbiol Immunol 24: 278-284.

Besuschio, SC; Perez Desanzo, AC; Croci, M. (1980). Epidemiological associations between arsenic and cancer in Argentina. Biol Trace Elem Res 2: 41-55. <http://dx.doi.org/10.1007/BF02789034>

Bhattacharyya, P; Sen, P; Ghosh, A; Saha, C; Bhattacharya, PP; Das, A; Majumdar, K; Mazumder, DG. (2014). Chronic lung disease and detection of pulmonary artery dilatation in high resolution computerized tomography of chest in chronic arsenic exposure. J Environ Sci Health A Tox Hazard Subst Environ Eng 49: 1453-1461. <http://dx.doi.org/10.1080/10934529.2014.937157>

Bhowmick, S; Halder, D; Kundu, AK; Saha, D; Iglesias, M; Nriagu, J; Guha Mazumder, DN; Roman-Ross, G; Chatterjee, D. (2013). Is saliva a potential biomarker of arsenic exposure? A case-control study in West Bengal, India. Environ Sci Technol 47: 3326-3332. <http://dx.doi.org/10.1021/es303756s>

Biswas, BK; Dhar, RK; Samanta, G; Mandal, BK; Chakraborti, D; Faruk, I; Islam, KS; Chowdhury, MM; Islam, A; Roy, S. (1998). Detailed study report of Samta, one of the arsenic-affected villages of Jessore District, Bangladesh. Curr Sci 74: 134-145.

Biswas, R; Ghosh, P; Banerjee, N; Das, JK; Sau, T; Banerjee, A; Roy, S; Ganguly, S; Chatterjee, M; Mukherjee, A; Giri, AK. (2008). Analysis of T-cell proliferation and cytokine secretion in the individuals exposed to arsenic. Hum Exp Toxicol 27: 381-386. <http://dx.doi.org/10.1177/0960327108094607>

Bloom, MS; Buck Louis, GM; Sundaram, R; Maisog, JM; Steuerwald, AJ; Parsons, PJ. (2015). Birth outcomes and background exposures to select elements, the Longitudinal Investigation of Fertility and the Environment (LIFE). Environ Res 138: 118-129. <http://dx.doi.org/10.1016/j.envres.2015.01.008>

Bloom, MS; Neamtiu, IA; Surdu, S; Pop, C; Anastasiu, D; Appleton, AA; Fitzgerald, EF; Gurzau, ES. (2016). Low level arsenic contaminated water consumption and birth outcomes in Romania-An exploratory study. Reproductive Toxicology 59: 8-16. http://dx.doi.org/10.1016/j.reprotox.2015.10.012

Bloom, MS; Neamtiu, IA; Surdu, S; Pop, C; Lupsa, IR; Anastasiu, D; Fitzgerald, EF; Gurzau, ES. (2014). Consumption of low-moderate level arsenic contaminated water does not increase spontaneous pregnancy loss: a case control study. Environ Health 13: 81. <http://dx.doi.org/10.1186/1476-069X-13-81>

Boffetta, P; Fontana, L; Stewart, P; Zaridze, D; Szeszenia-Dabrowska, N; Janout, V; Bencko, V; Foretova, L; Jinga, V; Matveev, V; Kollarova, H; Ferro, G; Chow, W, -H; Rothman, N; van Bemmel, D; Karami, S; Brennan, P; Moore, LE. (2011). Occupational exposure to arsenic, cadmium, chromium, lead and nickel, and renal cell carcinoma: A case-control study from Central and Eastern Europe. Occup Environ Med 68: 723-728. <http://dx.doi.org/10.1136/oem.2010.056341>

Borgoño, JM; Vicent, P; Venturino, H; Infante, A. (1977). Arsenic in the drinking water of the city of Antofagasta: Epidemiological and clinical study before and after the installation of a treatment plant. Environ Health Perspect 19: 103-105. <http://dx.doi.org/10.2307/3428458>

Bošnjak, Z; Cavar, S; Klapec, T; Milić, M; Klapec-Basar, M; Toman, M. (2008). Selected markers of cardiovascular disease in a population exposed to arsenic from drinking water. Environ Toxicol Pharmacol 26: 181-186. <http://dx.doi.org/10.1016/j.etap.2008.03.005>

Bräuner, EV; Nordsborg, RB; Andersen, ZJ; Tjønneland, A; Loft, S; Raaschou-Nielsen, O. (2014). Long-term exposure to low-level arsenic in drinking water and diabetes incidence: A prospective study of the diet, cancer and health cohort. Environ Health Perspect 122: 1059-1065. <http://dx.doi.org/10.1289/ehp.1408198>

Breton, CV; Houseman, EA; Kile, ML; Quamruzzaman, Q; Rahman, M; Mahiuddin, G; Christiani, DC. (2006). Gender-specific protective effect of hemoglobin on arsenic-induced skin lesions. Cancer Epidemiol Biomarkers Prev 15: 902-907. <http://dx.doi.org/10.1158/1055-9965.EPI-05-0859>

Buchet, JP; Lison, D. (1998). Mortality by cancer in groups of the Belgian population with a moderately increased intake of arsenic. Int Arch Occup Environ Health 71: 125-130. <http://dx.doi.org/10.1007/s004200050259>

Bulbulyan, MA; Jourenkova, NJ; Boffetta, P; Astashevsky, SV; Mukeria, AF; Zaridze, DG. (1996). Mortality in a cohort of Russian fertilizer workers. Scand J Work Environ Health 22: 27-33. <http://dx.doi.org/10.5271/sjweh.105>

Burgess, JL; Kurzius-Spencer, M; O'Rourke, MK; Littau, SR; Roberge, J; Meza-Montenegro, MM; Gutiérrez-Millán, LE; Harris, RB. (2013). Environmental arsenic exposure and serum matrix metalloproteinase-9. J Expo Sci Environ Epidemiol 23: 163-169. <http://dx.doi.org/10.1038/jes.2012.107>

Cebrián, ME; Albores, A; Aguilar, M; Blakely, E. (1983). Chronic arsenic poisoning in the north of Mexico. Hum Exp Toxicol 2: 121-133. <http://dx.doi.org/10.1177/096032718300200110>

Chakraborti, D; Mukherjee, SC; Pati, S; Sengupta, MK; Rahman, MM; Chowdhury, UK; Lodh, D; Chanda, CR; Chakraborti, AK; Basu, GK. (2003). Arsenic groundwater contamination in Middle Ganga Plain, Bihar, India: A future danger? Environ Health Perspect 111: 1194-1201. <http://dx.doi.org/10.1289/ehp.5966>

Chakraborti, D; Rahman, MM; Murrill, M; Das, R; Siddayya; Patil, SG; Sarkar, A; Dadapeer, HJ; Yendigeri, S; Ahmed, R; Das, KK. (2013). Environmental arsenic contamination and its health effects in a historic gold mining area of the Mangalur greenstone belt of Northeastern Karnataka, India. J Hazard Mater 262: 10481055. <http://dx.doi.org/10.1016/j.jhazmat.2012.10.002>

Chang, C, -C; Ho, S, -C; Tsai, S, -S; Yang, C, -Y. (2004). Ischemic heart disease mortality reduction in an arseniasis-endemic area in southwestern Taiwan after a switch in the tap-water supply system. J Toxicol Environ Health A 67: 1353-1361. <http://dx.doi.org/10.1080/15287390490471451>

Chattopadhyay, BP; Mukherjee, AK; Gangopadhyay, PK; Alam, J; Roychowdhury, A. (2010). Respiratory effect related to exposure of different concentrations of arsenic in drinking water in West Bengal, India. Indian J Environ Health 52: 147-154.

Chen, C, -J; Chen, CW; Wu, M, -M; Kuo, T, -L. (1992). Cancer potential in liver, lung, bladder and kidney due to ingested inorganic arsenic in drinking water. Br J Cancer 66: 888-892. <http://dx.doi.org/10.1038/bjc.1992.380>

Chen, C, -J; Chiou, H, -Y; Chiang, M, -H; Lin, L, -J; Tai, T, -Y. (1996). Dose-response relationship between ischemic heart disease mortality and long-term arsenic exposure. Arterioscler Thromb Vasc Biol 16: 504-510. <http://dx.doi.org/10.1161/01.ATV.16.4.504>

Chen, C, -J; Chuang, Y, -C; You, S, -L; Lin, T, -M; Wu, H, -Y. (1986). A retrospective study on malignant neoplasms of bladder, lung and liver in blackfoot disease endemic area in Taiwan. Br J Cancer 53: 399-405. <http://dx.doi.org/10.1038/bjc.1986.65>

Chen, C, -J; Hsueh, Y, -M; Lai, M, -S; Shyu, M, -P; Chen, S, -Y; Wu, M, -M; Kuo, T, -L; Tai, T, -Y. (1995). Increased prevalence of hypertension and long-term arsenic exposure. Hypertension 25: 53-60. <http://dx.doi.org/10.1161/01.HYP.25.1.53>

Chen, C, -J; Wu, M, -M; Lee, S, -S; Wang, J, -D; Cheng, S, -H; Wu, H, -Y. (1988). Atherogenicity and carcinogenicity of high-arsenic artesian well water. Multiple risk factors and related malignant neoplasms of blackfoot disease. 8: 452-460. <http://dx.doi.org/10.1161/01.ATV.8.5.452>

Chen, C, -L; Hsu, L, -I; Chiou, H, -Y; Hsueh, Y, -M; Chen, S, -Y; Wu, M, -M; Chen, C, -J; Group, ftBDS. (2004). Ingested arsenic, cigarette smoking, and lung cancer risk: A follow-up study in arseniasis-endemic areas in Taiwan. JAMA 292: 2984-2990. <http://dx.doi.org/10.1001/jama.292.24.2984>

Chen, CJ; Chuang, YC; Lin, TM; Wu, HY. (1985). Malignant neoplasms among residents of a blackfoot disease-endemic area in Taiwan: High-arsenic artesian well water and cancers. Cancer Res 45: 5895-5899.

Chen, CJ; Wang, CJ. (1990). Ecological correlation between arsenic level in well water and age-adjusted mortality from malignant neoplasms. Cancer Res 50: 5470-5474.

Chen, CL; Chiou, HY; Hsu, LI; Hsueh, YM; Wu, MM; Chen, CJ. (2010a). Ingested arsenic, characteristics of well water consumption and risk of different histological types of lung cancer in northeastern Taiwan. Environ Res 110: 455-462. <http://dx.doi.org/10.1016/j.envres.2009.08.010>

Chen, CL; Chiou, HY; Hsu, LI; Hsueh, YM; Wu, MM; Wang, YH; Chen, CJ. (2010b). Arsenic in drinking water and risk of urinary tract cancer: A follow-up study from northeastern Taiwan. Cancer Epidemiol Biomarkers Prev 19: 101-110. <http://dx.doi.org/10.1158/1055-9965.EPI-09-0333>

Chen, J, -W; Chen, H, -Y; Li, W, -F; Liou, S, -H; Chen, C, -J; Wu, J, -H; Wang, S, -L. (2011a). The association between total urinary arsenic concentration and renal dysfunction in a community-based population from central Taiwan. Chemosphere 84: 17-24. <http://dx.doi.org/10.1016/j.chemosphere.2011.02.091>

Chen, J, -W; Wang, S, -L; Wang, Y, -H; Sun, C, -W; Huang, Y, -L; Chen, C, -J; Li, W, -F. (2012a). Arsenic methylation, GSTO1 polymorphisms, and metabolic syndrome in an arseniasis endemic area of southwestern Taiwan. Chemosphere 88: 432-438. <http://dx.doi.org/10.1016/j.chemosphere.2012.02.059>

Chen, K; Liao, QL; Ma, ZW; Jin, Y; Hua, M; Bi, J; Huang, L. (2014). Association of soil arsenic and nickel exposure with cancer mortality rates, a town-scale ecological study in Suzhou, China. Environ Sci Pollut Res Int 22: 5395-5404. <http://dx.doi.org/10.1007/s11356-014-3790-y>

Chen, SC; Chen, CC; Kuo, CY; Huang, CH; Lin, CH; Lu, ZY; Chen, YY; Lee, HS; Wong, RH. (2012b). Elevated risk of hypertension induced by arsenic exposure in Taiwanese rural residents: Possible effects of manganese superoxide dismutase (MnSOD) and 8-oxoguanine DNA glycosylase (OGG1) genes. Arch Toxicol 86: 869-878. <http://dx.doi.org/10.1007/s00204-011-0797-8>

Chen, W; Chen, J. (2002). Nested case-control study of lung cancer in four Chinese tin mines. Occup Environ Med 59: 113-118. <http://dx.doi.org/10.1136/oem.59.2.113>

Chen, Y, -C; Guo, Y, -LL; Su, H, -JJ; Hsueh, Y, -M; Smith, TJ; Ryan, LM; Lee, M, -S; Chao, S, -C; Lee, JY, -Y; Christiani, DC. (2003a). Arsenic methylation and skin cancer risk in southwestern Taiwan. J Occup Environ Med 45: 241-248. <http://dx.doi.org/10.1097/01.jom.0000058336.05741.e8>

Chen, Y, -C; Su, H, -JJ; Guo, Y, -LL; Hsueh, Y, -M; Smith, TJ; Ryan, LM; Lee, M, -S; Christiani, DC. (2003b). Arsenic methylation and bladder cancer risk in Taiwan. Cancer Causes Control 14: 303-310. <http://dx.doi.org/10.1023/A:1023905900171>

Chen, Y; Ahsan, H; Slavkovich, V; Peltier, GL; Gluskin, RT; Parvez, F; Liu, X; Graziano, JH. (2010c). No association between arsenic exposure from drinking water and diabetes mellitus: A cross-sectional study in Bangladesh. Environ Health Perspect 118: 1299-1305. <http://dx.doi.org/10.1289/ehp.0901559>

Chen, Y; Factor-Litvak, P; Howe, GR; Graziano, JH; Brandt-Rauf, P; Parvez, F; van Geen, A; Ahsan, H. (2007a). Arsenic exposure from drinking water, dietary intakes of B vitamins and folate, and risk of high blood pressure in Bangladesh: A population-based, cross-sectional study. Am J Epidemiol 165: 541-552. <http://dx.doi.org/10.1093/aje/kwk037>

Chen, Y; Graziano, JH; Parvez, F; Hussain, I; Momotaj, H; van Geen, A; Howe, GR; Ahsan, H. (2006a). Modification of risk of arsenic-induced skin lesions by sunlight exposure, smoking, and occupational exposures in Bangladesh. Epidemiology 17: 459-467. <http://dx.doi.org/10.1097/01.ede.0000220554.50837.7f>

Chen, Y; Graziano, JH; Parvez, F; Liu, M; Slavkovich, V; Kalra, T; Argos, M; Islam, T; Ahmed, A; Rakibuz-Zaman, M; Hasan, R; Sarwar, G; Levy, D; van Geen, A; Ahsan, H. (2011b). Arsenic exposure from drinking water and mortality from cardiovascular disease in Bangladesh: Prospective cohort study. 342: d2431. <http://dx.doi.org/10.1136/bmj.d2431>

Chen, Y; Hakim, ME; Parvez, F; Islam, T; Rahman, AM; Ahsan, H. (2006b). Arsenic exposure from drinking-water and carotid artery intima-medial thickness in healthy young adults in Bangladesh. J Health Popul Nutr 24: 253-257.

Chen, Y; Hall, M; Graziano, JH; Slavkovich, V; van Geen, A; Parvez, F; Ahsan, H. (2007b). A prospective study of blood selenium levels and the risk of arsenic-related premalignant skin lesions. Cancer Epidemiol Biomarkers Prev 16: 207-213. <http://dx.doi.org/10.1158/1055-9965.EPI-06-0581>

Chen, Y; Wu, F; Graziano, JH; Parvez, F; Liu, M; Paul, RR; Shaheen, I; Sarwar, G; Ahmed, A; Islam, T; Slavkovich, V; Rundek, T; Demmer, RT; Desvarieux, M; Ahsan, H. (2013a). Arsenic exposure from drinking water, arsenic methylation capacity, and carotid intima-media thickness in Bangladesh. Am J Epidemiol 178: 372-381. <http://dx.doi.org/10.1093/aje/kwt001>

Chen, Y; Wu, F; Liu, M; Parvez, F; Slavkovich, V; Eunus, M; Ahmed, A; Segers, S; Argos, M; Islam, T; Rakibuz-Zaman, M; Hasan, R; Sarwar, G; Levy, D; Graziano, J; Ahsan, H. (2013b). A prospective study of arsenic exposure, arsenic methylation capacity, and risk of cardiovascular disease in Bangladesh. Environ Health Perspect 121: 832-838. <http://dx.doi.org/10.1289/ehp.1205797>

Chen, Y; Wu, F; Parvez, F; Ahmed, A; Eunus, M; McClintock, TR; Patwary, TI; Islam, T; Ghosal, AK; Islam, S; Hasan, R; Levy, D; Sarwar, G; Slavkovich, V; van Geen, A; Graziano, JH; Ahsan, H. (2013c). Arsenic exposure from drinking water and QT-interval prolongation: Results from the Health Effects of Arsenic Longitudinal Study. Environ Health Perspect 121: 427-432. <http://dx.doi.org/10.1289/ehp.1205197>

Cheng, PS; Weng, SF; Chiang, CH; Lai, FJ. (2015). Relationship between arsenic-containing drinking water and skin cancers in the arseniasis endemic areas in Taiwan. J Dermatol 43: 181-186. <http://dx.doi.org/10.1111/1346-8138.13058>

Cheng, TJ; Ke, DS; Guo, HR. (2010). The association between arsenic exposure from drinking water and cerebrovascular disease mortality in Taiwan. Water Res 44: 5770-5776. <http://dx.doi.org/10.1016/j.watres.2010.05.040>

Cherry, N; Shaik, K; Mcdonald, C; Chowdhury, Z. (2010). Manganese, arsenic, and infant mortality in Bangladesh: An ecological analysis. Arch Environ Occup Health 65: 148-153. <http://dx.doi.org/10.1080/19338240903390362>

Cherry, N; Shaikh, K; McDonald, C; Chowdhury, Z. (2008). Stillbirth in rural Bangladesh: Arsenic exposure and other etiological factors: A report from Gonoshasthaya Kendra. Bull World Health Organ 86: 172-177. <http://dx.doi.org/10.2471/blt.07.043083>

Chiang, HS; Guo, HR; Hong, CL; Lin, SM; Lee, EF. (1993). The incidence of bladder cancer in the black foot disease endemic area in Taiwan. Br J Urol 71: 274-278. <http://dx.doi.org/10.1111/j.1464-410X.1993.tb15942.x>

Chiazze, L, Jr; Watkins, DK; Fryar, C. (1997). Historical cohort mortality study of a continuous filament fiberglass manufacturing plant. I. White men. J Occup Environ Med 39: 432-441. <http://dx.doi.org/10.1097/00043764-199705000-00009>

Chiou, H, -Y; Chiou, S, -T; Hsu, Y, -H; Chou, Y, -L; Tseng, C, -H; Wei, M, -L; Chen, C, -J. (2001a). Incidence of transitional cell carcinoma and arsenic in drinking water: A follow-up study of 8,102 residents in an arseniasis-endemic area in northeastern Taiwan. Am J Epidemiol 153: 411-418. <http://dx.doi.org/10.1093/aje/153.5.411>

Chiou, H, -Y; Huang, W, -I; Su, C, -L; Chang, S, -F; Hsu, Y, -H; Chen, C, -J. (1997). Dose-response relationship between prevalence of cerebrovascular disease and ingested inorganic arsenic. Stroke 28: 1717-1723. <http://dx.doi.org/10.1161/01.STR.28.9.1717>

Chiou, HY; Hsueh, YM; Liaw, KF; Horng, SF; Chiang, MH; Pu, YS; Lin, JSN; Huang, CH; Chen, CJ. (1995). Incidence of internal cancers and ingested inorganic arsenic: A seven-year follow-up study in Taiwan. Cancer Res 55: 1296-1300.

Chiou, HY; Wang, IH; Hsueh, YM; Chiou, ST; Chou, YL; Teh, HW; Chen, CJ. (2001b). Arsenic exposure, null genotypes of glutathione S-transferase M1, T1 and P1, and risk of carotid atherosclerosis among residents in the Lanyang Basin of Taiwan. In WR Chappell; CO Abernathy; RL Calderon (Eds.), Arsenic Exposure and Health Effects IV (pp. 207-219). Amsterdam, The Netherlands: Elsevier Science.

Chiou, J, -M; Wang, S, -L; Chen, C, -J; Deng, C, -R; Lin, W; Tai, T, -Y. (2005). Arsenic ingestion and increased microvascular disease risk: Observations from the south-western arseniasis-endemic area in Taiwan. Int J Epidemiol 34: 936-943. <http://dx.doi.org/10.1093/ije/dyi108>

Chiu, H, -F; Chang, C, -C; Tsai, S, -S; Yang, C, -Y. (2006). Does arsenic exposure increase the risk for diabetes mellitus? J Occup Environ Med 48: 63-67. <http://dx.doi.org/10.1097/01.jom.0000184854.75053.03>

Chiu, H, -F; Ho, S, -C; Yang, C, -Y. (2004). Lung cancer mortality reduction after installation of tap-water supply system in an arseniasis-endemic area in southwestern Taiwan. Lung Cancer 46: 265-270. <http://dx.doi.org/10.1016/j.lungcan.2004.05.012>

Chiu, H, -F; Lin, M, -C; Yang, C, -Y. (2007). Primary intracerebral hemorrhage mortality reduction after installation of a tap-water supply system in an arseniasis-endemic area in southwestern Taiwan. J Toxicol Environ Health A 70: 539-546. <http://dx.doi.org/10.1080/15287390600870940>

Chung, C, -J; Huang, C, -Y; Pu, Y, -S; Shiue, H, -S; Su, C, -T; Hsueh, Y, -M. (2013). The effect of cigarette smoke and arsenic exposure on urothelial carcinoma risk is modified by glutathione S-transferase M1 gene null genotype. Toxicol Appl Pharmacol 266: 254-259. <http://dx.doi.org/10.1016/j.taap.2012.11.005>

Chung, C, -J; Pu, Y, -S; Chen, Y, -T; Su, C, -T; Wu, C, -C; Shiue, H, -S; Huang, C, -Y; Hsueh, Y, -M. (2011). Protective effects of plasma alpha-tocopherols on the risk of inorganic arsenic-related urothelial carcinoma. Sci Total Environ 409: 1039-1045. <http://dx.doi.org/10.1016/j.scitotenv.2010.11.037>

Coronado-González, JA; Del Razo, LM; García-Vargas, G; Sanmiguel-Salazar, F; Escobedo-de la Peña, J. (2007). Inorganic arsenic exposure and type 2 diabetes mellitus in Mexico. Environ Res 104: 383-389. <http://dx.doi.org/10.1016/j.envres.2007.03.004>

Currier, JM; Ishida, MC; González-Horta, C; Sánchez-Ramírez, B; Ballinas-Casarrubias, L; Gutiérrez-Torres, DS; Cerón, RH; Morales, DV; Terrazas, FA; Del Razo, LM; García-Vargas, GG; Saunders, RJ; Drobná, Z; Fry, RC; Matoušek, T; Buse, JB; Mendez, MA; Loomis, D; Stýblo, M. (2014). Associations between arsenic species in exfoliated urothelial cells and prevalence of diabetes among residents of Chihuahua, Mexico. Environ Health Perspect 122: 1088-1094. <http://dx.doi.org/10.1289/ehp.1307756>

D'Ippoliti, D; Santelli, E; De Sario, M; Scortichini, M; Davoli, M; Michelozzi, P. (2015). Arsenic in Drinking Water and Mortality for Cancer and Chronic Diseases in Central Italy, 1990-2010. PLoS ONE 10: e0138182. <http://dx.doi.org/10.1371/journal.pone.0138182>

Das, D; Bindhani, B; Mukherjee, B; Saha, H; Biswas, P; Dutta, K; Prasad, P; Sinha, D; Ray, MR. (2014). Chronic low-level arsenic exposure reduces lung function in male population without skin lesions. Int J Public Health 59: 655-663. <http://dx.doi.org/10.1007/s00038-014-0567-5>

Das, N; Paul, S; Chatterjee, D; Banerjee, N; Majumder, NS; Sarma, N; Sau, TJ; Basu, S; Banerjee, S; Majumder, P; Bandyopadhyay, AK; States, JC; Giri, AK. (2012). Arsenic exposure through drinking water increases the risk of liver and cardiovascular diseases in the population of West Bengal, India. BMC Public Health 12: 639. <http://dx.doi.org/10.1186/1471-2458-12-639>

Dastgiri, S; Mosaferi, M; Fizi, MAH; Olfati, N; Zolali, S; Pouladi, N; Azarfam, P. (2010). Arsenic exposure, dermatological lesions, hypertension, and chromosomal abnormalities among people in a rural community of northwest Iran. J Health Popul Nutr 28: 14-22. <http://dx.doi.org/10.3329/jhpn.v28i1.4519>

Dauphiné, DC; Ferreccio, C; Guntur, S; Yuan, Y; Hammond, SK; Balmes, J; Smith, AH; Steinmaus, C. (2011). Lung function in adults following in utero and childhood exposure to arsenic in drinking water: Preliminary findings. Int Arch Occup Environ Health 84: 591-600. <http://dx.doi.org/10.1007/s00420-010-0591-6>

Dauphiné, DC; Smith, AH; Yuan, Y; Balmes, JR; Bates, MN; Steinmaus, C. (2013). Case-control study of arsenic in drinking water and lung cancer in California and Nevada. Int J Environ Res Public Health 10: 3310-3324. <http://dx.doi.org/10.3390/ijerph10083310>

Davis, MA; Higgins, J; Li, Z; Gilbert-Diamond, D; Baker, ER; Das, A; Karagas, MR. (2015). Preliminary analysis of in utero low-level arsenic exposure and fetal growth using biometric measurements extracted from fetal ultrasound reports. Environ Health 14: 12. <http://dx.doi.org/10.1186/1476-069X-14-12>

De, BK; Majumdar, D; Sen, S; Guru, S; Kundu, S. (2004). Pulmonary involvement in chronic arsenic poisoning from drinking contaminated ground-water. J Assoc Physicians India 52: 395-400.

Del Razo, LM; García-Vargas, GG; Valenzuela, OL; Castellanos, EH; Sánchez-Peña, LC; Currier, JM; Drobná, Z; Loomis, D; Stýblo, M. (2011). Exposure to arsenic in drinking water is associated with increased prevalence of diabetes: A cross-sectional study in the Zimapán and Lagunera regions in Mexico. Environ Health 10: 73. <http://dx.doi.org/10.1186/1476-069X-10-73>

Drobná, Z; Del Razo, LM; Garcia-Vargas, G; Sánchez-Ramírez, B; González-Horta, C; Ballinas-Casarrubias, L; Loomis, D; Stýblo, M. (2012). Identification of the GST-T1 and GST-M1 null genotypes using high resolution melting analysis. Chem Res Toxicol 25: 216-224. <http://dx.doi.org/10.1021/tx200457u>

El-Baz, MA; El-Deeb, TS; El-Noweihi, AM; Mohany, KM; Shaaban, OM; Abbas, AM. (2015). Environmental factors and apoptotic indices in patients with intrauterine growth retardation: A nested case-control study. Environ Toxicol Pharmacol 39: 589-596. <http://dx.doi.org/10.1016/j.etap.2015.01.009>

Engel, RR; Smith, AH. (1994). Arsenic in drinking water and mortality from vascular disease: An ecologic analysis in 30 counties in the United States. Arch Environ Health 49: 418-427. <http://dx.doi.org/10.1080/00039896.1994.9954996>

Enterline, PE; Day, R; Marsh, GM. (1995). Cancers related to exposure to arsenic at a copper smelter. Occup Environ Med 52: 28-32. <http://dx.doi.org/10.1136/oem.52.1.28>

Enterline, PE; Marsh, GM. (1982). Cancer among workers exposed to arsenic and other substances in a copper smelter. Am J Epidemiol 116: 895-911.

Enterline, PE; Marsh, GM; Esmen, NA; Henderson, VL; Callahan, CM; Paik, M. (1987). Some effects of cigarette smoking, arsenic, and SO2 on mortality among US copper smelter workers. J Occup Med 29: 831-838.

Ettinger, AS; Zota, AR; Amarasiriwardena, CJ; Hopkins, MR; Schwartz, J; Hu, H; Wright, RO. (2009). Maternal arsenic exposure and impaired glucose tolerance during pregnancy. Environ Health Perspect 117: 1059-1064. <http://dx.doi.org/10.1289/ehp0800533>

Fan, YG; Hu, P; Jiang, Y; Chang, RS; Yao, SX; Wang, W; He, J; Prorok, P; Qiao, YL. (2009). Association between sputum atypia and lung cancer risk in an occupational cohort in Yunnan, China. Chest 135: 778-785. <http://dx.doi.org/10.1378/chest.08-1469>

Farzan, SF; Chen, Y, u; Rees, J. R.; Zens, MS; Karagas, MR. (2015a). Risk of death from cardiovascular disease associated with low-level arsenic exposure among long-term smokers in a US population-based study. Toxicol Appl Pharmacol 287: 93-97. <http://dx.doi.org/10.1016/j.taap.2015.05.013>

Farzan, SF; Chen, Y; Wu, F; Jiang, J; Liu, M; Baker, E; Korrick, SA; Karagas, MR. (2015b). Blood pressure changes in relation to arsenic exposure in a U.S. pregnancy cohort. Environ Health Perspect 123: 999-1006. <http://dx.doi.org/10.1289/ehp.1408472>

Farzan, SF; Korrick, S; Li, Z; Enelow, R; Gandolfi, AJ; Madan, J; Nadeau, K; Karagas, MR. (2013). In utero arsenic exposure and infant infection in a United States cohort: A prospective study. Environ Res 126: 24-30. <http://dx.doi.org/10.1016/j.envres.2013.05.001>

Farzan, SF; Li, Z; Korrick, SA; Spiegelman, D; Enelow, R; Nadeau, K; Baker, E; Karagas, MR. (2015c). Infant infections and respiratory symptoms in relation to in utero arsenic exposure in a U.S. cohort. Environ Health Perspect 124: 840-847. <http://dx.doi.org/10.1289/ehp.1409282>

Fatmi, Z; Abbasi, IN; Ahmed, M; Kazi, A; Kayama, F. (2013). Burden of skin lesions of arsenicosis at higher exposure through groundwater of taluka Gambat district Khairpur, Pakistan: A cross-sectional survey. Environ Geochem Health 35: 341-346. <http://dx.doi.org/10.1007/s10653-012-9498-3>

Fatmi, Z; Azam, I; Ahmed, F; Kazi, A; Gill, AB; Kadir, MM; Ahmed, M; Ara, N; Janjua, NZ; Pakistan, CGfAMi. (2009). Health burden of skin lesions at low arsenic exposure through groundwater in Pakistan. Is river the source? Environ Res 109: 575-581. <http://dx.doi.org/10.1016/j.envres.2009.04.002>

Feki-Tounsi, M; Olmedo, P; Gil, F; Khlifi, R; Mhiri, MN; Rebai, A; Hamza-Chaffai, A. (2013). Low-level arsenic exposure is associated with bladder cancer risk and cigarette smoking: A case-control study among men in Tunisia. Environ Sci Pollut Res Int 20: 3923-3931. <http://dx.doi.org/10.1007/s11356-012-1335-9>

Feng, W; Cui, X; Liu, B; Liu, C; Xiao, Y; Lu, W; Guo, H; He, M; Zhang, X; Yuan, J; Chen, W; Wu, T. (2015). Association of Urinary Metal Profiles with Altered Glucose Levels and Diabetes Risk: A Population-Based Study in China. PLoS ONE 10: e0123742. <http://dx.doi.org/10.1371/journal.pone.0123742>

Fernández, MI; López, JF; Vivaldi, B; Coz, F. (2012). Long-term impact of arsenic in drinking water on bladder cancer health care and mortality rates 20 years after end of exposure. J Urol 187: 856-861. <http://dx.doi.org/10.1016/j.juro.2011.10.157>

Ferreccio, C; González, C; Milosavjlevic, V; Marshall, G; Sancha, AM; Smith, AH. (2000). Lung cancer and arsenic concentrations in drinking water in Chile. Epidemiology 11: 673-679. <http://dx.doi.org/10.1097/00001648-200011000-00010>

Ferreccio, C; González Psych, C; Milosavjlevic Stat, V; Marshall Gredis, G; Sancha, AM. (1998). Lung cancer and arsenic exposure in drinking water: A case-control study in northern Chile. Cad Saude Publica 14: 193-198. <http://dx.doi.org/10.1590/S0102-311X1998000700021>

Ferreccio, C; Smith, AH; Durán, V; Barlaro, T; Benítez, H; Valdés, R; Aguirre, JJ; Moore, LE; Acevedo, J; Vásquez, MI; Pérez, L; Yuan, Y; Liaw, J; Cantor, KP; Steinmaus, C. (2013a). Case-control study of arsenic in drinking water and kidney cancer in uniquely exposed Northern Chile. Am J Epidemiol 178: 813-818. <http://dx.doi.org/10.1093/aje/kwt059>

Ferreccio, C; Yuan, Y; Calle, J; Benítez, H; Parra, RL; Acevedo, J; Smith, AH; Liaw, J; Steinmaus, C. (2013b). Arsenic, tobacco smoke, and occupation: Associations of multiple agents with lung and bladder cancer. Epidemiology 24: 898-905. <http://dx.doi.org/10.1097/EDE.0b013e31829e3e03>

Feseke, SK; St-Laurent, J; Anassour-Sidi, E; Ayotte, P; Bouchard, M; Levallois, P. (2015). Arsenic exposure and type 2 diabetes: Results from the 2007-2009 Canadian Health Measures Survey. 35: 63-72.

García-Esquinas, E; Pollán, M; Umans, JG; Francesconi, KA; Goessler, W; Guallar, E; Howard, B; Farley, J; Best, LG; Navas-Acien, A. (2013). Arsenic exposure and cancer mortality in a US-based prospective cohort: The strong heart study. Cancer Epidemiol Biomarkers Prev 22: 1944-1953. <http://dx.doi.org/10.1158/1055-9965.EPI-13-0234-T>

García-Vargas, GG; Del Razo, LM; Cebrián, ME; Albores, A; Ostrosky-Wegman, P; Montero, R; Gonsebatt, ME; Lim, CK; De Matteis, F. (1994). Altered urinary porphyrin excretion in a human population chronically exposed to arsenic in Mexico. Hum Exp Toxicol 13: 839-847. <http://dx.doi.org/10.1177/096032719401301204>

Gardner, RM; Kippler, M; Tofail, F; Bottai, M; Hamadani, J; Grandér, M; Nermell, B; Palm, B; Rasmussen, KM; Vahter, M. (2013). Environmental exposure to metals and children's growth to age 5 years: A prospective cohort study. Am J Epidemiol 177: 1356-1367. <http://dx.doi.org/10.1093/aje/kws437>

Gelmann, ER; Gurzau, E; Gurzau, A; Goessler, W; Kunrath, J; Yeckel, CW; Mccarty, KM. (2013). A pilot study: The importance of inter-individual differences in inorganic arsenic metabolism for birth weight outcome. Environ Toxicol Pharmacol 36: 1266-1275. <http://dx.doi.org/10.1016/j.etap.2013.10.006>

Ghosh, A. (2013). Evaluation of chronic arsenic poisoning due to consumption of contaminated ground water in West Bengal, India. 4: 976-979.

Ghosh, P; Banerjee, M; De Chaudhuri, S; Chowdhury, R; Das, JK; Mukherjee, A; Sarkar, AK; Mondal, L; Baidya, K; Sau, TJ; Banerjee, A; Basu, A; Chaudhuri, K; Ray, K; Giri, AK. (2007). Comparison of health effects between individuals with and without skin lesions in the population exposed to arsenic through drinking water in West Bengal, India. J Expo Sci Environ Epidemiol 17: 215-223. <http://dx.doi.org/10.1038/sj.jes.7500510>

Gilbert-Diamond, D; Li, Z; Perry, AE; Spencer, SK; Gandolfi, AJ; Karagas, MR. (2013). A population-based case-control study of urinary arsenic species and squamous cell carcinoma in New Hampshire, USA. Environ Health Perspect 121: 11541160. <http://dx.doi.org/10.1289/ehp.1206178>

Gong, G; O'Bryant, SE. (2012). Low-level arsenic exposure, AS3MT gene polymorphism and cardiovascular diseases in rural Texas counties. Environ Res 113: 52-57. <http://dx.doi.org/10.1016/j.envres.2012.01.003>

Graham, JH; Mazzanti, GR; Helwig, EB. (1961). Chemistry of Bowen's disease: Relationship to arsenic. J Invest Dermatol 37: 317-332. <http://dx.doi.org/10.1038/jid.1961.127>

Gribble, MO; Howard, BV; Umans, JG; Shara, NM; Francesconi, KA; Goessler, W; Crainiceanu, CM; Silbergeld, EK; Guallar, E; Navas-Acien, A. (2012). Arsenic exposure, diabetes prevalence, and diabetes control in the strong heart study. Am J Epidemiol 176: 865-874. <http://dx.doi.org/10.1093/aje/kws153>

Grimsrud, TK; Berge, SR; Haldorsen, T; Andersen, A. (2005). Can lung cancer risk among nickel refinery workers be explained by occupational exposures other than nickel? Epidemiology 16: 146-154. <http://dx.doi.org/10.1097/01.ede.0000152902.48916.d7>

Guan, H; Piao, F; Zhang, X; Li, X; Li, Q; Xu, L; Kitamura, F; Yokoyama, K. (2012). Prenatal exposure to arsenic and its effects on fetal development in the general population of Dalian. Biol Trace Elem Res 149: 10-15. <http://dx.doi.org/10.1007/s12011-012-9396-7>

Guha Mazumder, D; Purkayastha, I; Ghose, A; Mistry, G; Saha, C; Nandy, AK; Das, A; Majumdar, KK. (2012). Hypertension in chronic arsenic exposure: A case control study in West Bengal. J Environ Sci Health A Tox Hazard Subst Environ Eng 47: 1514-1520. <http://dx.doi.org/10.1080/10934529.2012.680329>

Guha Mazumder, DN; Deb, D; Biswas, A; Saha, C; Nandy, A; Ganguly, B; Ghose, A; Bhattacharya, K; Majumdar, KK. (2013). Evaluation of dietary arsenic exposure and its biomarkers: A case study of West Bengal, India. J Environ Sci Health A Tox Hazard Subst Environ Eng 48: 896-904. <http://dx.doi.org/10.1080/10934529.2013.761495>

Guha Mazumder, DN; Ghosh, A; Majumdar, KK; Ghosh, N; Saha, C; Guha Mazumder, RN. (2010). Arsenic contamination of ground water and its health impact on population of district of Nadia, West Bengal, India. Indian J Community Med 35: 331-338. <http://dx.doi.org/10.4103/0970-0218.66897>

Guha Mazumder, DN; Haque, R; Ghosh, N; De, BK; Santra, A; Chakraborti, D; Smith, AH. (2000). Arsenic in drinking water and the prevalence of respiratory effects in West Bengal, India. Int J Epidemiol 29: 1047-1052. <http://dx.doi.org/10.1093/ije/29.6.1047>

Guha Mazumder, DN; Haque, R; Ghosh, N; De, BK; Santra, A; Chakraborty, D; Smith, AH. (1998). Arsenic levels in drinking water and the prevalence of skin lesions in West Bengal, India. Int J Epidemiol 27: 871-877. <http://dx.doi.org/10.1093/ije/27.5.871>

Guha Mazumder, DN; Majumdar, KK; Santra, SC; Kol, H; Vicheth, C. (2009). Occurrence of arsenicosis in a rural village of Cambodia. J Environ Sci Health A Tox Hazard Subst Environ Eng 44: 480-487. <http://dx.doi.org/10.1080/10934520902719886>

Guha Mazumder, DN; Steinmaus, C; Bhattacharya, P; von Ehrenstein, OS; Ghosh, N; Gotway, M; Sil, A; Balmes, JR; Haque, R; Hira-Smith, MM; Smith, AH. (2005). Bronchiectasis in persons with skin lesions resulting from arsenic in drinking water. Epidemiology 16: 760-765. <http://dx.doi.org/10.1097/01.ede.0000181637.10978.e6>

Gunduz, O; Bakar, C; Simsek, C; Baba, A; Elci, A; Gurleyuk, H; Mutlu, M; Cakir, A. (2015). Statistical analysis of causes of death (2005-2010) in villages of Simav Plain, Turkey, with high arsenic levels in drinking water supplies. Arch Environ Occup Health 70: 35-46. <http://dx.doi.org/10.1080/19338244.2013.872076>

Guo, HR. (2003). The lack of a specific association between arsenic in drinking water and hepatocellular carcinoma. J Hepatol 39: 383-388. <http://dx.doi.org/10.1016/S0168-8278(03)00297-6>

Guo, HR. (2004). Arsenic level in drinking water and mortality of lung cancer (Taiwan). Cancer Causes Control 15: 171-177. <http://dx.doi.org/10.1023/B:CACO.0000019503.02851.b0>

Guo, HR; Chiang, HS; Hu, H; Lipsitz, SR; Monson, RR. (1997). Arsenic in drinking water and incidence of urinary cancers. Epidemiology 8: 545-550.

Guo, HR; Lipsitz, SR; Hu, H; Monson, RR. (1998). Using ecological data to estimate a regression model for individual data: The association between arsenic in drinking water and incidence of skin cancer. Environ Res 79: 82-93. <http://dx.doi.org/10.1006/enrs.1998.3863>

Guo, HR; Wang, NS; Hu, H; Monson, RR. (2004). Cell type specificity of lung cancer associated with arsenic ingestion. Cancer Epidemiol Biomarkers Prev 13: 638-643.

Guo, HR; Yu, HS; Hu, H; Monson, RR. (2001). Arsenic in drinking water and skin cancers: Cell-type specificity (Taiwan, R.O.C.). Cancer Causes Control 12: 909-916. <http://dx.doi.org/10.1023/A:1013712203455>

Guo, JX; Hu, L; Yand, PZ; Tanabe, K; Miyatalre, M; Chen, Y. (2007). Chronic arsenic poisoning in drinking water in Inner Mongolia and its associated health effects. J Environ Sci Health A Tox Hazard Subst Environ Eng 42: 1853-1858. <http://dx.doi.org/10.1080/10934520701566918>

Guo, X; Fujino, Y; Ye, X; Liu, J; Yoshimura, T; Group, JIMAPS. (2006a). Association between multi-level inorganic arsenic exposure from drinking water and skin lesions in China. Int J Environ Res Public Health 3: 262-267. <http://dx.doi.org/10.3390/ijerph2006030031>

Guo, X; Liu, Z; Huang, C; You, L. (2006b). Levels of arsenic in drinking-water and cutaneous lesions in Inner Mongolia. J Health Popul Nutr 24: 214-220.

Halatek, T; Sinczuk-Walczak, H; Janasik, B; Trzcinka-Ochocka, M; Winnicka, R; Wasowicz, W. (2014). Health effects and arsenic species in urine of copper smelter workers. J Environ Sci Health A Tox Hazard Subst Environ Eng 49: 787-797. <http://dx.doi.org/10.1080/10934529.2014.882207>

Halatek, T; Sinczuk-Walczak, H; Rabieh, S; Wasowicz, W. (2009). Association between occupational exposure to arsenic and neurological, respiratory and renal effects. Toxicol Appl Pharmacol 239: 193-199. <http://dx.doi.org/10.1016/j.taap.2009.04.022>

Hall, M; Chen, Y; Ahsan, H; Slavkovich, V; van Geen, A; Parvez, F; Graziano, J. (2006). Blood arsenic as a biomarker of arsenic exposure: Results from a prospective study. Toxicology 225: 225-233. <http://dx.doi.org/10.1016/j.tox.2006.06.010>

Han, YY; Weissfeld, JL; Davis, DL; Talbott, EO. (2009). Arsenic levels in ground water and cancer incidence in Idaho: An ecologic study. Int Arch Occup Environ Health 82: 843-849. <http://dx.doi.org/10.1007/s00420-008-0362-9>

Haque, R; Mazumder, DNG; Samanta, S; Ghosh, N; Kalman, D; Smith, MM; Mitra, S; Santra, A; Lahiri, S; Das, S; De, BK; Smith, AH. (2003). Arsenic in drinking water and skin lesions: Dose-response data from West Bengal, India. Epidemiology 14: 174-182. <http://dx.doi.org/10.1097/01.EDE.0000040361.55051.54>

Hashim, JH; Radzi, RS; Aljunid, SM; Nur, AM; Ismail, A; Baguma, D; Sthiannopkao, S; Phan, K; Wong, MH; Sao, V; Yasin, MS. (2013). Hair arsenic levels and prevalence of arsenicosis in three Cambodian provinces. Sci Total Environ 463-464: 1210-1216. <http://dx.doi.org/10.1016/j.scitotenv.2013.04.084>

Hawkesworth, S; Wagatsuma, Y; Kippler, M; Fulford, AJ; Arifeen, SE; Persson, LA; Moore, SE; Vahter, M. (2013). Early exposure to toxic metals has a limited effect on blood pressure or kidney function in later childhood, rural Bangladesh. Int J Epidemiol 42: 176-185. <http://dx.doi.org/10.1093/ije/dys215>

Heaney, CD; Kmush, B; Navas-Acien, A; Francesconi, K; Gössler, W; Schulze, K; Fairweather, D; Mehra, S; Nelson, KE; Klein, SL; Li, W; Ali, H; Shaikh, S; Merrill, RD; Wu, L; West, KP; Christian, P; Labrique, AB. (2015). Arsenic exposure and hepatitis E virus infection during pregnancy. Environ Res 142: 273-280. <http://dx.doi.org/10.1016/j.envres.2015.07.004>

Heck, JE; Andrew, AS; Onega, T; Rigas, JR; Jackson, BP; Karagas, MR; Duell, EJ. (2009). Lung cancer in a U.S. population with low to moderate arsenic exposure. Environ Health Perspect 117: 1718-1723. <http://dx.doi.org/10.1289/ehp.0900566>

Hertz-Picciotto, I; Arrighi, HM; Hu, SW. (2000). Does arsenic exposure increase the risk for circulatory disease? Am J Epidemiol 151: 174-181. <http://dx.doi.org/10.1093/oxfordjournals.aje.a010185>

Hinwood, AL; Jolley, DJ; Sim, MR. (1999). Cancer incidence and high environmental arsenic concentrations in rural populations: Results of an ecological study. Int J Environ Health Res 9: 131-141. <http://dx.doi.org/10.1080/09603129973272>

Hon, KL; Lui, H; Wang, SS; Lam, HS; Leung, TF. (2012). Fish consumption, fish atopy and related heavy metals in childhood eczema. Iran J Allergy Asthma Immunol 11: 230-235. <http://dx.doi.org/011.03/ijaai.230235>

Hopenhayn-Rich, C; Biggs, ML; Fuchs, A; Bergoglio, R; Tello, EE; Nicolli, H; Smith, AH. (1996). Bladder cancer mortality associated with arsenic in drinking water in Argentina. Epidemiology 7: 117-124. <http://dx.doi.org/10.1097/00001648-199603000-00003>

Hopenhayn-Rich, C; Biggs, ML; Smith, AH. (1998). Lung and kidney cancer mortality associated with arsenic in drinking water in Córdoba, Argentina. Int J Epidemiol 27: 561-569. <http://dx.doi.org/10.1093/ije/27.4.561>

Hopenhayn-Rich, C; Browning, SR; Hertz-Picciotto, I; Ferreccio, C; Peralta, C; Gibb, H. (2000). Chronic arsenic exposure and risk of infant mortality in two areas of Chile. Environ Health Perspect 108: 667-673. <http://dx.doi.org/10.1289/ehp.00108667>

Hopenhayn-Rich, C; Hertz-Picciotto, I; Browning, S; Ferreccio, C; Peralta, C. (1999). Reproductive and developmental effects associated with chronic arsenic exposure. In Arsenic Exposure and Health Effects III. New York, NY: Elsevier. <http://www.sciencedirect.com/science/book/9780080436487#ancST4>

Hopenhayn, C; Ferreccio, C; Browning, SR; Huang, B; Peralta, C; Gibb, H; Hertz-Picciotto, I. (2003). Arsenic exposure from drinking water and birth weight. Epidemiology 14: 593-602. <http://dx.doi.org/10.1097/01.ede.0000072104.65240.69>

Hsieh, FI; Hwang, TS; Hsieh, YC; Lo, HC; Su, CT; Hsu, HS; Chiou, HY; Chen, CJ. (2008a). Risk of erectile dysfunction induced by arsenic exposure through well water consumption in Taiwan. Environ Health Perspect 116: 532-536. <http://dx.doi.org/10.1289/ehp.10930>

Hsieh, Y, -C; Hsieh, F, -I; Lien, L, -M; Chou, Y, -L; Chiou, H, -Y; Chen, C, -J. (2008b). Risk of carotid atherosclerosis associated with genetic polymorphisms of apolipoprotein E and inflammatory genes among arsenic exposed residents in Taiwan. Toxicol Appl Pharmacol 227: 1-7. <http://dx.doi.org/10.1016/j.taap.2007.10.013>

Hsu, L, -I; Chen, G, -S; Lee, C, -H; Yang, T, -Y; Chen, Y, -H; Wang, Y, -H; Hsueh, Y, -M; Chiou, H, -Y; Wu, M, -M; Chen, C, -J. (2013a). Use of arsenic-induced palmoplantar hyperkeratosis and skin cancers to predict risk of subsequent internal malignancy. Am J Epidemiol 177: 202-212. <http://dx.doi.org/10.1093/aje/kws369>

Hsu, L, -I; Wang, Y, -H; Chiou, H, -Y; Wu, M, -M; Yang, T, -Y; Chen, Y, -H; Tseng, C, -H; Chen, C, -J. (2013b). The association of diabetes mellitus with subsequent internal cancers in the arsenic-exposed area of Taiwan. J Asian Earth Sci 73: 452-459. <http://dx.doi.org/10.1016/j.jseaes.2013.04.048>

Hsu, L; Chiu, A; Huan, S; Chen, C; Wang, Y; Hsieh, F; Chou, W; Wang, L; Chen, C. (2008). SNPs of GSTM1, T1, P1, epoxide hydrolase and DNA repair enzyme XRCC1 and risk of urinary transitional cell carcinoma in southwestern Taiwan. Toxicol Appl Pharmacol 228: 144-155. <http://dx.doi.org/10.1016/j.taap.2007.12.003>

Hsu, LI; Wu, MM; Wang, YH; Lee, C; Yang, T; Hsiao, B; Chen, C. (2015). Association of environmental arsenic exposure, genetic polymorphisms of susceptible genes, and skin cancers in Taiwan. BioMed Res Int 2015: 892579. <http://dx.doi.org/10.1155/2015/892579>

Hsueh, Y, -M; Wu, W, -L; Huang, Y, -L; Chiou, H, -Y; Tseng, C, -H; Chen, C, -J. (1998). Low serum carotene level and increased risk of ischemic heart disease related to long-term arsenic exposure. Atherosclerosis 141: 249-257. <http://dx.doi.org/10.1016/S0021-9150(98)00178-6>

Hsueh, YM; Cheng, GS; Wu, MM; Yu, HS; Kuo, TL; Chen, CJ. (1995). Multiple risk factors associated with arsenic-induced skin cancer: Effects of chronic liver disease and malnutritional status. Br J Cancer 71: 109-114. <http://dx.doi.org/10.1038/bjc.1995.22>

Hsueh, YM; Chiou, HY; Huang, YL; Wu, WL; Huang, CC; Yang, MH; Lue, LC; Chen, GS; Chen, CJ. (1997). Serum beta-carotene level, arsenic methylation capability, and incidence of skin cancer. Cancer Epidemiol Biomarkers Prev 6: 589-596.

Hu, SW; Hertz-Picciotto, I; Siemiatycki, J. (1999). When to be skeptical of negative studies: Pitfalls in evaluating occupational risks using population-based case-control studies. Can J Public Health 90: 138-142.

Hu, X; Zheng, T; Cheng, Y; Holford, T; Lin, S; Leaderer, B; Qiu, J, ie; Bassig, BA; Shi, K; Zhang, Y; Niu, J; Zhu, Y; Li, Y; Guo, H; Chen, Q; Zhang, J; Xu, S; Jin, Y. (2015). Distributions of heavy metals in maternal and cord blood and the association with infant birth weight in China. J Reprod Med 60: 21-29.

Huang, C, -Y; Chu, J, -S; Pu, Y, -S; Yang, H, -Y; Wu, C, -C; Chung, C, -J; Hsueh, Y, -M. (2011). Effect of urinary total arsenic level and estimated glomerular filtration rate on the risk of renal cell carcinoma in a low arsenic exposure area. J Urol 185: 2040-2044. <http://dx.doi.org/10.1016/j.juro.2011.01.079>

Huang, C, -Y; Su, C, -T; Chung, C, -J; Pu, Y, -S; Chu, J, -S; Yang, H, -Y; Wu, C, -C; Hsueh, Y, -M. (2012). Urinary total arsenic and 8-hydroxydeoxyguanosine are associated with renal cell carcinoma in an area without obvious arsenic exposure. Toxicol Appl Pharmacol 262: 349-354. <http://dx.doi.org/10.1016/j.taap.2012.05.013>

Huang, JW; Cheng, YY; Sung, TC; Guo, HR; Sthiannopkao, S. (2014). Association between arsenic exposure and diabetes mellitus in Cambodia. BioMed Res Int 2014: 683124. <http://dx.doi.org/10.1155/2014/683124>

Huang, Y, -K; Huang, Y, -L; Hsueh, Y, -M; Yang, M, -H; Wu, M, -M; Chen, S, -Y; Hsu, L, -I; Chen, C, -J. (2008a). Arsenic exposure, urinary arsenic speciation, and the incidence of urothelial carcinoma: A twelve-year follow-up study. Cancer Causes Control 19: 829-839. <http://dx.doi.org/10.1007/s10552-008-9146-5>

Huang, Y, -K; Pu, Y, -S; Chung, C, -J; Shiue, H, -S; Yang, M, -H; Chen, C, -J; Hsueh, Y, -M. (2008b). Plasma folate level, urinary arsenic methylation profiles, and urothelial carcinoma susceptibility. Food Chem Toxicol 46: 929-938. <http://dx.doi.org/10.1016/j.fct.2007.10.017>

Huyck, KL; Kile, ML; Mahiuddin, G; Quamruzzaman, Q; Rahman, M; Breton, CV; Dobson, CB; Frelich, J; Hoffman, E; Yousuf, J; Afroz, S; Islam, S; Christiani, DC. (2007). Maternal arsenic exposure associated with low birth weight in Bangladesh. J Occup Environ Med 49: 1097-1104. <http://dx.doi.org/10.1097/JOM.0b013e3181566ba0>

Ihrig, MM; Shalat, SL; Baynes, C. (1998). A hospital-based case-control study of stillbirths and environmental exposure to arsenic using an atmospheric dispersion model linked to a geographical information system. Epidemiology 9: 290-294. <http://dx.doi.org/10.1097/00001648-199805000-00013>

Islam, LN; Nurun Nabi, AHM, N; Rahman, MM; Shamin, M; Zahid, H. (2007). Association of respiratory complications and elevated serum immunoglobulins with drinking water arsenic toxicity in human. J Environ Sci Health A Tox Hazard Subst Environ Eng 42: 1807-1814. <http://dx.doi.org/10.1080/10934520701566777>

Islam, MR; Khan, I; Attia, J; Hassan, SMN; Mcevoy, M; D'Este, C; Azim, S; Akhter, A; Akter, S; Shahidullah, SM; Milton, AH. (2012a). Association between hypertension and chronic arsenic exposure in drinking water: A cross-sectional study in Bangladesh. Int J Environ Res Public Health 9: 4522-4536. <http://dx.doi.org/10.3390/ijerph9124522>

Islam, R; Khan, I; Hassan, SN; Mcevoy, M; D'Este, C; Attia, J; Peel, R; Sultana, M; Akter, S; Milton, AH. (2012b). Association between type 2 diabetes and chronic arsenic exposure in drinking water: A cross sectional study in Bangladesh. Environ Health 11: 38. <http://dx.doi.org/10.1186/1476-069X-11-38>

James, KA; Byers, T; Hokanson, JE; Meliker, JR; Zerbe, GO; Marshall, JA. (2015). Association between Lifetime Exposure to Inorganic Arsenic in Drinking Water and Coronary Heart Disease in Colorado Residents. Environ Health Perspect 123: 128-134. <http://dx.doi.org/10.1289/ehp.1307839>

James, KA; Marshall, JA; Hokanson, JE; Meliker, JR; Zerbe, GO; Byers, TE. (2013). A case-cohort study examining lifetime exposure to inorganic arsenic in drinking water and diabetes mellitus. Environ Res 123: 33-38. <http://dx.doi.org/10.1016/j.envres.2013.02.005>

Järup, L; Pershagen, G. (1991). Arsenic exposure, smoking, and lung cancer in smelter workers--a case-control study [Erratum]. Am J Epidemiol 134: 545-551.

Jarup, L; Pershagen, G; Wall, S. (1989). Cumulative arsenic exposure and lung cancer in smelter workers: A dose-response study. Am J Ind Med 15: 31-41. <http://dx.doi.org/10.1002/ajim.4700150105>

Jensen, GE; Hansen, ML. (1998). Occupational arsenic exposure and glycosylated haemoglobin. Analyst 123: 77-80. <http://dx.doi.org/10.1039/a705699k>

Jiang, J; Liu, M; Parvez, F; Wang, B; Wu, F; Eunus, M; Bangalore, S; Newman, JD; Ahmed, A; Islam, T; Rakibuz-Zaman, M; Hasan, R; Sarwar, G; Levy, D; Slavkovich, V; Argos, M; Scannell Bryan, M; Farzan, SF; Hayes, RB; Graziano, JH; Ahsan, H; Chen, Y. (2015). Association between Arsenic Exposure from Drinking Water and Longitudinal Change in Blood Pressure among HEALS Cohort Participants. Environ Health Perspect 123: 806-812. <http://dx.doi.org/10.1289/ehp.1409004>

Jones, MR; Tellez-Plaza, M; Sharrett, AR; Guallar, E; Navas-Acien, A. (2011). Urine arsenic and hypertension in US adults: The 2003-2008 National Health and Nutrition Examination Survey. Epidemiology 22: 153-161. <http://dx.doi.org/10.1097/EDE.0b013e318207fdf2>

Jones, SR; Atkin, P; Holroyd, C; Lutman, E; Batlle, JVI; Wakeford, R; Walker, P. (2007). Lung cancer mortality at a UK tin smelter. Occup Med (Lond) 57: 238-245. <http://dx.doi.org/10.1093/occmed/kql153>

Jovanovic, D; Rasic-Milutinovic, Z; Paunovic, K; Jakovljevic, B; Plavsic, S; Milosevic, J. (2013). Low levels of arsenic in drinking water and type 2 diabetes in Middle Banat region, Serbia. Int J Hyg Environ Health 216: 50-55. <http://dx.doi.org/10.1016/j.ijheh.2012.01.001>

Jovanović, DD; Paunović, K; Manojlović, DD; Jakovljević, B; Rasic-Milutinović, Z; Dojcinović, BP. (2012). Arsenic in drinking water and acute coronary syndrome in Zrenjanin municipality, Serbia. Environ Res 117: 75-82. <http://dx.doi.org/10.1016/j.envres.2012.04.016>

Karagas, MR; Stukel, TA; Morris, JS; Tosteson, TD; Weiss, JE; Spencer, SK; Greenberg, ER. (2001). Skin cancer risk in relation to toenail arsenic concentrations in a US population-based case-control study. Am J Epidemiol 153: 559-565. <http://dx.doi.org/10.1093/aje/153.6.559>

Karagas, MR; Tosteson, TD; Morris, JS; Demidenko, E; Mott, LA; Heaney, J; Schned, A. (2004). Incidence of transitional cell carcinoma of the bladder and arsenic exposure in New Hampshire. Cancer Causes Control 15: 465-472. <http://dx.doi.org/10.1023/B:CACO.0000036452.55199.a3>

Karim, MR; Rahman, M; Islam, K; Mamun, AA; Hossain, S; Hossain, E; Aziz, A; Yeasmin, F; Agarwal, S; Hossain, MI; Saud, ZA; Nikkon, F; Hossain, M; Mandal, A; Jenkins, RO; Haris, PI; Miyataka, H; Himeno, S; Hossain, K. (2013). Increases in oxidized low density lipoprotein and other inflammatory and adhesion molecules with a concomitant decrease in high density lipoprotein in the individuals exposed to arsenic in Bangladesh. Toxicol Sci 135: 17-25. <http://dx.doi.org/10.1093/toxsci/kft130>

Khlifi, R; Olmedo, P; Gil, F; Feki-Tounsi, M; Hammami, B; Rebai, A; Hamza-Chaffai, A. (2014). Risk of laryngeal and nasopharyngeal cancer associated with arsenic and cadmium in the Tunisian population. Environ Sci Pollut Res Int 21: 2032-2042. <http://dx.doi.org/10.1007/s11356-013-2105-z>

Kile, ML; Houseman, EA; Baccarelli, AA; Quamruzzaman, Q; Rahman, M; Mostofa, G; Cardenas, A; Wright, RO; Christiani, DC. (2014a). Effect of prenatal arsenic exposure on DNA methylation and leukocyte subpopulations in cord blood. Epigenetics 9: 774-782. <http://dx.doi.org/10.4161/epi.28153>

Kile, ML; Rodrigues, EG; Mazumdar, M; Dobson, CB; Diao, N; Golam, M; Quamruzzaman, Q; Rahman, M; Christiani, DC. (2014b). A prospective cohort study of the association between drinking water arsenic exposure and self-reported maternal health symptoms during pregnancy in Bangladesh. Environ Health 13: 29. <http://dx.doi.org/10.1186/1476-069X-13-29>

Kim, NH; Mason, CC; Nelson, RG; Afton, SE; Essader, AS; Medlin, JE; Levine, KE; Hoppin, JA; Lin, C; Knowler, WC; Sandler, DP. (2013). Arsenic exposure and incidence of type 2 diabetes in Southwestern American Indians. Am J Epidemiol 177: 962-969. <http://dx.doi.org/10.1093/aje/kws329>

Kim, Y; Lee, BK. (2011). Association between urinary arsenic and diabetes mellitus in the Korean general population according to KNHANES 2008. Sci Total Environ 409: 4054-4062. <http://dx.doi.org/10.1016/j.scitotenv.2011.06.003>

Kippler, M; Wagatsuma, Y; Rahman, A; Nermell, B; Persson, LÅ; Raqib, R; Vahter, M. (2012). Environmental exposure to arsenic and cadmium during pregnancy and fetal size: A longitudinal study in rural Bangladesh. Reprod Toxicol 34: 504-511. <http://dx.doi.org/10.1016/j.reprotox.2012.08.002>

Knobeloch, LM; Zierold, KM; Anderson, HA. (2006). Association of arsenic-contaminated drinking-water with prevalence of skin cancer in Wisconsin's Fox River Valley. J Health Popul Nutr 24: 206-213.

Kunrath, J; Gurzau, E; Gurzau, A; Goessler, W; Gelmann, ER; Thach, TT; Mccarty, KM; Yeckel, CW. (2013). Blood pressure hyperreactivity: An early cardiovascular risk in normotensive men exposed to low-to-moderate inorganic arsenic in drinking water. J Hypertens 31: 361-369. <http://dx.doi.org/10.1097/HJH.0b013e32835c175f>

Kurttio, P; Pukkala, E; Kahelin, H; Auvinen, A; Pekkanen, J. (1999). Arsenic concentrations in well water and risk of bladder and kidney cancer in Finland. Environ Health Perspect 107: 705-710.

Kusiak, RA; Ritchie, AC; Muller, J; Springer, J. (1993). Mortality from lung cancer in Ontario uranium miners. Br J Ind Med 50: 920-928.

Kusiak, RA; Springer, J; Ritchie, AC; Muller, J. (1991). Carcinoma of the lung in Ontario gold miners: possible aetiological factors. Br J Ind Med 48: 808-817.

Kwok, RK; Kaufmann, RB; Jakariya, M. (2006). Arsenic in drinking-water and reproductive health outcomes: A study of participants in the Bangladesh integrated nutrition programme. J Health Popul Nutr 24: 190-205.

Kwok, RK; Mendola, P; Liu, ZY; Savitz, DA; Heiss, G; Ling, HL; Xia, Y; Lobdell, D; Zeng, D; Thorp, JM, Jr; Creason, JP; Mumford, JL. (2007). Drinking water arsenic exposure and blood pressure in healthy women of reproductive age in Inner Mongolia, China. Toxicol Appl Pharmacol 222: 337-343. <http://dx.doi.org/10.1016/j.taap.2007.04.003>

Lagerkvist, B; Linderholm, H; Nordberg, GF. (1986). Vasospastic tendency and Raynaud's phenomenon in smelter workers exposed to arsenic. Environ Res 39: 465-474. <http://dx.doi.org/10.1016/S0013-9351(86)80070-6>

Lagerkvist, BEA; Linderholm, H; Nordberg, GF. (1988). Arsenic and Raynaud's phenomenon: Vasospastic tendency and excretion of arsenic in smelter workers before and after the summer vacation. Int Arch Occup Environ Health 60: 361-364. <http://dx.doi.org/10.1007/BF00405671>

Lai, M, -S; Hsueh, Y, -M; Chen, C, -J; Shyu, M, -P; Chen, S, -Y; Kuo, T, -L; Wu, M, -M; Tai, T, -Y. (1994). Ingested inorganic arsenic and prevalence of diabetes mellitus. Am J Epidemiol 139: 484-492.

Laine, JE; Bailey, KA; Rubio-Andrade, M; Olshan, AF; Smeester, L; Drobná, Z; Herring, AH; Stýblo, M; García-Vargas, GG; Fry, RC. (2015). Maternal Arsenic Exposure, Arsenic Methylation Efficiency, and Birth Outcomes in the Biomarkers of Exposure to ARsenic (BEAR) Pregnancy Cohort in Mexico. Environ Health Perspect 123: 186-192. <http://dx.doi.org/10.1289/ehp.1307476>

Lamm, SH; Byrd, DM; Kruse, MB; Feinleib, M; Lai, SH. (2003). Bladder cancer and arsenic exposure: Differences in the two populations enrolled in a study in southwest Taiwan. Biomed Environ Sci 16: 355-368.

Lamm, SH; Engel, A; Kruse, MB; Feinleib, M; Byrd, DM; Lai, S; Wilson, R. (2004). Arsenic in drinking water and bladder cancer mortality in the United States: An analysis based on 133 U.S. counties and 30 years of observation. J Occup Environ Med 46: 298-306. <http://dx.doi.org/10.1097/01.jom.0000116801.67556.8f>

Lamm, SH; Luo, ZD; Bo, FB; Zhang, GY; Zhang, YM; Wilson, R; Byrd, DM; Lai, S; Li, FX; Polkanov, M; Tong, Y; Loo, L; Tucker, SB; (IMCAP), atIMCAP. (2007). An epidemiologic study of arsenic-related skin disorders and skin cancer and the consumption of arsenic-contaminated well Waters in Huhhot, Inner Mongolia, China. Hum Ecol Risk Assess 13: 713-746. <http://dx.doi.org/10.1080/10807030701456528>

Lee-Feldstein, A. (1989). A comparison of several measures of exposure to arsenic. Matched case-control study of copper smelter employees. Am J Epidemiol 129: 112-124.

Leonardi, G; Vahter, M; Clemens, F; Goessler, W; Gurzau, E; Hemminki, K; Hough, R; Koppova, K; Kumar, R; Rudnai, P; Surdu, S; Fletcher, T. (2012). Inorganic arsenic and basal cell carcinoma in areas of Hungary, Romania, and Slovakia: A case-control study. Environ Health Perspect 120: 721-726. <http://dx.doi.org/10.1289/ehp.1103534>

Lewis, DR; Southwick, JW; Ouellet-Hellstrom, R; Rench, J; Calderon, RL. (1999). Drinking water arsenic in Utah: A cohort mortality study. Environ Health Perspect 107: 359-365.

Li, W, -F; Sun, C, -W; Cheng, T, -J; Chang, K, -H; Chen, C, -J; Wang, S, -L. (2009). Risk of carotid atherosclerosis is associated with low serum paraoxonase (PON1) activity among arsenic exposed residents in Southwestern Taiwan. Toxicol Appl Pharmacol 236: 246-253. <http://dx.doi.org/10.1016/j.taap.2009.01.019>

Li, X; Li, B; Xi, S; Zheng, Q; Lv, X; Sun, G. (2013a). Prolonged environmental exposure of arsenic through drinking water on the risk of hypertension and type 2 diabetes. Environ Sci Pollut Res Int 20: 8151-8161. <http://dx.doi.org/10.1007/s11356-013-1768-9>

Li, X; Li, B; Xi, S; Zheng, Q; Wang, D; Sun, G. (2013b). Association of urinary monomethylated arsenic concentration and risk of hypertension: A cross-sectional study from arsenic contaminated areas in northwestern China. Environ Health 12: 37. <http://dx.doi.org/10.1186/1476-069X-12-37>

Li, Y; Wang, D; Li, X; Zheng, Q; Sun, G. (2015). A potential synergy between incomplete arsenic methylation capacity and demographic characteristics on the risk of hypertension: findings from a cross-sectional study in an arsenic-endemic area of inner Mongolia, China. Int J Environ Res Public Health 12: 3615-3632. <http://dx.doi.org/10.3390/ijerph120403615>

Liao, Y, -T; Chen, C, -J; Li, W, -F; Hsu, L, -Y; Tsai, L, -Y; Huang, Y, -L; Sun, C, -W; Chen, WJ; Wang, S, -L. (2012). Elevated lactate dehydrogenase activity and increased cardiovascular mortality in the arsenic-endemic areas of southwestern Taiwan. Toxicol Appl Pharmacol 262: 232-237. <http://dx.doi.org/10.1016/j.taap.2012.04.028>

Liao, YT; Li, WF; Chen, CJ; Prineas, RJ; Chen, WJ; Zhang, ZM; Sun, CW; Wang, SL. (2009). Synergistic effect of polymorphisms of paraoxonase gene cluster and arsenic exposure on electrocardiogram abnormality. Toxicol Appl Pharmacol 239: 178-183. <http://dx.doi.org/10.1016/j.taap.2008.12.017>

Liaw, J; Marshall, G; Yuan, Y; Ferreccio, C; Steinmaus, C; Smith, AH. (2008). Increased childhood liver cancer mortality and arsenic in drinking water in northern Chile. Cancer Epidemiol Biomarkers Prev 17: 1982-1987. <http://dx.doi.org/10.1158/1055-9965.EPI-07-2816>

Lin, H, -J; Sung, T, -I; Chen, C, -Y; Guo, H, -R. (2013). Arsenic levels in drinking water and mortality of liver cancer in Taiwan. J Hazard Mater 262: 1132-1138. <http://dx.doi.org/10.1016/j.jhazmat.2012.12.049>

Lin, HC; Huang, YK; Shiue, HS; Chen, LS; Choy, CS; Huang, SR; Han, BC; Hsueh, YM. (2014). Arsenic methylation capacity and obesity are associated with insulin resistance in obese children and adolescents. Food Chem Toxicol 74C: 60-67. <http://dx.doi.org/10.1016/j.fct.2014.08.018>

Lindberg, AL; Rahman, M; Persson, LA; Vahter, M. (2008). The risk of arsenic induced skin lesions in Bangladeshi men and women is affected by arsenic metabolism and the age at first exposure. Toxicol Appl Pharmacol 230: 9-16. <http://dx.doi.org/10.1016/j.taap.2008.02.001>

Lindberg, AL; Sohel, N; Rahman, M; Persson, LA; Vahter, M. (2010). Impact of smoking and chewing tobacco on arsenic-induced skin lesions. Environ Health Perspect 118: 533-538. <http://dx.doi.org/10.1289/ehp.0900728>

Lisabeth, LD; Ahn, HJ; Chen, JJ; Sealy-Jefferson, S; Burke, JF; Meliker, JR. (2010). Arsenic in drinking water and stroke hospitalizations in Michigan. Stroke 41: 2499-2504. <http://dx.doi.org/10.1161/STROKEAHA.110.585281>

Liu, FF; Wang, J, -P; Zheng, Y, -J; Ng, JC. (2013). Biomarkers for the evaluation of population health status 16 years after the intervention of arsenic-contaminated groundwater in Xinjiang, China. J Hazard Mater 262: 1159-1166. <http://dx.doi.org/10.1016/j.jhazmat.2013.03.058>

Lubin, JH; Fraumeni, JF, Jr. (2000). Re: "Does arsenic exposure increase the risk for circulatory disease?" [Letter]. Am J Epidemiol 152: 290-293. <http://dx.doi.org/10.1093/aje/152.3.290>

Lubin, JH; Moore, LE; Fraumeni, JF, Jr; Cantor, KP. (2008). Respiratory cancer and inhaled inorganic arsenic in copper smelters workers: A linear relationship with cumulative exposure that increases with concentration. Environ Health Perspect 116: 1661-1665. <http://dx.doi.org/10.1289/ehp.11515>

Lubin, JH; Pottern, LM; Blot, WJ; Tokudome, S; Stone, BJ; Fraumeni, JF, Jr. (1981). Respiratory cancer among copper smelter workers: Recent mortality statistics. J Occup Med 23: 779-784. <http://dx.doi.org/10.1097/00043764-198111000-00014>

Lundstrom, NG; Englyst, V; Gerhardsson, L; Jin, T; Nordberg, G. (2006). Lung cancer development in primary smelter workers: A nested case-referent study. J Occup Environ Med 48: 376-380. <http://dx.doi.org/10.1097/01.jom.0000201556.95982.95>

Luo, J; Hendryx, M; Ducatman, A. (2011). Association between six environmental chemicals and lung cancer incidence in the United States. J Environ Public Health 2011: 463701. <http://dx.doi.org/10.1155/2011/463701>

Maden, N; Singh, A; Smith, LS; Maharjan, M; Shrestha, S. (2011). Factors associated with arsenicosis and arsenic exposure status in Nepal: Implications from community based study. J Community Health 36: 76-82. <http://dx.doi.org/10.1007/s10900-010-9282-1>

Maharjan, M; Shrestha, RR; Ahmad, SA; Watanabe, C; Ohtsuka, R. (2006). Prevalence of arsenicosis in terai, Nepal. J Health Popul Nutr 24: 246-252.

Maharjan, M; Watanabe, C; Ahmad, SA; Ohtsuka, R. (2005). Arsenic contamination in drinking water and skin manifestations in lowland Nepal: The first community-based survey. Am J Trop Med Hyg 73: 477-479.

Maharjan, M; Watanabe, C; Ahmad, SA; Umezaki, M; Ohtsuka, R. (2007). Mutual interaction between nutritional status and chronic arsenic toxicity due to groundwater contamination in an area of Terai, lowland Nepal. J Epidemiol Community Health 61: 389-394. <http://dx.doi.org/10.1136/jech.2005.045062>

Maiti, S; Chattopadhyay, S; Deb, B; Samanta, T; Maji, G; Pan, B; Ghosh, A; Ghosh, D. (2012). Antioxidant and metabolic impairment result in DNA damage in arsenic-exposed individuals with severe dermatological manifestations in Eastern India. Environ Toxicol 27: 342-350. <http://dx.doi.org/10.1002/tox.20647>

Majumdar, KK; Ghose, A; Ghose, N; Biswas, A; Mazumder, DN. (2014). Effect of safe water on arsenicosis: A follow-up study. 3: 124-128. <http://dx.doi.org/10.4103/2249-4863.137626>

Majumdar, KK; Guha Mazumder, DN; Ghose, N; Ghose, A; Lahiri, S. (2009). Systemic manifestations in chronic arsenic toxicity in absence of skin lesions in West Bengal. Indian J Med Res 129: 75-82.

Makris, KC; Christophi, CA; Paisi, M; Ettinger, AS. (2012). A preliminary assessment of low level arsenic exposure and diabetes mellitus in Cyprus. BMC Public Health 12: 334. <http://dx.doi.org/10.1186/1471-2458-12-334>

Marsh, GM; Esmen, NA; Buchanich, JM; Youk, AO. (2009). Mortality patterns among workers exposed to arsenic, cadmium, and other substances in a copper smelter. Am J Ind Med 52: 633-644. <http://dx.doi.org/10.1002/ajim.20714>

Marshall, G; Ferreccio, C; Yuan, Y; Bates, MN; Steinmaus, C; Selvin, S; Liaw, J; Smith, AH. (2007). Fifty-year study of lung and bladder cancer mortality in Chile related to arsenic in drinking water. J Natl Cancer Inst 99: 920-928. <http://dx.doi.org/10.1093/jnci/djm004>

Mazumdar, S; Redmond, CK; Enterline, PE; Marsh, GM; Costantino, JP; Zhou, SYJ; Patwardhan, RN. (1989). Multistage modeling of lung cancer mortality among arsenic-exposed copper-smelter workers. Risk Anal 9: 551-563. <http://dx.doi.org/10.1111/j.1539-6924.1989.tb01266.x>

McCarty, KM; Houseman, EA; Quamruzzaman, Q; Rahman, M; Mahiuddin, G; Smith, T; Ryan, L; Christiani, DC. (2006). The impact of diet and betel nut use on skin lesions associated with drinking-water arsenic in Pabna, Bangladesh. Environ Health Perspect 114: 334-340. <http://dx.doi.org/10.1289/ehp.7916>

Mcdermott, S; Bao, W; Aelion, CM; Cai, B; Lawson, AB. (2014). Does the metal content in soil around a pregnant woman's home increase the risk of low birth weight for her infant? Environ Geochem Health 36: 1191-1197. <http://dx.doi.org/10.1007/s10653-014-9617-4>

McDonald, C; Hoque, R; Huda, N; Cherry, N. (2006). Prevalence of arsenic-related skin lesions in 53 widely-scattered villages of Bangladesh: An ecological survey. J Health Popul Nutr 24: 228-235.

McDonald, C; Hoque, R; Huda, N; Cherry, N. (2007). Risk of arsenic-related skin lesions in Bangladeshi villages at relatively low exposure: A report from Gonoshasthaya Kendra. Bull World Health Organ 85: 668-673. <http://dx.doi.org/10.2471/BLT.06.036764>

Mclaughlin, JK; Chen, JQ; Dosemici, M; Chen, RA; Rexing, SH; Wu, Z; Hearl, FJ; Mccawley, MA; Blot, WJ. (1992). A nested case-control study of lung cancer among silica exposed workers in China. Occup Environ Med 49: 167-171.

Medrano, MJ; Boix, R; Pastor-Barriuso, R; Palau, M; Damián, J; Ramis, R; del Barrio, JL; Navas-Acien, A. (2010). Arsenic in public water supplies and cardiovascular mortality in Spain. Environ Res 110: 448-454. <http://dx.doi.org/10.1016/j.envres.2009.10.002>

Meliker, J. R.; Slotnick, MJ; Avruskin, GA; Schottenfeld, D; Jacquez, GM; Wilson, ML; Goovaerts, P; Franzblau, A; Nriagu, JO. (2010). Lifetime exposure to arsenic in drinking water and bladder cancer: A population-based case-control study in Michigan, USA. Cancer Causes Control 21: 745-757. <http://dx.doi.org/10.1007/s10552-010-9503-z>

Meliker, JR; Wahl, RL; Cameron, LL; Nriagu, JO. (2007). Arsenic in drinking water and cerebrovascular disease, diabetes mellitus, and kidney disease in Michigan: A standardized mortality ratio analysis. Environ Health 6. <http://dx.doi.org/10.1186/1476-069X-6-4>

Melkonian, S; Argos, M; Pierce, BL; Chen, Y; Islam, T; Ahmed, A; Syed, EH; Parvez, F; Graziano, J; Rathouz, PJ; Ahsan, H. (2011). A prospective study of the synergistic effects of arsenic exposure and smoking, sun exposure, fertilizer use, and pesticide use on risk of premalignant skin lesions in Bangladeshi men. Am J Epidemiol 173: 183-191. <http://dx.doi.org/10.1093/aje/kwq357>

Michaud, DS; Wright, ME; Cantor, KP; Taylor, PR; Virtamo, J; Albanes, D. (2004). Arsenic concentrations in prediagnostic toenails and the risk of bladder cancer in a cohort study of male smokers. 160: 853-859. <http://dx.doi.org/10.1093/aje/kwh295>

Mikoczy, Z; Schutz, A; Stromberg, U; Hagmar, L. (1996). Cancer incidence and specific occupational exposures in the Swedish leather tanning industry: A cohort based case-control study. Occup Environ Med 53: 463-467.

Milton, AH; Hasan, Z; Rahman, A; Rahman, M. (2001). Chronic arsenic poisoning and respiratory effects in Bangladesh. J Occup Health 43: 136-140. <http://dx.doi.org/10.1539/joh.43.136>

Milton, AH; Rahman, M. (2002). Respiratory effects and arsenic contaminated well water in Bangladesh. Int J Environ Health Res 12: 175-179. <http://dx.doi.org/10.1080/09603120220129346>

Milton, AH; Smith, W; Rahman, B; Hasan, Z; Kulsum, U; Dear, K; Rakibuddin, M; Ali, A. (2005). Chronic arsenic exposure and adverse pregnancy outcomes in Bangladesh. Epidemiology 16: 82-86. <http://dx.doi.org/10.1097/01.ede.0000147105.94041.e6>

Mitra, AK; Bose, BK; Kabir, H; Das, BK; Hussain, M. (2002). Arsenic-related health problems among hospital patients in southern Bangladesh. J Health Popul Nutr 20: 198-204.

Moon, KA; Guallar, E; Umans, JG; Devereux, RB; Best, LG; Francesconi, KA; Goessler, W; Pollak, J; Silbergeld, EK; Howard, BV; Navas-Acien, A. (2013). Association between exposure to low to moderate arsenic levels and incident cardiovascular disease: A prospective cohort study. Ann Intern Med 159: 649-659. <http://dx.doi.org/10.7326/0003-4819-159-10-201311190-00719>

Moore, SE; Prentice, AM; Wagatsuma, Y; Fulford, AJC; Collinson, AC; Raqib, R; Vahter, M; Persson, LÅ; Arifeen, SE. (2009). Early-life nutritional and environmental determinants of thymic size in infants born in rural Bangladesh. Acta Paediatr 98: 1168-1175. <http://dx.doi.org/10.1111/j.1651-2227.2009.01292.x>

Morales, KH; Ryan, L; Kuo, T, -L; Wu, M, -M; Chen, C, -J. (2000). Risk of internal cancers from arsenic in drinking water. Environ Health Perspect 108: 655-661.

Mordukhovich, I; Wright, RO; Amarasiriwardena, C; Baja, E; Baccarelli, A; Suh, H; Sparrow, D; Vokonas, P; Schwartz, J. (2009). Association between low-level environmental arsenic exposure and QT interval duration in a general population study. Am J Epidemiol 170: 739-746. <http://dx.doi.org/10.1093/aje/kwp191>

Morton, W; Starr, G; Pohl, D; Stoner, J; Wagner, S; Weswig, P. (1976). Skin cancer and water arsenic in Lane County, Oregon. Cancer 37: 2523-2532. [http://dx.doi.org/10.1002/1097-0142(197605)37:5<2523::aid-cncr2820370545>3.0.co;2-b](http://dx.doi.org/10.1002/1097-0142(197605)37:5%3c2523::aid-cncr2820370545%3e3.0.co;2-b)

Mosaferi, M; Yunesian, M; Dastgiri, S; Mesdaghinia, A; Esmailnasab, N. (2008). Prevalence of skin lesions and exposure to arsenic in drinking water in Iran. Sci Total Environ 390: 69-76. <http://dx.doi.org/10.1016/j.scitotenv.2007.09.035>

Mostafa, MG; Cherry, N. (2013). Arsenic in drinking water and renal cancers in rural Bangladesh. Occup Environ Med 70: 768-773. <http://dx.doi.org/10.1136/oemed-2013-101443>

Mostafa, MG; McDonald, JC; Cherry, NM. (2008). Lung cancer and exposure to arsenic in rural Bangladesh. Occup Environ Med 65: 765-768. <http://dx.doi.org/10.1136/oem.2007.037895>

Mukherjee, SC; Saha, KC; Pati, S; Dutta, RN; Rahman, MM; Sengupta, MK; Ahamed, S; Lodh, D; Das, B; Hossain, MA; Nayak, B; Mukherjee, A; Chakraborti, D; Dulta, SK; Palit, SK; Kaies, I; Barua, AK; Asad, KA. (2005). Murshidabad--One of the nine groundwater arsenic-affected districts of West Bengal, India. Part II: Dermatological, neurological, and obstetric findings. J Toxicol Clin Toxicol 43: 835-848. <http://dx.doi.org/10.1080/15563650500357495>

Mumford, JL; Wu, K; Xia, Y; Kwok, R; Yang, Z; Foster, J; Sanders, WE, Jr. (2007). Chronic arsenic exposure and cardiac repolarization abnormalities with QT interval prolongation in a population-based study. Environ Health Perspect 115: 690-694. <http://dx.doi.org/10.1289/ehp.9686>

Myers, SL; Lobdell, DT; Liu, Z; Xia, Y; Ren, H; Li, Y; Kwok, RK; Mumford, JL; Mendola, P. (2010). Maternal drinking water arsenic exposure and perinatal outcomes in Inner Mongolia, China. J Epidemiol Community Health 64: 325-329. <http://dx.doi.org/10.1136/jech.2008.084392>

Nabi, AHM, N; Rahman, MM; Islam, LN. (2005). Evaluation of biochemical changes in chronic arsenic poisoning among Bangladeshi patients. Int J Environ Res Public Health 2: 385-393. <http://dx.doi.org/10.3390/ijerph2005030002>

Nadeau, KC; Li, Z; Farzan, S; Koestler, D; Robbins, D; Fei, DL; Malipatlolla, M; Maecker, H; Enelow, R; Korrick, S; Karagas, MR. (2014). In utero arsenic exposure and fetal immune repertoire in a US pregnancy cohort. Clin Immunol 155: 188-197. <http://dx.doi.org/10.1016/j.clim.2014.09.004>

Nafees, AA; Kazi, A; Fatmi, Z; Irfan, M; Ali, A; Kayama, F. (2011). Lung function decrement with arsenic exposure to drinking groundwater along River Indus: A comparative cross-sectional study. Environ Geochem Health 33: 203-216. <http://dx.doi.org/10.1007/s10653-010-9333-7>

Nakadaira, H; Endoh, K; Katagiri, M; Yamamoto, M. (2002). Elevated mortality from lung cancer associated with arsenic exposure for a limited duration. J Occup Environ Med 44: 291-299. <http://dx.doi.org/10.1097/00043764-200203000-00017>

Navas-Acien, A; Silbergeld, EK; Pastor-Barriuso, R; Guallar, E. (2008). Arsenic exposure and prevalence of type 2 diabetes in US adults. JAMA 300: 814-822. <http://dx.doi.org/10.1001/jama.300.7.814>

Navas-Acien, A; Silbergeld, EK; Pastor-Barriuso, R; Guallar, E. (2009). Rejoinder: Arsenic exposure and prevalence of type 2 diabetes: Updated findings from the National Health Nutrition and Examination Survey, 2003-2006 [Comment]. Epidemiology 20: 816-820. <http://dx.doi.org/10.1097/EDE.0b013e3181afef88>

Nizam, S; Kato, M; Yatsuya, H; Khalequzzaman, M; Ohnuma, S; Naito, H; Nakajima, T. (2013). Differences in urinary arsenic metabolites between diabetic and non-diabetic subjects in Bangladesh. Int J Environ Res Public Health 10: 1006-1019. <http://dx.doi.org/10.3390/ijerph10031006>

NRC (National Research Council). (2013). Critical aspects of EPA's IRIS assessment of inorganic arsenic: Interim report. Washington, D.C: The National Academies Press.

Olivas-Calderon, E; Recio-Vega, R; Gandolfi, A; Lantz, RC; Gonzalez-Cortes, T; Gonzalez-De Alba, C; Froines, J. R.; Espinosa-Fematt, JA. (2015). Lung inflammation biomarkers and lung function in children chronically exposed to arsenic. Toxicol Appl Pharmacol 287: 161-167. <http://dx.doi.org/10.1016/j.taap.2015.06.001>

Osorio-Yáñez, C; Ayllon-Vergara, JC; Aguilar-Madrid, G; Arreola-Mendoza, L; Hernández-Castellanos, E; Barrera-Hernández, A; De Vizcaya-Ruiz, A; Del Razo, LM. (2013). Carotid intima-media thickness and plasma asymmetric dimethylarginine in Mexican children exposed to inorganic arsenic. Environ Health Perspect 121: 1090-1096. <http://dx.doi.org/10.1289/ehp.1205994>

Osorio-Yáñez, C; Ayllon-Vergara, JC; Arreola-Mendoza, L; Aguilar-Madrid, G; Hernández-Castellanos, E; Sánchez-Peña, LC; Del Razo, LM. (2015). Blood pressure, left ventricular geometry, and systolic function in children exposed to inorganic arsenic. Environ Health Perspect 123: 629-635. <http://dx.doi.org/10.1289/ehp.1307327>

Pan, WC; Seow, WJ; Kile, ML; Hoffman, EB; Quamruzzaman, Q; Rahman, M; Mahiuddin, G; Mostofa, G; Lu, Q; Christiani, DC. (2013). Association of low to moderate levels of arsenic exposure with risk of type 2 diabetes in Bangladesh. Am J Epidemiol 178: 1563-1570. <http://dx.doi.org/10.1093/aje/kwt195>

Parvez, F; Chen, Y; Brandt-Rauf, PW; Bernard, A; Dumont, X; Slavkovich, V; Argos, M; D'Armiento, J; Foronjy, R; Hasan, MR; Eunus, HEM, M; Graziano, JH; Ahsan, H. (2008). Nonmalignant respiratory effects of chronic arsenic exposure from drinking water among never-smokers in Bangladesh. Environ Health Perspect 116: 190-195. <http://dx.doi.org/10.1289/ehp.9507>

Parvez, F; Chen, Y; Brandt-Rauf, PW; Slavkovich, V; Islam, T; Ahmed, A; Argos, M; Hassan, R; Yunus, M; Haque, SE; Balac, O; Graziano, JH; Ahsan, H. (2010). A prospective study of respiratory symptoms associated with chronic arsenic exposure in Bangladesh: Findings from the Health Effects of Arsenic Longitudinal Study (HEALS). Thorax 65: 528-533. <http://dx.doi.org/10.1136/thx.2009.119347>

Parvez, F; Chen, Y; Yunus, M; Olopade, C; Segers, S; Slavkovich, V; Argos, M; Hasan, R; Ahmed, A; Islam, T; Akter, MM; Graziano, JH; Ahsan, H. (2013). Arsenic exposure and impaired lung function. Findings from a large population-based prospective cohort study. Am J Respir Crit Care Med 188: 813-819. <http://dx.doi.org/10.1164/rccm.201212-2282OC>

Paul, S; Das, N; Bhattacharjee, P; Banerjee, M; Das, JK; Sarma, N; Sarkar, A; Bandyopadhyay, AK; Sau, TJ; Basu, S; Banerjee, S; Majumder, P; Giri, AK. (2013). Arsenic-induced toxicity and carcinogenicity: A two-wave cross-sectional study in arsenicosis individuals in West Bengal, India. J Expo Sci Environ Epidemiol 23: 156-162. <http://dx.doi.org/10.1038/jes.2012.91>

Pavittranon, S; Sripaoraya, K; Ramchuen, S; Kachamatch, S; Puttaprug, W; Pamornpusirikul, N; Thaicharuen, S; Rujiwanitchkul, S; Walueng, W. (2003). Laboratory case identification of arsenic in Ronpibul village, Thailand (2000-2002). J Environ Sci Health A Tox Hazard Subst Environ Eng 38: 213-221. <http://dx.doi.org/10.1081/ESE-120016890>

Pei, Q; Ma, N; Zhang, J; Xu, W; Li, Y; Ma, Z; Li, Y; Tian, F; Zhang, W; Mu, J; Li, Y; Wang, D; Liu, H; Yang, M; Ma, C; Yun, F. (2013). Oxidative DNA damage of peripheral blood polymorphonuclear leukocytes, selectively induced by chronic arsenic exposure, is associated with extent of arsenic-related skin lesions. Toxicol Appl Pharmacol 266: 143-149. <http://dx.doi.org/10.1016/j.taap.2012.10.031>

Peng, Q; Harlow, SD; Park, SK. (2015a). Urinary arsenic and insulin resistance in US adolescents. Int J Hyg Environ Health 218: 407-413. <http://dx.doi.org/10.1016/j.ijheh.2015.03.006>

Peng, S; Liu, L; Zhang, X; Heinrich, J; Zhang, J, ie; Schramm, KW; Huang, Q; Tian, M; Eqani, S; Shen, H. (2015b). A nested case-control study indicating heavy metal residues in meconium associate with maternal gestational diabetes mellitus risk. Environ Health 14. <http://dx.doi.org/10.1186/s12940-015-0004-0>

Perry, K; Bowler, RG; Buckell, HM; Druett, HA; Schilling, RSF. (1948). Studies in the incidence of cancer in a factory handling inorganic compounds of arsenic: II. Clinical and environmental investigations. Br J Ind Med 5: 6-15.

Pesch, B; Ranft, U; Jakubis, P; Nieuwenhuijsen, MJ; Hergemöller, A; Unfried, K; Jakubis, M; Miskovic, P; Keegan, T; Group, ES. (2002). Environmental arsenic exposure from a coal-burning power plant as a potential risk factor for nonmelanoma skin carcinoma: Results from a case-control study in the district of Prievidza, Slovakia. Am J Epidemiol 155: 798-809. <http://dx.doi.org/10.1093/aje/155.9.798>

Pesola, GR; Parvez, F; Chen, Y; Ahmed, A; Hasan, R; Ahsan, H. (2012). Arsenic exposure from drinking water and dyspnoea risk in Araihazar, Bangladesh: A population-based study. Eur Respir J 39: 1076-1083. <http://dx.doi.org/10.1183/09031936.00042611>

Philipp, R; Hughes, AO; Robertson, MC; Mitchell, TF. (1983). Malignant melanoma incidence and association with arsenic. Bristol Med Chir J 98: 165-169.

Pi, J; Yamauchi, H; Sun, G; Yoshida, T; Aikawa, H; Fujimoto, W; Iso, H; Cui, R; Waalkes, MP; Kumagai, Y. (2005). Vascular dysfunction in patients with chronic arsenosis can be reversed by reduction of arsenic exposure. Environ Health Perspect 113: 339-341. <http://dx.doi.org/10.1289/ehp.7471>

Pierce, BL; Argos, M; Chen, Y; Melkonian, S; Parvez, F; Islam, T; Ahmed, A; Hasan, R; Rathouz, PJ; Ahsan, H. (2011). Arsenic exposure, dietary patterns, and skin lesion risk in Bangladesh: A prospective study. Am J Epidemiol 173: 345-354. <http://dx.doi.org/10.1093/aje/kwq366>

Pinto, SS; Henderson, V; Enterline, PE. (1978). Mortality experience of arsenic-exposed workers. Arch Environ Health 33: 325-331. <http://dx.doi.org/10.1080/00039896.1978.10667356>

Pou, SA; Osella, AR; Diaz, M, del P. (2011). Bladder cancer mortality trends and patterns in Córdoba, Argentina (19862006). Cancer Causes Control 22: 407-415. <http://dx.doi.org/10.1007/s10552-010-9711-6>

Pu, Y, -S; Yang, S, -M; Huang, Y, -K; Chung, C, -J; Huang, SK; Chiu, AW, -H; Yang, M, -H; Chen, C, -J; Hsueh, Y, -M. (2007). Urinary arsenic profile affects the risk of urothelial carcinoma even at low arsenic exposure. Toxicol Appl Pharmacol 218: 99-106. <http://dx.doi.org/10.1016/j.taap.2006.09.021>

Qiao, YL; Taylor, PR; Yao, SX; Erozan, YS; Luo, XC; Barrett, MJ; Yan, QY; Giffen, CA; Huang, SQ; Maher, MM; Forman, MR; Tockman, MS. (1997). Risk factors and early detection of lung cancer in a cohort of Chinese tin miners. Ann Epidemiol 7: 533-541.

Rahman, A; Persson, L, -Å; Nermell, B; El Arifeen, S; Ekström, E, -C; Smith, AH; Vahter, M. (2010). Arsenic exposure and risk of spontaneous abortion, stillbirth, and infant mortality. Epidemiology 21: 797-804. <http://dx.doi.org/10.1097/EDE.0b013e3181f56a0d>

Rahman, A; Vahter, M; Ekström, E, -C; Persson, L, -Å. (2011). Arsenic exposure in pregnancy increases the risk of lower respiratory tract infection and diarrhea during infancy in Bangladesh. Environ Health Perspect 119: 719-724. <http://dx.doi.org/10.1289/ehp.1002265>

Rahman, A; Vahter, M; Ekström, E, -C; Rahman, M; Golam Mustafa, AHM; Wahed, MA; Yunus, M; Persson, L, -Å. (2007). Association of arsenic exposure during pregnancy with fetal loss and infant death: A cohort study in Bangladesh. Am J Epidemiol 165: 1389-1396. <http://dx.doi.org/10.1093/aje/kwm025>

Rahman, A; Vahter, M; Smith, AH; Nermell, B; Yunus, M; El Arifeen, S; Persson, L, -Å; Ekström, E, -C. (2009). Arsenic exposure during pregnancy and size at birth: A prospective cohort study in Bangladesh. Am J Epidemiol 169: 304-312. <http://dx.doi.org/10.1093/aje/kwn332>

Rahman, M; Axelson, O. (1995). Diabetes mellitus and arsenic exposure: A second look at case-control data from a Swedish copper smelter. Occup Environ Med 52: 773-774. <http://dx.doi.org/10.1136/oem.52.11.773>

Rahman, M; Axelson, O. (2001). Arsenic ingestion and health effects in Bangladesh: Epidemiological observations. In WR Chappell; CO Abernathy; RL Calderon (Eds.), Arsenc Exposure and Health Effects IV (1 ed., pp. 193-199). Amsterdam, The Netherlands: Elsevier Science. <https://books.google.com/books?id=kpT2eIxOCi8C&printsec=frontcover&source=gbs_ge_summary_r&cad=0#v=onepage&q&f=false>

Rahman, M; Sohel, N; Yunus, M; Chowdhury, ME; Hore, SK; Zaman, K; Bhuiya, A; Streatfield, PK. (2014). A prospective cohort study of stroke mortality and arsenic in drinking water in Bangladeshi adults. BMC Public Health 14: 174. <http://dx.doi.org/10.1186/1471-2458-14-174>

Rahman, M; Tondel, M; Ahmad, SA; Axelson, O. (1998). Diabetes mellitus associated with arsenic exposure in Bangladesh. Am J Epidemiol 148: 198-203.

Rahman, M; Tondel, M; Ahmad, SA; Chowdhury, IA; Faruquee, MH; Axelson, O. (1999a). Hypertension and arsenic exposure in Bangladesh. Hypertension 33: 74-78. <http://dx.doi.org/10.1161/01.HYP.33.1.74>

Rahman, M; Tondel, M; Chowdhury, IA; Axelson, O. (1999b). Relations between exposure to arsenic, skin lesions, and glucosuria. Occup Environ Med 56: 277-281. <http://dx.doi.org/10.1136/oem.56.4.277>

Rahman, M; Vahter, M; Wahed, MA; Sohel, N; Yunus, M; Streatfield, PK; El Arifeen, S; Bhuiya, A; Zaman, K; Chowdhury, AMR; Ekström, E, -C; Persson, LÅ. (2006). Prevalence of arsenic exposure and skin lesions. A population based survey in Matlab, Bangladesh. J Epidemiol Community Health 60: 242-248. <http://dx.doi.org/10.1136/jech.2005.040212>

Rahman, M; Wingren, G; Axelson, O. (1996). Diabetes mellitus among Swedish art glass workers -- An effect of arsenic exposure? Scand J Work Environ Health 22: 146-149. <http://dx.doi.org/10.5271/sjweh.123>

Rahman, MM; Mandal, BK; Chowdhury, TR; Sengupta, MK; Chowdhury, UK; Lodh, D; Chanda, CR; Basu, GK; Mukherjee, SC; Saha, KC; Chakraborti, D. (2003). Arsenic groundwater contamination and sufferings of people in North 24-Parganas, one of the nine arsenic affected districts of West Bengal, India. J Environ Sci Health A Tox Hazard Subst Environ Eng 38: 25-59. <http://dx.doi.org/10.1081/ESE-120016658>

Rahman, MM; Sengupta, MK; Ahamed, S; Chowdhury, UK; Hossain, MA; Das, B; Lodh, D; Saha, KC; Pati, S; Kaies, I; Barua, AK; Chakraborti, D. (2005a). The magnitude of arsenic contamination in groundwater and its health effects to the inhabitants of the Jalangi--One of the 85 arsenic affected blocks in West Bengal, India. Sci Total Environ 338: 189-200. <http://dx.doi.org/10.1016/j.scitotenv.2004.06.022>

Rahman, MM; Sengupta, MK; Ahamed, S; Chowdhury, UK; Lodh, D; Hossain, A; Das, B; Roy, N; Saha, KC; Palit, SK; Chakraborti, D. (2005b). Arsenic contamination of groundwater and its health impact on residents in a village in West Bengal, India. Bull World Health Organ 83: 49-57.

Rahman, MM; Sengupta, MK; Ahamed, S; Chowdhury, UK; Lodh, D; Hossain, MA; Das, B; Saha, KC; Kaies, I; Barua, AK; Chakraborti, D. (2005c). Status of groundwater arsenic contamination and human suffering in a Gram Panchayet (cluster of villages) in Murshidabad, one of the nine arsenic affected districts in West Bengal, India. J Water Health 3: 283-296. <http://dx.doi.org/0.2166/wh.2005.038>

Raqib, R; Ahmed, S; Sultana, R; Wagatsuma, Y; Mondal, D; Hoque, AMW; Nermell, B; Yunus, M; Roy, S; Persson, LA; El Arifeen, S; Moore, S; Vahter, M. (2009). Effects of in utero arsenic exposure on child immunity and morbidity in rural Bangladesh. Toxicol Lett 185: 197-202. <http://dx.doi.org/10.1016/j.toxlet.2009.01.001>

Recio-Vega, R; Gonzalez-Cortes, T; Olivas-Calderon, E; Lantz, RC; Gandolfi, AJ; Alba, CG. (2015). In utero and early childhood exposure to arsenic decreases lung function in children. J Appl Toxicol 35: 358-366. <http://dx.doi.org/10.1002/jat.3023>

Rhee, SY; Hwang, YC; Woo, JT; Chin, SO; Chon, S; Kim, YS. (2013). Arsenic exposure and prevalence of diabetes mellitus in Korean adults. J Korean Med Sci 28: 861-868. <http://dx.doi.org/10.3346/jkms.2013.28.6.861>

Rivara, MI; Cebrián, M; Corey, G; Hernández, M; Romieu, I. (1997). Cancer risk in an arsenic-contaminated area of Chile. Toxicol Ind Health 13: 321-338. <http://dx.doi.org/10.1177/074823379701300217>

Rosales-Castillo, JA; Acosta-Saavedra, LC; Torres, R; Ochoa-Fierro, J; Borja-Aburto, VH; Lopez-Carrillo, L; Garcia-Vargas, GG; Gurrola, GB; Cebrian, ME; Calderón-Aranda, ES. (2004). Arsenic exposure and human papillomavirus response in non-melanoma skin cancer Mexican patients: A pilot study. Int Arch Occup Environ Health 77: 418-423. <http://dx.doi.org/10.1007/s00420-004-0527-0>

Saha, A; Chowdhury, MI; Nazim, M; Alam, MM; Ahmed, T; Hossain, MB; Hore, SK; Sultana, GNN; Svennerholm, A, -M; Qadri, F. (2013). Vaccine specific immune response to an inactivated oral cholera vaccine and EPI vaccines in a high and low arsenic area in Bangladeshi children. Vaccine 31: 647-652. <http://dx.doi.org/10.1016/j.vaccine.2012.11.049>

Saha, KC; Poddar, D. (1986). Further studies on chronic arsenical dermatosis. Indian J Dermatol 31: 29-33.

Saha, KK; Engström, A; Hamadani, JD; Tofail, F; Rasmussen, KM; Vahter, M. (2012). Pre- and postnatal arsenic exposure and body size to two years of age: A cohort study in rural Bangladesh. Environ Health Perspect 120: 1208-1214. <http://dx.doi.org/10.1289/ehp.1003378>

Sawada, N; Iwasaki, M; Inoue, M; Takachi, R; Sasazuki, S; Yamaji, T; Shimazu, T; Tsugane, S. (2013). Dietary arsenic intake and subsequent risk of cancer: The Japan Public Health Center-based (JPHC) prospective study. Cancer Causes Control 24: 1403-1415. <http://dx.doi.org/10.1007/s10552-013-0220-2>

Schäfer, T; Heinrich, J; Wjst, M; Krause, C; Adam, H; Ring, J; Wichmann, HE. (1999). Indoor risk factors for atopic eczema in school children from East Germany. Environ Res 81: 151-158. <http://dx.doi.org/10.1006/enrs.1999.3964>

Sen, J; Chaudhuri, ABD. (2008). Arsenic exposure through drinking water and its effect on pregnancy outcome in Bengali women. Arh Hig Rada Toksikol 59: 271-275. <http://dx.doi.org/10.2478/10004-1254-59-2008-1871>

Seow, WJ; Pan, W, -C; Kile, ML; Baccarelli, AA; Quamruzzaman, Q; Rahman, M; Mahiuddin, G; Mostofa, G; Lin, X; Christiani, DC. (2012). Arsenic reduction in drinking water and improvement in skin lesions: A follow-up study in Bangladesh. Environ Health Perspect 120: 1733-1738. <http://dx.doi.org/10.1289/ehp.1205381>

Ser, PH; Banu, B; Jebunnesa, F; Fatema, K; Rosy, N; Yasmin, R; Furusawa, H; Ali, L; Ahmad, SA; Watanabe, C. (2014). Arsenic exposure increases maternal but not cord serum immunoglobulin G level in Bangladesh. Pediatrics International 57: 119-125. <http://dx.doi.org/10.1111/ped.12396>

Shapiro, GD; Dodds, L; Arbuckle, TE; Ashley-Martin, J; Fraser, W; Fisher, M; Taback, S; Keely, E; Bouchard, MF; Monnier, P; Dallaire, R; Morisset, AS; Ettinger, AS. (2015). Exposure to phthalates, bisphenol A and metals in pregnancy and the association with impaired glucose tolerance and gestational diabetes mellitus: The MIREC study. Environ Int 83: 63-71. <http://dx.doi.org/10.1016/j.envint.2015.05.016>

Shiue, I. (2013). Association of urinary arsenic, heavy metal, and phthalate concentrations with food allergy in adults: National Health and Nutrition Examination Survey, 2005-2006 [Letter]. Ann Allergy Asthma Immunol 111: 421-423. <http://dx.doi.org/10.1016/j.anai.2013.08.006>

Skröder, H; Hawkesworth, S; Kippler, M; El Arifeen, S; Wagatsuma, Y; Moore, SE; Vahter, M. (2015). Kidney function and blood pressure in preschool-aged children exposed to cadmium and arsenic - potential alleviation by selenium. Environ Res 140: 205-213. <http://dx.doi.org/10.1016/j.envres.2015.03.038>

Smith, AH; Arroyo, AP; Mazumder, DN; Kosnett, MJ; Hernandez, AL; Beeris, M; Smith, MM; Moore, LE. (2000). Arsenic-induced skin lesions among Atacameño people in Northern Chile despite good nutrition and centuries of exposure. Environ Health Perspect 108: 617-620.

Smith, AH; Goycolea, M; Haque, R; Biggs, ML. (1998). Marked increase in bladder and lung cancer mortality in a region of Northern Chile due to arsenic in drinking water. Am J Epidemiol 147: 660-669. <http://dx.doi.org/10.1093/oxfordjournals.aje.a009507>

Smith, AH; Marshall, G; Liaw, J; Yuan, Y; Ferreccio, C; Steinmaus, C. (2012). Mortality in young adults following in utero and childhood exposure to arsenic in drinking water. Environ Health Perspect 120: 1527-1531. <http://dx.doi.org/10.1289/ehp.1104867>

Smith, AH; Marshall, G; Yuan, Y; Ferreccio, C; Liaw, J; von Ehrenstein, O; Steinmaus, C; Bates, MN; Selvin, S. (2006). Increased mortality from lung cancer and bronchiectasis in young adults after exposure to arsenic in utero and in early childhood. Environ Health Perspect 114: 1293-1296. <http://dx.doi.org/10.1289/ehp.8832>

Smith, AH; Marshall, G; Yuan, Y; Liaw, J; Ferreccio, C; Steinmaus, C. (2011). Evidence from Chile that arsenic in drinking water may increase mortality from pulmonary tuberculosis. Am J Epidemiol 173: 414-420. <http://dx.doi.org/10.1093/aje/kwq383>

Smith, AH; Yunus, M; Khan, AF; Ercumen, A; Yuan, Y; Smith, MH; Liaw, J; Balmes, J; von Ehrenstein, O; Raqib, R; Kalman, D; Alam, DS; Streatfield, PK; Steinmaus, C. (2013). Chronic respiratory symptoms in children following in utero and early life exposure to arsenic in drinking water in Bangladesh. Int J Epidemiol 42: 1077-1086. <http://dx.doi.org/10.1093/ije/dyt120>

Sohel, N; Persson, LÅ; Rahman, M; Streatfield, PK; Yunus, M; Ekström, E, -C; Vahter, M. (2009). Arsenic in drinking water and adult mortality: A population-based cohort study in rural Bangladesh. Epidemiology 20: 824-830. <http://dx.doi.org/10.1097/EDE.0b013e3181bb56ec>

Sorahan, T. (2009). Lung cancer mortality in arsenic-exposed workers from a cadmium recovery plant. Occup Med (Lond) 59: 264-266. <http://dx.doi.org/10.1093/occmed/kqp046>

Soto-Peña, GA; Luna, AL; Acosta-Saavedra, L; Conde-Moo, P; López-Carrillo, L; Cebrián, ME; Bastida, M; Calderón-Aranda, ES; Vega, L. (2006). Assessment of lymphocyte subpopulations and cytokine secretion in children exposed to arsenic. FASEB J 20: 779-781. <http://dx.doi.org/10.1096/fj.05-4860fje>

Steinmaus, C; Ferreccio, C; Acevedo, J; Yuan, Y; Liaw, J; Durán, V; Cuevas, S; García, J; Meza, R; Valdés, R; Valdés, G; Benítez, H; Vanderlinde, V; Villagra, V; Cantor, KP; Moore, LE; Perez, SG; Steinmaus, S; Smith, AH. (2014a). Increased lung and bladder cancer incidence in adults after in utero and early-life arsenic exposure. Cancer Epidemiol Biomarkers Prev 23: 1529-1538. <http://dx.doi.org/10.1158/1055-9965.EPI-14-0059>

Steinmaus, C; Ferreccio, C; Yuan, Y; Acevedo, J; González, F; Perez, L; Cortés, S; Balmes, JR; Liaw, J; Smith, AH. (2014b). Elevated lung cancer in younger adults and low concentrations of arsenic in water. Am J Epidemiol 180: 1082-1087. <http://dx.doi.org/10.1093/aje/kwu238>

Steinmaus, C; Yuan, Y; Bates, MN; Smith, AH. (2003). Case-control study of bladder cancer and drinking water arsenic in the western United States. Am J Epidemiol 158: 1193-1201. <http://dx.doi.org/10.1093/aje/kwg281>

Steinmaus, C; Yuan, Y; Liaw, J; Smith, AH. (2009). Low-level population exposure to inorganic arsenic in the United States and diabetes mellitus: A reanalysis. Epidemiology 20: 807-815. <http://dx.doi.org/10.1097/EDE.0b013e3181b0fd29>

Steinmaus, CM; Ferreccio, C; Romo, JA; Yuan, Y; Cortes, S; Marshall, G; Moore, LE; Balmes, JR; Liaw, J; Golden, T; Smith, AH. (2013). Drinking water arsenic in northern Chile: High cancer risks 40 years after exposure cessation. Cancer Epidemiol Biomarkers Prev 22: 623-630. <http://dx.doi.org/10.1158/1055-9965.EPI-12-1190>

Stocks, P. (1960). On the relations between atmospheric pollution in urban and rural localities and mortality from cancer, bronchitis and pneumonia, with particular reference to 3:4 benzopyrene, beryllium, molybdenum, vanadium and arsenic. Br J Cancer 14: 397-418. <http://dx.doi.org/10.1038/bjc.1960.45>

Su, CC; Lu, JL; Tsai, KY; Lian, IB. (2011). Reduction in arsenic intake from water has different impacts on lung cancer and bladder cancer in an arseniasis endemic area in Taiwan. Cancer Causes Control 22: 101-108. <http://dx.doi.org/10.1007/s10552-010-9679-2>

Surdu, S; Fitzgerald, EF; Bloom, MS; Boscoe, FP; Carpenter, DO; Haase, RF; Gurzau, E; Rudnai, P; Koppova, K; Févotte, J; Vahter, M; Leonardi, G; Goessler, W; Kumar, R; Fletcher, T. (2013). Occupational exposure to arsenic and risk of non-melanoma skin cancer in a multinational European study. Int J Cancer 133: 2182-2191. <http://dx.doi.org/10.1002/ijc.28216>

Taeger, D; Johnen, G; Wiethege, T; Tapio, S; Mohner, M; Wesch, H; Tannapfel, A; Muller, KM; Bruning, T; Pesch, B. (2009). Major histopathological patterns of lung cancer related to arsenic exposure in German uranium miners. Int Arch Occup Environ Health 82: 867-875. <http://dx.doi.org/10.1007/s00420-008-0386-1>

Taylor, PR; Qiao, Y, -L; Schatzkin, A; Yao, S, -X; Lubin, J; Mao, B, -L; Rao, J, -Y; McAdams, M; Xuan, X, -Z; Li, J, -Y. (1989). Relation of arsenic exposure to lung cancer among tin miners in Yunnan Province, China. Br J Ind Med 46: 881-886. <http://dx.doi.org/10.1136/oem.46.12.881>

Thomas, S; Arbuckle, TE; Fisher, M; Fraser, WD; Ettinger, A; King, W. (2015). Metals exposure and risk of small-for-gestational age birth in a Canadian birth cohort: The MIREC study. Environ Res 140: 430-439. <http://dx.doi.org/10.1016/j.envres.2015.04.018>

Tondel, M; Rahman, M; Magnuson, A; Chowdhury, IA; Faruquee, MH; Ahmad, SA. (1999). The relationship of arsenic levels in drinking water and the prevalence rate of skin lesions in Bangladesh. Environ Health Perspect 107: 727-729.

Tsai, SM; Wang, TN; Ko, YC. (1999). Mortality for certain diseases in areas with high levels of arsenic in drinking water. Arch Environ Health 54: 186-193. <http://dx.doi.org/10.1080/00039899909602258>

Tseng, C, -H; Chong, C, -K; Chen, C, -J; Tai, T, -Y. (1996). Dose-response relationship between peripheral vascular disease and ingested inorganic arsenic among residents in Blackfoot disease endemic villages in Taiwan. Atherosclerosis 120: 125-133. <http://dx.doi.org/10.1016/0021-9150(95)05693-9>

Tseng, C, -H; Chong, C, -K; Chen, C, -J; Tai, T, -Y. (1997). Lipid profile and peripheral vascular disease in arseniasis-hyperendemic villages in Taiwan. Angiology 48: 321-335. <http://dx.doi.org/10.1177/000331979704800405>

Tseng, C, -H; Chong, C, -K; Tseng, C, -P; Hsueh, Y, -M; Chiou, H, -Y; Tseng, C, -C; Chen, C, -J. (2003). Long-term arsenic exposure and ischemic heart disease in arseniasis-hyperendemic villages in Taiwan. Toxicol Lett 137: 15-21. <http://dx.doi.org/10.1016/S0378-4274(02)00377-6>

Tseng, C, -H; Huang, Y, -K; Huang, Y, -L; Chung, C, -J; Yang, M, -H; Chen, C, -J; Hsueh, Y, -M. (2005). Arsenic exposure, urinary arsenic speciation, and peripheral vascular disease in Blackfoot disease-hyperendemic villages in Taiwan. Toxicol Appl Pharmacol 206: 299-308. <http://dx.doi.org/10.1016/j.taap.2004.11.022>

Tseng, C, -H; Tai, T, -Y; Chong, C, -K; Tseng, C, -P; Lai, M, -S; Lin, BJ; Chiou, H, -Y; Hsueh, Y, -M; Hsu, K, -H; Chen, C, -J. (2000). Long-term arsenic exposure and incidence of non-insulin-dependent diabetes mellitus: A cohort study in arseniasis-hyperendemic villages in Taiwan. Environ Health Perspect 108: 847-851. <http://dx.doi.org/10.1289/ehp.00108847>

Tseng, WP. (1977). Effects and dose-response relationships of skin cancer and Blackfoot disease with arsenic. Environ Health Perspect 19: 109-119.

Tsuda, T; Babazono, A; Yamamoto, E; Kurumatani, N; Mino, Y; Ogawa, T; Kishi, Y; Aoyama, H. (1995). Ingested arsenic and internal cancer: A historical cohort study followed for 33 years. Am J Epidemiol 141: 198-209. <http://dx.doi.org/10.1016/S0190-9622(96)90452-1>

Valentine, JL; Bennett, RG; Borok, ME; Faraji, B. (1991). Environmental arsenic and skin toxicity. In B Molčilović (Ed.), Trace Elements in Man and Animals 7 (pp. 383-384). Zagreb, Yugoslavia: Institute for Medical Research and Occupational Health, University of Zagreb.

Valentine, JL; He, S, -Y; Reisbord, LS; Lachenbruch, PA. (1992). Health response by questionnaire in arsenic-exposed populations. J Clin Epidemiol 45: 487-494. <http://dx.doi.org/10.1016/0895-4356(92)90097-7>

Vall, O; Gómez-Culebras, M; Garcia-Algar, O; Joya, X; Velez, D; Rodríguez-Carrasco, E; Puig, C. (2012). Assessment of prenatal exposure to arsenic in Tenerife Island. PLoS ONE 7: e50463. <http://dx.doi.org/10.1371/journal.pone.0050463>

Varsányi, I; Fodré, Z; Bartha, A. (1991). Arsenic in drinking water and mortality in the Southern Great Plain, Hungary. Environ Geochem Health 13: 14-22. <http://dx.doi.org/10.1007/BF01783491>

von Ehrenstein, OS; Guha Mazumder, DN; Hira-Smith, M; Ghosh, N; Yuan, Y; Windham, G; Ghosh, A; Haque, R; Lahiri, S; Kalman, D; Das, S; Smith, AH. (2006). Pregnancy outcomes, infant mortality, and arsenic in drinking water in West Bengal, India. Am J Epidemiol 163: 662-669. <http://dx.doi.org/10.1093/aje/kwj089>

von Ehrenstein, OS; Guha Mazumder, DN; Yuan, Y; Samanta, S; Balmes, J; Sil, A; Ghosh, N; Hira-Smith, M; Haque, R; Purushothamam, R; Lahiri, S; Das, S; Smith, AH. (2005). Decrements in lung function related to arsenic in drinking water in West Bengal, India. Am J Epidemiol 162: 533-541. <http://dx.doi.org/10.1093/aje/kwi236>

Wade, TJ; Xia, Y; Mumford, J; Wu, K; Le, XC; Sams, E; Sanders, WE. (2015). Cardiovascular disease and arsenic exposure in Inner Mongolia, China: a case control study. Environ Health 14: 35. <http://dx.doi.org/10.1186/s12940-015-0022-y>

Wade, TJ; Xia, Y; Wu, K; Li, Y; Ning, Z; Le, XC; Lu, X; Feng, Y; He, X; Mumford, JL. (2009). Increased mortality associated with well-water arsenic exposure in Inner Mongolia, China. Int J Environ Res Public Health 6: 1107-1123. <http://dx.doi.org/10.3390/ijerph6031107>

Wadhwa, SK; Kazi, TG; Chandio, AA; Afridi, HI; Kolachi, NF; Khan, S; Kandhro, GA; Nasreen, S; Shah, AQ; Baig, JA. (2011a). Comparative study of liver cancer patients in arsenic exposed and non-exposed areas of Pakistan. Biol Trace Elem Res 144: 86-96. <http://dx.doi.org/10.1007/s12011-011-9036-7>

Wadhwa, SK; Kazi, TG; Kolachi, NF; Afridi, HI; Khan, S; Chandio, AA; Shah, AQ; Kandhro, GA; Nasreen, S. (2011b). Case-control study of male cancer patients exposed to arsenic-contaminated drinking water and tobacco smoke with relation to non-exposed cancer patients. Hum Exp Toxicol 30: 2013-2022. <http://dx.doi.org/10.1177/0960327111408154>

Wang, C, -H; Chen, C, -L; Hsiao, CK; Chiang, F, -T; Hsu, L, -I; Chiou, H, -Y; Hsueh, Y, -M; Wu, M, -M; Chen, C, -J. (2009a). Increased risk of QT prolongation associated with atherosclerotic diseases in arseniasis-endemic area in southwestern coast of Taiwan. Toxicol Appl Pharmacol 239: 320-324. <http://dx.doi.org/10.1016/j.taap.2009.06.017>

Wang, C, -H; Chen, C, -L; Hsiao, CK; Chiang, F, -T; Hsu, L, -I; Chiou, H, -Y; Hsueh, Y, -M; Wu, M, -M; Chen, C, -J. (2010). Arsenic-induced QT dispersion is associated with atherosclerotic diseases and predicts long-term cardiovascular mortality in subjects with previous exposure to arsenic: A 17-Year follow-up study. Cardiovasc Toxicol 10: 17-26. <http://dx.doi.org/10.1007/s12012-009-9059-x>

Wang, C, -H; Jeng, J, -S; Yip, P, -K; Chen, C, -L; Hsu, L, -I; Hsueh, Y, -M; Chiou, H, -Y; Wu, M, -M; Chen, C, -J. (2002). Biological gradient between long-term arsenic exposure and carotid atherosclerosis. Circulation 105: 1804-1809. <http://dx.doi.org/10.1161/01.CIR.0000015862.64816.B2>

Wang, JP; Wang, SL; Lin, Q; Zhang, L; Huang, D; Ng, JC. (2009b). Association of arsenic and kidney dysfunction in people with diabetes and validation of its effects in rats. Environ Int 35: 507-511. <http://dx.doi.org/10.1016/j.envint.2008.07.015>

Wang, S, -L; Chiou, J, -M; Chen, C, -J; Tseng, C, -H; Chou, W, -L; Wang, C, -C; Wu, T, -N; Chang, LW. (2003). Prevalence of non-insulin-dependent diabetes mellitus and related vascular diseases in southwestern arseniasis-endemic and nonendemic areas in Taiwan. Environ Health Perspect 111: 155-159. <http://dx.doi.org/10.1289/ehp.5457>

Wang, SL; Li, WF; Chen, CJ; Huang, YL; Chen, JW; Chang, KH; Tsai, LY; Chou, KM. (2011). Hypertension incidence after tap-water implementation: A 13-year follow-up study in the arseniasis-endemic area of southwestern Taiwan. Sci Total Environ 409: 4528-4535. <http://dx.doi.org/10.1016/j.scitotenv.2011.07.058>

Wang, Y, -H; Wu, M, -M; Hong, C, -T; Lien, L, -M; Hsieh, Y, -C; Tseng, H, -P; Chang, S, -F; Su, C, -L; Chiou, H, -Y; Chen, C, -J. (2007). Effects of arsenic exposure and genetic polymorphisms of p53, glutathione S-transferase M1, T1, and P1 on the risk of carotid atherosclerosis in Taiwan. Atherosclerosis 192: 305-312. <http://dx.doi.org/10.1016/j.atherosclerosis.2006.07.029>

Wang, YH; Yeh, SD; Shen, KH; Shen, CH; Juang, GD; Hsu, LI; Chiou, HY; Chen, CJ. (2009c). A significantly joint effect between arsenic and occupational exposures and risk genotypes/diplotypes of CYP2E1, GSTO1 and GSTO2 on risk of urothelial carcinoma. Toxicol Appl Pharmacol 241: 111-118. <http://dx.doi.org/10.1016/j.taap.2009.08.008>

Wasserman, GA; Liu, X; Parvez, F; Ahsan, H; Factor-Litvak, P; van Geen, A; Slavkovich, V; Lolacono, NJ; Cheng, Z; Hussain, I; Momotaj, H; Graziano, JH. (2004). Water arsenic exposure and children's intellectual function in Araihazar, Bangladesh. Environ Health Perspect 112: 1329-1333. <http://dx.doi.org/10.1289/ehp.6964>

Welch, K; Higgins, I; Oh, M; Burchfiel, C. (1982). Arsenic exposure, smoking, and respiratory cancer in copper smelter workers. Arch Environ Occup Health 37: 325-335. <http://dx.doi.org/10.1080/00039896.1982.10667586>

Wheeler, BW; Kothencz, G; Pollard, AS. (2013). Geography of non-melanoma skin cancer and ecological associations with environmental risk factors in England. Br J Cancer 109: 235-241. <http://dx.doi.org/10.1038/bjc.2013.288>

Wu, C, -C; Huang, Y, -K; Chung, C, -J; Huang, C, -Y; Pu, Y, -S; Shiue, H, -S; Lai, L, -A; Lin, Y, -C; Su, C, -T; Hsueh, Y, -M. (2013). Polymorphism of inflammatory genes and arsenic methylation capacity are associated with urothelial carcinoma. Toxicol Appl Pharmacol 272: 30-36. <http://dx.doi.org/10.1016/j.taap.2013.05.019>

Wu, C, -C; Su, C, -T; Lee, H, -L; Chung, C, -J; Huang, C, -Y; Pu, Y, -S; Lin, P; Hsueh, Y, -M. (2012a). Joint effect of arsenic methylation profile and NNK metabolites on urothelial carcinoma. J Urol 188: 1701-1705. <http://dx.doi.org/10.1016/j.juro.2012.07.025>

Wu, F; Jasmine, F; Kibriya, MG; Liu, M; Wójcik, O; Parvez, F; Rahaman, R; Roy, S; Paul-Brutus, R; Segers, S; Slavkovich, V; Islam, T; Levy, D; Mey, JL; van Geen, A; Graziano, JH; Ahsan, H; Chen, Y. (2012b). Association between arsenic exposure from drinking water and plasma levels of cardiovascular markers. Am J Epidemiol 175: 1252-1261. <http://dx.doi.org/10.1093/aje/kwr464>

Wu, M, -M; Chiou, H, -Y; Hsueh, Y, -M; Hong, C, -T; Su, C, -L; Chang, S, -F; Huang, W, -L; Wang, H, -T; Wang, Y, -H; Hsieh, Y, -C; Chen, C, -J. (2006). Effect of plasma homocysteine level and urinary monomethylarsonic acid on the risk of arsenic-associated carotid atherosclerosis. Toxicol Appl Pharmacol 216: 168-175. <http://dx.doi.org/10.1016/j.taap.2006.05.005>

Wu, M, -M; Kuo, T, -L; Hwang, Y, -H; Chen, C, -J. (1989). Dose-response relation between arsenic concentration in well water and mortality from cancers and vascular diseases. Am J Epidemiol 130: 1123-1132. <http://dx.doi.org/10.1161/01.STR.28.9.1717>

Wu, MM; Chiou, HY; Lee, TC; Chen, CL; Hsu, LI; Wang, YH; Huang, WL; Hsieh, YC; Yang, TY; Lee, CY; Yip, PK; Wang, CH; Hsueh, YM; Chen, CJ. (2010). GT-repeat polymorphism in the heme oxygenase-1 gene promoter and the risk of carotid atherosclerosis related to arsenic exposure. J Biomed Sci 17: 70. <http://dx.doi.org/10.1186/1423-0127-17-70>

Xia, Y; Wade, TJ; Wu, K; Li, Y; Ning, Z; Le, XC; He, X; Chen, B; Feng, Y; Mumford, JL. (2009). Well water arsenic exposure, arsenic induced skin-lesions and self-reported morbidity in Inner Mongolia. Int J Environ Res Public Health 6: 1010-1025. <http://dx.doi.org/10.3390/ijerph6031010>

Yang, C, -Y. (2006). Does arsenic exposure increase the risk of development of peripheral vascular diseases in humans? J Toxicol Environ Health A 69: 1797-1804. <http://dx.doi.org/10.1080/15287390600630237>

Yang, C, -Y; Chang, C, -C; Tsai, S, -S; Chuang, H, -Y; Ho, C, -K; Wu, T, -N. (2003). Arsenic in drinking water and adverse pregnancy outcome in an arseniasis-endemic area in northeastern Taiwan. Environ Res 91: 29-34. <http://dx.doi.org/10.1016/S0013-9351(02)00015-4>

Yang, C, -Y; Chiu, H, -F; Chang, C, -C; Ho, S, -C; Wu, T, -N. (2005). Bladder cancer mortality reduction after installation of a tap-water supply system in an arsenious-endemic area in southwestern Taiwan. Environ Res 98: 127-132. <http://dx.doi.org/10.1016/j.envres.2004.07.013>

Yang, CY; Chiu, HF; Wu, TN; Chuang, HY; Ho, SC. (2004). Reduction in kidney cancer mortality following installation of a tap water supply system in an arsenic-endemic area of Taiwan. Arch Environ Health 59: 484-488. <http://dx.doi.org/10.1080/00039890409603430>

Yang, T, seYen; Hsu, LI; Chen, H, uiChi; Chiou, HY, i; Hsueh, Y, uMei; Wu, MM; Chen, C, hiL; Wang, YH; Liao, Y, aT; Chen, CJ, en. (2013). Lifetime risk of urothelial carcinoma and lung cancer in the arseniasis-endemic area of Northeastern Taiwan. J Asian Earth Sci 77: 332-337. <http://dx.doi.org/10.1016/j.jseaes.2013.03.023>

Yildiz, A; Karaca, M; Biceroglu, S; Nalbantcilar, MT; Coskun, U; Arik, F; Aliyev, F; Yiginer, O; Turkoglu, C. (2008). Effect of chronic arsenic exposure from drinking waters on the QT interval and transmural dispersion of repolarization. J Int Med Res 36: 471-478. <http://dx.doi.org/10.1177/147323000803600311>

Yorifuji, T; Tsuda, T; Doi, H; Grandjean, P. (2011). Cancer excess after arsenic exposure from contaminated milk powder. Environ Health Prev Med 16: 164-170. <http://dx.doi.org/10.1007/s12199-010-0182-x>

Yoshikawa, M; Aoki, K; Ebine, N; Kusunoki, M; Okamoto, A. (2008). Correlation between the arsenic concentrations in the air and the SMR of lung cancer. Environ Health Prev Med 13: 207-218. <http://dx.doi.org/10.1007/s12199-008-0032-2>

Yu, G; Sun, D; Zheng, Y. (2007). Health effects of exposure to natural arsenic in groundwater and coal in China: An overview of occurrence. Environ Health Perspect 115: 636-642. <http://dx.doi.org/10.1289/ehp.9268>

Yuan, Y; Marshall, G; Ferreccio, C; Steinmaus, C; Liaw, J; Bates, M; Smith, AH. (2010). Kidney cancer mortality: Fifty-year latency patterns related to arsenic exposure. Epidemiology 21: 103-108. <http://dx.doi.org/10.1097/EDE.0b013e3181c21e46>

Yuan, Y; Marshall, G; Ferreccio, C; Steinmaus, C; Selvin, S; Liaw, J; Bates, MN; Smith, AH. (2007). Acute myocardial infarction mortality in comparison with lung and bladder cancer mortality in arsenic-exposed region II of Chile from 1950 to 2000. Am J Epidemiol 166: 1381-1391. <http://dx.doi.org/10.1093/aje/kwm238>

Zhang, C; Mao, G; He, S; Yang, Z; Yang, W; Zhang, X; Qiu, W; Ta, N; Cao, L; Yang, H; Guo, X. (2013). Relationship between long-term exposure to low-level arsenic in drinking water and the prevalence of abnormal blood pressure. J Hazard Mater 262: 1154-1158. <http://dx.doi.org/10.1016/j.jhazmat.2012.09.045>

Zierold, KM; Knobeloch, L; Anderson, H. (2004). Prevalence of chronic diseases in adults exposed to arsenic-contaminated drinking water. Am J Public Health 94: 1936-1937. <http://dx.doi.org/10.2105/ajph.94.11.1936>
